# MR perfusion measurements on pharyngeal tumors: Comparison of quantification strategies

**DOI:** 10.1120/jacmp.v5i4.2009

**Published:** 2004-11-24

**Authors:** Volker Hietschold, Thomas Kittner, Michele Schreyer, Steffen Appold, Bettina Beuthien‐Baumann, Michael Laniado

**Affiliations:** ^1^ University of Technology Dresden Department of Diagnostic Radiology Fetscherstr. 74, D–01307 Dresden; ^2^ University of Technology Dresden Clinic of Radiation Therapy and Radiation Oncology Fetscherstr. 74, D–01307 Dresden; ^3^ University of Technology Dresden Clinic of Nuclear Medicine/PET Center Rossendorf Fetscherstr. 74, D–01307 Dresden Germany

**Keywords:** magnetic resonance imaging, perfusion

## Abstract

For the case of pharyngeal carcinomas, the clinical value as well as the stability of several evaluation methods of MR tomographic perfusion measurement are compared. Eighteen patients suffering from histologically proven squamous cell carcinomas were investigated by MR tomography (1.5 T, 0.2 mmol/kg Gd‐DTPA) prior to and during radiation therapy. Perfusion measurements were performed using a double‐echo FLASH sequence. Parameters describing regional blood flow, blood volume, mean transit time, and interstitial concentration of contrast medium (CM) were calculated, applying seven different combinations of correction approaches (separating the shortening of $T_{1}$ and $T_{2}^{*}$, arterial input function (AIF), and tumor shunts). Their correlations to MR independent tumor physiological parameters were analyzed (metabolic activity measurements using ^18^F‐FDG‐PET, polarographical ${\text{pO}}_{2}$ measurement, tumor volume). Significant improvements of the correlation between perfusion‐dependent and other tumor physiological parameters could be achieved by decoupling the shortening of $T_{1}$ and $T_{2}^{*}$ and by applying of the tumor shunt model. Deconvolution from the AIF deteriorated the correlation. Therefore, the elimination of the $T_{1}$ shortening due to interstitial CM proves to be essential for MR perfusion measurements on contrast medium uptaking lesions. Depending on the measurement conditions (temporal resolution, signal‐to‐noise ratio), the consideration of the AIF can even make the results significantly worse by introducing additional measuring errors.

PACS numbers: 87.61.‐c

## I. INTRODUCTION

Every year in the Federal Republic of Germany, approximately 7700 males and 2300 females develop malignant tumors of the oral cavity and the pharynx. This is equivalent to 5% of all malignant tumors in men and 1% in women. Depending on the stage, these tumors are treated by surgery, radiotherapy with or without chemotherapy, or by a combination of the different therapeutic approaches. Since the oxygen supply of the tumor tissue is one of the determinants of the response to radiation therapy,^(^ ^1^ ^,^ ^2^ ^)^ a correlation between oxygenation parameters and therapy response is to be expected and is described in the literature.^(^ ^3^ ^)^

In the initial stage of tumor growth, up to approximately 1 mm^3^ (equivalent to about 10^6^ cells), the tumor cells can be fed via diffusion alone. Larger tumor volumes entail the growth of additional blood vessels (tumor neoangiogenesis).^(^ ^4^ ^)^ These vessels lack smooth muscle cells, so leakages, blind endings, as well as arteriovenous shunts appear. The irregular arrangement of the endothelial cells or their locally complete absence results in an increased permeability of the vessel walls.

These irregularities of the vessel walls may change the permeability of the walls for contrast media (CM) compared to healthy tissue, which can result in an increased $T_{1}$ related signal enhancement. The modified vessel geometry, on the other hand, can influence parameters that are accessible by susceptibility contrast‐based MR perfusion measurements. However, interstitially stored CM cause susceptibility inhomogeneities and thus contributes to shortening of the $T_{2}^{*}$ relaxation time as well. Therefore, measurements of the time course of CM concentrations in tumor tissue based on changes of both $T_{1}$ and $T_{2}^{*}$ are justified from the physiological as well as from the physical points of view.

For quantification of the dynamics of contrast enhancement, a number of more or less sophisticated approaches are conceivable. As a rule, more detailed modeling requires the determination of a larger number of parameters and thus will be more sensitive to systematic and stochastic errors of the MR measurement compared to simpler and more robust approaches. For given measurement conditions, it is useful to determine the postprocessing and evaluation strategies that are clinically most relevant. In the present paper, the most robust MR perfusion parameters are determined for given measurement and injection conditions. Furthermore, the value of compensations for the influence of interstitially stored CM as well as a deconvolution from the arterial input function are investigated. Therefore, correlations between MR perfusion parameters and other MR‐independent tumor physiological parameters are analyzed.

### II. METHODS

Blood perfusion, which is accessible by MR perfusion parameters, is a determinative parameter for oxygenation. Using MR tomography, oxygenation can be described by means of perfusion parameters. In a study of the physiology of pharyngeal tumors (approved by the ethics commission of the University Hospital “Carl Gustav Carus” at the Technical University of Dresden),^(^ ^5^ ^)^ the invasively measured extracellular oxygen partial pressure in the tumor tissue was determined. Its polarographical measurement is the gold standard for the description of tumor oxygenation (many other methods, such as oxygen tension, interstitial fluid pressure, and vital tumor fraction, do not correlate to the measured data by means of ${\text{pO}}_{2}$ probes^(^ ^6^ ^)^). Glucose metabolism activity was described by means of positron emission tomography (PET). The therapy response was considered by measurement of the change of the metabolic activity as well as by volumetry of the tumor (including its metastases).

### A. Measurements

#### A.1 Patients

Twenty patients with primarily nonoperable pharyngeal or laryngeal squamous cell carcinoma were included in the study. Measurements were performed at several stages of the therapy (Table 1). In total, data from 69 MR investigations were collected. Two patients were excluded from further evaluations (see section II.D). The mean age of the 18 included patients was 55 years and 10 months (minimum 43 years and 9 months, maximum 67 years and 6 months). All patients suffered from histologically proven squamous cell carcinomas of the head and neck.

**Table 1:** Flow chart of investigations within the study about the physiology of pharyngeal tumors

Date	Endoscopy	Eppendorf ${\text{pO}}_{2}$	pet	MRI
1. initial	X	X	X	X
2. after 25 Gy	X			X
3. after 50 Gy	X	X	X	X
4. end of radiation therapy	X			X
5. 3 months after radiation therapy	X		X	X
6. 6 months after radiation therapy	X		X	X
7. 12 months after radiation therapy	X		X	X
8. 18 months after radiation therapy	X		X	X
9. 24 months after radiation therapy	X		X	X

#### A.2 Magnetic resonance tomography

The measurements were performed on a 1.5 T whole‐body tomograph (Siemens Magnetom Vision). The CM dynamics were measured by means of a double‐echo FLASH sequence (2D‐FLASH, $T_{E,1}=\;15\;\text{ms}$, $T_{E,2}=\;35\;\text{ms}$, $T_{R}=\;50\;\text{ms}$, α=30°, temporal resolution approximately 5 s, Scanmatrix 60×128, one slice only). The tumor volumes were determined on the base of 3D FLASH measurements (3D FLASH, $T_{E}=\;6\;\text{ms}$, $T_{R}=32\;\text{ms}$, α=40°; 0.8 mm‐slice thickness interpolated).

For the dynamic studies, a paramagnetic contrast medium (Gd‐DTPA, Magnevist, Schering) was injected as a bolus using a CM injector (SPECTRIS, Medrad). The amount of CM per body weight (0.4mL/kg being equivalent to 0.2mmol/kg) as well as the flow (2mL/s) were kept constant. The injection took approximately 11 s to 18 s, which is equivalent to approximately 2 or 3 scans. In order to contain bolus broadening, 20 mL of a physiological solution of sodium chloride was injected immediately after the CM. Prior to the injection, 10 baseline scans were acquired, the first of which was discarded in order to measure within a stationary state of the net magnetization. Forty scans were recorded covering approximately 2.5 min after the start of the bolus injection. Tumor volumes were determined by slice‐by‐slice manual drawing of the lesion boundaries from the $T_{1}$ volume data (Kittner et al.^(^ ^5^ ^)^). Total tumor volume comprised the primary tumor metastasis as well as necrotic areas.

#### A.3 Oxygen partial pressure

Measurements of the (mainly extracellular) intratumoral ${\text{pO}}_{2}$ were performed using a polarographic needle (KIMOC‐6650, Eppendorf), which automatically penetrated into the tissue in steps of 0.5 mm.^(^ ^7^ ^)^ The measurements were controlled sonographically and visually. An average of 108 measurement points per tumor were obtained (range 47 to 193). For statistical evaluations, the median and the relative number of values with a ${\text{pO}}_{2}$ of less then 5 mmHg (hypoxic fraction) were used.

#### A.4 Metabolic activity

The metabolic activity of the tumors was determined by means of PET. For this, fluor‐deoxyglucose (FDG) marked with the positron emitter F18 was injected in a quantity equivalent to 300 MBq. Imaging was performed 60 min after injection using a PET scanner of the ECAT EXACT HR+type (Siemens) (Beuthien‐Baumann^(^ ^5^ ^)^). Regions of interest (ROIs) were defined by threshold‐based segmentation at 50% to 70% of the maximum tracer uptake of the tumor under visual control. A tumor volume was derived from the sum of these ROIs. These volumes differ by principle from the volumes determined by means of MR, since they do not cover necrosis but include only viable regions (viable tumor volume). From the mean values of the activities in the volumes segmented as tumor tissue, the standard uptake value (SUV) was calculated:

(1) $$\text{SUV}=\frac{\text{activity}\;\text{in}\;\text{ROI}\;(\text{Bq}/g)\times \text{body}\;\text{weight}(g)}{\text{injected}\;\text{activity}\;(\text{Bq})}$$

The sequence of the investigations described is shown in Table 1.

### B. Evaluation

#### B.1 Determination of concentrations of contrast medium

##### B.1.1 Interstitial contrast medium

**Figure 1:**
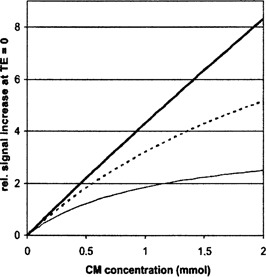
Dependence of the signal intensity of an idealized FLASH sequence $\left(T_{E}=0\right)$ on the concentration of Gd‐DTPA in tissue with a $T_{1}$ of 1000 ms, to be measured at a repetition time $T_{R}$ of 24 ms. Flip angle α=15° (—), α=20° (—), and α=30° (—).

In 1948, Bloembergen, Purcel, and Pound^(^ ^8^ ^)^ derived an approximately linear relationship between the CM concentration and the change of the spin‐lattice relaxation rate. Under certain conditions, which can be derived from the signal intensity equation of FLASH sequences (Eq. (2) below) and the relaxivity of the applied contrast medium, the relative change of the signal intensity of a $T_{1}$ only weighted FLASH sequence can be treated as being proportional to the interstitial CM concentration $C_{\text{tis}}$ (Fig. 1):

(2) $$C_{\text{tis}}\sim S=\rho \;\text{sin}(\alpha )\;\text{exp}(\frac{‐T_{E}}{T_{2}^{*}})\frac{1‐\text{exp}(‐T_{R}{/}_{T_{1}})}{1‐\text{cos}(\alpha )\text{exp}(‐T_{R}{/}_{T_{1}})}$$

##### B.1.2 Intravascular contrast medium

The intravascular CM concentration $C_{\text{ves}}$ in good approximation can be treated as being proportional to the change of the relaxation rate ${\Delta R}_{2}$.^(^ ^9^ ^–^ ^11^ ^)^ From this, for sequences with an exponential dependence of the intensity on the $T_{2}^{*}$ relaxation time according to Eq. (3) below, in the case of the measurement of an intensity series using a $T_{2}^{*}$ weighted single‐echo sequence follows Eq. (4):

(3) $$S\sim \text{exp}(‐\overset{T_{E}}{\;}/\underset{T_{2}^{*}}{\;})$$

(4) $$C_{\text{ves}}(t)=‐\frac{k}{T_{E}}\ln(\overset{S(t)}{\;}/\underset{S(t_{0})}{\;})$$

where $t_{0}$ is a time point prior to the beginning of CM injection (baseline).

##### B.1.3 Simultaneous CM uptake and passage through the capillary bed

If the CM leaks through the vessel walls, in highly vascularized tissues simultaneous shortening of $T_{1}$ and $T_{2}^{*}$ relaxation times will occur. Under the conditions of realistic echo times $\left(T_{E}>0\right)$ and finite repetition times $\left(T_{R}<\infty \right)$, this leads to a superimposition of the $T_{1}$ related signal increase due to contrast uptake and the $T_{2}^{*}$ related signal loss during the passage of the CM bolus. If the measurement is performed as a double‐echo sequence, these effects can be separated from each other.^(^ ^12^ ^–^ ^14^ ^)^

From the equations giving the signal intensities for spin echo and gradient echo, respectively, Eqs. (5) and (2), $T_{2}^{*}$ can be estimated:

(5) $$S=\rho \;\text{exp}(\overset{‐T_{E}}{\;}/\underset{T_{2}}{\;})(1‐\text{exp}(\overset{‐T_{R}}{\;}/\underset{T_{1}}{\;}))$$

(6) $$T_{2}^{*}=\frac{T_{E2}‐T_{E1}}{\text{ln}\left(S_{1}{/}_{S_{2}}\right)}$$

With the proportionality between CM concentration and change of the relaxation rate ${\Delta R}_{2}$ as postulated above, the intravascular CM concentration can be estimated according to the following:

(7) $$C_{\text{ves}}(t)=\frac{1}{r}\Delta R_{2}(t)=\frac{1}{r}\frac{\text{ln}\left(s_{1}(t){/}_{S_{2}(t)}\right)‐\text{ln}\left(S_{1}(0){/}_{S_{2}(0)}\right)}{T_{E2}‐T_{E1}}$$

If $T_{2}^{*}$ is known from Eq. (6), its influence on the signal intensity of the first echo can be eliminated by estimation of a hypothetical intensity for a sequence not underlying the influence of $T_{2}^{*}$, that is, with an echo time $T_{E}=0$:

(8) $$S_{\text{TE}‐0}(t)=\frac{S_{1}(t)}{\text{exp}\left(‐T_{E2}{/}_{T_{2}^{*}}\right)}=S_{1}(t){\left(\frac{S_{1}}{S_{2}}\right)}^{\frac{T_{E1}}{T_{E2}‐T_{E1}}}$$

### C. Modeling of the time course of the CM concentration

#### C.1 Uptake of contrast medium

After intravenous injection of a CM bolus, its concentration in the blood decreases approximately biexponentially.^(^ ^15^ ^)^

The duration of the bolus injection and its distribution in the blood pool are considered to be completed within an infinitely short time compared to the temporal resolution of the measurement. We describe the elimination of the CM from the blood pool with an elimination rate that is proportional to the CM concentration, which is an exponential decay. According to Weinmann et al.,^(^ ^16^ ^)^ this simplification compared to the biexponential model is justified, since the elimination from the blood pool is a slow process compared to the CM uptake.

The exchange of the CM between the blood and the tissue takes place independently of the direction and is proportional to the concentration difference. Therefore, it can be described by means of the first Fick diffusion law:

(9) $$\frac{\partial C}{\partial t}=‐D\;A\frac{\partial C}{\partial x}$$

Over a sufficiently short distance the concentration gradient is proportional to the difference between the concentrations in the blood and in the tissue. If the time course of the concentration in the blood after bolus injection of the CM is modeled as exponentially decaying, Eq. (9) can be transcribed as follows:

(10) $$\frac{\partial C}{\partial t}=‐D\;A\frac{\partial C}{\partial x}\sim ‐D\left(C‐C_{\text{max}}\;\text{exp}\left(‐\frac{t}{T}\right)\right)$$

The solution of this differential equation is

(11) $$C(t)\sim \left(\text{const}+\frac{C_{\text{max}}\;D}{DT‐1}\text{exp}\left(t\left(D‐\frac{1}{T}\right)\right)\right)\text{exp}(‐Dt)$$

The constant const results from the constraint C(0)=0 (at the beginning of the injection the tissue is free of contrast medium) as

(12) $$\text{const}=C_{\text{max}}\frac{C_{\text{max}}D}{1‐DT}$$

As shown above, under certain measuring conditions the relative change of the signal intensity can be assumed to be proportional to the change of the relaxation rate (simulation in Fig. 1). With the abbreviations

$$T_{w}=\frac{1}{D-1/T}=\frac{T}{TD-1}\text{and}\;T_{e}=1/D,$$

the relative change of the signal intensity can be formulated as^(^ ^17^ ^)^

(13) $$\frac{S}{S_{0}}=a\;\text{exp}\left(\frac{t_{0}‐t}{T_{w}}\right)\left\{1‐\text{exp}\left(\frac{t_{0}‐t}{T_{e}}\right)\right\}$$

where *a* is a proportionality factor that depends on $C_{\text{max}},D,T$, the measuring conditions, the area of the vessel walls, and their permeability $(t_{0}$ only generalizes the time axis to any arrival time of the CM in the tissue).

There exists a relation between this model function and the model developed by Brix et al.^(^ ^18^ ^)^:

(14) $$\frac{S}{S_{0}}=1+A\{v\left[\text{exp}(k_{\text{el}}\;t\prime )‐1\right]\text{exp}(‐k_{\text{el}}\;t)‐u\left[\text{exp}(k_{21}\;t\prime )‐1\right]\text{exp}(‐k_{21}\;t)\}$$

The latter simplifies for an extremely short duration of the infusion τ since all terms containing $t^{\prime }$ become constant:

(15) $$\frac{S}{S_{0}}=1+B\{\text{exp}(‐k_{21}\;t)‐C\;\text{exp}(‐k_{el}\;t)\}$$

where constants *B* and *C* depend on the measuring and injection conditions as well as on $k_{21}$ and $K_{\text{el}}$. With reasonable accuracy, $1/T_{e}=D$ is equivalent to the exchange rate between blood and the lesion $k_{21}$, and $1/T_{w}=D-1/T$ for not too large *t* is in sufficient approximation equivalent to the elimination rate of the CM from the blood pool $k_{\text{el}}$ of the Brix model.

Considering the washout term in Eq. (13) by multiplication, $S/S_{0}\geq 0$ is guaranteed for any time t≥0. $S/S_{0}<0$ is possible in Eq. (15), but it is not possible physically, since signal loss due to $T_{2}^{*}$ shortening is not included in the model.

#### C.2 Passage of contrast medium

In a first approximation, the intravascular CM concentration was assumed to be proportional to the relative signal loss of the second echo of the double‐echo FLASH sequence. Aiming for a robust quantification, only the maximum relative signal loss was considered.

The changes in the relaxation rate, which were determined by applying Eqs. (4) and (7), respectively, and which should be proportional to the CM concentration, were subject to a numerical deconvolution from the arterial input function using the singular value decomposition (SVD) algorithm.^(^ ^19^ ^,^ ^20^ ^)^

The shortening of $T_{2}^{*}$ due to interstitially stored CM was taken into consideration empirically as proposed by the author in Ref. 21. The interstitial CM concentration was assumed to be proportional to the relative change of the signal intensity at $T_{E}=0$. Its contribution to ${\Delta R}_{2}^{*}$ was assumed to fulfill the (simplifying) condition of an intravascular CM concentration to be zero at the end of the dynamic study.

A simplified gamma variate function (GVF) according to Eq. (16) below or in a simplified form (Eq. (17)) was fit to the concentration time curves. Equation (17) contains a term for the description of the development of an equilibrium concentration^(^ ^22^ ^)^:

(16) $$\begin{array}{ll} C(t)=A\;t^{B}\;\text{exp}(‐t{/}_{C}) & t\leq T_{a}\\ C(t)=D & t>T_{a} \end{array}$$

(17) $$C(t)=A\;t{/}_{t_{p}^{2}}\text{exp}\left(\frac{‐t}{t_{p}}\right)+B\left(1‐\exp\left(\frac{‐Ct}{t_{p}}\right)\right)$$

From the model curves, parameters were derived according to the indicator dilution theory.^(^ ^23^ ^)^. The area of the gamma variate function (which describes the CM bolus without recirculation) can be derived from Eq. (17) as shown in Eq. (18):

(18) $$A_{\text{GVFsimplified}}=\int_{0}^{\infty }A\frac{t}{t_{p}^{2}}\text{exp}\left(‐\frac{t}{t_{p}}\right)dt={\left[\frac{‐A(t_{p}^{2}+t_{p}t)}{t_{p}^{2}}\exp\left(‐\frac{t}{t_{p}}\right)\right]}_{0}^{\infty }=a$$

The mean transit time (MTT) as the first moment of the GVF is given by the parameter $t_{p}$:

(19) $${\text{MTT}}_{\text{simplified}}=\frac{\int_{0}^{\infty }t\;A\frac{t}{t_{p}^{2}}\text{exp}\left(‐\frac{t}{t_{p}}\right)dt}{\int_{0}^{\infty }A\frac{t}{t_{p}^{2}}\text{exp}\left(‐\frac{t}{t_{p}}\right)dt}=\frac{at_{p}}{a}=t_{p}$$

Using *a,* which is proportional to the regional blood volume, a measure for the regional blood flow ${\text{rBF}}_{\text{simplified}}$ can be formulated:

(20) $${\text{rBF}}_{\text{simplified}}=\frac{a}{t_{p}}$$

A prerequisite for this approach is that the injection conditions are comparable. As a robust method, the area under ${\Delta R}_{2}^{*}(t)$ was determined by numeric integration $\left(\Sigma {\Delta R}_{2}^{*}(t)\Delta t\right)$ as well.

For the gamma variate function according to Eq. (16) without break‐off, the above‐mentioned parameters can be calculated from Eqs. (21) to (23) accordingly:

(21) $$A_{\text{GVF}}=\frac{A}{C^{1+B}}\Gamma (1+B)$$

(22) $${\text{MTT}}_{\text{OVF}}=\frac{C^{1+B}}{C^{2+B}}\frac{\Gamma (2+B)}{\Gamma (1+B)}=C(2+B)$$

(23) $${\text{rBF}}_{\text{GVF}}=\frac{A}{(2+B)C^{2+B}}\Gamma (1+B)$$

The chaotic vessel structure of the intratumoral capillary bed with, for example, arteriovenous shunts was considered according to Ref. 24, modeling of ${\Delta R}_{2}^{*}(t)$ by the sum of two gamma variate functions.

## D. Correlation of MR perfusion parameters to other tumor‐physiological parameters

For reasons related to the signal‐to‐noise ratio, all analysis of time courses of signal intensities or measures derived from them were performed using ROIs. They were defined by an experienced radiologist (Kittner^(^ ^5^ ^)^). Modeling and quantification were done using a program that was developed under IDL (Research Systems, Inc.). For the statistical analysis, Excel (Microsoft) with extensions written under VBA was used.

**Figure 2:**
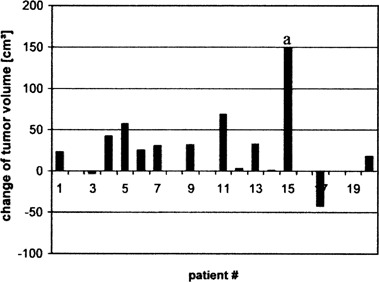
Changes of tumor volumes measured by means of MRI after 50 Gy of radiation therapy. The data of the patient labeled with “a” were excluded from the evaluations discussed below.

**Figure 3:**
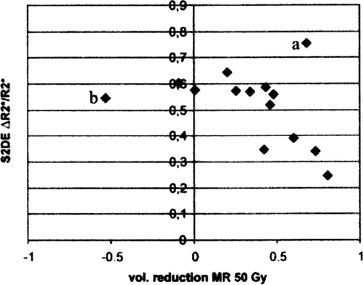
Correlation of the tumor volume reduction measured with MRI against the maximum relative change of $R_{2}^{*}$. The data points of the patients which were excluded from the evaluations discussed below are labeled “a” and “b.”

The data of two patients were conspicuous concerning several parameters. In one case, strong progression of tumor growth during the radiation therapy was observed. The other patient impressed with both maximum initial tumor size and volume reduction under therapy (Fig. 2). Since both patients give rise to outlying data points in the correlation analysis (e.g., Fig. 3), they were excluded from the evaluations, which are discussed in section III in order to guarantee a sample sufficiently homogeneous for the methodical question described above. Nevertheless, since these exclusions were made during the data analysis process, the results of evaluations with and without these data will be presented in Table 2, Table 3, and Table 4.

**Table 2:** Sample size and coefficients of determination of the correlations between $T_{1}$ related MR parameters and other tumor physiological parameters. Results after exclusion of two outliers (bold) and from the complete data set (italics).

Parameter	S1 rel. enhancement	S1 time constant	S1 rel. slope	S1DE rel. enhancement	S1DE time constant	S1DE rel. slope
${\text{pO}}_{2}$ median	**17 / 0.02**	**17 / 0.00**	**17 / 0.01**	**17 / 0.00**	**17 / 0.02**	**17 / 0.02**
initial	*19 / 0.03*	*19 / 0.00*	*19 / 0.01*	*19 / 0.00*	*19 / 0.02*	*19 / 0.02*
${\text{pO}}_{2}$ median	**24 / 0.02**	**24 / 0.01**	**24 / 0.01**	**24 / 0.00**	**24 / 0.02**	**24 / 0.00**
initial+50Gy	*27 / 0.03*	*27 / 0.01*	*27 / 0.02*	*27 / 0.00*	*27 / 0.02*	*27 / 0.00*
${\text{pO}}_{2}<5\;\text{mmHg}$	***17 / 0.01***	**17 / 0.06**	**17 / 0.00**	**17 / 0.00**	**17 / 0.00**	**17 / 0.03**
initial	*19 / 0.02*	*19 / 0.03*	*19 / 0.00*	*19 / 0.00*	*19 / 0.00*	*19 / 0.01*
${\text{pO}}_{2}<5\;\text{mm Hg}$	**24 / 0.01**	**24 / 0.00**	**24 / 0.00**	**24 / 0.00**	**24 / 0.00**	**24 / 0.01**
initial+50Gy	*27 / 0.01*	*27 / 0.00*	*27 / 0.00*	*27 / 0.00*	*27 / 0.00*	*27 / 0.00*
SUV mean	**16 / 0.03**	**16 / 0.02**	**16 / 0.04**	**16 / 0.01**	**16 / 0.02**	**16 / 0.18**
initial	*18 / 0.08*	*18 / 0.00*	*18 / 0.02*	*18 / 0.08*	*18 / 0.01*	*18 / 0.28* **
SUV mean	**24 / 0.03**	**24 / 0.15** *	**24 / 0.13** *	**24 / 0.05**	**24 / 0.03**	**24 / 0.19** **
initial+50Gy	*27 / 0.06*	*27 / 0.08*	*27 / 0.09*	*27 / 0.12* *	*27 / 0.02*	*27 / 0.24* **
SUV mean	**30 / 0.02**	**30 / 0.13** *	**30 / 0.13** *	**30 / 0.05**	**30 / 0.04**	**30 / 0.21** **
	*35 / 0.05*	*35 / 0.06*	*35 / 0.08*	*35 / 0.09* *	*35 / 0.03*	*35 / 0.25* **
volume MR	**18 / 0.01**	**18 / 0.13**	**18 / 0.00**	**18 / 0.02**	**18 / 0.01**	**18 / 0.01**
initial	*20 / 0.03*	*20 / 0.15*	*20 / 0.11*	*20 / 0.00*	*20 / 0.01*	*20 / 0.00*
volume MR	**39 / 0.00**	**39 / 0.03**	**39 / 0.01**	**39 / 0.02**	**39 / 0.00**	**39 / 0.00**
	*43 / 0.01*	*43 / 0.01*	*43 / 0.01*	*43 / 0.00*	*43 / 0.00*	*43 / 0.01*
vol. reduction MR	**12 / 0.01**	**12 / 0.06**	**12 / 0.00**	**12 / 0.15**	**12 / 0.00**	**12 / 0.08**
50 Gy	*14 / 0.00*	*14 / 0.01*	*14 / 0.02*	*14 / 0.00*	*14 / 0.00*	*14 / 0.00*
volume PET	**16 / 0.14**	**16 / 0.03**	**16 / 0.06**	**16 / 0.17**	**16 / 0.01**	**16 / 0.00**
initial	*18 / 0.19* *	*18 / 0.08*	*18 / 0.02*	*18 / 0.25* **	*18 / 0.02*	*18 / 0.01*
volume PET	**31 / 0.06**	**31 / 0.03**	**31 / 0.09**	**31 / 0.11** *	**31 / 0.00**	**31 / 0.00**
	*35 / 0.10* *	*35 / 0.01*	*35 / 0.06*	*35 / 0.18* **	*35 / 0.00*	*35 / 0.04*
vol. reduction PET	**11 / 0.12**	**11 / 0.00**	**11 / 0.11**	**11 / 0.18**	**11 / 0.00**	**11 / 0.36** *
50 Gy	*13 / 0.12*	*13 / 0.01*	*13 / 0.14*	*13 / 0.17*	*13 / 0.01*	*13 / 0.19*

* $R^{2}\neq 0$ with significance levels ≤0.1

** $R^{2}\neq 0$ with significance levels ≤0.05

**Table 3:** Sample sizes and coefficients of determination of the correlations between MR perfusion parameters derived from the time course of the second echo and other parameters. Results after exclusion of two outliers (bold) and from the complete data set (italics).

Parameter n / R2	S2 rel. signal loss	S2 flow	S2 MTT	S2 RBV	S2 rBV num.
${\text{pO}}_{2}$ median	**17 / 0.09**	**17 / 0.06**	**17 / 0.05**	**17 / 0.19** *	**17 / 0.07**
initial	*19 / 0.05*	*19 / 0.06*	*19 / 0.04*	*19 / 0.13*	*19 / 0.05*
${\text{pO}}_{2}$ median	**24 / 0.07**	**24 / 0.07**	**24 / 0.06**	**24 / 0.02**	**24 / 0.04**
initial+50Gy	*27 / 0.04*	*27 / 0.07*	*27 / 0.06*	*27 / 0.01*	*27 / 0.04*
${\text{pO}}_{2}<5\text{mmHg}$	**17 / 0.11**	**17 / 0.18**	**17 / 0.13**	**17 / 0.13**	**17 / 0.05**
initial	*19 / 0.07*	*19 / 0.19* *	*19 / 0.09*	*19 / 0.10*	*19 / 0.04*
${\text{pO}}_{2}<5\;\text{mmHg}$	**24 / 0.10**	**24 / 0.21** **	**24 / 0.15** *	**24 / 0.02**	**24 / 0.04**
initial+50Gy	*27 / 0.07*	*27 / 0.18* **	*27 / 0.09*	*27 / 0.02*	*27 / 0.04*
SUV mean	**16 / 0.14**	**16 / 0.03**	**16 / 0.00**	**16 / 0.02**	**16 / 0.01**
initial	*18 / 0.16*	*18 / 0.00*	*18 / 0.02*	*18 / 0.04*	*18 / 0.00*
SUV mean	**24 / 0.11**	**24 / 0.11**	**24 / 0.06**	**24 / 0.01**	**24 / 0.00**
initial+50Gy	*27 / 0.11*	*27 / 0.08*	*27 / 0.01*	*27 / 0.01*	*27 / 0.00*
SUV mean	**30 / 0.14** *	**30 / 0.11** *	**30 / 011** *	**30 / 0.00**	**30 / 0.00**
	*35 / 0.12* *	*35 / 0.05*	*35 / 0.06*	*35 / 0.01*	*35 / 0.01*
volume MR	**18 / 0.16**	**18 / 0.31** **	**18 / 0.20** *	**18 / 0.22** *	**18 / 0.06**
initial	*20 / 0.09*	*20 / 0.23* **	*20 / 0.22* **	*20 / 0.13*	*20 / 0.03*
volume MR	**39 / 0.01**	**39 / 0.02**	**39 / 0.04**	**39 / 0.02**	**39 / 0.01**
	*43 / 0.00*	*43 / 0.01*	*43 / 0.05*	*43 / 0.01*	*43 / 0.00*
vol. reduction MR	**12 / 0.25**	**12 / 0.08**	**12 / 0.03**	**12 / 0.02**	**12 / 0.10**
50 Gy	*14 / 0.00*	*14 / 0.18*	*14 / 0.06*	*14 / 0.08*	*14 / 0.03*
volume PET	**16 / 0.04**	**16 / 0.09**	**16 / 0.09**	**16 / 0.05**	**16 / 0.03**
initial	*18 / 0.02*	*18 / 0.05*	*18 / 0.17*	*18 / 0.03*	*18 / 0.02*
volume PET	**31 / 0.00**	**31 / 0.00**	**31 / 0.02**	**31 / 0.02**	**31 / 0.00**
	*35 / 0.00*	*35 / 0.00*	*35 / 0.01*	*35 / 0.01*	*35 / 0.00*
vol. reduction PET	**11 / 0.00**	**11 / 0.03**	**11 / 0.17**	**11 / 0.01**	**11 / 0.06**
50 Gy	*13 / 0.01*	*13 / 0.06*	*13 / 0.05*	*13 / 0.04*	*13 / 0.05*

* $R^{2}\neq 0$ with significance levels ≤0.1

** $R^{2}\neq 0$ with significance levels ≤0.05

**Table 4:**
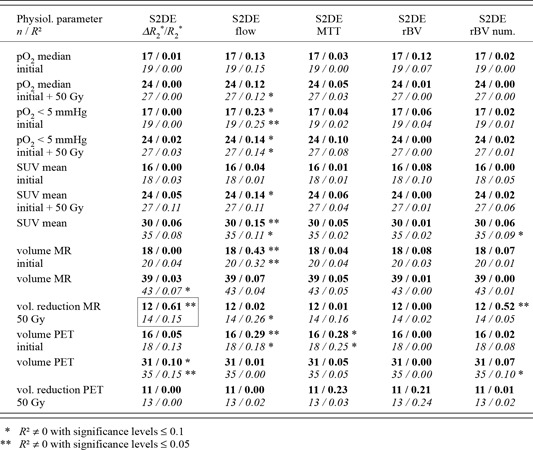
Sample sizes and coefficients of determination of the correlations between the estimations of $T_{2}^{*}$ derived from the quotient of the signal intensities of both echos and other tumor‐physiological parameters. Results after exclusion of two outliers (bold) and from the complete data set (italics).

Linear regressions were calculated for the combinations of perfusion‐independent parameters (tumor volume derived from PET and MR, volume reduction after 50 Gy of radiation therapy, ${\text{pO}}_{2}$, hypoxic fraction, SUV, survival time) with all parameters derived from the CM dynamics. In order to select the most appropriate reference parameter for the evaluation of the clinical relevance of the MR perfusion parameters, the maximum coefficient of determination of these regressions being significantly different from zero on the 5% level was determined. Further, the influence of the corrections described above on the correlation coefficients to this reference parameter and to other parameters was evaluated.

In the explorative data analysis presented in this paper, multiple tests were applied to the same sample. In the case of no correlation between any parameters under investigation, the chance probability of finding at least one correlation to be incorrectly significant increases with the number of tests. At a significance level of 0.05, in every 20th test, the null hypothesis “no correlation” would be rejected by chance. In order to analyze a sample for *any* correlation, the significance level of each of the single tests would have to be divided roughly by the number of tests (Bonferroni correction) to keep the *overall* significance level constant. A consequence of this correction is an increased type two error (to overlook an existing correlation). However, the significance of one single correlation between two parameters should not depend on what else was done with the sample.^(^ ^25^ ^)^ So corrections according to Bonferroni or Scheffe, for example, would not be appropriate for the design of the presented study.

## III. RESULTS AND DISCUSSION

As an example, results of the correlation analysis between CM‐independent and CM‐dynamic MR parameters are shown in Table 2 for the $T_{1}$ related parameters, in Table 3 for perfusion evaluations based on the time course of the intensity of the second echo alone, and in Table 4 for evaluations based on $T_{2}^{*}$ estimations according to Eq. (6). Here, S1 and S2 represent the signal intensities of the first and second echo of the double‐echo FLASH sequence respectively. The label DE identifies data that result from the decoupling of the influences of $T_{1}$ and $T_{2}^{*}$ as described above (Eqs. (7) and (8)).

Within the $T_{1}$ related MR parameters, the strongest correlations were observed with the mean standard uptake value of FDG (Table 2). The elimination of the $T_{2}^{*}$ shortening (labeled with DE for double‐echo correction in the table) results in increased coefficients of determination at decreasing significance levels. This overall tendency is especially clear for the relative slope and is violated for the time constant of enhancement. The relative slope after double‐echo correction turned out to be the most relevant MR parameter that is the relative asymptotic signal increase divided by the time constant ($a/T_{e}$ in Eq. (13)). The weaker improvement of correlations for the relative enhancement as well as the lack of improved correlations for the time constants might be caused by the fact that simultaneous changes of these parameters in opposite directions during the curve fit procedure tend to compensate each other's influence on the curve shape within the time window of the measurements.

**Figure 4:**
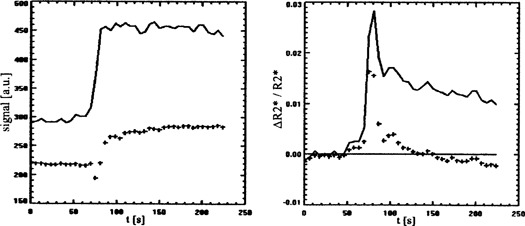
Double‐echo correction: On the left time course of the signals at $T_{E}=15$ (+++) and at $T_{E}=0$ (—), on the right ${\Delta R}_{2}^{*}$ from the signal at $T_{E}=35$ (+++) and from the quotient of the signals at $T_{E}=15$ and $T_{E}=35$ (—) (patient #1 prior to therapy).

The estimation of ${\Delta R}_{2}^{*}$ according to Eq. (7) (double‐echo correction) instead of Eq. (4) avoids the occurrence of apparent negative CM concentrations due to the violation of the condition $T_{1}=\text{const}$. As a result, clearly more plausible concentration time courses are observed (Fig. 4).

Among the correlations significantly differing from zero, the highest coefficient of determination was found between the change of the total tumor volume after 50 Gy and the maximum relative change of $R_{2}^{*}$ calculated from both echoes according to Eq. (7) (framed in Table 4, N=12). Frequently, significant correlations of MR perfusion parameters to the median standard uptake value or to the hypoxic fraction (N=24) are observed as well (each measured contemporary to the MR investigation). Furthermore, significant correlations to the initial total tumor volume (N=18) occurred. For other physiological parameters only occasionally significant correlations were found.

**Figure 5:**
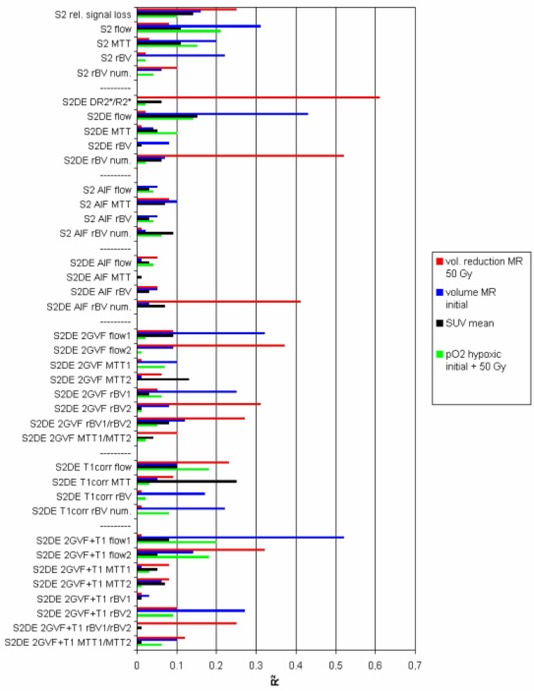
Coefficients of determination of the correlations of MR‐tomographic perfusion parameters to other physiological parameters. The perfusion parameters are grouped according to the applied corrections.

Figure 5 shows the coefficients of determination of all investigated MR perfusion parameters against the four most relevant physiological parameters as mentioned above. (The minimum values for statistical significance are summarized in Table 5.)

**Table 5:** Minimum coefficients of determination at significance levels α for rejection of the null hypothesis R2=0 for the correlations shown in Fig. 5.

Parameter	*N*	α	$R^{2}$
SUV mean	30	0.05	0.145
SUV mean	30	0.1	0.100
${\text{pO}}_{2}$ hypoxic (initial+50Gy)	24	0.05	0.187
${\text{pO}}_{2}$ hypoxic (initial+50Gy)	24	0.1	0.128
volume MR (initial)	18	0.05	0.264
volume MR (initial)	18	0.1	0.179
vol. reduction MR (50 Gy)	12	0.05	0.451
vol. reduction MR (50 Gy)	12	0.1	0.299

The strongest correlation is observed between the MR tomographically determined volume reduction and perfusion parameters which were calculated using very simple and thus very robust algorithms (maximum relative change of $R_{2}^{*}$, numeric integral over ${\Delta R}_{2}^{*}$, both after double‐echo correction). After deconvolution from the arterial input function (with and without application of the double‐echo correction), the lowest coefficients of determination were found. The compensation of ${\Delta R}_{2}^{*}$ for the interstitial component does not have a favorable effect on its correlation to independent parameters. The hypoxic fraction as well as the pretherapeutic tumor volume seems to correlate to flow parameters.

The correlations of the blood volume parameters derived from curve fits (rBV) and from simple numeric integrations (rBV num) differ considerably from each other. Here, the stronger correlation of the rBV num values to the volume reduction after 50 Gy should be a consequence of the influence of the interstitial CM concentration on this parameter, the relative enhancement with and without double‐echo correction strongly correlates to the volume reduction as well (Table 2).

Modeling the intravascular concentration time courses with two gamma variate functions seems to have a favorable effect on the observed correlations. Flow calculations in particular profit from this model.

**Figure 6:**
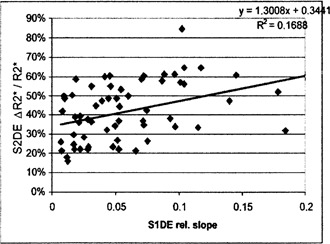
Correlation of the relative slope of the signal intensity corrected to $T_{E}=0$ (and so depending only on changes in $T_{1}$) and the maximum relative change of the spin‐spin relaxation rate $R_{2}^{*}$.

For the validation of correction approaches, the degree of correlation between blood perfusion and CM uptake into the interstitium is relevant. It might be best validated by comparing the relative slope of the signal intensity corrected to $T_{E}=0$ (S1DE) to the relative change of $R_{2}^{*}$ for two reasons: these measures most strongly correlate to independent physiological parameters, respectively, and $T_{1}$ and $T_{2}^{*}$ related effects are separated from each other in these measures. The correlation between both measures observed in the present study is significant (p=0.1%, Fig. 6).

## IV. CONCLUSIONS

Maximum correlations of MR perfusion parameters to independent physiological parameters were observed after calculation of changes in $T_{2}^{*}$ from both signals of a double‐echo sequence. This observation, together with the improved plausibility of concentration time curves applying Eq. (7) instead of Eq. (4) (avoiding apparent negative CM concentrations), leads us to conclude that the elimination of the influence of the $T_{1}$ shortening on the MR tomographical determination of perfusion parameters proves to be the most important among all correction methods analyzed in this study. The plausibility of the time courses of the intravascular CM concentrations is considerably improved by this step (elimination of the decay of the concentration up to negative values; Fig. 4). Because of the correlation between $T_{1}$ shortening due to interstitially stored contrast medium and $T_{2}^{*}$ related perfusion parameters (Fig. 6) as well as due to the opposite directions of the influences of shortening of $T_{1}$ and $T_{2}^{*}$ on the signal intensity, this correction leads to an increased correlation between some MR parameters and independent reference parameters. On the other hand, the compensation of ${\Delta R}_{2}^{*}$ for the contribution of the interstitial CM results in a reduction of the measured effect due to the high correlation between CM uptake and perfusion, since here both CM concentrations act in the identical direction (Fig. 5).

**Figure 7:**
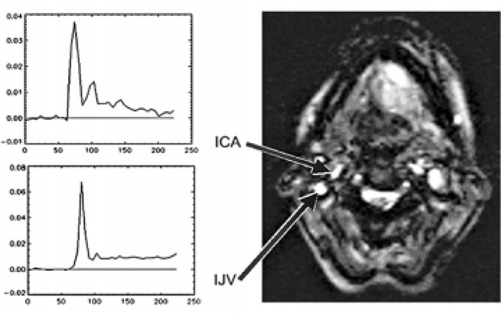
Variability of the arterial input function in dependence on the pixel selection. Although localized behind in the blood pathway, the bolus passage through the venous pixels appears narrower compared to the passage through the arterial pixels.

At least under the given measuring conditions, if the estimates of the intravascular CM concentrations are deconvolved from the arterial input function, the introduced additional uncertainties prevail over the theoretically expected benefit of this correction. One reason for this may be the poor temporal resolution of the perfusion measurements in this study (about 5 s) and the deconvolution problems resulting from it. Further aspects concerning this are the systematic error of the determination of low signal intensities due to an additive noise component and the influence of the pixel selection on the course of the AIF (Fig. 7).

The slightly improved correlation between perfusion and other physiological parameters after modeling the intravascular CM concentrations with two gamma variate functions has to be interpreted cautiously. in the light of the proneness of the deconvolution from the AIF to be influenced by other parameters, it is not possible to decide to which extent a second gamma variate function merely models recirculation effects. But the present data at least suggest that the application of the two‐peak model and the compensation of ${\Delta R}_{2}^{*}$ for the contribution of interstitially stored CM together could allow one to work out more clearly the correlation between flow (derived from the first GVF) and initial tumor volume.

Considerations concerning the significance show that the tendencies discussed here need to be proved by an increased sample size.

## Supporting information

Supplementary Material
