# Supplementary material for: MR perfusion measurements on pharyngeal tumors: Comparison of quantification strategies
Source: J Appl Clin Med Phys. 2004 Nov 24;5(4):96–111. doi: 10.1120/jacmp.v5i4.2009 (PMC5723518; doi:10.1120/jacmp.v5i4.2009)
Supplement: Supplementary file 1 — Supplementary Material [file ACM2-5-096-s001.pdf]

Technische Universität Dresden

Aspekte einer quantitativen Gewebecharakterisierung mittels Kontrastmitteldynamik in der Kernspintomographie

Dr. rer. nat. Volker Hietschold

der Fakultät Elektrotechnik und Informationstechnik der Technischen Universität Dresden

zur Erlangung des akademischen Grades

Doktoringenieur habilitatus

(Dr.-Ing. habil.)

vorgelegte Habilitation

Diese Veröffentlichung stimmt mit der Habilitationsschrift

“Aspekte einer quantitativen Gewebecharakterisierung mittels Kontrastmitteldynamik in der Kernspintomographie“

vorgelegt von Dr. rer. nat. Volker Hietschold
an der Fakultät Elektrotechnik und Informationstechnik
der Technischen Universität Dresden
überein.

Vorsitzender der Habilitationskommision:	Prof. Dr-Ing. habil. Adolf Finger
Gutachter:	Prof. Dr. med. habil. Dipl.-Ing. Rüdiger Poll<br>Prof. (i.R.) Dr. med. habil. Heinrich Platzbecker<br>Prof. Dr. med. Dr. rer. nat. Wolfhard Semmler
Probevorlesung:	19. April 2004

Inhalt

1 Einleitung 1

2 Zielstellung 4

2.1 Bedarf – Klinischer Hintergrund und messtechnische Aspekte 4
2.1.1 MR-Mammographie 4
2.1.1.1 Kontrastmittel-Aufnahme 4
2.1.1.2 Perfusion 5
2.1.2 Hirnläsionen 6
2.1.2.1 Infarkt 6
2.1.2.2 Tumoren und Metastasen 9
2.1.3 Pharyngeale Tumoren 9
2.1.4 Zusammenfassung 10

2.2 Stand der Technik und methodische Grundlagen 10
2.2.1 Prinzip der kernspintomographischen Bildgebung 10
2.2.2 Einige grundlegende Sequenztechniken (Auswahl) 16
2.2.2.1 Spinecho-Sequenz 21
2.2.2.2 Gradientenecho-Sequenz 22
2.2.2.3 Inversion Recovery 25
2.2.2.4 Das Turbo-Prinzip 26
2.2.2.5 Echo Planar Imaging (EPI) 27
2.2.2.6 Präparation 29
2.2.3 Gerätetechnik für die MR-Tomographie 30
2.2.4 Physikalische Effekte bei Anwendung paramagnetischer Kontrastmittel 33
2.2.4.1 Eigenschaften der Kontrastmittel 33
2.2.4.2 Aufnahme eines paramagnetischen Kontrastmittels (statisch) 36
2.2.4.3 Aufnahme eines paramagnetischen Kontrastmittels (dynamisch) 37
2.2.4.4 Passage eines paramagnetischen Kontrastmittels 40
2.2.4.5 Verbleib eines paramagnetischen Kontrastmittels im Gefäßbett 42
2.2.5 Andere MR-tomographische Kontrastmittel 42
2.2.5.1 Klassifikation nach Verteilungsverhalten im Körper 42
2.2.5.2 Körpereigene Substanzen als Kontrastmittel 43
2.2.6 Andere physikalische Parameter zur Gewebecharakterisierung 45
2.2.6.1 Relaxometrie 45
2.2.6.2 Diffusion 49
2.2.6.3 Volumetrie 51

2.3 Gerätetechnische Präzisierung der Aufgabenstellung 54

3 Messung kontrastmitteldynamischer Parameter 59

3.1 Modellierung der Signalverläufe 59
3.1.1 Kontrastmittelaufnahme 59
3.1.2 Indikator-Verdünnungs-Theorie 62
3.1.3 Modelle für die Zeitabhängigkeit der intravasalen KM-Konzentration 65

3.2 Entkopplung einander überlagernder Effekte 66
3.2.1 Trennung von Aufnahme- und Passage-Effekten durch Einsatz intravasaler Kontrastmittel 66
3.2.2 Entkopplung über Modellannahmen 67
3.2.3 Vorinjektion von Kontrastmittel 69
3.2.4 Zusammenfassung 70

4 Material und Methoden 71
4.1 Patienten 71
4.2 Benutzte Geräte und Sequenzen 71
4.3 Messmethoden 72
4.4 Auswerte-Werkzeuge 74
5 Ergebnisse 75
5.1 Mathematische Entkopplung der T_1- und T_2^*-Verkürzung nach Doppelecho-Messung für gefäßwand-gängige Kontrastmittel 75
5.2 Empirische Trennung von perfusions- und KM-Aufnahme-bedingter T_2^*-Verkürzung 76
5.2.1 Methode 78
5.2.2 Ergebnisse 80
5.2.3 Diskussion 83
5.3 MR-Mammographie 83
5.3.1 MR-Mammographie bei 0.5 Tesla und Übertragung statistischen Wissens zwischen unterschiedlichen Messbedingungen 84
5.3.2 Modell-Entwicklung und Spezifitäten von Modellparametern der dynamischen Kernspin-Mammographie 86
5.4 Hirntumoren und -metastasen 90
5.5 Pharyngeale Tumoren 91
5.5.1 Auswerte-Strategie für die MR-Daten 92
5.5.2 Ergebnisse 95
5.5.3 Diskussion 103
5.6 Darstellung von Blutgefäßen – Koronarangiographie 105
5.6.1 Ausgangssituation 105
5.6.2 Optimierung der Messmethode 106
5.6.3 Ergebnisse 109
5.6.3.1 Optimierung des Flipwinkels 109
5.6.3.2 Simulationen 110
5.6.3.3 Messungen 113
5.6.4 Diskussion 115

6 Fehlerbetrachtungen 116
6.1 Fehlerfortpflanzung 116
6.2 Intravasale Kontrastmittel-Konzentration 116
6.3 Arterielle Inputfunktion 117
6.4 Schätzung niedriger Signalintensitäten 121
6.5 Funktionelle Bilder 123
6.6 Diskussion 128
7 Diskussion und gerätetechnische Schlussfolgerungen 130
7.1 Globale Einschätzung 130
7.2 Spezielle Aspekte 130
7.2.1 Kontrastmittelaufnahme 130
7.2.2 Perfusion 131
7.2.3 Bildgebung 132
8 Ausblick 134
8.1 Einige Charakteristika der bisherigen Entwicklung der bildgebenden medizinischen Diagnostik 134
8.2 Zukünftige Entwicklung: Versuch einer Prognose: 136
Anhang 138
Literaturverzeichnis 138
Verzeichnis der verwendeten Symbole 154
Verzeichnis der verwendeten Begriffe und Abkürzungen 156

1 Einleitung

Im Rahmen der medizinischen Diagnostik findet eine Vielzahl bildgebender Verfahren Anwendung, die auf den unterschiedlichsten physikalischen Prinzipien basieren [40]. Das bekannteste ist sicher die klassische Röntgenaufnahme, in der der Grauwert jedes Bildpunktes im wesentlichen durch das Integral der linearen Absorptionskoeffizienten über die Gerade zwischen Ausgangspunkt der Strahlung und Ort der Strahlungsregistrierung bestimmt ist. Als grundlegende Effekte werden in der Bildgebung neben der Absorption und Streuung aber auch die Reflektion (z. B. Sonographie) sowie die Emission (z. B. nuklearmedizinische Verfahren, Kernspintomographie) benutzt und unterschiedliche Eigenschaften biologischer Gewebe bezüglich der gemessenen Parameter in Form von Bildern dargestellt.

Bei verschiedenen bildgebenden Verfahren wird der Kontrast zwischen gewissen Strukturen durch die Gabe von Kontrastmitteln manipuliert. Als elementare Beispiele seien hier die lokale Erhöhung des Röntgenabsorptionskoeffizienten durch Füllung von Hohlräumen mit jod- oder bariumbasierten Medien (z. B. Darstellung des Magen-Darm-Traktes, Fistelfüllungen) oder die Erzeugung eines im Röntgenbild nachweisbaren Absorptionsunterschiedes zwischen Körperflüssigkeit und dem umgebenden Gewebe durch Injektion von Kontrastmitteln (Angiographie, Myelographie) genannt. Bei der auf die Messung lokaler linearer Röntgen-Schwächungskoeffizienten hinauslaufenden Computertomographie, bei der (um den Preis einer spürbar höheren Strahlenbelastung für den Patienten) erheblich geringere Absorptionsunterschiede und damit Gewebearten unterschieden werden können, kommt auch ein vermehrter Übertritt von in die Blutbahn injiziertem Kontrastmittel in Gewebe zur Darstellung. Der Nachweis einer solchen Kontrastmittelaufnahme durch Gewebe ist je nach Körperregion u. U. ein deutliches Indiz für pathologische Prozesse, da z. B. im Gehirn Moleküle von der für Kontrastmittel typischen Größe normalerweise das Gefäßbett nicht verlassen ("Blut-Hirn-Schranke"). Bei anderen Fragestellungen wird der durch Veränderungen der Blutversorgung beeinflusste Zeitablauf der Kontrastmittel-Aufnahme zur Diagnosefindung benutzt (z. B. Angiome, Gefäßneubildung in Tumoren, Infarkte).

In der Sonographie kann der auf Grund des Doppler-Effektes geschwindigkeitsabhängig frequenzverschobene Signalanteil aus den Blutgefäßen durch Injektion eines im wesentlichen aus stabilisierten kleinsten Gasblasen beruhenden Kontrastmittels erhöht werden.

Anders als bei den auf der Absorption von Röntgenstrahlen basierenden bildgebenden Verfahren beeinflussen in der Kernspintomographie mehrere physikalische Effekte den Bildkontrast (z. B. Dichte von Wasserstoff-Atomen, T_1, T_2, T_2^*-Relaxationszeiten, chemische Verschiebung, Fluss- und Diffusionseffekte). Daraus ergibt sich, dass durch geeignete Messstrategien auch eine deutlich größere Informationsmenge vollständig "nicht-invasiv", d. h. ohne Verletzung des Körpers und ohne Einbringung von Substanzen in diesen, gewonnen werden kann. In den letzten Jahren wird jedoch zunehmend davon Gebrauch gemacht, solche Effekte durch die Gabe von unterschiedlichen, sehr gut verträglichen und/oder mehr oder weniger gewebespezifischen Kontrastmitteln deutlicher zur Darstellung kommen zu lassen (z. B. kontrastverstärkte MR-Angiographie, Perfusionsuntersuchungen, leberspezifische Kontrastmittel).

Das Ziel gerätetechnischer Weiterentwicklungen auf dem Gebiet der Bildgebung im Rahmen der medizinische Diagnostik muss generell die Verbesserung des Kosten-Nutzen-Verhältnisses für den Patienten sein. Unter Kosten sind hierbei sowohl ökonomische Aspekte als auch gesundheitliche Risiken diagnostischer Maßnahmen zu verstehen, während der Nutzen als therapie-beeinflussender Informationsgewinn aufgefasst wird. Aus dieser Definition des Nutzens folgt, dass in Abhängigkeit von der klinischen Fragestellung und den bildbestimmenden physiologischen Parametern unterschiedlich hohe Anforderungen an die Bildqualität gestellt werden müssen. Zur Optimierung des Kosten-Nutzen-Verhältnisses beim Einsatz bildgebender Verfahren können Weiterentwicklungen auf folgenden Bereichen beitragen:

1. Verbesserung der räumlichen und/oder zeitlichen Auflösung und/oder von Kontrasten, Untersuchung größerer Volumina pro Zeiteinheit

2. Reduktion des Schädigungspotenzials bildgebender Methoden (absolut oder relativ zum Informationsgewinn)

3. Erschließung weiterer physiologischer und/oder physikalischer Effekte als Informationsträger für physiologische Zustände bzw. Prozesse und deren Umsetzung in Bildinformation (z. B. neue MR-Sequenzen, Kontrastmittel mit spezifischem Akkumulations- und/oder Transportverhalten, neue Prinzipien der Bilderzeugung)

4. Informations-Aufbereitung: Verknüpfung von verschiedene physiologische Aspekte betreffender Information (z. B. Image Fusion, Triggerung, Multispektral-Analyse, fMRI); Modellbildung und Parametrisierung, Bildanalyse (CAD – computer aided diagnosis)

5. Erschließung neuer oder Verbesserung existierender Kombinationen aus Bilderzeugung und therapeutischen Maßnahmen (z. B. im Rahmen der interventionellen Radiologie)

Das Entwicklungspotenzial der auf ionisierender Strahlung beruhenden Methoden ist bezüglich der räumlichen Auflösung durch die Beziehung zwischen Signal-Rausch-Verhältnis und Patientendosis physikalisch begrenzt, wenn auch heute noch nicht völlig ausgeschöpft. Die Beschleunigung der Daten-Akquisition in der Röntgen-Computer-tomographie durch Einführung der Multislice-CT ermöglichte beispielsweise einen erheblichen Qualitätssprung in der nicht-invasiven Darstellung der Blutgefäße. Die niedrigen Nachweisgrenzen von Methoden, die auf der Emission von Strahlung durch in den Körper eingebrachte Tracer beruhen, lassen für die nahe Zukunft erwarten, dass – beispielsweise durch die Kopplung von Tracern an monoklonale Antikörper oder die Markierung von Substraten – sehr spezifische Zelleigenschaften oder Stoffwechsel-Spezifika der Bildgebung zugänglich werden („molecular imaging“).

Bezüglich der Kernspintomographie darf nach gegenwärtigem Kenntnisstand davon ausgegangen werden, dass deren Schädigungspotenziale durch die Wahl geeigneter Untersuchungsparameter sowie durch die Formulierung von patientenbezogenen Kontraindikationen für die Methode gut beherrscht werden können. Allerdings resultieren hieraus auch physiologische Grenzen für gewisse gerätetechnische Weiterentwicklungen. So dürften die Möglichkeiten der Steigerung von zeitlichen Magnetfeld-Veränderungen sowie der Exposition der Patienten mit elektromagnetischen Wechselfeldern bereits ausgeschöpft sein. Dessen ungeachtet erfuhr die MRT in allen

oben aufgezählten thematischen Bereichen in den vergangenen Jahren bemerkenswerte Weiterentwicklungen – resultierend in einer wachsenden Zahl von sowohl diagnostischen als auch therapeutischen Anwendungsgebieten.

Die im Rahmen der vorliegenden Schrift durchgeführten Arbeiten sind den Punkten 1, 3 und insbesondere 4 der obigen Klassifizierung zuzuordnen.

2 Zielstellung

Bei verschiedenen Geweben äußern sich unterschiedliche physiologische bzw. pathophysiologische Zustände in unterschiedlichen Bedingungen der Sauerstoff- und Nährstoffversorgung durch das Blut. Dies kann sowohl die Blutgefäß-Architektur als auch den Aufbau der Gefäßwände betreffen. Sowohl über morphologische als auch funktionelle Aspekte der Blutversorgung sind Informationen ableitbar, wenn die räumliche und zeitliche Verteilung eines injizierten Indikators analysiert wird.

In der Kernspintomographie werden u.a. paramagnetische Kontrastmittel eingesetzt. Diese beeinflussen die signalintensitäts-bestimmenden Spin-Gitter- und Spin-Spin-Relaxationszeiten T_1 und $T_2^{(*)}$ in jeweils unterschiedlich großen Regionen um die Kontrastmittel-Moleküle. Daraus leitet sich das Potenzial der kontrastmittel-gestützten MRT ab, parallel Aussagen über das Kapillarbett von Geweben als auch über den Stoffaustausch zwischen Kapillaren und umgebendem Gewebe zu gewinnen. Dieses Potenzial stellt gleichzeitig eine Fehlerquelle dar, indem diese Informationen einander kontaminieren.

Die vorliegende Arbeit enthält Beiträge zur Messung und quantitativen Analyse der Aufnahme und Passage paramagnetischer Kontrastmittel in der Kernspintomographie sowie zur durch Kontrastmittel unterstützten Bildgebung. Diese werden im Kontext weiterer quantitativer Verfahren dargestellt. Hierbei wird versucht, unter Wahrung physikalischer und mathematischer Exaktheit die Verbindung zwischen methodischen Aspekten und deren klinischer Anwendbarkeit anklingen zu lassen.

2.1 Bedarf – Klinischer Hintergrund und messtechnische Aspekte

Im Anfangsstadium eines Tumorwachstums bis zu einer Größe von ca. 1 mm³ (entsprechend ca. 10^6 Zellen) können die Tumorzellen über Diffusion versorgt werden. Größere Volumina bedingen das Wachstum zusätzlicher Blutgefäße (Tumor-Neoangiogenese) [28]. Die neu entstandenen Gefäße besitzen keine glatten Muskelzellen, dafür treten Leckagen, blinde Endungen sowie arterio-venöse Shunts auf. Die irreguläre Anordnung der Endothelzellen bzw. deren stellenweise komplettes Fehlen äußern sich in einer erhöhten Permeabilität der Gefäßwand.

Die Unregelmäßigkeiten der Gefäßwände können zu einer im Vergleich zu gesundem Gewebe veränderten Durchlässigkeit von Kontrastmittel führen, welche sich ggflls. in einer verstärkten T_1-bedingten Signalzunahme niederschlägt. Die veränderte Gefäßgeometrie dagegen kann die der suszeptibilitätskontrast-basierten MR-Perfusionsmessung zugänglichen Parameter beeinflussen. Allerdings führt auch interstitiell eingelagertes KM zu Suszeptibilitäts-Inhomogenitäten.

2.1.1 MR-Mammographie

2.1.1.1 Kontrastmittel-Aufnahme

Kontrastmitteldynamische Messungen erfolgen in der MR-Mammographie (MRM) vorwiegend mit T_1-gewichteten Sequenzen, wobei auf Grund der höheren erreichbaren zeitlichen Auflösung FLASH-Sequenzen in 2D- und 3D-Technik angewandt werden. In vielen Fällen ergibt sich durch die Bewertung des Zeitverlaufes der KM-Aufnahme ein diagnostischer Gewinn verglichen mit der statischen Bewertung des Signalzuwachses

nach KM-Gabe [65]. Die Zeitauflösung der dynamischen Messungen wird zwischen 20 s und 90 s gewählt Die Quantifizierung dieses Verlaufes erfolgt mit recht unterschiedlichem Aufwand: von qualitativer Klassifikation des Kurvenverlaufes [25] bis hin zu pharmakokinetischen Modellierungen (z. B. [17, 27], siehe auch Abschnitt 3.1.1). Knopp et al. [104] zeigten, dass in Analogie zur Analyse der intravasalen KM-Konzentration auch bei der geringen zeitlichen Auflösung von 25 s in der MR-Mammographie die Berücksichtigung zumindest von Parametern der arteriellen Inputfunktion (zumindest Zeitpunkt des Maximums, evtl. auch arterielle Maximal-konzentration) die diagnostische Wertigkeit verbessern kann. Müller-Schimpfle et al. [127] verglichen die Unterscheidbarkeit maligner von benignen Läsionen einerseits anhand des relativen Signalzuwachses 1 Minute nach Injektion und des Anstieges einer Geraden zwischen den Intensitäten zu den Zeitpunkten 2 Minuten p.i. und 10 Minuten p.i. und andererseits anhand pharmakologischer Parameter nach Brix [17]. Die pharmakologische Modellierung zeigte keinen klinisch verwertbaren Vorteil.

Die Therapiekontrolle bei der Behandlung des Mamma-Karzinoms stellt ebenfalls einen Anwendungsbereich der MR-Mammographie dar. Rieber et al. [145] beobachteten bei Patienten, die präoperativ eine Chemotherapie erhielten, eine Abnahme des relativen Signalzuwachses nach KM-Gabe - in Einzelfällen bis hin zu fehlender KM-Aufnahme. Dem prognostischen Wert dieser Aussage steht die Gefahr gegenüber, dass das präoperative Tumorvolumen als zu klein dargestellt wird.

Leach [115] stellt in einer Übersichtsarbeit verschiedene Studien zur Korrelation zwischen kontrastmitteldynamischen Parametern und histopathologischen Daten zusammen. Mehrfach wird hier über einen Zusammenhang zwischen der Gefäßdichte (MVD – microvessel density) und dem Signalzuwachs kurze Zeit nach Injektion berichtet. Frouge et al. [[12] in [115]] identifizierten mittels Faktoranalyse drei unabhängig voneinander aufladende Komponenten. Die schnellste KM-Aufnahme (Maximum 40 s p.i., danach schneller Abfall) assoziieren sie mit der Konzentration von Arteriolen und Reparaturprozessen innerhalb des Tumors. Die zweite Komponente (Maximum 2 min p.i., langsamerer Abfall) wird den Tumor-Kapillaren zugeschrieben. Eine dritte Komponente (Maximum 2 min p.i., kein Abfall) wird von den zitierten Autoren mit fibrotischem Gewebe assoziiert. Dagegen fanden Stomper et al. [[14] in [115]] keinen Zusammenhang zwischen Gefäßdichte einerseits und Geschwindigkeit von KM-Aufnahme oder –Auswaschung andererseits. Dies wird in Übereinstimmung zur schlechten Unterscheidbarkeit zwischen malignen Mammaläsionen und Fibroadenomen gesehen. Furuta et al. [[13] in [115]] fanden sowohl eine Korrelation zwischen Gefäßdichte und KM-Aufnahme als auch zwischen histologischem Grad der Läsion einerseits und steilstem Abschnitt der KM-Aufnahmekurve sowie Gefäßdichte andererseits. Allerdings schätzt Leach [115] diese Arbeit wegen ihrer geringen Zeitauflösung als kritisch ein.

2.1.1.2 Perfusion

Die je nach Patientengut recht niedrige Spezifität der Verfahren zur Mamma-Diagnostik gibt Anlass zur Suche nach weiteren, von der KM-Aufnahme der Läsion mehr oder weniger unabhängigen Parametern. Seit etwa 1997 wird die diagnostische Wertigkeit von T_2^*-basierten Perfusionsmessungen untersucht (Kuhl et al. [110]). Während z. B. von Kuhl et al., Kvistad et al. [112] und auch von der Dresdener Arbeitsgruppe [146] ein diagnostischer Nutzen gefunden wurde, wird der Zugewinn an verwertbarer Information von anderen Autoren (z. B. Dannert et al [25]) eher kritisch betrachtet.

Das messtechnische Vorgehen besteht in der Regel darin, innerhalb einer diagnostischen Sitzung zunächst nach einer Bolus-Injektion eines paramagnetischen Kontrastmittels mittels T_1-gewichteter dynamischer Messung der gesamten Mamma(e) die Morphologie, das Zeitverhalten der Kontrastmittel-Aufnahme sowie ggflls. Multizentrizität zu analysieren und ca. 10 bis 20 Minuten nach dieser Injektion während einer zweiten Injektion den first pass des KM-Bolus mittels T_2^*-gewichteter Messung aufzunehmen.

Eine Ursache für die unterschiedlichen Ergebnisse der Gruppen könnte in der Beeinflussung des maximalen relativen Signalverlustes während der Boluspassage durch die gleichzeitig auftretende T_1-Verkürzung liegen. Wenn man die prozentuale Wichtung einer Sequenz nach einem Parameter einer Idee von Elster et al. [33] folgend als Verhältnis des partiellen Differentialquotienten der Signalintensität nach diesem Parameter zur Summe der partiellen Differentialquotienten nach T_R, T_E und ρ formuliert und die Signalintensitäten aller benutzten Sequenzen als Gleichung (15) folgend annimmt, ergeben sich für die zitierten Arbeiten Wichtungen gemäß Tabelle 1:

Quelle	α	T_E	T_R	T_1-Wichtung	T_2^*-Wichtung
Kuhl et al. [110]	10°	35 ms	50 ms	10 %	48 %
Kvistad et al. [112]	10°	35 ms	54 ms	9 %	48 %
Dannert et al [25]	20°	36 ms	24 ms	25 %	40 %

Tabelle 1: Wichtungen von Sequenzen, die bei Perfusionsmessungen im Rahmen der MR-Mammographie eingesetzt wurden

Damit ist die von Dannert benutzte Sequenz doppelt so stark T_1-gewichtet wie die von Kvistad eingesetzte. Dies könnte dazu beigetragen haben, dass bei gleicher Feldstärke und KM-Dosis Kvistad prinzipiell maximale relative Signalverluste > 0 % fand, während von Dannert bei 11 von 23 Messungen kein Signalverlust beobachtet wurde.

2.1.2 Hirnläsionen

2.1.2.1 Infarkt

Für kontrastmitteldynamische Untersuchungen mittels MRT herrschen im Hirn besonders günstige Bedingungen. In den Kapillaren von intaktem Hirnparenchym bilden die Endothelzellen über „tight-junction-Proteine“ eine praktisch lückenlose Auskleidung des Gefäßlumens. Diese Transportbarriere dient der Aufrechterhaltung eines konstanten chemischen Milieus im Gehirn. In Kapillaren anderer Gewebe wird durch lockerere Verbände der Endothelzellen ein intensiver Stoffaustausch zwischen dem Blutkreislauf und dem jeweiligen Organ ermöglicht. Die Konsequenz dieser Blut-Hirn-Schranke für die MR-tomographische Bildgebung mit Kontrastmittel ist, dass dieses praktisch nicht in das Parenchym übertritt. Damit beschränkt sich die Signalveränderung nach Bolus-Injektion eines prinzipiell gefäßwandgängigen Kontrastmittels auf die Relaxationszeit-Verkürzungen, die durch die (zeitabhängige)

KM-Konzentration im Blut erklärt werden können. Insbesondere bedeutet das, dass Perfusionseinschränkungen beim frischen Hirninfarkt durch die suszeptibilitäts-inhomogenitäts-bedingten Signalabsenkungen der Messung mit Kontrastmitteln wie Gd-DTPA zugänglich sind. Von einer intakten Blut-Hirn-Schranke kann in den 8 Stunden, meist sogar innerhalb der ersten 24 Stunden nach Eintritt der Ischämie ausgegangen werden [32]. Es sind für diese klinische Fragestellung somit Messstrategien einsetzbar, die die Spin-Gitter-Relaxationszeit T_1 als konstant annehmen. Als minimale Zeitauflösung werden von Heiland et al. [61] mit Bezug auf Simulationen von Benner et al. [10] 1,5 Sekunden gefordert, wenn der Fehler der Parameter, die aus der Kurvenanpassung einer Gamma-Variate-Funktion bestimmt werden, unter 10 % bleiben soll. Mit EPI-Sequenzen kann unter Gewährleistung einer hinreichend guten Zeitauflösung von wenigen Sekunden das gesamte Hirn kontrastmitteldynamisch untersucht werden. Die Darstellung des Perfusions-Defizits als Reduktion der Amplitude der Spin-Spin-Relaxationsrate ΔR_2 mit EPI wurde z. B. von Kucharczyk et al. [109] über den zeitweiligen Verschluss der mittleren Hirnarterie (A. cerebri media) im Tierversuch nachgewiesen (Abbildung 1).

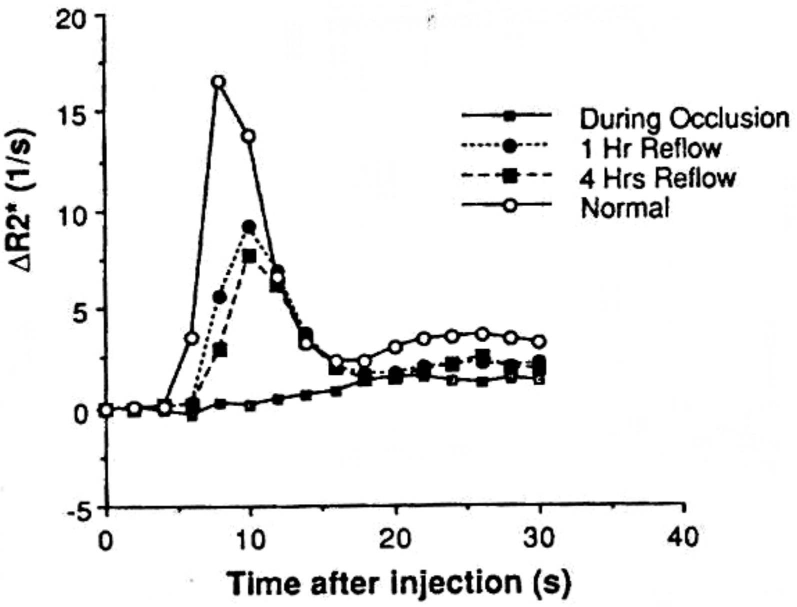

Abbildung 1: Passage eines KM-Bolus durch den parietalen Kortex im Katzenhirn. —o— Ausgangszustand; —■— während Verschluss der mittleren Hirnarterie; - - ● - - 1 Stunde nach Wiederherstellung des Flusses; — — ■ — — 4 Stunden nach Wiederherstellung des Flusses (aus [109]).

In einer Arbeit von Emerson et al. [35] wurde eine Korrelation zwischen maximaler Änderung von R_2* und dem mittels Xenon-Inhalation und SPECT bestimmten relativen zerebralen Blutfluss (rCBF) nachgewiesen. Es konnte – bei insgesamt 49 Patienten – ein zumindest im Mittel bezogen auf die Kontrollgruppe verringertes ΔR_2 bei vaskulären Erkrankungen und Alzheimer-Patienten nachgewiesen werden. Die Messungen erfolgten hier mit einer GRASS-Sequenz (Gradient Acquisition in Steady State), also einer Gradientenecho-Sequenz, bei der über Rephasierungen der Frequenz- und Phasencodierung in der Transversalebene ebenfalls ein stationärer Zustand der Magnetisierung erreicht wird, der bei kurzem T_R für Gewebe mit langer T_2 einen zusätzlichen Signalbeitrag (verglichen zu FLASH) liefert.

Reith et al. [142] zeigten mit einer auf eine Schicht beschränkten FLASH-Sequenz, dass sowohl bei chronischen zerebrovaskularen Erkrankungen als auch bei akuter Ischämie die Mean Transit Time und das relative regionale Blutvolumen erhöht sind – letzteres durch die Gefäßerweiterung, mit der die Minderversorgung des betroffenen Areals kompensiert werden soll.

Lythgoe et al. [119] zeigten bei Patienten mit einseitiger Stenose der Arteria carotis eine signifikante Korrelation zwischen dem Verhältnis der Mean Transit Time (MTT) im Hirnparenchym der betroffenen Region und der Gegenseite einerseits und dem Stenosegrad andererseits (Bestimmtheitsmaß $r^2 = 0,416$). Die Korrelation zwischen dem entsprechenden Verhältnis der ersten Momente der $\Delta R_2(t)$-Kurven (entspricht der MTT unter der Annahme einer deltafunktionsförmigen AIF) und dem Stenosegrad war deutlich geringer ($r^2 = 0,226$).

Klose et al. [102] zeigten mit einer EPI-Sequenz, deren zeitliche Auflösung durch Beschränkung auf drei Schichten auf 0,67 Sekunden eingestellt worden war in Übereinstimmung mit anderen Quellen, dass die sensitivsten Parameter für den Nachweis einer Durchblutungsstörung im Hirn die Parameter sind, die die Dauer der Boluspassage beschreiben (Zeitpunkt des maximalen relativen Signalverlustes, MTT). Weiterhin zeigten sie die hohe Korrelation von maximalem relativen Signalverlust, regionalem zerebralen Blutvolumen (rCBV) und ~fluss (rCBF) (Abbildung 2).

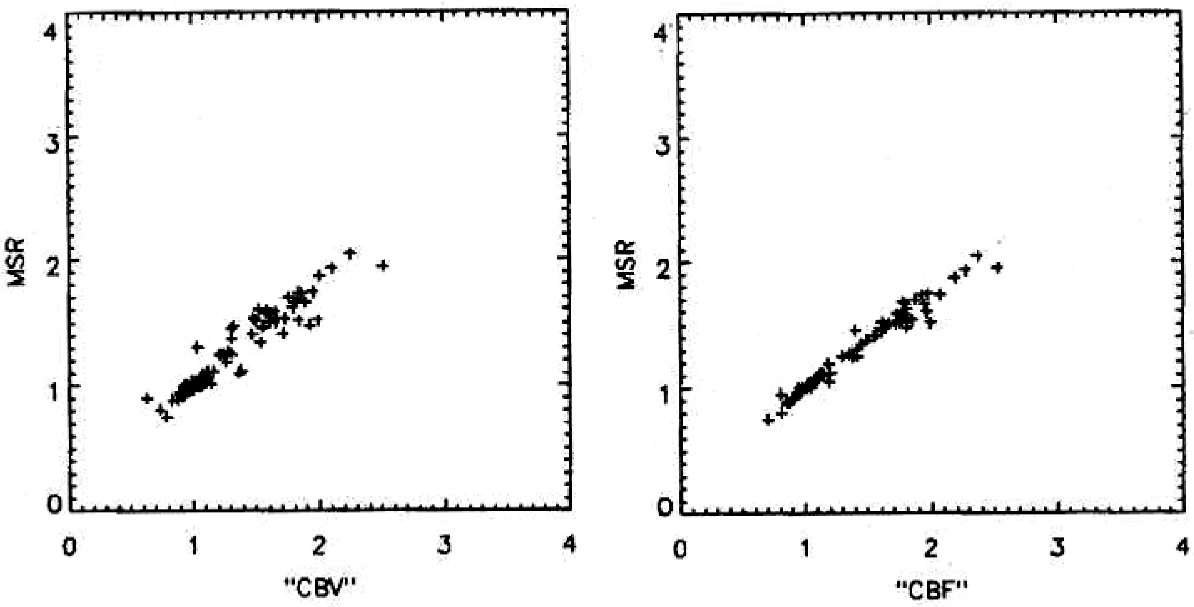

Abbildung 2: Korrelation zwischen dem maximalen relativen Signalverlust (max. rel. Signalreduktion – MSR) einerseits und berechnetem zerebralen Blutvolumen (CBV) und ~fluss (CBF) (aus [102])

Auf die Entfaltung von der arteriellen Inputfunktion verzichteten Klose et al., da die kurzzeitig sehr hohe KM-Konzentration in den großen Arterien bei EPI-Sequenzen zu einer zeitabhängigen Orts-Fehlcodierung führt (vgl. auch unsere Messungen in Abschnitt 6.3).

Ein über die Erfassung eines status quo hinausgehender Ansatz wird von Gückel et al. [50] beschrieben. rCBV und rCBF werden hier jeweils vor und nach Stimulation mit Acetazolamid bestimmt. Bei 8 von 10 Patienten konnte über den reduzierten Zuwachs des rCBF nach Stimulation auf eine eingeschränkte zerebrovaskuläre Reservekapazität in der betroffenen Region geschlossen werden. Allerdings unterliegen die Zuwächse in nicht betroffenen Arealen einer beträchtlichen und, wie die Autoren vermuten, nicht nur messtechnisch begründeten Schwankung.

Die auf der Indikator-Verdünnungs-Theorie basierende Berechnung von Absolutwerten für rCBV und rCBF [142, 50], je nach Interpretation auch der MTT ist prinzipiell an die Bestimmung der arteriellen Inputfunktion gebunden. Es muss jedoch bedacht werden, ob dieser Übergang zu physiologischen, also geräte- und sequenz-unabhängigen Werten angesichts der damit verbundenen zusätzlichen systematischen (vgl. [102, 16, 180]) und zufälligen Fehler mit einem klinischen Nutzen verbunden ist. Simonsen et al. [161] halten (zumindest bezüglich ihrer Tierversuche an Yorkshire-Schweinen) wegen des Partialvolumen-Effektes bei der Bestimmung der AIF deren Kurvenverlauf, nicht aber deren Amplitude für verwertbar und benutzen für eine Normierung von CBF und CBV die injizierten Kontrastmittelmengen.

2.1.2.2 Tumoren und Metastasen

Im Gegensatz zur Situation bei frischen Durchblutungsstörungen kann bei Tumoren und Metastasen in der Regel nicht von einer intakten Blut-Hirn-Schranke ausgegangen werden, da im Rahmen der Gefäßneubildung beim Tumorwachstum die lückenlose Auskleidung des Lumens durch Endothelzellen nicht gewährleistet ist. Damit ist die Voraussetzung einer näherungsweise konstanten T_1-Relaxationszeit über den Zeitraum der Boluspassage im Allgemeinen nicht erfüllt. Ausnahmen können z. B. bei langsam und infiltrativ wachsenden Hirntumoren wie z. B. niedriggradigen Astrozytomen auftreten (Wenz et al. [182]). Diese Autoren fanden keinen schlüssigen Zusammenhang zwischen den Perfusionsmessungen vor, während und nach Strahlentherapie von niedriggradigen Astrozytomen einerseits und der Prognose der Patienten andererseits. Obwohl Zellproliferation und Angiogenese in engem Zusammenhang zueinander stehen, wird das Schicksal der Patienten durch die die Therapie überlebenden entarteten Zellen bestimmt, die den Perfusionseffekt nicht unmittelbar beeinflussen.

2.1.3 Pharyngeale Tumoren

In der Bundesrepublik Deutschland erkranken jährlich etwa 7700 Männer und 2300 Frauen an bösartigen Neubildungen von Mundhöhle und Rachen (ICD 140-149). Das entspricht einem Anteil von 5% aller bösartigen Neubildungen bei Männern und von 1% bei Frauen. Die Behandlung dieser Tumoren erfolgt je nach Stadium mittels chirurgischer, chemo- oder radiotherapeutischer Maßnahmen bzw. Kombinationen verschiedener Therapien. Für die Optimierung der Lebensqualität der Patienten ist u.a. eine frühzeitige Bewertung der Wirksamkeit belastender therapeutischer Maßnahmen wichtig. Da die Sauerstoffversorgung des Tumorgewebes eine bestimmende Größe für die Therapierbarkeit mittels Strahlentherapie darstellt [55, 45], ist eine Korrelation zwischen Oxygenierungs-Parametern und Therapieresponse zu erwarten. Zur Beschreibung der Oxygenierung sind mittels Kernspintomographie Perfusionsparameter zugänglich. Deren diagnostische und prognostische Relevanz gilt es zu bewerten.

2.1.4 Zusammenfassung

Kontrastmittel-dynamische Untersuchungen mittels Kernspintomographie erscheinen sowohl vom physiologischen Hintergrund als auch von der Vielzahl der in der Literatur dokumentierten methodischen Untersuchungen bzw. Anwendungen her relevant für die Diagnostik und das Therapie-Monitoring sowohl vaskulärer als auch neoplastischer Veränderungen in verschiedenen Körperregionen. Sowohl vom gerätetechnischen als auch vom klinischen Standpunkt her ist es erforderlich, aus den Zeitverläufen von unter verschiedenen Messbedingungen ableitbaren Veränderungen kernspintomographischer Signale auf die räumliche und zeitliche Verteilung von Kontrastmittel in verschiedenen Gewebs-Kompartimenten zu schließen und daraus hinreichend zuverlässige Aussagen über physiologisch bedeutsame Eigenschaften der Gewebe abzuleiten. Hierfür ist die Analyse von Beziehungen messtechnisch zugänglicher Parameter untereinander sowie zu physiologischen Parametern erforderlich. Aus einer solchen Analyse sich ergebende Algorithmen zur Datengewinnung bzw. -nachverarbeitung müssen bezüglich ihres Einflusses auf systematische und zufällige Fehler des Ergebnisses bewertet werden. Aus Sicht des Autors ist es hierbei notwendig, auch das Verhältnis von methodischem Mehraufwand für die Verbesserung der methodischen Exaktheit kontrastmittel-dynamischer Parameter zum Zugewinn an klinisch verwertbarer Information zu bestimmen und im Sinne einer Empfehlung für die Anwendung und Interpretation kontrastmittel-dynamischer MR-Untersuchungen in der klinische Routine zu interpretieren.

2.2 Stand der Technik und methodische Grundlagen

2.2.1 Prinzip der kernspintomographischen Bildgebung

Die Grundlagen der kernspintomographischen Bildgebung mit Standard- (Spinecho, Gradientenecho) und schnellen Sequenzen (Turbo-Spinecho, Turbo-Gradientenecho, Echo Planar Imaging) sind in der Literatur ausführlich beschrieben (z. B. [175, 118, 131, 81, 176, 91, 51, 41, 52]).

Die physikalische Basis der magnetischen Kernresonanz (NMR = Nuclear Magnetic Resonance) ist das magnetische Moment, welches an die Eigenschaft des Spins gekoppelt ist (Spinquantenzahl $I \neq 0$). Die Spinquantenzahl lässt sich aus der Anzahl von Protonen p und Neutronen n ableiten: Insbesondere gelten folgende Regeln:

p gerade, n gerade	(gg-Kern):	I = 0
p gerade, n ungerade	(gu-Kern)	I ist ungeradzahliges Vielfaches von ½
p ungerade, n ungerade	(uu-Kern)	I ist ganzzahliges Vielfaches von 1
p ungerade, n gerade	(ug-Kern)	I ist ungeradzahliges Vielfaches von ½

Für die magnetische Quantenzahl m_I existieren $2 * I + 1$ verschiedene Werte $m_I = I, I-1, I-2,...-I$. Das magnetische Moment μ_z (in z-Richtung – siehe unten) des Kerns ergibt sich dann aus der magnetischen Quantenzahl m_I, dem gyromagnetischen Verhältnis γ und dem Planckschen Wirkungsquantum $\hbar = h/2\pi$ zu (Gleichung (1))

$$\mu_z = \gamma * \hbar * m_I \qquad (1)$$

Teilchen mit Spin lassen sich makroskopisch also als „kleine Magnete“ veranschaulichen. Die Spinzustände stellen sich in diesem Modell als unterschiedliche

Neigungen dieser Magnete relativ zu den Feldlinien eines äußeren Magnetfeldes dar, wobei die „kleinen Magnete“ um diese Feldlinien taumeln (Präzession –Abbildung 3)

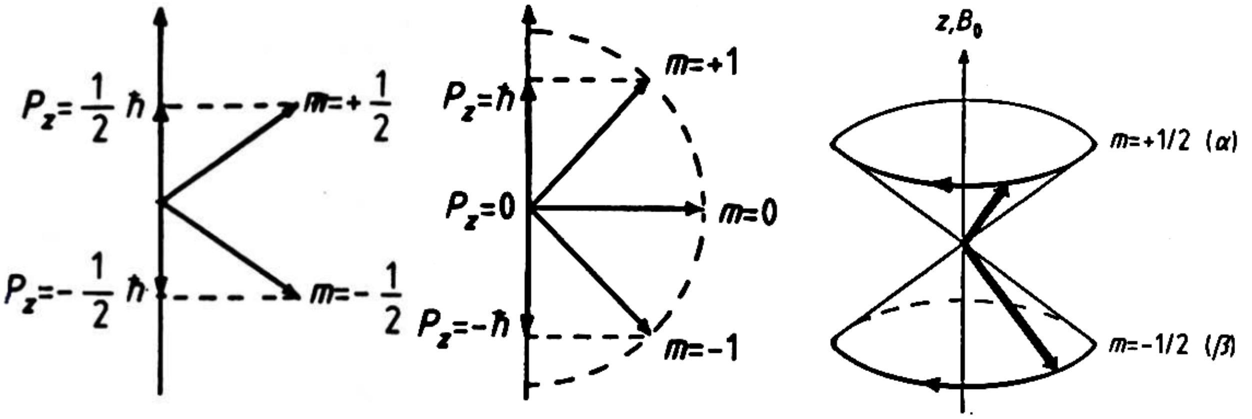

Abbildung 3: Richtungsquantelung des Drehimpulses (und damit Quantelung der z-Komponente des magnetischen Momentes). Links für Kern mit Spinquantenzahl I = ½, Mitte I = 1. Rechts: Präzessionskegel um die z-Achse, die mit der Richtung des äußeren Magnetfeldes gleichgesetzt ist. Für I = ½ ergibt sich ein Öffnungswinkel von 54°44’ (aus [41])

Die Drehfrequenz f dieser Präzession ist dem Magnetfeld am Ort des betrachteten Teilchens direkt proportional (Gleichung (2)):

$$\omega = 2 * \pi * f = \gamma * B_0 \qquad (2)$$

B_0 ist die magnetische Induktion am Ort des Spins. Ein Quant eines elektromagnetischen Wechselfeldes dieser Frequenz besitzt genau die Energie, die der Differenz zweier benachbarter Energiezustände des betrachteten Teilchens ΔE entspricht (Gleichung (3)).

$$\Delta E = h * f = \hbar * \gamma * B_0 \qquad (3)$$

Daher stellt diese, als Larmor-Frequenz bezeichnete Frequenz f die Resonanzfrequenz dar, über die dem spin-tragenden Teilchen Energie zugeführt werden kann, bzw. über die es Energie emittiert. Dies wird im Kernspinresonanz-Experiment benutzt, um mittels Einstrahlung von Energie in Form elektromagnetischer Wellen mit der Larmor-Frequenz Spins in einen energetisch weniger günstigen Zustand zu überführen.

Wegen der günstigen relativen Empfindlichkeit des Wasserstoff-Isotops ^1H, aber auch wegen dessen natürlicher Häufigkeit in biologischem Material (Tabelle 2) wird vorwiegend ^1H zur kernspintomographischen Bildgebung benutzt.

Kern	Spin-quanten-zahl I	Magnet. Moment μ (in Einheiten von μ_0)	Magneto-gyrisches Verhältnis γ (10^8 rad $T^{-1}s^{-1}$)	Resonanz-frequenz ν_0 in MHz bei einem Feld B_0 von 1.0 T	Relative Empfind-lichkeit bei konstantem Feld	Natürliches Vorkom-men (in %)
^{1}H	1/2	2,79277	2,675	42,577	1,000	99,98
^{2}H	1	0,85735	0,411	6,536	0,009	0,0158
^{10}B	3	1,8007	0,288	4,575	0,02	18,83
^{11}B	3/2	2,6880	0,858	13,660	0,165	81,17
^{13}C	1/2	0,70216	0,673	10,705	0,016	1,108
^{14}N	1	0,40369	0,193	3,076	0,001	99,635
^{15}N	1/2	-0,28298	-0,271	4,315	0,001	0,365
^{17}O	5/2	-1,8930	-0,363	5,772	0,029	0,037
^{19}F	1/2	2,6273	2,517	40,055	0,843	100,0
^{29}Si	1/2	-0,55492	-0,531	8,460	0,079	4,70
^{31}P	1/2	1,1316	1,083	17,235	0,066	100,0

Tabelle 2: Kerneigenschaften einiger für die NMR-Spektroskopie organischer Verbindungen wichtiger Kerne (nach [51])

Der Tendenz jedes Spins, (im Rahmen verfügbarer Zustände) den energieärmsten Zustand einzunehmen, wirkt die stochastische Energiezufuhr durch die Wärmebewegung der Atome/Moleküle entgegen. In Abhängigkeit von der Energiedifferenz zwischen zwei Zuständen und von der Temperatur stellt sich damit eine Gleichgewichtsverteilung zwischen Spins in energieärmeren und energiereicheren Zuständen ein. Im Fall des Wasserstoff-Kerns, also eines einzelnen Protons, der wegen seiner Spinquantenzahl I = ½ (Tabelle 2) zwei Spinzustände hat ($m_I = \pm½$), ergibt sich die Gleichgewichtsverteilung nach Gleichung (4):

$$\frac{N_\alpha}{N_\beta} = \exp\left(\frac{-\Delta E}{k * T}\right) = \exp\left(\frac{-\gamma * \hbar * B_0}{k * T}\right) \qquad (4)$$

mit N_α und N_β als den Besetzungszahlen im angeregten und im Grundzustand, ΔE der Energiedifferenz zwischen den Zuständen, k der Boltzmann-Konstanten und T der absoluten Temperatur.

Bei einem Magnetfeld von B_0 = 1.41 T und Raumtemperatur (300 K) ergibt sich ein Besetzungsverhältnis $N_\alpha/N_\beta \approx 0{,}9999904$, bei 7 T verbessert sich dies auf $N_\alpha/N_\beta \approx 0{,}99995$. D.h. unter diesen Bedingungen befinden sich lediglich 9,6 bzw. 50 pro Million Wasserstoffkernen mehr im energiearmen als im energiereichen Zustand und werden damit im Kernspinresonanz-Experiment effektiv wirksam. Im Weiteren werden nur noch diese Spins außerhalb des thermischen Gleichgewichts betrachtet. Aus der gezeigten Besetzungswahrscheinlichkeit für die Spinzustände leitet sich ab, dass der zu erwartende Messeffekt relativ schwach sein wird. Aus Gleichung (2) und Tabelle 2 ergibt sich für ^{1}H bei 0.5 bis 2.0 T eine Larmor-Frequenz zwischen 21.28 MHz und 85.12 MHz, also Frequenzen im ultrakurzwelligen Bereich der Rundfunktechnik (im Folgenden Radiofrequenz RF).

Die Summe der magnetischen Momente der Spins außerhalb des thermischen Gleichgewichts wird im Weiteren als Nettomagnetisierung $\vec{M}$ betrachtet. Das Grundprinzip der Kernspintomographie wie auch der FT-NMR-Spektroskopie[1] kann nun durch die Beeinflussung von M durch das äußere Magnetfeld, durch lokale Magnetfeld-Unterschiede, durch die eben erwähnte Radiofrequenz sowie durch Übergänge von Spins zwischen Grund- und angeregtem Zustand beschrieben werden. Vereinbarungsgemäß sei die z-Achse eines kartesischen Koordinatensystems antiparallel zum äußeren Magnetfeld orientiert. Das System rotiere mit der Larmorfrequenz um die z-Achse.

Mittels eines RF-Impulses mit der Larmor-Frequenz wird ein Teil der Spins aus dem energieärmeren in den energiereichern Zustand überführt. Dies kann durch eine Rotation der ursprünglich antiparallel zum äußeren Magnetfeld ausgerichteten Nettomagnetisierung um den magnetischen Vektor der des RF-Feldes beschrieben werden. Dieser sei parallel zur x-Achse des rotierenden Koordinatensystems orientiert. Damit bewirkt der RF-Impuls eine Auslenkung von $\vec{M}$ in Richtung auf die xy-Ebene. $\vec{M}$ präzediert nun mit der Larmorfrequenz um die z-Achse (vgl. Abbildung 3). Die Energie des RF-Impulses kann so gewählt werden, dass $\vec{M}$ um 90°, also genau in die xy-Ebene gedreht wird („90°-Puls“). Dies entspricht im quantenmechanischen Modell der Überführung genau der Hälfte der Spins vom Grund- in den angeregten Zustand. Diese Spins werden in den energieärmeren Zustand zurückkehren, der Zeitverlauf dieser Rückkehr folgt einem „Zerfallsgesetz“ (Gleichung (5)):

$$M_z(t) = M_{z,0} * \left(1 - \exp\left(\frac{-t}{T_1}\right)\right) \qquad (5)$$

Die Zeitkonstante T_1 bezeichnet hier die Spin-Gitter-Relaxationszeit.

Allgemein bewirkt ein hinreichend schnell ein- und ausgeschalteter RF-Impuls der Länge τ eine Rotation der Netto-Magnetisierung um die x-Achse im rotierenden Koordinatensystem um den Winkel α gemäß Gleichung (6)

$$\alpha = \gamma * B_1 * \tau \qquad (6)$$

[52]). Die Rotation einer Magnetisierung in der xy-Ebene wiederum kann als Quelle der RF betrachtet werden, die den Messeffekt der Kernspintomographie darstellt (vgl. Diskussion der Bloch-Gleichung (8) weiter unten). Die emittierte RF-Energie wird durch die bei der Rückkehr von Spins in den energetisch günstigeren Zustand freiwerdenden Energie gespeist. Ein weitaus größerer Anteil wird jedoch an die Umgebung (bei Kristallen: das Gitter) abgegeben, woraus sich die Bezeichnung „Spin-Gitter-Relaxation“ erklärt.

Wenn das lokale Magnetfeld für alle betrachteten Spins exakt gleich ist, genügt ein einziger Nettomagnetisierungs-Vektor zu deren Beschreibung. Tatsächlich tauschen die

---

[1] FT steht hier für Fourier-Transformation im Unterschied zur CW-NMR-Spektroskopie (continuous wave).

Kerne jedoch Energie untereinander aus (Übergänge zwischen den Zuständen „spin up“ und „spin down“), was zu Magnetfeldfluktuationen führt. Eine weitere Veranschaulichung hierfür sind Magnetfeldfluktuationen durch thermische Bewegungen: hierdurch ändern sich Abstände zu benachbarten Spins und damit auch deren Beiträge zum Magnetfeld des betrachteten Spins. Die lokalen Magnetfeldunterschiede führen zu leicht unterschiedlichen Larmor-Frequenzen. Dies kann durch die Einführung mehrerer, unterschiedlich schnell präzedierender Magnetisierungs-Vektoren für Gruppen von Spins mit jeweils gleicher lokaler Magnetfeldstärke beschrieben werden. Diese Vektoren weisen nach dem Umklappen in die xy-Ebene zunächst eine gemeinsame Phasenlage auf. Die unterschiedlichen Präzessionsfrequenzen führen jedoch zum Verlust dieser Phasenkohärenz, also zur Dephasierung. Mit dem Zerfall der Phasenkohärenz reduziert sich auch die Komponente der Nettomagnetisierung in der xy-Ebene gemäß Gleichung (7):

$$M_{xy}(t) = M_{xy,0} * \exp\left(\frac{-t}{T_2}\right) \tag{7}$$

mit M_{xy} als Nettomagnetisierung in der xy-Ebene (Index 0 für den Zustand unmittelbar nach dem 90°-RF-Impuls). Bei dem hier beschriebenen Prozess wird keine Energie an die Umgebung abgegeben, er wird als Spin-Spin-Relaxation bezeichnet. Die Zeitkonstante des Zerfalls der Quermagnetisierung M_{xy} T_2 ist ebenso wie T_1 und wie die Dichte der „Überschuss-Spins“ ρ (vgl. oben) eine gewebscharakterisierende Größe.

Die Veränderung des Vektors der Netto-Magnetisierung $\vec{M}$ wird empirisch durch die Bloch-Gleichung (Gleichung (8)) [52] beschrieben:

$$\frac{d\vec{M}}{dt} = \gamma * \vec{M} \times \vec{B}_0 + \frac{1}{T_1} * (M_0 - M_z) * \hat{z} - \frac{1}{T_2} * \vec{M}_{xy} \tag{8}$$

Der erste der drei Summanden stellt die klassische Bewegungsgleichung eines magnetischen Moments $\vec{M}$ im äußeren Feld dar. Die folgenden Summanden erfassen phänomenologisch die in den Gleichungen (5) und (7) dargestellten Relaxationsprozesse. Die kompakte Darstellung wird durch den Bezug auf ein mit der Larmor-Frequenz um die z-Achse rotierendes Koordinatensystem erreicht.

Der dritte Summand in der Bloch-Gleichung beschreibt eine sich sehr schnell (nämlich mit der Larmor-Frequenz) ändernde Magnetisierung. Diese ist genau dann in der Lage, in einer Stromschleife eine Spannung zu induzieren, wenn der diese durchdringende magnetische Fluss sich ändert (Faradaysches Induktionsgesetz – Gleichung (9)). Dies ist der Fall, wenn die Stromschleife nicht exakt in der xy-Ebene liegt.

$$\oint \vec{E} * d\vec{l} = -\frac{d}{dt}\left(\int \vec{B} * d\vec{S}\right) \tag{9}$$

mit $\oint d\vec{l}$ als dem Umfang der Fläche $\int d\vec{S}$. Die Spannung $\oint \vec{E} * d\vec{l}$ wird maximal, wenn die Fläche der Stromschleife senkrecht zur xy-Ebene angeordnet ist. Dieser induzierte Strom stellt den Messeffekt in der kernspintomographischen Bildgebung dar

(auf den in der Kernspin-Spektroskopie ebenfalls denkbaren Ansatz, die Absorption der RF durch das untersuchte Material in Abhängigkeit von der Frequenz zu messen, wird hier nicht weiter eingegangen).

Unter realen Bedingungen wird die Zeitkonstante T_2 in Gleichung (7) durch lokale Feldinhomogenitäten, aber u. U. auch durch zusätzlich geschaltete ortsabhängige Magnetfelder („Gradientenfelder“ – siehe unten) verkürzt. Der dadurch bedingte sehr schnelle Abfall der Quermagnetisierung M_{xy} wird als „Free Induction Decay“ (FID) bezeichnet. Er wird üblicherweise durch die Zeitkonstante T_2^* gekennzeichnet (Gleichung (10))

$$\frac{1}{T_2^*} = \frac{1}{T_2} + \frac{1}{T_2'} \qquad (10)$$

Je nach Sequenzdesign lassen sich die durch Gradientenschaltung bedingten oder aber alle (relativ zur Echozeit T_E (Abschnitt 2.2.2) statischen) Anteile von T_2' refokussieren.

Anmerkung: Lokaler Suszeptibilitäts-Inhomogenitäten führen im allgemeinen Fall nicht zu einer exponentiell mit der Zeit abfallenden Quermagnetisierung, wie das Gleichung (10) impliziert. Allerdings kann zumindest unter gewissen Annahmen zur Häufigkeits-verteilung der Feldstärke in einem Voxel die Anfangsphase des inhomogenitäts-bedingten Magnetisierungs-Zerfalls hinreichend genau mit einer Exponentialfunktion beschrieben werden (Abbildung 4). Die zunehmende Abweichung von einer Exponentialfunktion zu späteren Zeitpunkten ist weniger relevant, da M_{xy} dann durch den Anteil von T_2 an T_2^* erheblich reduziert sein dürfte.

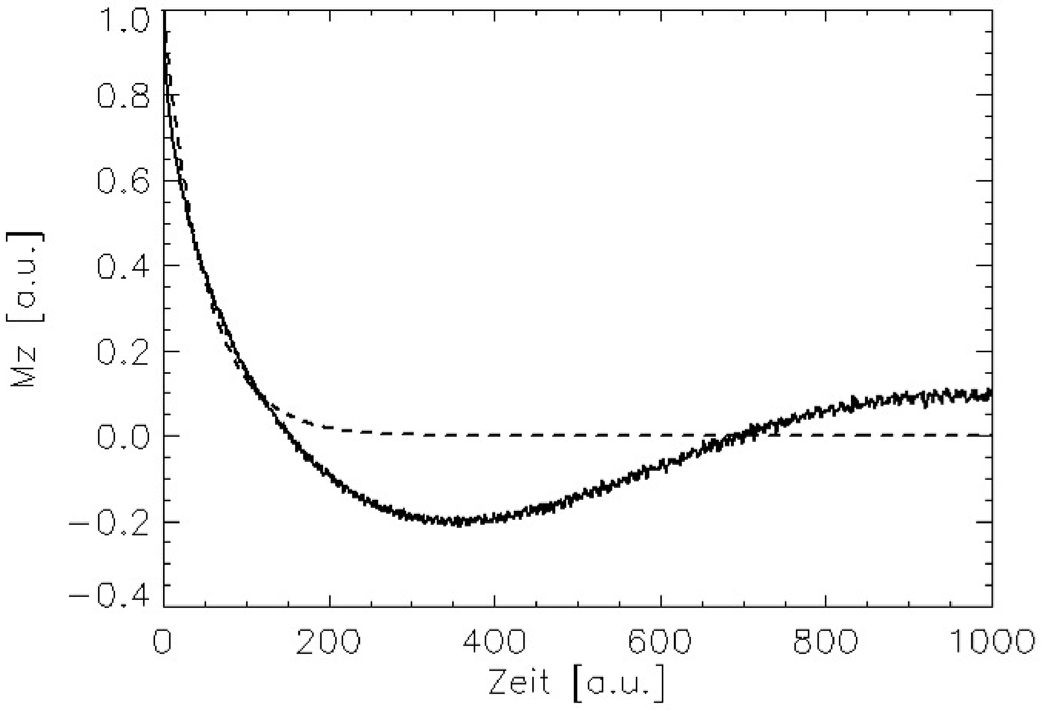

Abbildung 4: Zeitabhängigkeit der Quermagnetisierung unter Annahme einer quadratischen Abnahme einer linearen Magnetfeld-Störung in einer Ebene (——). Exponentialfunktion zum Vergleich (**- - - -**)

2.2.2 Einige grundlegende Sequenztechniken (Auswahl)

In der Kernspintomographie kommt eine Vielzahl von Sequenzen zum Einsatz, deren Bezeichnung zum Teil spezifisch für den jeweiligen Gerätehersteller ist [131, 130]. Zum prinzipiellen Verständnis der gerätetechnischen Anforderungen sollen hier einige grundlegende Sequenztypen näher erläutert werden (Abbildung 5 links).

Die Messstrategie bei der kernspintomographischen Bildgebung lässt sich im wesentlichen als eine zyklische Abfolge der folgenden Schritte beschreiben:

1. (teilweise) Inversion der Besetzung der Spinzustände mittels RF-Impuls
   ⇒ Reduktion der Komponente der Netto-Magnetisierung in z-Richtung (bis hin zur
   Vorzeichenumkehr („Inversion Recovery Sequence")
   ⇒ Erzeugung einer Netto-Magnetisierung in der xy-Ebene

2. (teilweiser) Verlust der Phasenkohärenz durch T_2-Relaxation sowie makroskopische Einflüsse
   ⇒ Abnahme der Netto-Magnetisierung in der xy-Ebene
   gleichzeitig Spin-Gitter-Relaxation
   ⇒ Wiederaufbau der Netto-Magnetisierung in z-Richtung

3. (teilweise) Phasen-Refokussierung
   ⇒ Wiederaufbau der Netto-Magnetisierung in der xy-Ebene (bis zu einem u.a. durch die
   Spin-Spin-Relaxationszeit sowie lokale Magnetfeldinhomogenitäten und ~fluktuationen gegebenen Maß)
   ⇒ Messung des von der in der xy-Ebene rotierenden Komponente der Netto-Magnetisierung verursachten RF-Signals

4. (teilweise) Relaxation des Spinsystems

5. evtl. Zerstörung restlicher Nettomagnetisierung in der xy-Ebene („Spoiling")

6. =1.

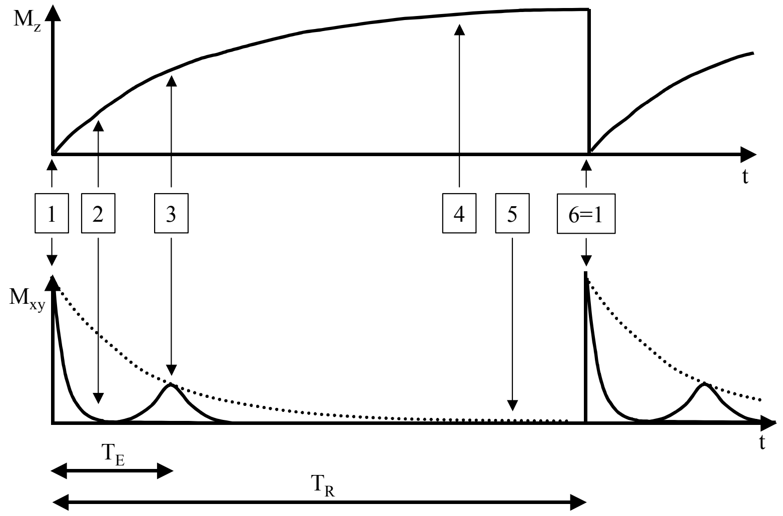

Abbildung 5: Prinzipieller Aufbau einer konventionellen Messsequenz. Legende der Nummerierung im Text.

Die Zeit zwischen Schritt 1 und Schritt 3 wird als Echozeit T_E, die Zeit zwischen Schritt 1 und Schritt 6 als Repetitionszeit T_R bezeichnet. Mit zunehmender Echozeit gewinnt also die $T_2^{(*)}$-Relaxation an Einfluss auf die Signalintensität, entsprechende Sequenzen werden als $T_2^{(*)}$-gewichtet bezeichnet (Abbildung 6 links.). Mit zunehmender T_E steht allerdings auch weniger Netto-Magnetisierung in der xy-Ebene und damit weniger Signal zur Verfügung. Man kann anhand der Signalintensitäts-Formeln (vgl. Gleichungen (14) für Spinecho und (15) für Gradientenecho) zeigen, dass ein optimales Kontrast-Rauschverhältnis bezüglich $T_2^{(*)}$ bei einer Echozeit von $T_E = T_2^{(*)}$ erreicht wird.

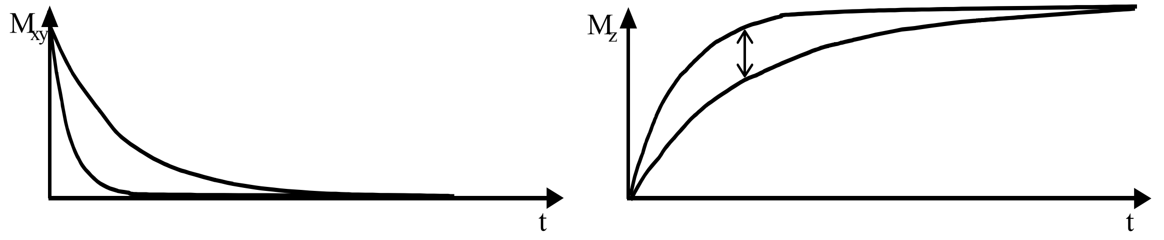

Abbildung 6: Links: Spin-Spin-Relaxation für zwei unterschiedliche T_2-Zeiten. Rechts: Spin-Gitter-Relaxation für zwei unterschiedliche T_1-Zeiten. Optimales Kontrast-Rausch-Verhältnis wird erreicht, wenn T_R an der Position des Doppelpfeils endet.

Die Spin-Gitter-Relaxation für zwei Substanzen mit unterschiedlicher T_1-Zeit nach einem 90°-Impuls ist in Abbildung 6 rechts dargestellt. Der Einfluss von T_1 auf die Netto-Magnetisierung in z-Richtung (die durch den nächsten RF-Impuls in die xy-Ebene geklappt werden kann), nimmt mit der Zeit ab. Analog zur $T_2^{(*)}$-Wichtung ergibt sich optimales Kontrast-Rausch-Verhältnis bezüglich T_1 für eine Repetitionszeit $T_R = T_1$ (unter der Annahme vollständigen Spoilings). Da die T_1-Zeiten der in der medizinischen Bildgebung relevanten Substanzen in der Größenordnung 100er Millisekunden liegen, folgt aus dem eben Gesagten die Notwendigkeit relativ langer Repetitionszeiten. Diese lassen sich u. U. vermeiden, wenn die Netto-Magnetisierung um kleinere Flipwinkel $\alpha < 90°$ aus der z-Richtung gekippt wird. Allerdings wird dann auch nur die Komponente in der xy-Ebene $M_{xy} = M_{z,0} * \sin(\alpha)$ wirksam (Anwendung z. B. in Gradientenecho-Sequenzen – Abschnitt 2.2.2.2).

Wenn der Einfluss sowohl von T_1 als auch von $T_2^{(*)}$ auf das Signal minimiert wird, verbleibt als bildkontrast-bestimmende Größe die Protonendichte ρ (Tabelle 3).

Bildkontrast	T_R	T_E
T_1-Wichtung	kurz	kurz
$T_2^{(*)}$-Wichtung	lang	lang
ρ-Wichtung	lang	kurz

Tabelle 3: Qualitativer Zusammenhang von Sequenzparametern und Bildwichtung

Mit der gezeigten Messstrategie lässt sich ein von der Protonendichte ρ, den Relaxationszeiten T_1 und T_2 (sowie weiteren Einflüssen wie z. B.

Suszeptibilitätsinhomogenitäten) abhängiges Signal gewinnen. Dieses ist jedoch zunächst ortsunabhängig (Empfindlichkeitsprofile von Sende-/Empfangsstufen sowie die endliche Ausdehnung des äußeren Magnetfeldes seien hier vernachlässigt).

Zum Aufprägen der Ortsinformation auf das Signal wird die Abhängigkeit der Larmor-Frequenz vom Magnetfeld am Ort des jeweiligen Spins benutzt (Gleichung (2)). Zu diesem Zweck wird das homogene äußere Magnetfeld („Hauptmagnetfeld“) zeitweilig mit linear von einer Raumrichtung abhängigen Magnetfeldern derselben Richtung wie das Hauptmagnetfeld überlagert (Magnetfeld-Gradienten). Hierfür gibt es drei grundlegende Ansätze:

Schichtselektion: Zuschaltung eines Gradienten während des RF-Impulses. Damit herrscht die Resonanzbedingung nur für eine Schicht senkrecht zur Gradientenrichtung, deren Dicke von der Bandbreite des RF-Impulses abhängt.

Frequenzkodierung: Zuschaltung eines Gradienten, während das RF-Signal empfangen wird. Damit hängt die Frequenz des Signals von der Position der „sendenden“ Spins in Richtung dieses Gradienten ab. Um die Frequenzen gemäß der gewünschten räumlichen Auflösung mittels Fourier-Transformation voneinander trennen zu können, muss das Empfangssignal über eine hinreichende Anzahl von Zeitpunkten akquiriert werden (siehe Nyquist-Theorem weiter unten).

Phasencodierung: Zuschaltung eines Gradienten in einem Zeitfenster zwischen RF-Anregung und Empfang des Signals. Damit wird die Phasenlage der Spins abhängig von ihrer Position in Richtung dieses Gradienten. Um eine gemäß der gewünschten räumlichen Auflösung erforderliche Anzahl von Phasenlagen (und damit Positionen in Richtung des Phasencodier-Gradienten) mittels Fourier-Transformation voneinander trennen zu können, müssen Signale nach einer hinreichenden Anzahl von Messungen mit unterschiedlich starken Phasencodier-Gradienten akquiriert werden (siehe Nyquist-Theorem weiter unten).

Je nach Design der Messsequenz werden alle drei Ansätze benutzt, oder es wird auf die Schichtselektion verzichtet und die entsprechende Raumrichtung ebenfalls phasencodiert („Volumenanregung“). Für die Codierung werden zueinander orthogonale Raumrichtungen gewählt.

Die gemessenen Signalintensitäten werden in Abhängigkeit von der Zeit (Frequenzcodierung) und Phasenkodier-Bedingungen in eine zwei- oder dreidimensionale Matrix eingetragen (letzteres bei Sequenzen mit Volumenanregung). Sie stellt die Fourier-Transformierte der Bildinformation, also den Raum der das Bild formierenden Ortsfrequenzen dar und wird daher als k-Raum bezeichnet (Abbildung 7 links). Die Position im k-Raum wird jeweils durch das Integral der Gradientenstärke über die Zeit bestimmt (Gleichung (11)) – dies erklärt die prinzipielle Gleichwertigkeit von Frequenz- und Phasenkodierung bezüglich der Fourier-Transformation[2].

---

[2] Der Zeitverlauf des Aufbaus des Gradienten-Zeit-Integrals ist jedoch durchaus relevant für die Ausbildung von Artefakten. Beispielsweise ergibt sich aus der relativ großen Zeitspanne zwischen den Phasenkodierschritten (bei Standard-Sequenzen jeweils T_R) eine größere Anfälligkeit für Bewegungsartefakte.

$$k(t) = \frac{\gamma}{2 * \pi} * \int_0^t G(t')\,dt' \quad (11)$$

Aus der Interpretation als Ortsfrequenzraum lässt sich ableiten, dass die zentralen Bereiche des k-Raumes den Bildkontrast bestimmen – sie repräsentieren die niedrigen Ortsfrequenzen (Abbildung 7 Mitte). Die peripheren Bereiche dagegen (also die hohen Ortsfrequenzen) sind für die Detailerkennbarkeit relevant (Abbildung 7 rechts).

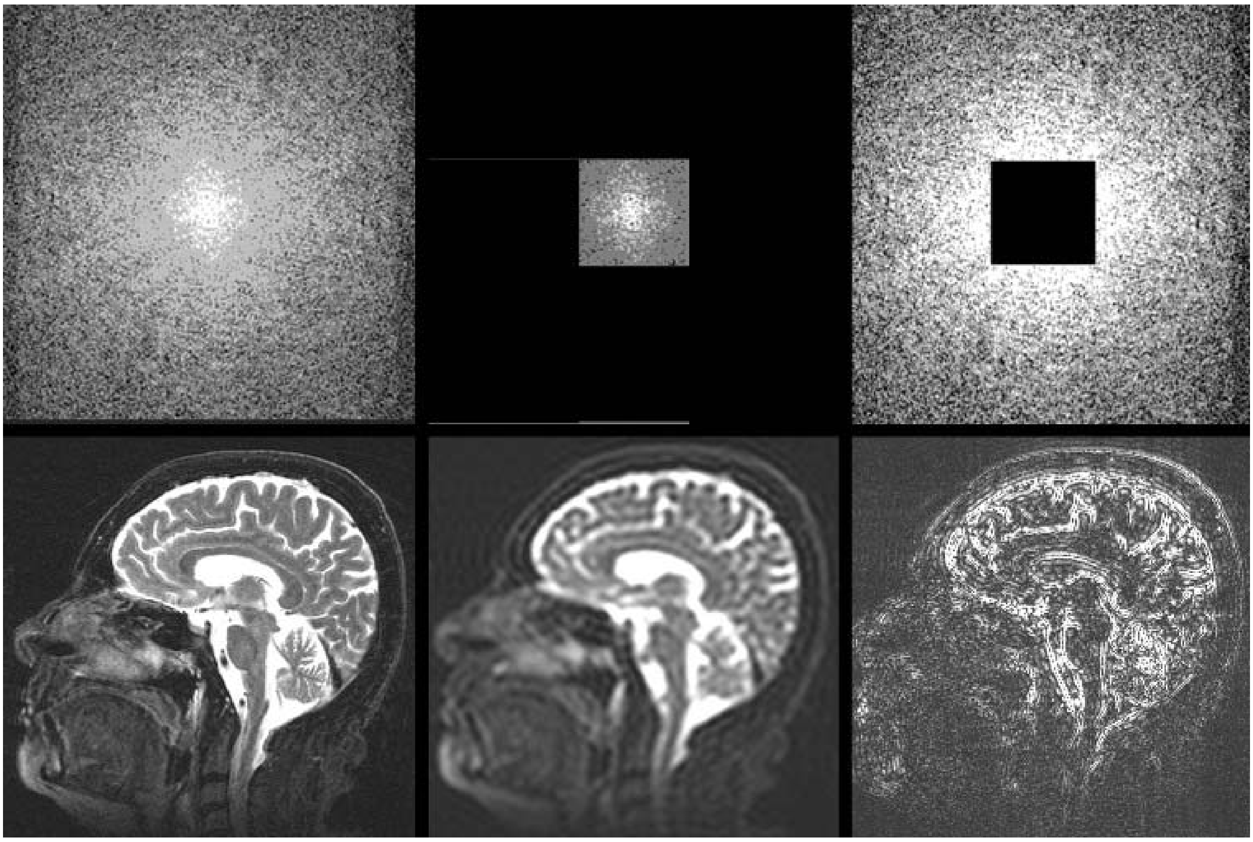

Abbildung 7: Beziehung zwischen k-Raum-Füllung und Bildinformation. Oben: k-Räume. Unten: zugehörige rekonstruierte Bilder. Links: vollständige k-Raum-Füllung. Mitte: der innere Anteil des k-Raums repräsentiert die niedrigen Ortsfrequenzen bestimmt die groben Strukturen („den Kontrast“). Rechts: die im äußeren Bereich des k-Raumes lokalisierten hohen Ortsfrequenzen definieren die feinen Strukturen (Kanten)

Das Signal, also die in der Empfangsspule induzierten Spannung, wird zu definierten Zeitpunkten Δt gemessen. Bei äquidistanter Signalaufnahme lassen sich diese als $t_q = \Delta t * q$ (q = 0, 1, 2, ...) und die entsprechende Position im k-Raum mit Gleichung (11) als $k = k_0 + q * \Delta k$ mit $\Delta k = \frac{\gamma}{2 * \pi} * \int_t^{t+\Delta t} G(t')\,dt'$ formulieren[3]. Das diskontinuierlich gemessene Signal während des Frequenzkodier-Gradienten in Abhängigkeit von der

[3] Bei Kenntnis von G(t) beschränkt sich die Forderung nach Äquidistanz auf die Schrittgröße im k-Raum (vgl. Beschreibung der EPI-Sequenzen in Abschnitt 2.2.2.5)

Position im k-Raum s(k) lässt sich mit Hilfe von Dirac-Delta-Funktionen darstellen (Gleichung (12)):

$$s(k) = \Delta k * \sum_q s(k_0 + q * \Delta k) * \delta\big(k - (k_0 + q * \Delta k)\big) \qquad (12)$$

Der Faktor Δk resultiert hierbei aus der Notwendigkeit, $s(k_0 + q * \Delta k)$ als „Signaldichte“ zu interpretieren. Die Fourier-Transformation zur Berechnung des Signals im Ortsraum S(x) aus dem gemessenen Signal während des Frequenzkodier-Gradienten in Abhängigkeit von der Position im k-Raum s(k) wird diskret (Gleichung (13)):

$$S(x) = \int s(k) * \exp\big(i * 2\pi * k * x\big)\, dk = \Delta k * \sum_q s(k_0 + q * \Delta k) * \exp\big(i * 2\pi * q * \Delta k * x\big) \qquad (13)$$

Der Term exp(i * 2π * q * Δk * x) ist periodisch für ganzzahlige Schritte in q * Δk * x. Dies gilt insbesondere für den Übergang von x zu x + 1/Δk. Das Ergebnis der diskreten Fourier-Transformation ist damit ein mit 1/Δk periodisches Bild. Daraus resultiert die artefaktfreie Darstellbarkeit von Objekten mit einer Maximalgröße von 1/Δk (Abbildung 8), also ein durch die Schrittweite im k-Raum gegebenes maximales Field of View.

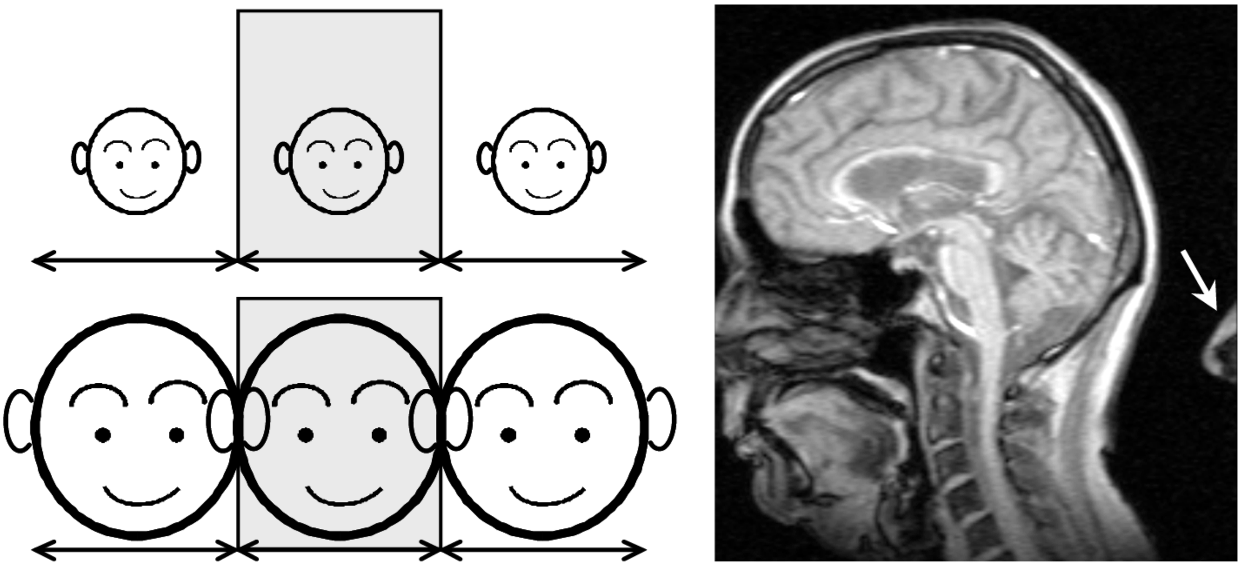

Abbildung 8: Rückfaltungsartefakt durch Verletzung der Nyquist-Bedingung. Links: Periodische Abbildung eines Objektes mit der Periode 1/Δk. Oben: Korrekte Darstellung eines Objektes kleiner als 1/Δk. Unten: Überlappung bei einem Objekt größer als 1/Δk. Grau unterlegt: rekonstruiertes Bild. Rechts: reale Messung, Rückfaltung der Nasenspitze.

Wegen der oben dargestellten Äquivalenz bezüglich Frequenz- und Phasenkodier-Richtung gelten diese Überlegungen ebenso in Phasenkodierrichtung[4].

[4] In praxi ist der Rückfaltungsartefakt nur in Phasenkodier-Richtung von Bedeutung, da in Frequenzkodier-Richtung praktisch ohne zusätzlichen Messzeit-Aufwand Δk kleiner als für das gewünschte FOV gewählt werden kann („Oversampling“)

2.2.2.1 Spinecho-Sequenz

Mittels 90°-Impuls wird die gesamte Nettomagnetisierung in z-Richtung M_z in die xy-Ebene geklappt und beginnt sofort zu dephasieren. Nach einer Zeit $T_E/2$ wird ein RF-Impuls mit doppeltem Amplituden-Zeit-Produkt gesendet (Gleichung (6)). Da dieser die Netto-Magnetisierung um 180° um die x-Achse dreht, kehren sich die Vorzeichen der in Gleichung (10) mit T_2' erfassten Differenzen der Phasenlagen zum lokalen Mittelwert um, so dass nach einem weiteren Zeitintervall $T_E/2$ diejenigen Phasendifferenzen, die durch während T_E stabile lokale Magnetfeldunterschiede bedingt sind, kompensiert werden (Abbildung 9).

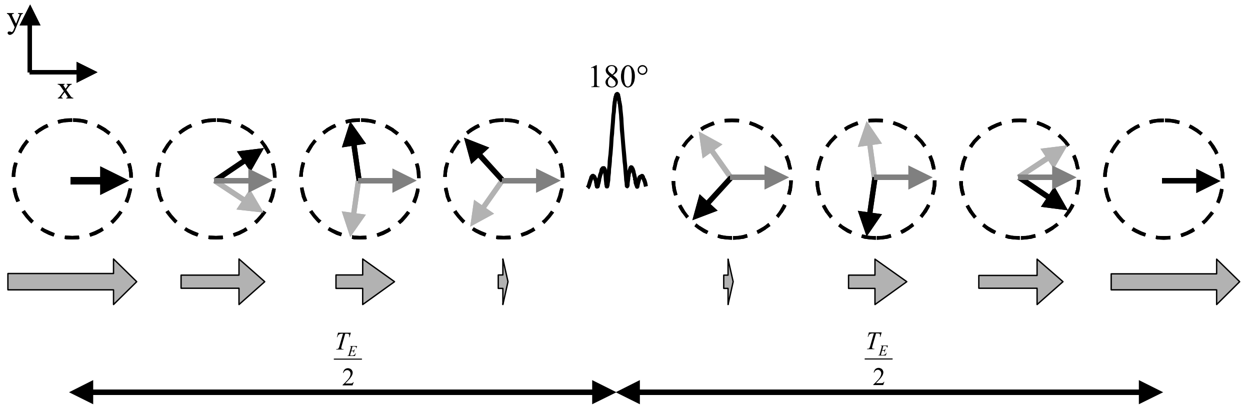

Abbildung 9: Um die z-Achse (senkrecht zur Bildebene) präzedierende Spins im rotierenden Koordinatensystem. Präzessionsfrequenz ω (dunkelgrau), $\omega + \Delta\omega$ (schwarz), $\omega - \Delta\omega$ (hellgrau). ⇨ Nettomagnetisierung in der xy-Ebene.

M_{xy} erreicht damit ein lokales Maximum („Spin-Echo"). Zu diesem Zeitpunkt wird das Signal aufgenommen. Zum Zeitpunkt T_R nach dem 90°-Anregungsimpuls wird der Zyklus wiederholt. Die Signalintensität kann mit Gleichung (14) beschrieben werden:

$$S = \rho * \exp(-T_E/T_2) * (1 - \exp(-T_R/T_1)) \qquad (14)$$

Zur Ortskodierung werden Schicht-, Phasen- und Frequenzkodiergradienten benutzt, wobei der Schichtselektionsgradient während beider RF-Impulse geschaltet werden muss (Abbildung 10 oben). Im Allgemeinen gilt $T_R >> T_E$, d.h. der Hauptteil der Zykluszeit wird lediglich für eine hinreichende Spin-Gitter-Relaxation benötigt. Da sowohl Anregung als auch Refokussierung mittels RF schichtselektiv sind, kann die Messung mehrerer Schichten ineinander verschachtelt durchgeführt werden (Abbildung 10 unten)

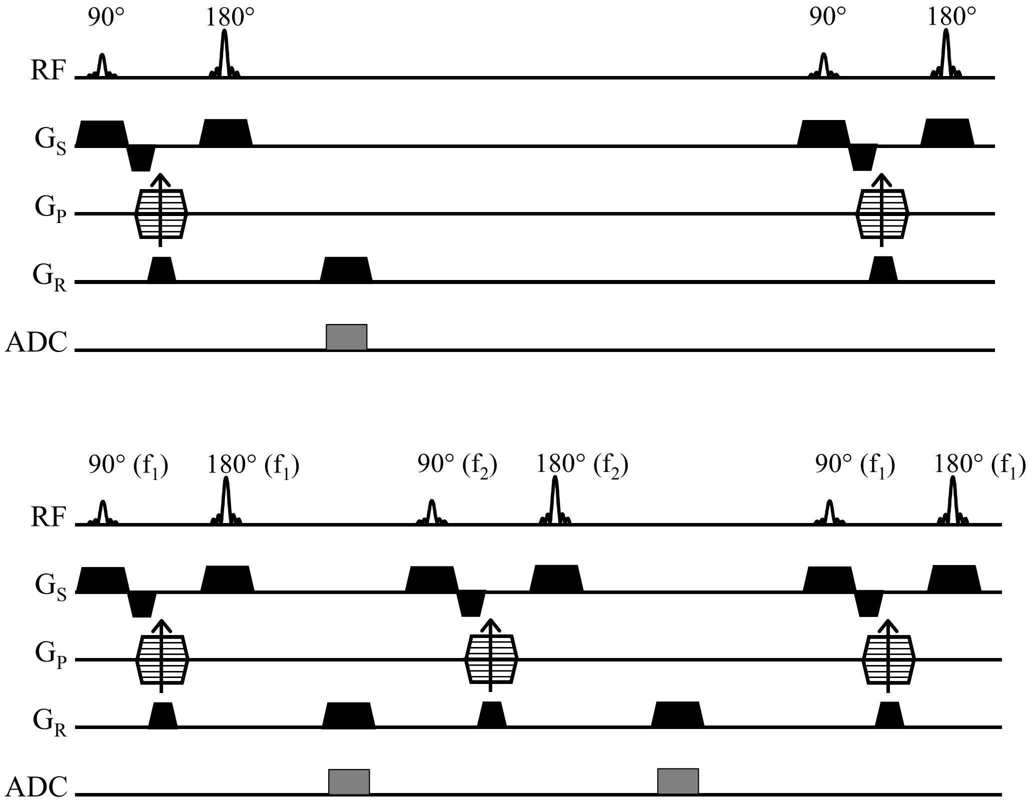

Abbildung 10: Sequenzdiagramm einer Spinecho-Sequenz. Oben: Selektive Anregung nur einer Schicht. Unten: In der Wartezeit zwischen Auslesen der mit einer Frequenz f_1 angeregten Schicht und erneuter Anregung derselben wird eine zweite Schicht (Anregungsfrequenz f_2) angeregt und ausgelesen.

2.2.2.2 Gradientenecho-Sequenz

Nach dem Anregungsimpuls wird mittels eines Gradienten in Richtung der Frequenzkodierung eine schnelle Dephasierung (kurzes $T_2^{'}$) erzwungen. Später wird ein Gradient in selber Raumrichtung mit umgekehrtem Vorzeichen geschaltet. Der durch den zuerst genannten Gradienten bewirkte Anteil der Dephasierung ist zu dem Zeitpunkt kompensiert, an dem sich die Integrale der Gradientenfeldstärken über die Zeit aufheben („Gradienten-Echo“). Zu diesem Zeitpunkt wird das Signal aufgenommen (Abbildung 11 oben). Im Unterschied zur Spinecho-Sequenz wird der durch lokale, zeitunabhängige Magnetfeldunterschiede bedingte Anteil der Dephasierung bei Gradientenechosequenzen also nicht rephasiert und bleibt daher signalmindernd wirksam. Dadurch bilden sich Suszeptibilitäts-Inhomogenitäten z. B. an Knochen-Weichteil-Grenzen oder zwischen Gewebe und KM-haltigen Blutgefäßen in mittels Gradientenecho-Sequenzen gewonnenen Bildern im Gegensatz zu Spinecho-Sequenzen deutlich signalgemindert ab. Da bei dieser Form der Rephasierung nur ein RF-Impuls benötigt wird, lassen sich relativ kurze Repetitionszeiten erreichen. Um für den Anregungsimpuls des nächsten Zyklus ausreichend Netto-Magnetisierung in z-Richtung zur Verfügung zu haben, ist es bei kurzen T_R sinnvoll, Anregungswinkel $\alpha < 90°$ zu wählen. Aus Gleichung (15) für die Signalintensität einer gespoilten Gradientenecho-Sequenz lässt sich ableiten, dass sich maximales Signal ergibt, wenn der Anregungswinkel α (Flipwinkel) gleich dem Ernst-Winkel α_E gewählt wird (Gleichung (16))

$$S = \rho * \sin(\alpha) * \exp(-T_E / T_2^*) * \frac{1-\exp(-T_R / T_1)}{1-\cos(\alpha) * \exp(-T_R / T_1)} \tag{15}$$

$$\cos(\alpha_E) = \exp\left(\frac{-T_R}{T_1}\right) \tag{16}$$

Da das Refokussierungs-Prinzip der Gradientenecho-Sequenzen nicht schichtselektiv wirkt, ist ein zeitlich verschachteltes Messen mehrerer Schichten hier nicht möglich.

Zur Ortskodierung können Schicht-, Phasen- und Frequenzkodiergradienten benutzt werden. Alternativ kann auf den Schichtselektionsgradienten verzichtet („Volumenanregung) und die Ortsinformation in dieser Richtung durch einen zusätzlichen Phasenkodiergradienten aufgeprägt werden[5] (Abbildung 11 unten). Je nach Dimensionalität des daraus resultierenden k-Raumes spricht man von 2D-FLASH bzw. 3D-FLASH (Fast Low Angle Shot).

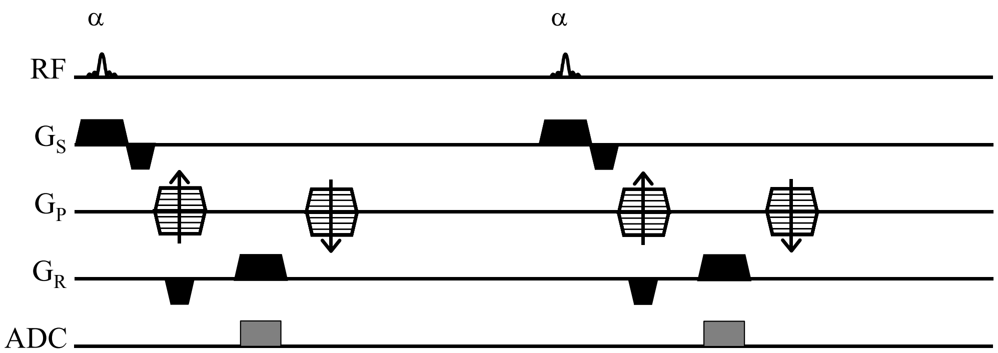

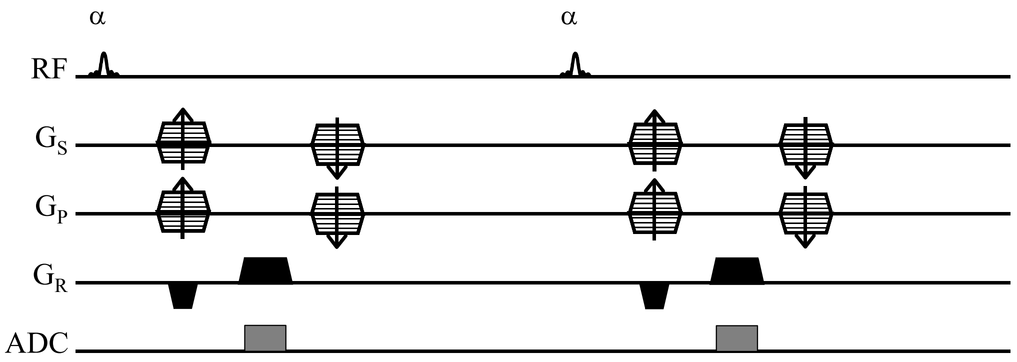

Abbildung 11: Sequenzdiagramm einer refokussierten Gradientenecho-Sequenz (FLASH). Da der Anregungswinkel α in der Regel < 90° gewählt wird, ist für hinreichende Spin-Gitter-Relaxation eine geringere Wartezeit bis zur nächsten

---

[5] Aus Gründen des Signal-Rausch-Verhältnisses wird man auch hier einen Schichtselektions-Gradienten anwenden und den RF-Anregungsimpuls so breitbandig wählen, dass die Resonanzbedingung für das ganze *darzustellende* Volumen erfüllt ist.

Anregung (T_R) erforderlich (vgl. Abbildung 10). Oben: Schichtselektive Anregung, Phasenkodierung in einer Raumrichtung (2D-FLASH). Unten: Volumenanregung (3D-FLASH), d.h. es wird auf den Schichtselektionsgradienten während des RF-Impulses verzichtet. Dafür wird auch in dieser Richtung eine Phasenkodierung vorgenommen.

Bei Anwendung von Anregungswinkeln $\alpha < 90°$ bildet sich erst nach einigen Anregungen ein stationärer Zustand aus – die Nettomagnetisierung M_z erreicht innerhalb einer Repetitionszeit T_R einen niedrigeren Wert, als das bei vollständiger Spin-Gitter-Relaxation ($T_R \to \infty$) der Fall wäre. Substanz, die erst nach einigen Anregungszyklen in das gemessene Volumen eintritt, besitzt daher bei gleicher Protonendichte zunächst eine höhere Nettomagnetisierung M_z, die mit dem nächsten RF-Impuls in eine stärkere Magnetisierung in der xy-Ebene und damit letztlich in eine höhere Signalintensität umgesetzt wird (Abbildung 12). Dies ist der Grundgedanke der Darstellung von strömendem Blut mittels Time-of-Flight-Angiographie (die Zeit, während der das Blut durch das mit RF-Impulsen angeregte Volumen „fliegt", reduziert das messbare Signal und damit den Kontrast zum umgebenden Gewebe).

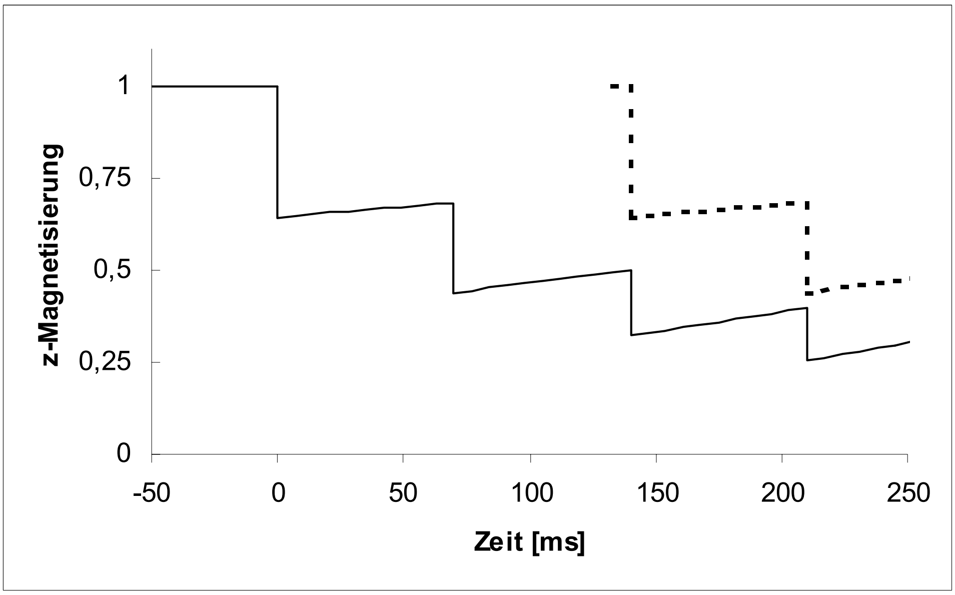

Abbildung 12: Ausbildung einer stationären z-Magnetisierung nach mehreren Anregungen ($\alpha = 50°$, $T_R = 70$ ms, $T_1 = 600$ ms) (—). Erst später (hier nach dem zweiten RF-Impuls) in das angeregte Volumen eintretendes Blut weist beim nächsten RF-Impuls eine höhere z-Magnetisierung als das Gewebe auf und stellt sich dadurch signalstärker dar.

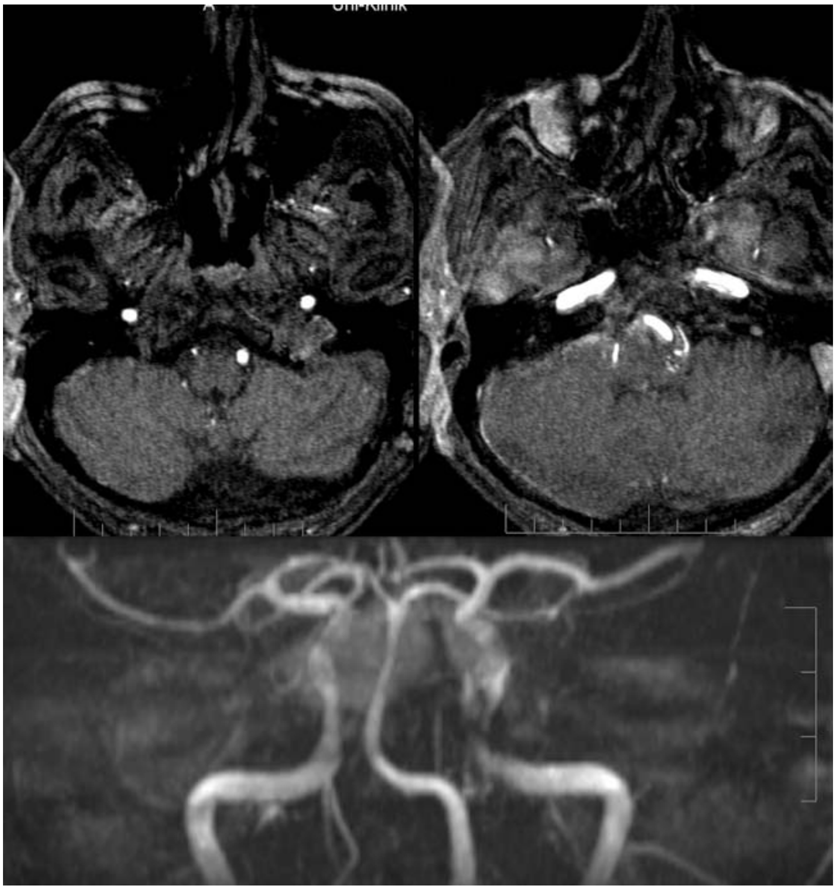

Abbildung 13: Time-Of-Flight-Angiographie der großen basalen Hirngefäße. Oben: primäre Schnittbilder, unten Maximum Intensity Projection.

2.2.2.3 Inversion Recovery

Bei diesem Sequenztyp wird die gesamte Nettomagnetisierung in z-Richtung mittels eines 180°-RF-Impulses invertiert. Da der T_1-Relaxationsprozess hier nicht nur die Hälfte (wie bei der Spinecho-Sequenz), sondern alle („Überschuss"-)Spins betrifft, lässt sich mit dieser Sequenz eine ausgeprägte T_1-Wichtung erreichen (Gleichung (17)).

$$S = \rho * \exp\left(-T_E / T_2\right) * \left(1 - 2 * \exp\left(-T_I / T_1\right) + \exp\left(-T_R / T_1\right)\right) \qquad (17)$$

Im Gegensatz zur Spinecho-Sequenz steht nach dem Anregungsimpuls zunächst keine Magnetisierung in der xy-Ebene zur Verfügung. Diese wird nach einer gewissen Wartezeit (inversion time T_I) durch einen 90°-Impuls erzeugt. Die Situation nach diesem 90°-Impuls entspricht der nach der 90°-Anregung bei der Spinecho-Sequenz. Die Signal-Messung kann damit auch in Analogie zur Spinecho-Sequenz (also mittels Refokussierung durch einen 180°-Impuls) erfolgen (Abbildung 14).

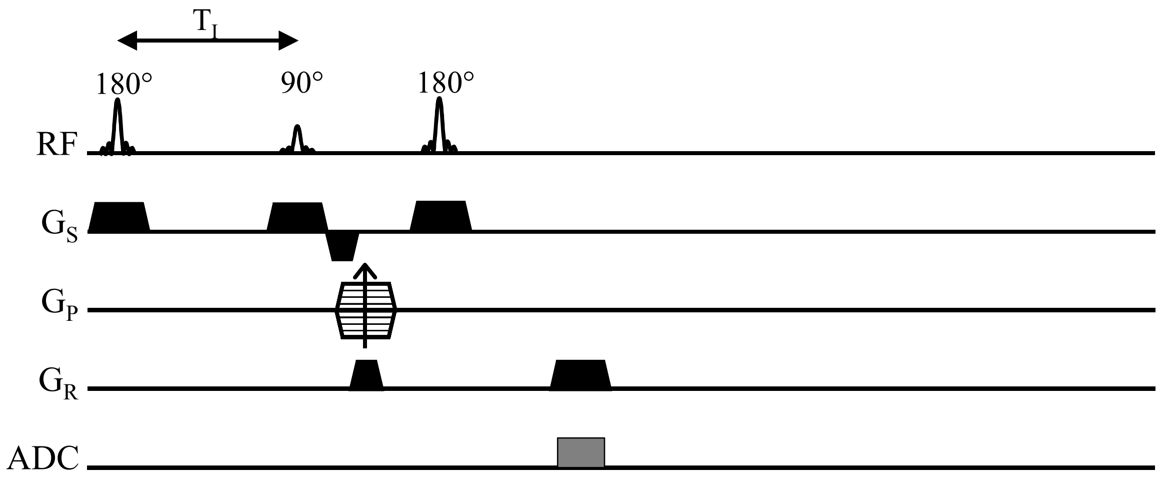

Abbildung 14: Sequenzdiagramm einer Inversion Recovery Sequenz. Der Inversionsimpuls ist im gezeigten Beispiel einer Spinecho-Sequenz zur Signalauslesung vorgeschaltet

Im Gegensatz zu den bisher beschriebenen Sequenztypen wechselt M_z im Zuge der Spin-Gitter-Relaxation das Vorzeichen. Dies erlaubt, durch geeignete Wahl der Wartezeit T_I dafür zu sorgen, dass Gewebe mit einer bestimmten T_1-Relaxationszeit keinen Beitrag zum Signal liefert. Dieses Prinzip findet Anwendung bei der Unterdrückung von Fettgewebe – es trägt wegen der relativ kurzen Wartezeit T_I hier den Eigennamen STIR (Short Tau Inversion Recovery). Bei entsprechend längerer T_I kann auch der Beitrag von Wasser (im klinischen Betrieb in der Regel von Hirnwasser (Liquor)) unterdrückt werden. Wenn die dem 90°-Impuls folgende Auslesesequenz mit einer langen Echozeit arbeitet (also eine T_2-Wichtung erzeugt wird), lassen sich flüssigkeitsreiche Strukturen (T_2 groß) in der Nähe von Liquorräumen darstellen (T_2 dort ebenfalls groß, jedoch Signalunterdrückung auf Grund von T_1). Dieser Spezialfall wird als FLAIR (Flow Attenuated Inversion Recovery) bezeichnet.

2.2.2.4 Das Turbo-Prinzip

Bei den bisher dargestellten Sequenz-Designs wird für jeden Phasenkodierschritt der vollständige Sequenzzyklus durchlaufen. Wenn nach einem RF-Anregungsimpuls mehrmals hintereinander refokussiert wird, lassen sich innerhalb einer Repetitionszeit T_R mehrere Zeilen des k-Raumes füllen. Um den entsprechenden Faktor („Turbo-Faktor") verkürzt sich die Gesamt-Messzeit (Abbildung 15). Allerdings gilt für die Folge von innerhalb eines Zyklus gemessenen Spin- oder Gradientenechos keine einheitliche Echozeit T_E mehr. Da sich wegen des fortschreitenden, nicht refokussierbaren Anteils an T_2^* die Signalintensitäten innerhalb eines solchen Echozuges unterscheiden, sind in Abhängigkeit davon, mit welchen Echos welche Linien des k-Raumes gefüllt werden, Bildartefakte zu erwarten. In Abhängigkeit davon, welche Echos in welche Position des k-Raumes eingeordnet werden, kann sich bei gleichem Echo-Abstand und gleicher Echozug-Länge eine unterschiedliche Bildwichtung ergeben (vgl. Erklärung zu Abbildung 7). Des weiteren führt die dichte Folge von RF-Impulsen bei Turbo-Spinecho-Sequenzen zu teilweiser Refokussierung des Beitrages der J-Kopplung zur Dephasierung und damit zu einer signalreicheren Darstellung von Fett [20].

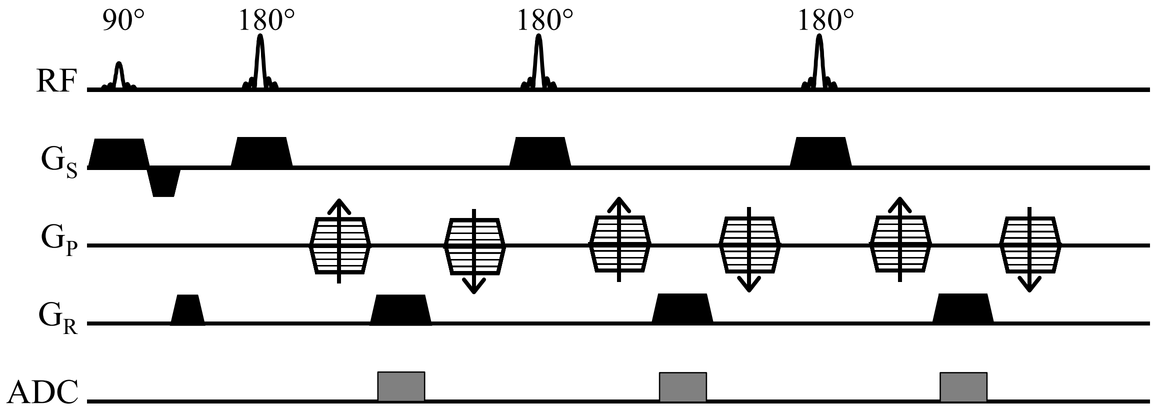

Abbildung 15: Sequenzdiagramm einer Turbo-Spinecho-Sequenz mit dem Turbo-Faktor 3. Die Phasenkodierung des n-ten Schrittes wird vor der Kodierung des (n+1)-ten Schrittes jeweils zurückgestellt.

In völliger Analogie kann auch mittels Gradientenschaltungen ein mehrfacher Wechsel zwischen De- und Rephasierung vorgenommen werden (Turbo-Gradientenecho). Mit Ausnahme der Refokussierung des Beitrages der J-Kopplung entsprechen die Konsequenzen denen bei der Turbo-Spinecho-Sequenz.

2.2.2.5 Echo Planar Imaging (EPI)

Beim Turbo-Gradientenecho wird nach Auslesung jedes Echos die durch den Phasenkodiergradienten erzeugte Phasendispersion zunächst durch einen Gradienten umgekehrter Polarität aufgehoben, ehe der nächste Phasenkodierschritt erfolgt. Wenn auf diese Refokussierung verzichtet wird, kann das der nächsten Zeile im k-Raum entsprechende Gradienten-Zeit-Integral auch durch einen schwachen und/oder kurzzeitigen Phasenkodiergradienten erreicht werden, was eine erhebliche Zeitersparnis darstellt (Abbildung 16).

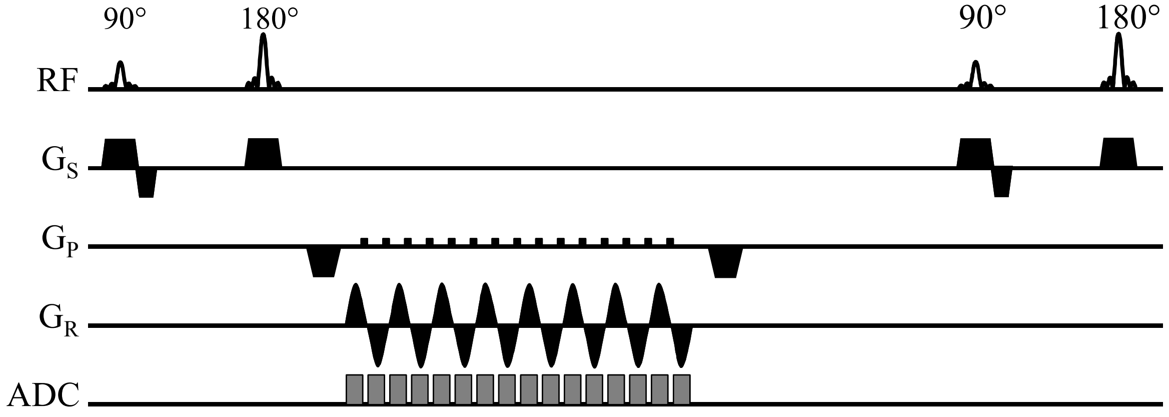

Abbildung 16: Sequenzdiagramm einer spinecho-präparierten EPI-Sequenz.

Die Konsequenz des Verzichts auf Refokussierung ist allerdings, dass sich Phasendifferenzen durch Suszeptibilitätsunterschiede über die gesamte Dauer des Echozuges akkumulieren. Dies resultiert in räumlicher Fehlkodierung in solchen Bereichen bis hin zu destruktiver Interferenz. Betroffen hiervon sind beispielsweise die

Bereiche um die Schädelbasis und Nasennebenhöhlen, aber auch Metallartefakte werden hierdurch erheblich vergrößert (Abbildung 17).

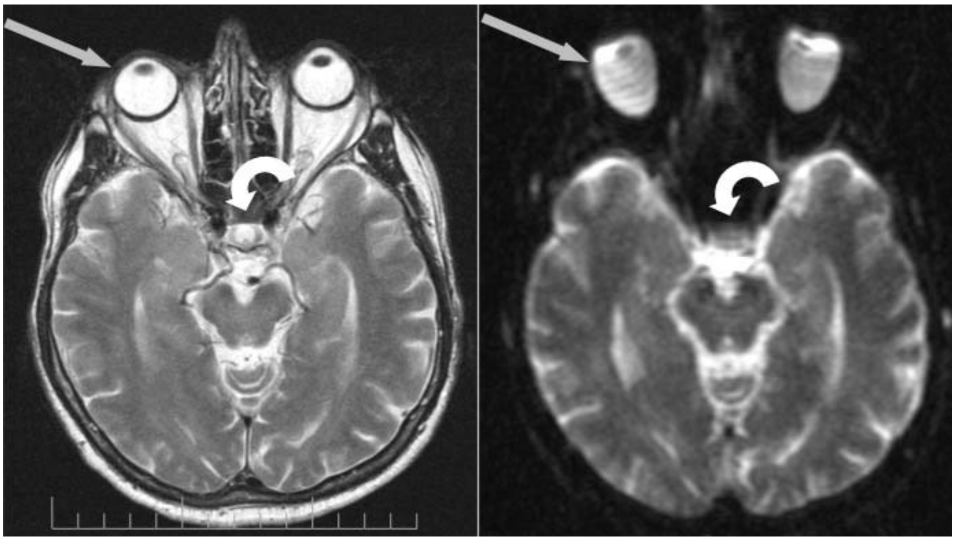

Abbildung 17: Darstellung des Kopfes in Schädelbasis-Nähe. Links: mittels Turbo-Spinecho-, rechts mittels EPI-Sequenz. Gerade graue Pfeile: geometrische Verzerrung des Augapfels. Weiße geschwungene Pfeile: Signalauslöschung im Bereich des Klivus.

Um die Zeitersparnis durch den Verzicht auf Refokussierung in Phasenkodierrichtung maximal zu nutzen, wird auch der Ausleseschritt maximal beschleunigt, indem darauf verzichtet wird, die Signalauslesung nur bei konstantem Frequenzkodiergradienten vorzunehmen. Stattdessen erfolgt die Signalgewinnung auch auf den Flanken eines trapezförmigen Gradientenimpulses. Alternativ werden die für die Erzeugung des Frequenzkodiergradienten eingesetzten Spulen innerhalb eines Schwingkreises betrieben – dies hat einen sinusförmigen Verlauf (oder zumindest Anstieg/Abfall) des Gradienten zur Folge. Unter diesen Bedingungen entspricht ein zeitlich äquidistantes Auslesen von Signalintensitäten nicht mehr äquidistanten Zuwächsen des Gradienten-Zeit-Integrals und damit nicht mehr äquidistanten Schritten im k-Raum. Diese werden entweder durch zeitlich nicht-äquidistantes Auslesen oder durch nachträgliche Interpolation erzeugt. Durch alternierende Vorzeichen des Frequenzkodiergradienten refokussiert der jeweils (n+1)-te den n-ten Gradienten. Dieses wechselnde Vorzeichen führt dazu, dass der k-Raum in alternierender Richtung gefüllt wird. Eventuelle Fehler im Gleichspannungsanteil des Signals (der Null sein sollte), führen dadurch zu einem artifiziellen Oszillieren mit maximaler Frequenz im k-Raum. Diese bildet sich nach Fourier-Transformation als maximale Verschiebung in Phasenkodierrichtung ab – das Bild (oder ein Anteil davon) ist um die halbe Bildlänge verschoben (N/2-Artefakt - Abbildung 18).

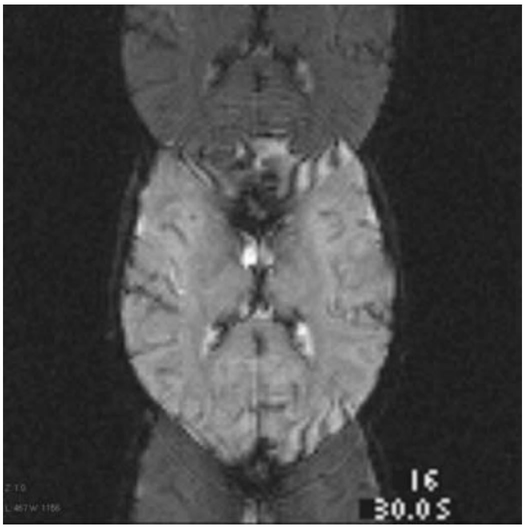

Abbildung 18: N/2-Artefakt bei EPI-Sequenz

2.2.2.6 Präparation

<u>Inversion Recovery:</u> Den oben beschriebenen Sequenzen können verschiedene vorbereitende Abläufe vorangehen. Das Vorgehen entspricht im wesentlichen dem bei der Diskussion der Inversion-Recovery-Sequenz beschriebenen Ablauf (Abschnitt 2.2.2.3). Dort wurde die Spin-Inversion (180°-Impuls, Wartezeit T_I) einer Spinecho-Auslesesequenz vorgeschaltet. Anwendbar sind aber ebenso die im Weiteren beschriebenen Ausleseprinzipien wie z. B. Turbo-Spinecho (TIRM = Turbo Inversion Recovery Modulus (Betragsdarstellung des komplexen Signals)) oder EPI.

<u>Spinecho:</u> In analogem Herangehen ist der in Abbildung 16 dargestellten EPI-Auslese-Impulsfolge eine Spinecho-Präparation vorangestellt worden.

<u>Saturation Recovery:</u> Die Magnetisierung in z-Richtung wird mittels 90°-Impuls in die xy-Ebene geklappt und durch einen Spoiling-Gradienten dephasiert. Die nach einer Wartezeit sich durch T_1-Relaxation wieder aufbauende Magnetisierung in z-Richtung wird durch einen zweiten 90°-Impuls in die xy-Ebene gebracht und durch eine Auslese-sequenz gemessen.

<u>Vorsättigung:</u> Pulsierende Gefäße oder die durch die Atmung bewegte Bauchdecke können zu Artefakten führen, die sich in Phasenkodierrichtung über das Bild ausbreiten. Entsprechende Bereiche können z. B. mittels 90°-Impuls ($M_z \rightarrow 0$) und anschließendem Spoiling mittels starker Gradienten ($M_{xy} \rightarrow 0$) vor der eigentlichen bilderzeugenden Sequenz unterdrückt werden.

Die Vorsättigung kann statt schichtselektiv auch frequenzselektiv erfolgen. In CH_2-Gruppen (überwiegend Fettmoleküle) gebundene Protonen besitzen eine um 3,5 ppm niedrigere Resonanzfrequenz als in H_2O gebundene Protonen. Durch einen schmalbandigen RF-Impuls entsprechender Frequenz und nachfolgendes Spoiling kann der Fett-Anteil am Bild unterdrückt werden. Analoge Überlegungen führen zur selektiven Wasserunterdrückung oder aber auch zur selektiven Wasser- oder Fett-anregung. Voraussetzung für diese frequenzselektiven Methoden ist ein sehr homogenes Magnetfeld.

Magnetization Transfer: An Proteine gebundene Wasserstoffatome weisen eine relativ starre Bindung auf. Die ausgeprägte Dipol-Dipol-Wechselwirkung führt zu einer relativ kurzen Spin-Spin-Relaxationszeit [154] in der Größenordnung von < 1 ms. Aus der umgekehrten Proportionalität zwischen T_2^* und der Linienbreite auf halber Peakhöhe $\Delta\nu$ ([87], Gleichung (18))

$$\Delta \nu = \frac{1}{\pi * T_2^*} \tag{18}$$

resultiert eine Linienbreite in der Größenordnung von 10 kHz [7]. Daher können die Protein-Protonen mit einer „Off-Resonance"-Frequenz angeregt werden (Abbildung 19).

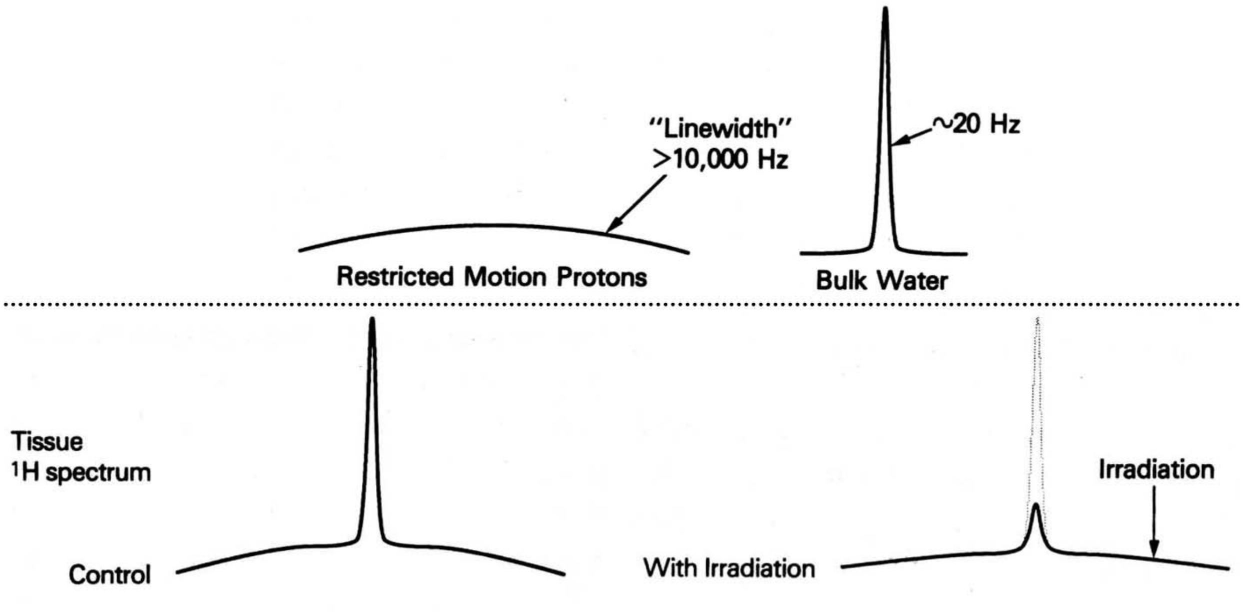

Abbildung 19: Anwendung von Magnetization Transfer zwischen an Proteine und in Wassermolekülen gebundenen Protonen zur Signalreduktion von Wasser. Oben: Linienformen für relativ starr gebundene Protonen bzw. Wasser, Unten links: Kombination wie in biologischem Gewebe, Unten rechts: Unterdrückung des Wassersignals durch Sättigungsübertragung von den „off resonance" angeregten Protein-Protonen. (aus [7])

Wegen ihrer sehr kurzen T_2 tragen diese Protonen in der Bildgebung nicht signifikant zum Signal bei. Sie tauschen aber mit in Wassermolekülen gebundene Protonen aus („Magnetization transfer"). Das Vorschalten solcher Off-Resonance-Impulse wird zur Kontrast-Modifikation (MTC = Magnetization Transfer Contrast), aber auch zur Sättigung von stationärem Gewebe in MR-Angiographie-Sequenzen eingesetzt.

2.2.3 Gerätetechnik für die MR-Tomographie

Magnet: Wesentliche Voraussetzung für ein NMR-Experiment ist ein statisches Magnetfeld. Zwar ist prinzipiell bereits im Erdmagnetfeld (ca. 0,00005 Tesla) Bildgebung möglich, aber höhere Magnetfelder führen zu einem ausgeprägteren Besetzungsunterschied der Spinzustände (Gleichung (4)) und damit zu stärkerem Signal. Klinische MR-Ganzkörper-Scanner weisen daher ein statisches Magnetfeld zwischen 0.2 T und 3 T auf, bei Forschungsgeräten erstreckt sich die Feldstärke bis zu

4 T [31]. Für Körperteile spezialisierte Geräte (Kopf, Extremitäten) werden mit Feldstärken um 0,2 T betrieben. Während sich niedrige Feldstärken und Abmessungen mit Permanent- oder Widerstandsmagneten realisieren lassen, werden diagnostische Ganzkörper-Geräte heute fast ausschließlich mit supraleitenden Magneten ausgestattet. Geschlossene Bauweisen erleichtern das Erzielen einer möglichst hohen Magnetfeld-Homogenität. Für Interventionen unter MR-Kontrolle sind jedoch auch offene Geometrien verfügbar (Tabelle 4).

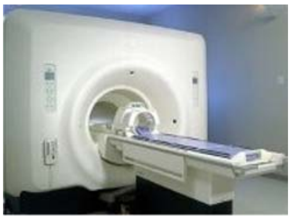	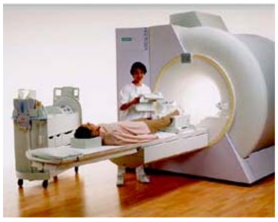	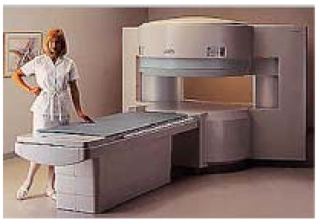	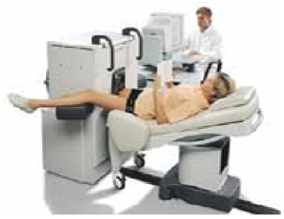
Signa 3T (General Electrics)<br>Ganzkörpertomograph<br>3,0 Tesla<br>supraleitend	Magnetom Symphony (Siemens)<br>Ganzkörpertomograph<br>1.5 Tesla<br>supraleitend	AIRIS (Hitachi)<br>offenes System<br>0.3 Tesla<br>Permanentmagnet	ARTOSCAN (Esaote)<br>Extremitätenscanner<br>0.2 Tesla<br>Permanentmagnet

Tabelle 4: MR-Scanner verschiedener Bauart und Feldstärke (Abbildungen von den Websites der Hersteller)

Zusätzliche Shim-Spulen ermöglichen es, das durch in den Magneten eingebrachte Objekte (i.d.R. den Patienten) beeinflusste Feld zumindest lokal zu homogenisieren.

<u>Gradientensystem:</u> Die für die Ortskodierung, aber auch für die Zerstörung von Magnetisierung in der xy-Ebene erforderlichen Magnetfeld-Gradienten werden im wesentlichen durch je eine fest in den Magneten integrierte Spule pro orthogonaler Raumrichtung erzeugt. Das nutzbare Bildfeld wird im wesentlichen durch die Abmessungen dieser Spulen bestimmt. Die Linearität der Gradienten setzt sich direkt in geometrische Abbildungstreue um.

Im Interesse einer möglichst schnellen Bildgebung sind möglichst starke und schnell schaltbare Gradienten wünschenswert. Dies ist insbesondere für kardiologische und neurologische Fragestellungen von Bedeutung. Da zeitlich veränderliche Magnetfelder Ladungsverschiebungen in den Nerven induzieren, sind Gradientenstärke und ~anstiegsgeschwindigkeit jedoch physiologische Grenzen gesetzt [58]. Da bei gegebener Gradientenanstiegsgeschwindigkeit (in mT/(m*s)) die zeitliche Änderung des Magnetfeldes linear mit dem Abstand zum Zentrum der Gradientenspule anwächst, werden Geräte mit hohen Gradientenfeldstärken heute mit kürzeren Gradientenspulen (z. B. Siemens Symphony, Sonata) oder aber mit einem kurzen und einem langen Gradientensystem für maximales Bildfeld oder maximale Performance (General Electrics TwinSpeed) ausgerüstet. Die mit dem Schalten der Gradienten verbundenen Kräfte und damit Deformationen sind für die Lärmentwicklung bei kernspintomographischen Messungen – insbesondere bei EPI-Sequenzen – verantwortlich. Die durch Induktion von Wirbelströmen beim Schalten der Gradienten verursachten Magnetfelder verringern die Bildqualität (Verzerrungen, Signalverlust).

Durch aktive Abschirmung, d.h. Aufbau eines dem gewünschten Gradientenfeld entgegengesetzt gerichteten Magnetfeldes können sowohl Wirbelströme reduziert als auch die Gradientenfelder im wesentlichen auf die Größe des Scanners beschränkt werden.

Sende- und Empfangsspulen: Aus Gleichung (6) ist ersichtlich, dass der Anregungswinkel eines RF-Impulses u.a. proportional zur Feldstärke des elektromagnetischen Wechselfeldes ist. Daraus resultiert die Notwendigkeit eines hinreichend homogenen RF-Feldes über den darzustellenden Bereich. Das zu empfangende Signal ist relativ schwach und u.a. durch thermisches Rauschen kontaminiert. Ein homogenes RF-Feld lässt sich mit großen Sendespulen leichter erzeugen. Da sich das Signal-Rausch-Verhältnis mit der Größe des durch die Empfangsspule erfassten Volumens verschlechtert, sollte eine Empfangsspule sowohl möglichst dicht am darzustellenden Bereich lokalisiert sein als auch möglichst nur aus diesem empfangen. Aus diesen unterschiedlichen Anforderungen resultiert, dass zum Teil mit getrennten Sende- und Empfangsspulen gearbeitet wird, wobei als Sendespule häufig der fest in den Magneten integrierte Ganzkörper-Resonator verwendet wird. Für die Untersuchung verschiedener Körperregionen sind Empfangsspulen, aber auch kombinierte Sende-/Empfangsspulen unterschiedlicher Geometrie erforderlich.

Bei der Darstellung größerer Regionen werden die unterschiedlichen Anforderungen nach einer großen Empfangsspule im Interesse eines großen Field of View einerseits und einer möglichst kleinen Spule im Interesse eines optimalen Signal-Rausch-Verhältnisses vereint, indem das Field of View mit einer Kombination mehrerer kleiner Spulen eingesehen wird („phased array coils“).

Die in der xy-Ebene rotierende Nettomagnetisierung führt zur Emission zirkular polarisierter Radiofrequenz. Quadratur-Empfangsspulen tragen dem Rechnung, indem sie aus zwei orthogonal zueinander angeordneten Teilspulen bestehen. Dies erlaubt eine um (theoretisch) $\sqrt{2}$ höhere Signalausbeute als mit einer Einzelspule.

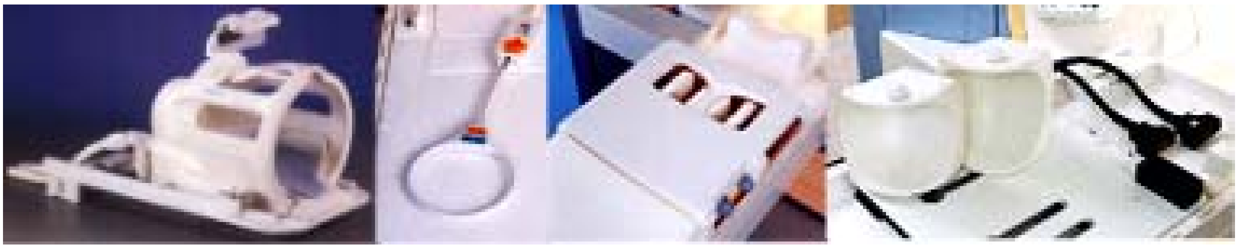

Abbildung 20: Oberflächenspulen. Von links nach rechts: Schädel-, Ring-, Mamma- und Schulterspule (Abbildungen von Websites der Fa. Siemens und MRI Devices Cooperation)

Hochfrequenz-System: Das Hochfrequenzsystem muss in der Lage sein, verschiedene Pulsformen sehr frequenz- und phasenstabil, reproduzierbar und mit relativ hoher Leistung (einige Kilowatt Spitzenleistung und einige Hundert Watt Dauerleistung) abzugeben. Frequenz und Pulsform bestimmen zusammen mit dem während des Sendens geschalteten Gradienten den Bereich, in dem die Resonanzbedingung (Gleichung (2)) erfüllt ist. Dieser Bereich, d.h. das Schichtprofil und damit auch die

Frequenzverteilung eines Anregungsimpulses, sollten i.A. rechteckig sein. Da die Fourier-Transformierte einer Rechteck-Funktion die sinc-Funktion ist, werden zur Schichtanregung häufig sinc-förmige RF-Impulse angewandt. Um Nebenmaxima außerhalb der anzuregenden Schicht zu unterdrücken, wird für eine kontinuierliche Annäherung des zeitlich endlichen sinc-Impulses an Null gesorgt (Apodisation – Abbildung 21). Je nach Sequenzdesign sollen sich Magnetisierungen über mehrere Anregungszyklen addieren oder aber voneinander unabhängig aufbauen. Die relative Phasenlage aufeinanderfolgender Anregungsimpulse kann benutzt werden, um unerwünschte Summationen von xy-Magnetisierungen zu vermeiden (RF-Spoiling).

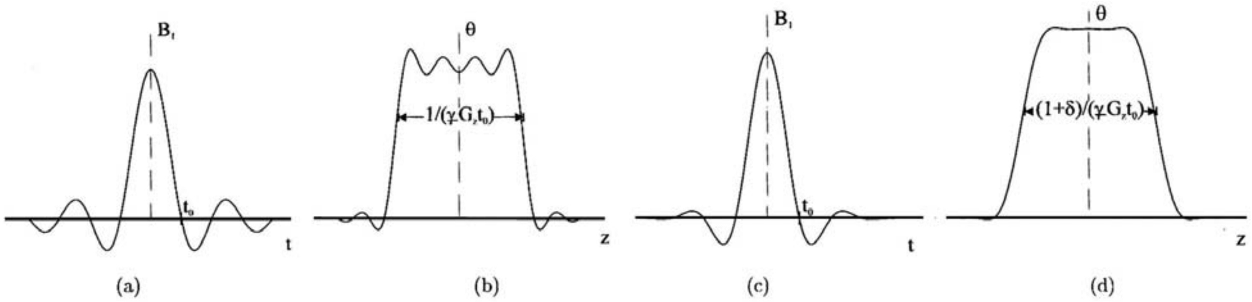

Abbildung 21: sinc-förmiger RF-Impuls in der Zeitdomäne (a, c) und Frequenzdomäne (b, d). Links (a, b) ohne, rechts (c, d) mit Apodisation (Hanning-Funktion) (aus [52]).

Computertechnik: Das Computersystem eines MR-Scanners hat eine Vielzahl von Aufgaben zum Teil parallel zu bearbeiten, u.a.

- Erzeugung der RF-Impulsformen und Ansteuerung der Leistungselektronik
- Erzeugung der Gradienten-Impulsformen und Ansteuerung der Leistungselektronik
- Aufnahme und Zwischenspeicherung der Messdaten (Datenrate einige 10 bis einige 100 kByte/s)
- Bildrekonstruktion (ca. 50 bis 200 Bilder pro Patient, Bildmatrix 128^2 bis 1024^2 (meist 256^2), Bildinformation in Größenordnung von 15 bis 20 MByte/Patient)
- Bildverwaltung (Datenbank) und –kommunikation (DICOM)
- Bilddarstellung und –verarbeitung
- Kommunikation mit Nutzer (Bedienoberfläche)

Diese Menge von zum Teil zeitkritischen Prozessen wird häufig mittels Dezentralisation der Rechenleistung bewältigt. Der Nutzer kommuniziert in der Regel nur mit einem Steuerrechner.

2.2.4 Physikalische Effekte bei Anwendung paramagnetischer Kontrastmittel

2.2.4.1 Eigenschaften der Kontrastmittel

Paramagnetische Substanzen lassen sich auf Grund der Eigenschaften ihrer Elektronenhülle in vier Klassen einteilen [181]:

1. Stoffe, deren Atome, Moleküle oder Radikale eine ungerade Anzahl von Elektronen haben. Deren Gesamtspin ist dadurch ungleich Null.

2. Stoffe mit teilweise gefüllten inneren Elektronenschalen (z. B. Übergangselemente, Seltene Erden, Aktinide)

3. Stoffe mit speziellen Besetzungen der Elektronenzustände im Molekül (z. B. O_2)

4. viele Metalle (durch Orientierung der Leitungselektronen im äußeren Magnetfeld)

Die am häufigsten klinisch eingesetzten Kontrastmittel enthalten Atome Seltener Erden, die aus Gründen der Verträglichkeit in Makromolekülen integriert sind (z. B. Gadolinium-Diethyltriamin-Pentaazetat (Gd-DTPA)).

Kontrastmittel auf Basis von Metallen werden auf Grund ihrer sehr starken Verkürzung der T_2- bzw. T_2^*-Relaxationszeit im klinischen Sprachgebrauch auch als superparamagnetisch bezeichnet.

Die durch thermische Bewegung der Moleküle paramagnetischer Kontrastmittel bewirkten schnellen Magnetfeld-Fluktuationen beschleunigen die Energieübertragung zwischen den im NMR-Experiment angeregten Protonen und deren Umgebung. Dies wird durch die Verkürzung der T_1-Relaxationszeit beschrieben. Gleichzeitig führen diese stochastischen Magnetfeld-Fluktuationen auch zur beschleunigten Dephasierung, also zu einer Verkürzung der T_2- bzw. T_2^*-Relaxationszeit [46, 177, 147, 18]:

$$\Delta R_i = \frac{1}{T_{i,obs}} - \frac{1}{T_i} = r_i * C \qquad (19)$$

Gleichung (19) beschreibt den Zusammenhang zwischen Kontrastmittelkonzentration und Veränderung der Relaxationszeit T_i bzw. Relaxationsrate ΔR_i (i = (1; 2)) mit der Relaxivität r_i als Proportionalitätsfaktor. Der Index *obs* steht für die nach Kontrastmittelgabe beobachtete Relaxationszeit. Für die Spin-Gitter-Relaxation wurde die näherungsweise lineare Beziehung zwischen Änderung der Relaxationsrate und KM-Konzentration 1948 von Bloembergen, Purcell und Pound abgeleitet [12] (Gleichung (20)):

$$\Delta R_1 = \frac{12 * N * \pi^2 * \gamma^2 * \eta * \mu^2}{k * T} \qquad (20)$$

mit N = Anzahl der Ionen pro cm³, μ = magnetisches Moment des Ions, γ = gyromagnetisches Verhältnis, T = Temperatur, k = Boltzmann-Konstante.

Der lineare Zusammenhang zwischen ΔR_1 und der Gadolinium-Konzentration wurde experimentell [170] bis zu einer Konzentration von > 2 mM in Agar und Blut bzw. mittels Computersimulation [171] bestätigt. Für nicht zu große Kontrastmittel-Konzentrationen kann in guter Näherung der T_1-bedingte relative Signalintensitäts-Zuwachs als proportional zur KM-Konzentration angenommen werden (Ableitung in [17], siehe auch Abbildung 43). Der Proportionalitätsfaktor zwischen N und ΔR_1 erweist sich im Experiment allerdings als stark von der Umgebung des Kontrastmittels (Lösungsmittel, weitere gelöste Stoffe) abhängig. Die Ursache für diese Diskrepanz liegt in der Vernachlässigung des Frequenzspektrums der vom betrachteten Proton „gesehenen“ Magnetfeld-Fluktuationen. Dieses Spektrum hängt u.a. von der Größe der umgebenden Moleküle ab. Nur die der Larmor-Frequenz (genauer: dem Zweifachen davon) entsprechende Komponente kann T_1-Relaxation vermitteln [47]. Aus diesem Grund dürfte auch die Temperaturabhängigkeit dieses Proportionalitätsfaktors komplexer sein, als es die ausschließliche Berücksichtigung der Energiedifferenz zwischen den Spinzuständen in Gleichung (20) vorhersagt. Insbesondere aber wird dadurch erklärt,

dass die Relaxationsrate durch an Makromoleküle gebundene Metalle deutlich stärker erhöht werden kann als durch die selbe Konzentration von freien Metallionen – Makromoleküle führen zu langsameren Magnetfeldfluktuationen und damit zu einem größeren Anteil von in der Nähe der doppelten Larmor-Frequenz liegenden Frequenzanteilen (Abbildung 22 links). Dieser Effekt kann jedoch überkompensiert werden, wenn das Makromolekül auf Grund seiner Struktur den Zugang von Wasser-Protonen behindert, wie das bei den klinisch wegen ihrer guten Verträglichkeit häufig angewandten Chelaten der Fall ist (Abbildung 22 rechts).

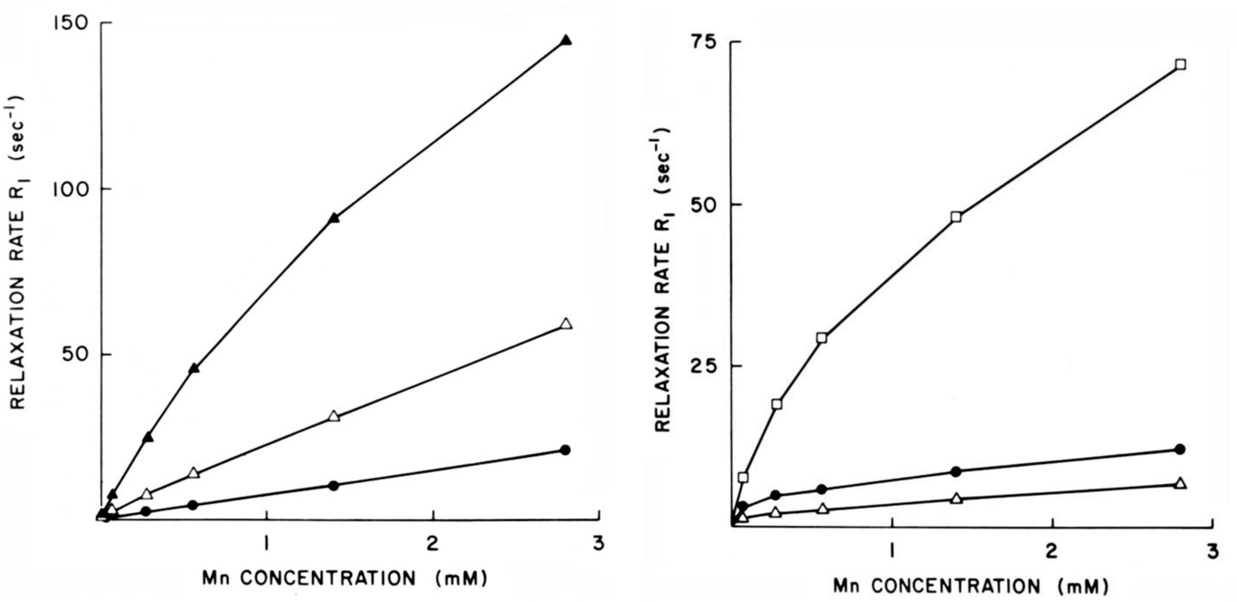

Abbildung 22: Abhängigkeit der Relaxationsrate R_1 von der Mangan-Konzentration. Links: ● freies Mangan, ∆ an γ-Globulin gebunden, ▲ an Albumin gebunden. Rechts: □ freies Mangan, ● Mn-EDTA, ∆ Mn-DTPA in Blut mit $MnCl_2$ (aus [47])

Die ebenfalls hinreichend lineare Beziehung zwischen ΔR_2 und der Konzentration im Kapillarbett ist z. B. in [148] beschrieben.

In dem Beitrag von Gore et al. [47] wird die Abhängigkeit der Signalintensitäten für Spin- und Gradientenechosequenzen von der Autokorrelationszeit der Magnetfeld-fluktuationen τ_c, unter Benutzung der „Anderson-Weiss mean field theory“ [1] dargestellt (Gleichungen (21) und (22)):

$$E(TE) = \exp\left[-(\Delta\omega_0^2)\tau_c^2 * \left\{ 4 * \exp\left(\frac{-T_E}{2 * \tau_c} \right) - \exp\left(\frac{-T_E}{\tau_c} \right) + \frac{T_E}{\tau_c} - 3 \right\} \right] \qquad (21)$$

$$G(TE) = \exp\left[-(\Delta\omega_0^2)\tau_c^2 * \left\{ \exp\left(\frac{-T_E}{\tau_c} \right) + \frac{T_E}{\tau_c} - 1 \right\} \right] \qquad (22)$$

Für Spins, die sich hinreichend langsam an Magnetfeldstörungen vorbeibewegen (genauer: für die die Autokorrelationszeit der Magnetfeldfluktuationen größer als die Echozeit wird), wird ein mit zunehmender Autokorrelationszeit wachsender Anteil der Dephasierung durch Spinecho-Sequenzen refokussiert. Unter diesen Bedingungen, die

bei Perfusionsmessungen vorliegen, wird mittels Gradientenecho eine stärkere Erhöhung der Relaxationsrate und damit ein ausgeprägterer Signalverlust beobachtet (Abbildung 23, Abbildung 24).

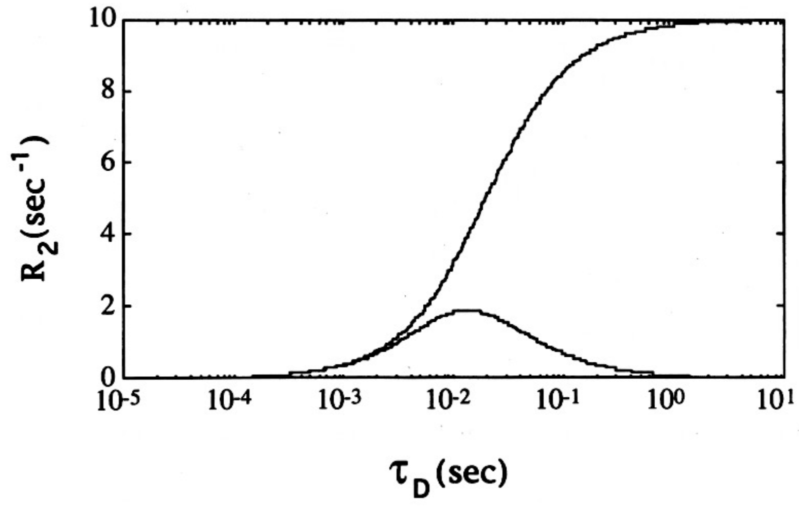

Abbildung 23: Darstellung der Abhängigkeit der Relaxationsraten-Änderung ΔR_2 von der Autokorrelationszeit der lokalen Magnetfeldfluktuationen τ_C für Gradientenecho (oben) und Spinecho (unten) gemäß Gleichungen (21) und (22) (aus [47])

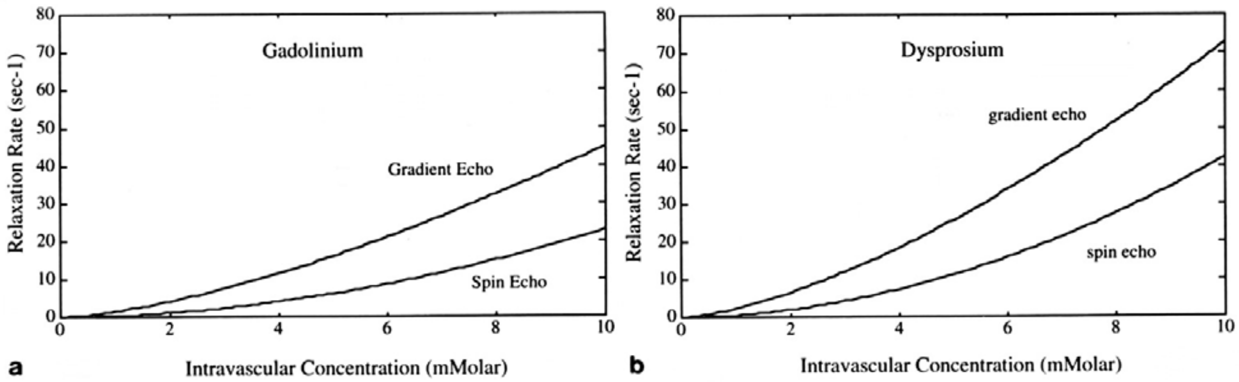

Abbildung 24: Relaxationsraten R_2^* von Gd-DTPA (links) und Dy-DTPA (rechts) für T_E = 50 ms. Volumenanteil der Kapillaren 3 %, Diffusionskoeffizient D = 0.65*10^{-5} cm²/s, parallele Kapillaren von 5 µm Durchmesser (aus [47])

2.2.4.2 Aufnahme eines paramagnetischen Kontrastmittels (statisch)

Die Gabe eines paramagnetischen Kontrastmittels gehört bei einer Vielzahl von mittels Kernspintomographie bearbeiteten diagnostischen Fragestellungen zu den Standardverfahren. Dabei ist es meist ausreichend, den Grad der Kontrastmittel-aufnahme in der zu untersuchenden Region anhand der durch die T_1-Verkürzung bewirkten Signalzunahme qualitativ mit der Umgebung zu vergleichen und zu bewerten (schwacher - mäßiger - starker Intensitätszuwachs sowie morphologisch zu diskutieren (groß / klein, singulär / multipel, glatt / unscharf begrenzt). Da die Relaxationszeiten T_1

und T_2 bzw. T_2^* gleichzeitig verkürzt werden, muss durch die Gestaltung der Messsequenz für eine "T_1-Wichtung" der gewonnenen Bilder gesorgt werden. Das heißt, eine prozentuale Veränderung der Spin-Gitter-Relaxationsrate muss die Signalintensität in höherem Maße beeinflussen als eine vergleichbare Veränderung anderer kontrastbeeinflussender Parameter (hier insbesondere T_2 bzw. T_2^* und die Protonendichte). Dies wird bei Spinecho- und Gradientenecho-Sequenzen durch kurze Repetitionszeiten T_R bei möglichst kurzen Echozeiten T_E erreicht, wie sich aus den Signalintensitätsformeln für diese Sequenzen (Gleichung (14) für Spinecho, Gleichung (15) für Gradientenecho) durch Vergleich von deren partiellen Differentialen nach diesen Einflussgrößen zeigen lässt.

Aus den Vorzeichen der partiellen Differentiale ist ersichtlich, dass eine verkürzte T_1-Relaxationszeit auf Grund des schnelleren Wiederaufbaus der Nettomagnetisierung in z-Richtung zu einem Signalzuwachs und eine verkürzte T_2- bzw. T_2^*-Relaxationszeit auf Grund der schnelleren Dephasierung zu einer Signalverringerung führt.

Diese Aussagen gelten in für qualitative Betrachtungen ausreichender Näherung auch für die "Turbo"-Modifikationen dieser Standardsequenzen.

Da prinzipiell zwischen Anregung des Spinsystems mittels RF-Impuls und dem Lesen des Signals ein gewisser zeitlicher Abstand nicht zu vermeiden ist (auch bei FID-Sequenzen), sind auch mit T_1-gewichteten Sequenzen gewonnene Bilder vom mit T_2 bzw. T_2^* beschriebenen Zerfall der Nettomagnetisierung in der xy-Ebene (senkrecht zum Hauptmagnetfeld) beeinflusst. Da paramagnetische Kontrastmittel nun sowohl T_1 als auch T_2 bzw. T_2^* verkürzen, kann eine relativ hohe Kontrastmittelkonzentration sich auf Grund einer starken T_2- bzw. T_2^*-Verkürzung im T_1-gewichteten Bild als Signalverlust darstellen (Abbildung 25).

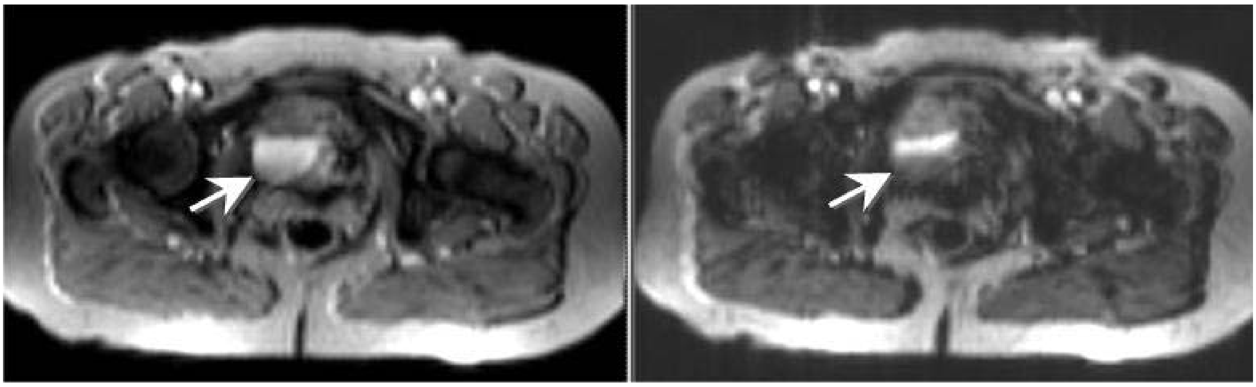

Abbildung 25: Kontrastmittel-bedingte Signalveränderungen in der Harnblase. Links stärker T_1-, rechts T_2^*-gewichtetes Signal einer Doppelecho-FLASH-Sequenz. Die durch Sedimentation nach unten ansteigende KM-Konzentration bewirkt bereits in der T_1-Wichtung einen Signalabfall verglichen zur Intensität in der Nähe des Flüssigkeitsspiegels. Bei längerer Echozeit (rechts) noch deutlichere T_2-bedingte Signalreduktion.

2.2.4.3 Aufnahme eines paramagnetischen Kontrastmittels (dynamisch)

Im einfachsten Fall werden der Grad und die Geschwindigkeit der durch die kontrastmittel-bedingte T_1-Verkürzung verursachten Signalzunahme qualitativ oder semiquantitativ bewertet. Intensiver und schneller Signalzuwachs kann dann unter gewissen messtechnischen und physiologischen Bedingungen als Hinweis auf ein

malignes Geschehen interpretiert werden. Als Ursachen dafür werden u.a. die Tumor-Neoangiogenese, sowie eine gestörte Permeabilität der Kapillarwände im Tumor mit daraus resultierender vermehrter Extrazellularflüssigkeit und damit erhöhter Aufnahmefähigkeit für Kontrastmittel diskutiert [z. B. 125].

Für genauere quantitative Analysen ist die Betrachtung eines pharmakokinetischen Multikompartment-Systems sinnvoll, wie es z. B. von Parker et al. [137] beschrieben wird. Das Kontrastmittel wird in das Plasma-Kompartment injiziert. Von dort aus diffundiert es in den Extrazellular-Raum des gesamten Körpers, insbesondere auch in den der untersuchten Läsion, welcher gesondert betrachtet wird. Außerdem wird es über die Nieren ausgeschieden.

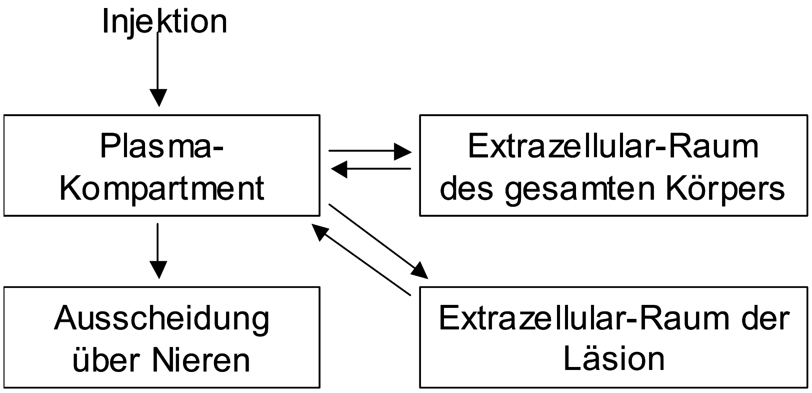

Abbildung 26: Vier-Kompartment-Modell für quantitative Beschreibung der Kontrastmittelaufnahme einer Läsion (nach [137])

Nach intravenöser Injektion eines Kontrastmittel-Bolus fällt die Konzentration etwa biexponentiell ab [167] (Gleichung (23))

$$C_p^{bol}(t) = D^{bol} * \sum_{i=1}^{2} a_i * \exp(-m_i * t) \qquad (23)$$

mit C_P^{bol} = KM-Konzentration im Blut, D^{bol} = KM-Dosis der Bolus-Injektion, i = 1 für den Austausch zwischen dem Plasma und dem extrazellulären Wasser bzw. i = 2 für die renale Ausscheidung (welche der deutlich langsamere Prozess ist). Weinmann et al. geben für die KM-Verteilung am Menschen folgende Parameter an: a_1 = 3,99 kg/l, a_2 = 4,78 kg/l, m_1 = 0,144 min^{-1}, m_2 = 0,0111 min^{-1} [178]. Die Austauschprozesse zwischen den Kompartments können als Diffusionsprozesse aufgefasst werden, die Lösung des 1. Fickschen Diffusionsgesetzes (24)

$$\frac{\partial C}{\partial t} = -D * A * \frac{\partial C}{\partial x} \qquad (24)$$

mit C als Konzentration, D dem Diffusionskoeffizienten, A der Fläche, durch die die Diffusion erfolgt und $\partial C/\partial x$ dem Konzentrationsgradienten führt für die Zeitabhängigkeit der KM-Konzentration innerhalb einer Läsion auf eine Kombination von Exponentialfunktionen.

Tofts et al. [167] leiteten für die Kontrastmittel-Dynamik der Retina ab, dass mit einer Bolus-Injektion stets eine höhere KM-Gewebe-Konzentration erreicht wird als mit einer

Infusion der gleichen KM-Menge. Diese Ableitung basiert auf den Annahmen einer relativ geringen Permeabilität sowie eines großen „leakage space" der Läsion (Anteil des Läsions-Volumens, der KM aufnehmen kann, am Gesamtvolumen).

Zur Beschreibung der Zeitabhängigkeit der KM-Konzentration im Gewebe benutzte der Autor folgende Annahmen:

- beliebig schnelle Bolus-Injektion des KM
- monoexponentielle Ausscheidung aus dem Blutpool (Vereinfachung im Vergleich zu Weinmann et al. [178])
- richtungsunabhängiger, diffusions-ähnlicher KM-Austausch zwischen Blut und Gewebe

Hiermit lässt sich ein biexponentieller Konzentrationsverlauf ableiten (vgl. Abschnitt 5.3.2, Gleichung (70))

Unter den Annahmen einer beliebig schnellen Bolus-Injektion des KM, einer (im Vergleich zu Weinmann et al. [178] vereinfachend) monoexponentiellen Ausscheidung aus dem Blutpool sowie einem richtungsunabhängigen, diffusions-ähnlichen KM-Austausch zwischen Blut und Gewebe leitete der Autor eine biexponentielle Zeitabhängigkeit der KM-Konzentration im Gewebe ab (vgl. Abschnitt 5.3.2, Gleichung (70)). Für zwei Gewebe, die sich nur durch ihren Diffusionskoeffizienten unterscheiden, bei denen also insbesondere die zur Diffusion zur Verfügung stehende Gefäßfläche pro Gewebsvolumen sowie die Gefäßgeometrie übereinstimmen, ergibt sich damit die in Abbildung 27 dargestellte Situation:

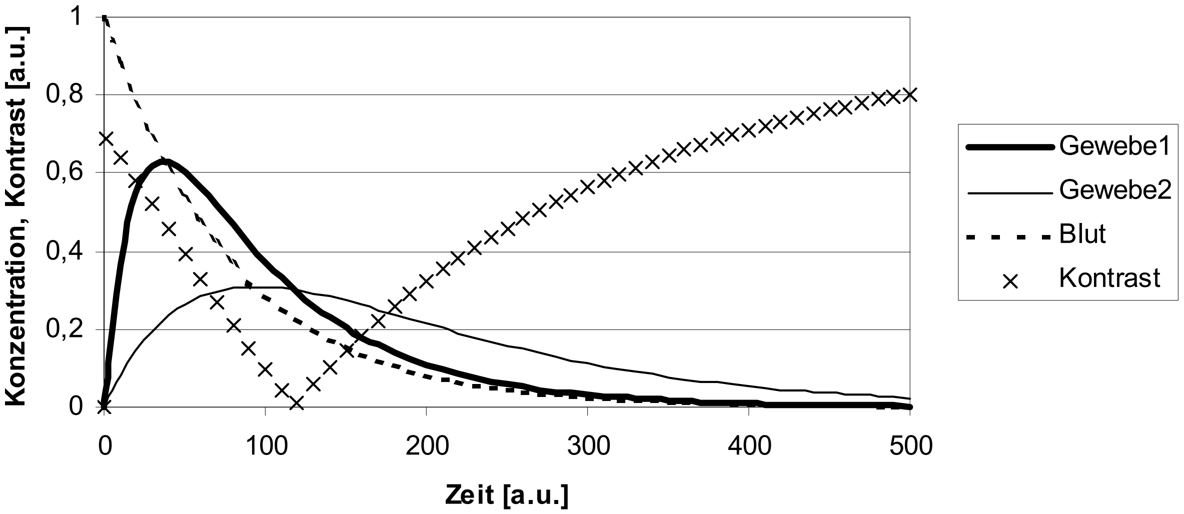

Abbildung 27: KM-Konzentrationsverläufe in Geweben, die sich nur durch ihren Diffusionskoeffizienten voneinander unterscheiden sowie der durch die KM-Konzentrationen ausgebildete Kontrast (dargestellt ist der Betrag $|(C_2 - C_1) / (C_2 + C_1)|$).

Diese Situation wird nach Auffassung des Autors in der MR-tomographischen Untersuchung des Herzmuskels genutzt. Unmittelbar nach KM-Injektion bildet sich ein Konzentrationskontrast auf hohem Konzentrationsniveau (und damit bei gutem erreichbarem Kontrast-Rausch-Verhältnis) aus. Für dessen Nachweis steht jedoch nur

ein relativ kurzes Zeitfenster zur Verfügung. Da das schneller KM aufnehmende Gewebe dieses auch schneller wieder abgibt, bildet sich nach hinreichend langer Zeit erneut ein Kontrast, diesmal mit umgekehrtem Vorzeichen (und auf niedrigerem Konzentrations-Niveau), der sich langsamer verändert als vor seinem Nulldurchgang und gegen einen Maximalwert strebt (wobei alle KM-Konzentrationen und damit auch das Kontrast-Rausch-Verhältnis gleichzeitig gegen Null gehen). In verschiedenen aktuellen Arbeiten wird dieser Effekt, das „delayed enhancement“ zum Auffinden infarzierter [139, 44] oder narbiger [183] Areale des Myokards benutzt.

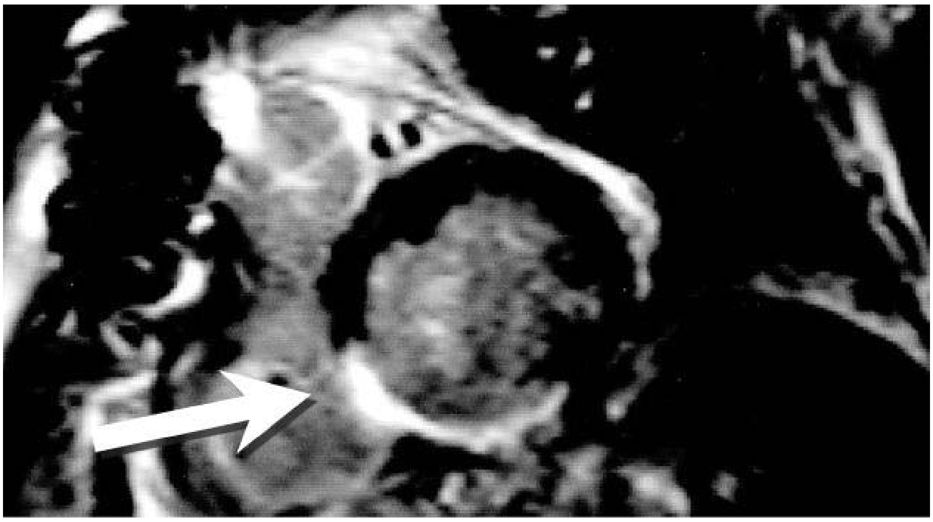

Abbildung 28: Delayed enhancement einer vernarbten Herzmuskelregion (Pfeil) (aus [139], modifiziert)

2.2.4.4 Passage eines paramagnetischen Kontrastmittels

Die Anwesenheit eines paramagnetischen Kontrastmittels wie Gd-DTPA bewirkt eine Verkürzung sowohl der Spin-Gitter-(T_1-) als auch der Spin-Spin-(T_2-)Relaxationszeit. Die durch das KM erheblich verstärkten Suszeptibilitätsunterschiede (vgl. Abschnitt 2.2.4; [122]) führen zu einer beschleunigten Dephasierung, deren Gesamtverlauf (einschließlich der T_2-bedingten Komponenten) mit der Zeitkonstanten T_2^* beschrieben wird.

Die intravaskuläre Kontrastmittelkonzentration wird als in guter Näherung proportional zur Änderung der Relaxationsrate ΔR_2 behandelt (Gleichung (19), [14, 177, 171]). Daraus ergibt sich für Sequenzen mit einer exponentiellen Abhängigkeit der Intensität von der T_2^*-Relaxiationszeit gemäß Gleichung (25)

$$S \sim \exp(-T_E / T_2^*) \qquad (25)$$

im Falle der Messung einer Zeitreihe mittels einer T_2^*-gewichteten Einzelecho-Sequenz Gleichung (26)

$$C(t) = -\frac{k}{T_E} \ln(S(t) / S(t_0)) \qquad (26)$$

t_0 ist ein Zeitpunkt vor Beginn der Kontrastmittelgabe (Grundlinie). Die Forderung gemäß Gleichung (25) ist für Gradientenecho-Sequenzen exakt sowie Spinecho-Sequenzen in guter Näherung erfüllt.

Die am Anfang dieses Abschnittes postulierte Proportionalität zwischen intravasaler KM-Konzentration C und Änderung der Relaxationsrate ΔR_2 gilt nur eingeschränkt. Boxerman, Weiskoff et al. [16, 180] zeigten für Gradientenecho-Sequenzen, dass bis zu Gefäßradien von ca. 10 µm die Abhängigkeit des Signalverlustes vom Suszeptibilitätsunterschied zunimmt, um für größere Radien in ein Plateau überzugehen. Für Spinecho-Sequenzen existiert ein Gefäßdurchmesser mit maximalem Einfluss der Suszeptibililtätsdifferenz $\Delta\chi$ auf das Signal. Dieser optimale Durchmesser liegt in Abhängigkeit von $\Delta\chi$ zwischen ca. 1,5 und 8 µm. Damit wird die Signalintensität beim Gradientenecho von Kapillaren (Radius < 8 µm) gleichermaßen wie von größeren Gefäßen beeinflusst, während sie beim Spinecho im wesentlichen vom Kapillarbett bestimmt wird. Insgesamt reagieren jedoch Gradientenecho-Sequenzen sensitiver (d.h. mit größeren relativen Signalverlusten) auf Suszeptibilitätsunterschiede als Spinecho-Sequenzen.

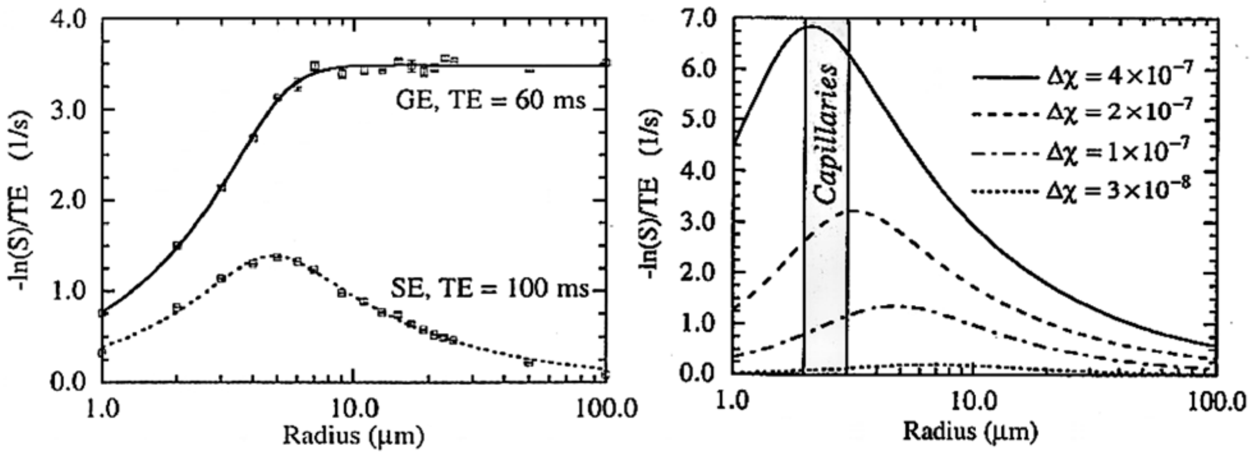

Abbildung 29: Monte-Carlo-Simulationen zur Abhängigkeit der beobachteten Veränderung der Relaxationsrate von Sequenz, Gefäßdurchmesser und Suszeptibililtätsdifferenz. Links: Abhängigkeit von ΔR_2^* vom Gefäßdurchmesser für Spinecho- und Gradientenecho-Sequenzen. Rechts: Abhängigkeit von ΔR_2^* von der Suszeptibilitätsdifferenz und dem Gefäßdurchmesser für eine Spinecho-Sequenz mit T_E = 100 ms (aus [16])

Für den Fall, dass für jeden Zeitpunkt der Konzentrations-Zeit-Kurve Messwerte aus T_1- und T_2^*-gewichteten Messungen zur Verfügung stehen (z. B. als Ergebnis der Messung mittels Doppelecho-Sequenz), lässt sich T_2^* punktweise bestimmen. Dies ist die Voraussetzung für die quantitative Auswertung der Kontrastmittelpassage in Regionen mit gleichzeitiger starker Verkürzung von T_1 durch Aufnahme von Kontrastmittel in den Interzellularraum [73, 78, 59, 76, 124]. Wenn wiederum die Signalintensität gemäß Gleichung (25) exponentiell von T_2^* abhängt, ist eine Separation des Einflusses der Verkürzung von T_1 bzw. T_2^* auf die Signalintensität möglich (vgl. Kapitel 5.1 weiter unten).

Die Zeitabhängigkeit der Kontrastmittelkonzentration nach Injektion in einem Gefäß kann mittels der auf Stewart [165] zurückgehenden Indikator-Verdünnungs-Theorie beschrieben werden [144, 171, 6] (siehe Kapitel 3.1.2).

2.2.4.5 Verbleib eines paramagnetischen Kontrastmittels im Gefäßbett

Für eine verbesserte Darstellung des Blutes und dessen Abgrenzung vom umgebenden Gewebe sind intravasal verbleibende Kontrastmittel von besonderem Interesse. Deren Einsatz bei "Bright-Blood-Sequenzen" wie FLASH führt bei geeigneter Wichtung über eine T_1-Verkürzung zu höherer Signalintensität des Blutes und damit zu besserem Kontrast/Rausch-Verhältnis. Kroft et al. [108] beschreiben in unterschiedlichen Gefäßen innerhalb des Abdomens von Schweinen Signalzuwächse von 200 bis über 700 %. Der Einsatz von Gadomer-17 (Schering) ermöglicht im Vergleich zu gefäßwandgängigen Kontrastmitteln ein um den Faktor 2 bis 4 besseres Signal/Rausch-Verhältnis [29].

Intravasal verbleibende Kontrastmittel vereinfachen ebenso die Darstellung der Perfusion in Geweben, bei denen KM „üblicher“ Molekülgröße wie z. B. Gadolinium-DTPA in die Zellzwischenräume eintreten und ebenso wie das intravasale KM zur Verkürzung der Relaxationszeiten beitragen.

2.2.5 Andere MR-tomographische Kontrastmittel

2.2.5.1 Klassifikation nach Verteilungsverhalten im Körper

Die folgende Klassifikation entspricht im wesentlichen der Darstellung in [113], ergänzt um den Aspekt der rezeptor-spezifischen Kontrastmittel.

Die höchste klinische Relevanz kommt der Klasse der extrazellulären Kontrastmittel zu, die sich nach intravenöser Injektion rasch im Blutvolumen verteilen und schnell in den Extrazellularraum diffundieren [179]. Hierbei handelt es sich vorwiegend um Gadolinium-Chelate, zu denen auch das in dieser Arbeit vorwiegend eingesetzte Gd-DTPA (zuerst unter dem Markennamen Magnevist® eingeführt) zählt.

Diesen Kontrastmitteln ähnlich verhalten sich die hepatobiliären KM, von denen ein unterschiedlich großer Anteil von den Leberzellen aufgenommen und langsam in die Galle ausgeschieden wird. Die Aufnahme in die Hepatozyten erfolgt hier auf Grund der Erkennung einer lipophilen Seitenkette durch die Leberzellmembran oder aber bei Mangan-basierten KM auch über Aufnahme freier Mn-Ionen in die Leberzellen. Metastasen leberfremder Tumoren stellen sich durch geringere Aufnahme eines Fe^{3+}-haltigen KM als das umgebende Lebergewebe dar.

Retikuloendotheliale Kontrastmittel bestehen aus dextran- bzw. carboxydextran-umhüllten Eisenoxidkristallen (SPIO – superparamagnetic iron oxide). Diese Partikel werden von den Kupffer-Sternzellen im retikulo-endothelialen System der Leber aufgenommen und führen durch die Veränderung des statischen Magnetfeldes in ihrer Umgebung zu einem T_2^*-bedingten Signalverlust.

Intravasale Kontrastmittel (blood pool contrast agent) basieren auf unterschiedlichen Prinzipien: Entweder wird Gd-DTPA (evtl. erst nach der Injektion) zeitweilig an Albumin gebunden, was durch die deutlich erhöhte Molekülgröße dessen Ausscheidung verzögert (vgl. Abschnitt 2.2.4.5). Aus Gründen der eingeschränkten Verträglichkeit von Albumin-Präparaten werden auch andere makromolekulare Gd-Verbindungen getestet (vgl. z. B. [106]) Alternativ werden SPIO mit im Vergleich zu den retikulo-endothelialen KM kleinerer Partikelgröße eingesetzt. Letztere verhalten sich in der Bildgebung ähnlich wie paramagnetische KM. Da sehr kleine SPIO von z. B. 17 ... 21 nm das Gefäß-Endothel durchwandern und von Makrophagen phagozytiert werden können, reichern

sie sich auch in gesunden Lymphknoten an (im Gegensatz zu Lymphknoten-Metastasen, in denen die lymphatischen Zellen verdrängt sind). Daher können kleine SPIO auch als lymphographische Kontrastmittel eingesetzt werden.

In der Gruppe der gastrointestinalen Kontrastmittel finden sich ebenfalls mehrere physikalisch-chemische Ansätze realisiert: Gadolinium-Chelate und andere Gd-Verbindungen, Mangansalze, SPIO. Darüber hinaus sind zumindest zur Abgrenzung des oberen Magen-Darm-Traktes erheblich preiswertere Substanzen wie Kaopromt (ein Durchfallmittel auf Kaolin- und Pektinbasis)), Blaubeersaft, Milch oder Wasser einsetzbar.

Gegenwärtig werden Kontrastmittel entwickelt, die spezifisch an ausgewählte Rezeptoren an Zelloberflächen oder an Zielsubstanzen (z. B. Fibrin [188]) binden [21]. Die spezifische Bindung an Rezeptoren wird in der Regel durch Konjugation eines paramagnetischen Kontrastmittels (beispielsweise Gd-DTPA [160]) oder paramagnetischer bzw. superparamagnetischer Nanopartikel [2, 134] mit einem monoklonalen Antikörper gewährleistet. Problematisch ist immer noch die relativ geringe Sensitivität der MR-Signalintensitäten auf Kontrastmittel. Im Vergleich zum Nachweis von Radiopharmaka sind um den Faktor 10^4 bis 10^6 höhere Konzentrationen erforderlich. Dem steht die im Vergleich zu optischen und nuklearmedizinischen Methoden deutlich höhere räumliche Auflösung gegenüber. Zur Sensitivitäts-Erhöhung existieren verschiedene biochemische bzw. zellphysiologische Ansätze. So kann die lokale KM-Konzentration im Vergleich zur Bindung an Rezeptoren erhöht werden, wenn das nachzuweisende Genprodukt zu einer intrazellulären Akkumulation von KM führt. Weiterhin kann die Umsetzung eines „Prä-Kontrastmittels“ in eine kontrasterzeugende Substanz durch das Genprodukt genutzt werden. Ähnlich kann ein KM (z. B. Gd) durch eine Umhüllung mit einem Substrat unwirksam gemacht werden. Es wirkt erst dann kontrasterzeugend, wenn das nachzuweisende Enzym das Substrat abbaut. Ein weiterer Ansatz besteht in der Konjugation von Antikörpern mit einer Peroxydase. Als Kontrastmittel wird hier ein an Phenol gebundenes Gadolinium-Chelat zusammen mit H_2O_2 gegeben. Im Ergebnis der Reaktion der Peroxydase mit H_2O_2 , in der das Phenol Elektronen abgibt, polymerisiert das Gd-Chelat, was einer lokalen Konzentrationserhöhung entspricht. Der Vorteil dieses Ansatzes besteht darin, dass die Verstärkungswirkung unabhängig vom konkreten Antikörper ist und damit zum Nachweis unterschiedlichster Substrate dienen kann. Der Fall, dass ein Genprodukt unmittelbar oder mittelbar selbst als Kontrastmittel wirkt, stellt schließlich die Verbindung zwischen diesem Abschnitt und Abschnitt 2.2.5.2 her. So wurde eine Tyrosinase-Expression über verstärkte Produktion von paramagnetischem Melanin nachgewiesen [86].

2.2.5.2 Körpereigene Substanzen als Kontrastmittel

Auch körpereigene paramagnetische Substanzen führen zu Relaxationszeit- und damit Signalveränderungen. Von klinischer Relevanz sind hier beispielsweise die vermehrte Einlagerung von Eisen in die Leber bei Hämochromatose (Abbildung 30) oder auch das in Melanom-Metastasen u. U. auftretende Melanin.

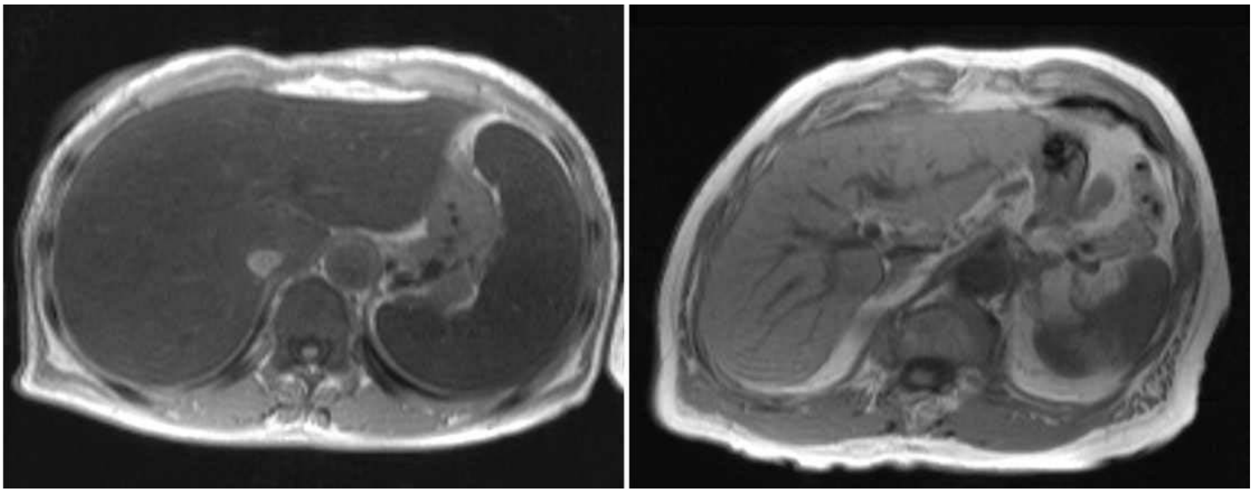

Abbildung 30: Signalabsenkung im T2-gewichteten Bild durch Eisen-Einlagerung in die Leber im Sinne eines körpereigenen Kontrastmittels. Links: Signalabgesenkte Leber und Milz bei Hämochromatose-Patientin, rechts: Leber mit normaler Signalintensität

Durch den Paramagnetismus von Desoxyhämoglobin führt eine höhere Sauerstoffbeladung der Erythrozyten im Blut (= niedrigere Konzentration von Desoxyhämoglobin) zu einer höheren Signalintensität in $T_2(^{*})$-gewichteten Bildern. Diese tritt z. B. bei aktivierten Hirnarealen auf, da der erhöhte Sauerstoffbedarf des Hirngewebes durch eine erhöhte Blutzufuhr überkompensiert wird. Hirnaktivität lässt sich dadurch prinzipiell als Signalzunahme in BOLD-fMRI-Sequenzen darstellen (Blood Oxygenetion Level Dependend functional Magnetic Resonance Imaging). Da die bei dieser Methode beobachtbaren Signalveränderungen klein sind, erfordert deren Nachweis in der Regel Mittelungen über eine Vielzahl von Messungen (ggflls. sogar über mehrere Patienten)

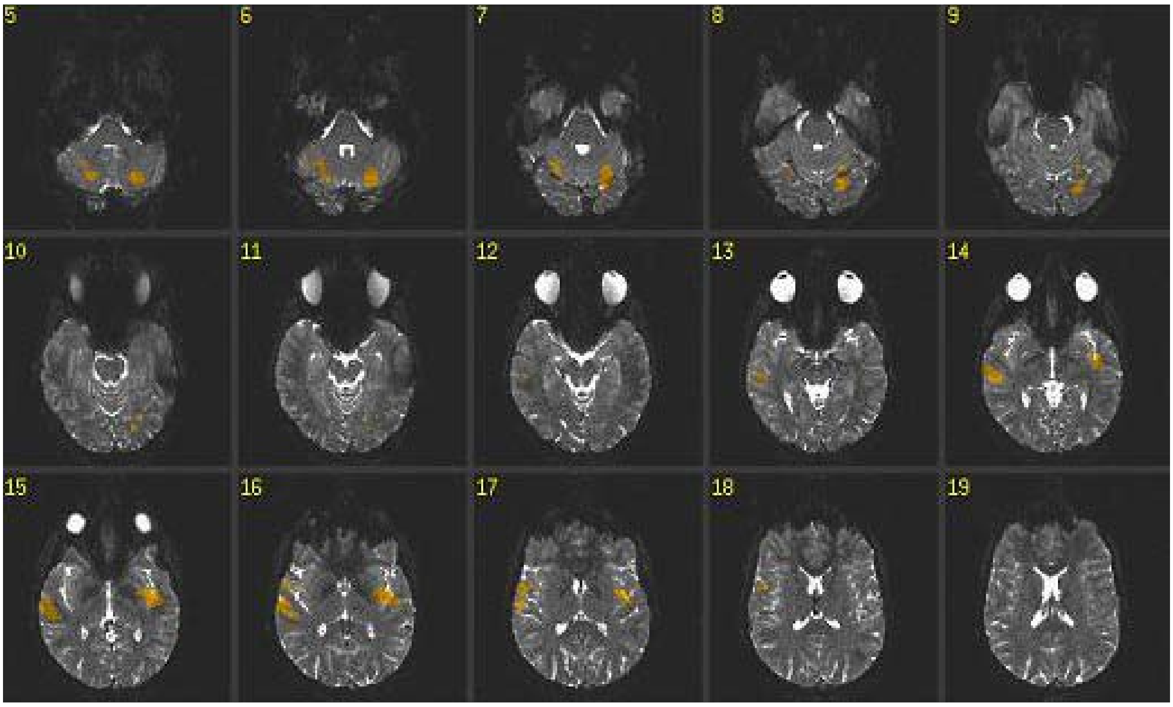

Abbildung 31: Hirnaktivierung bei Stimulation des Riechsinnes mittels Pfefferminz-Aroma. Die ein gewisses Signifikanzniveau übersteigenden mittleren Signaländerungen (z-scores) sind einer T_2-gewichteten Bildserie überlagert.

Ein weiterer Ansatz der Nutzung körpereigener Substanzen zur Erzeugung von Kontrasten ist die Darstellung der Gewebedurchblutung mittels Spin-Labeling. Hierbei wird einem in Bereich, der in Stromrichtung vor der zu untersuchenden Region liegt, eine Anregungsbedingung alterniert [96]. Beispielsweise kann ein Inversionspuls abwechselnd selektiv auf die darzustellende Region und unselektiv angewandt werden (ASSIST-Sequenz (attenuating the static signal in arterial spin tagging)) [187]. Spins, die auf diese Art markiert wurden, modifizieren das Signal der darzustellenden Region in dem Maße, in dem sie in das Gewebe einströmen. Mittels Subtraktion der Bilder, die unter diesen unterschiedlichen Anregungsbedingungen erzeugt wurden, lassen sich Perfusionsbilder erzeugen. Vorteilhaft an dieser Methode ist die komplette Nicht-Invasivität (es muss keine Substanz in den Körper eingebracht werden), nachteilig ist das (zumindest gegenwärtig noch) schlechtere Kontrast-Rausch-Verhältnis im Vergleich zu kontrastmittel-gebundenen Methoden der Perfusions-Bildgebung.

2.2.6 Andere physikalische Parameter zur Gewebecharakterisierung

2.2.6.1 Relaxometrie

Im Gegensatz zur Computertomographie, bei der die Pixelwerte den linearen Absorptionskoeffizienten (bei gegebenem Röntgenspektrum) repräsentieren, sind die Absolutwerte der Signalintensitäten bei Kernspintomographien massiv von den konkreten Messbedingungen abhängig. Die Relaxationszeiten T_1 und $T_2^{(*)}$ lassen sich jedoch über die Variation von Messbedingungen (insbesondere T_R und T_E) unter Benutzung von Gleichung (14) für Spinecho- bzw. Gleichung (15) für Gradientenecho-Sequenzen bestimmen. Prädestiniert für T_1-Messungen sind Inversion-Recovery-Sequenzen, auf Grund ihres initialen 180°-Pulses (Gleichung (17)).

Die Spin-Gitter-Relaxationszeit variiert mit der Magnetfeldstärke, da für die Energieübertragung zur Umgebung die Rotationsfrequenz des jeweiligen Moleküls gleich der Larmor-Frequenz sein muss [176]. Fischer et al. [37] formulierten ein empirisches Modell (Gleichung (27))

$$\frac{1}{T_1} = \frac{1}{T_{1,wasser}} + D + \frac{A}{1 + \left(\frac{\nu}{\nu_c}\right)^{\beta}} \qquad (\ 27)$$

mit ν als der Larmor-Frequenz, $T_{1,wasser}$ der Relaxationszeit von reinem Wasser sowie β, ν_c, A und D als freien Parametern (Tabelle 5), welches sich in guter Übereinstimmung mit den in vivo gemessenen experimentellen Daten von Henriksen et al. [64] befindet.

Gewebe	D [1/s]	A	ν_c [1/s]	β
Weiße Hirnsubstanz	-1,38	18,25	0,165	0,291
	-1,06	15,97	0,32	0,328
Graue Hirnsubstanz	0,105	11,66	0,059	0,42
	0,13	11,21	0,081	0,428
Skelettmuskel	0,026	25,7	0,161	0,617

Tabelle 5: Parameter für Gleichung (27) zur Abhängigkeit der Spin-Gitter-Relaxationszeit von der Larmor-Frequenz (Beziehung zwischen Larmor-Frequenz und Feldstärke gemäß Gleichung (2)), gewonnen an ex-vivo-Material. (nach [37])

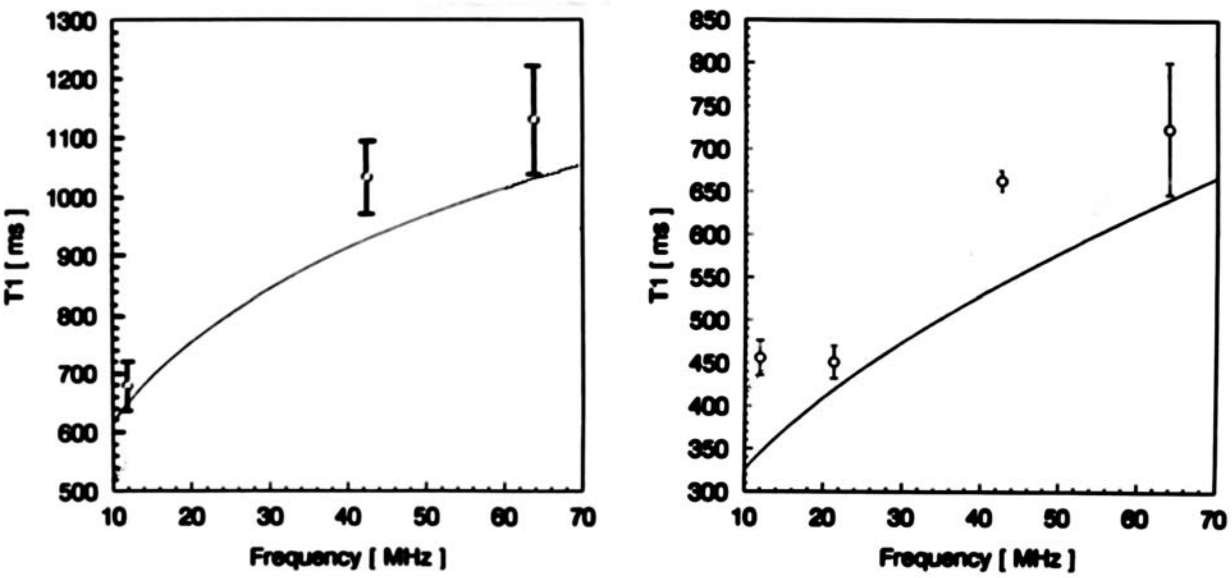

Abbildung 32: Abhängigkeit der T_1-Relaxationszeit von grauer (links) und weißer (rechts) Hirnsubstanz von der Larmor-Frequenz. Punkte: in-vivo-Messungen von Henriksen et al. [64], Kurven nach Fischer et al. [37]. (modifiziert aus [64])

Prinzipiell muss bei in-vivo-Messungen von Stoffgemischen mit unterschiedlichen Relaxationszeiten ausgegangen werden. Bei der Diskriminierung zwischen Ödem und Tumor im menschlichen Hirn mittels biexponentieller Modellierung wurden in [157] eine Genauigkeit von 94 % erzielt. In Abhängigkeit vom erzielten Signal-Rausch-Verhältnis ist es jedoch u. U. ausreichend anzunehmen, dass innerhalb einer Messregion (ROI oder Pixel) Material mit einheitlichen Relaxationszeiten erfasst wird Diese Annahme ist für homogene Gewebe experimentell gerechtfertigt. In [83] wurden in nur 10 % aller untersuchten normalen und pathologischen Strukturen im Rattenhirn signifikante Verletzungen dieser Hypothese ohne Systematik bezüglich der Gewebeart gefunden. Limitierend bei der Berechnung der Relaxationszeiten wirkt, dass der Fehler der

abgeleiteten Größe T_1 oder T_2 die Streuung der für deren Berechnung benutzten Signalintensitäten erheblich übersteigen kann [99], so dass die klinische Relevanz von Signalintensitäts-Veränderungen insbesondere bei gut reproduzierbaren Messbedingungen (z. B. bei Zeitreihen innerhalb einer Untersuchung am selben Patienten) größer als die der Relaxationszeiten sein kann (Tabelle 6) [43, 83].

Gewebe	T_1	T_2
Hirn (Mittelwert)	1057 ± 77	77 ± 6
Kortex	1076 ±102	75 ± 5
Corpus striatum	1020 ± 73	78 ± 8
Balken	946 ± 51	73 ± 8
Kleinhirn	1010 ± 91	nicht gemessen
Riechhirn	1298 ± 346	nicht gemessen
Tumor	1301 ± 167	91 ± 9
Ödem	1370 ± 172	109 ± 16
Hirnventrikel	1579 ± 383	172 ± 91

Tabelle 6: Relaxationszeiten ± Standardabweichung verschiedener Gewebe im Rattenhirn. Bestimmung in vitro bei 4,7 Tesla. Unter „Tumor“ sind die Zelllinien F98 Gliom, RN6 Schwanom und E367 Neuroblastom zusammengefasst, die sich relaxometrisch nicht unterscheiden ließen. (nach [83])

Eine epileptische Vorgeschichte ist offenbar mit Veränderungen im Hippocampus korreliert, die sich in einer Verlängerung der T_2-Zeit äußern (Tabelle 7, [90])

	Normal	Grenzwertig	Erhöht	Gesamt
Bedeutende Ereignisse				
Fiebriger Anfall	4	2	14	20
Fieberfreier Anfall			1	1
Bakterielle Meningitis			2	2
Mumpsenzephalitis	1			1
Gefäßentzündung			1	1
Geringfügige Ereignisse				
Zangengeburt	1			1
Vorgeburtliche Wachstumsverzögerung	1			1
Kopfverletzung	2	2		4
Geistige Unterentwicklung	1			1
Keine Vorgeschichte	59	3	9	71
Gesamt	69	7	27	103

Tabelle 7: In vivo bei 1,5 Tesla gemessene T_2-Relaxationszeiten in Abhängigkeit von der Vorgeschichte der Probanden/Patienten bezüglich Epilepsie (aus [90]).

Prinzipiell kann sich der u.a. durch vorangegangene Belastung beeinflusste physiologische Zustand in Veränderungen der Relaxationszeiten niederschlagen, die zumindest in aufeinanderfolgenden Messungen am selben Patienten innerhalb einer Sitzung als Signalveränderungen nachweisbar sind, wie vor und nach Pressübungen am Kaumuskel gezeigt werden konnte [43].

Zumindest im Mittel lassen sich Unterschiede in der T_2-Relaxationszeit auch zwischen depressiven (n = 20) und nicht-depressiven (n = 11) Parkinson-Patienten im Bereich des Mittelhirns und der Verbindung zwischen Groß- und Kleinhirn (Pons) nachweisen [11].

Huang et al. [88] fanden bei Patienten mit Duchenne-Muskeldystrophie deutliche Verschiebungen der T_1- und T_2^*-Relaxationszeiten in den betroffenen Muskeln, die sich durch den fettigen Umbau der Muskelsubstanz erklären dürften.

Aus der abnehmenden Häufigkeit von Veröffentlichungen zu relaxometrischen Untersuchungen in der Herzdiagnostik [141, 174] ist zu schließen, dass sich die Methode dort nicht etablieren konnte. Ein Grund dafür dürften die messtechnischen Schwierigkeiten sein, die durch den Herzschlag und die damit verbundenen Gewebe-Bewegungen, aber auch die durch Triggerung eingeführten Schwankungen der Repetitionszeit bedingt sind.

Generell erscheinen quantitative Bestimmungen der Relaxationszeiten wegen deren großer interindividueller Streuung im Vergleich zu pathologischen Veränderungen für diagnostische Zwecke eher weniger geeignet. Von größerer Bedeutung erscheinen die durch das veränderte Relaxationsverhalten beeinflussten Signalintensitäten, die durch

den direkten visuellen Vergleich mit umgebendem Gewebe am selben Patienten die „individuellen Normalwerte“ implizit berücksichtigen.

Schad et al. [156] zeigten bei der Klassifikation von Hirngewebe auf Basis von MR-Parametern, dass mittels der T_1- und T_2^*-Zeiten eine korrekte Unterscheidung grauer und weißer Substanz, Liquor und pathologischer Hirnläsionen gelingt. Weitergehende Unterscheidungen wie z. B. zwischen Tumor und Ödem waren unter Benutzung von Strukturparametern wie z. B. Histogrammanalyse von Grauwertgradienten, Kontrast, Homogenität, Entropie und Korrelation von Grauwerten sowie die Verteilung von deren Lauflängen möglich.

2.2.6.2 Diffusion

Analog zu Flusseffekten führt auch die Diffusionsbewegung von Wasserstoffkernen in einem Gradientenfeld zu einer Dephasierung, die durch Standard-Rephasierungsgradienten (Gradientenecho) bzw. RF-Impulse (Spinecho) nicht rephasiert werden kann. Durch gezieltes Zuschalten eines Gradienten während der Dephasierungs- bzw. Rephasierungsphase einer Sequenz kann diese in Richtung dieses Gradienten gezielt für Diffusionseffekte sensibilisiert werden (Stejskal-Tanner-Technik [114]). Über den Inhalt des physikalischen Diffusionsbegriffes hinaus werden hier auch ungerichtete Bewegungen der Wasserstoffkerne mit erfasst, solange sie über den Zeitraum von De- und Rephasierung hinweg zu einer (evtl. temporären) Ortsveränderung führen. Diese Bewegungen führen beispielsweise in Flüssigkeitsräumen auch ohne Konzentrationsgradienten zur Messung eines großen „scheinbaren Diffusionskoeffizienten“ (apparent diffusion coefficient ADC) D. Die Empfindlichkeit der Signalintensität für Diffusionseffekte lässt sich durch die Gradienten-Parameter einstellen (Gleichung (28):

$$\frac{M}{M_0} = \exp(-(\gamma * G * \delta)^2 * D * \frac{T_E}{2} \qquad (28)$$

mit $G * \delta$ als dem Integral der Gradientenfeldstärke über die Zeit.

Durch aufeinanderfolgende diffusionsgewichtete Bildgebung mit sechs verschiedenen Diffusionsgradienten-Kombinationen lässt sich prinzipiell der Diffusionstensor bestimmen. Indem zusammenhängende Bereiche mit ähnlicher, starker Anisotropie verfolgt werden, können Faserverläufe der weißen Hirnsubstanz segmentiert werden („Fibre tracking“ - Abbildung 33).

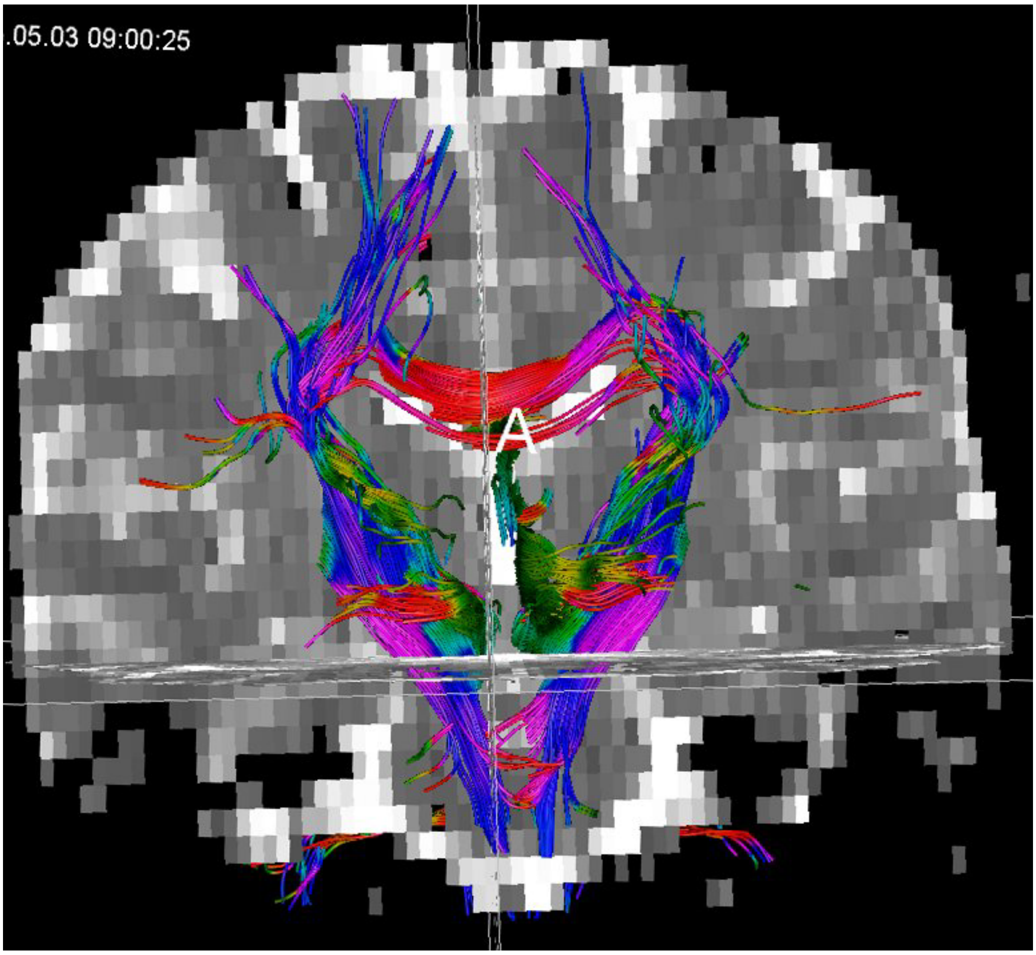

Abbildung 33: Darstellung von Nervenbahnen des Balkens (corpus callosum) und deren Fortsetzung mittels „Fibre tracking".

Da physiologische oder stochastische Bewegungen des darzustellenden Objektes (häufig menschlicher Schädel) ebenfalls zu Dephasierungen führen, werden In der klinischen Routine diffusionsgewichtete Bilder häufig mittels EPI-Sequenzen aufgenommen, um die Messzeit und damit die Wahrscheinlichkeit für das Auftreten solcher Bewegungen zu minimieren. In einer Präparationsphase kann analog zu konventionellen SE- oder FLASH-Sequenzen das Paar von Diffusions-Wichtungs-Gradienten einmal geschaltet werden; der gesamte k-Raum kann danach mit EPI als Auslese-Sequenz gefüllt werden.

Die klinische Bedeutung der diffusionsgewichteten Bildgebung besteht in der Früherkennung von Hirninfarkten und der Einschätzung von Therapiemöglichkeiten. Eine temporäre Minderdurchblutung von Gewebe, wie sie beispielsweise mittels T_2^*-gewichteter Perfusions-Bildgebung nachweisbar ist, ist nicht zwingend einer irreversiblen Schädigung gleichzusetzen. Gewebs-Untergang ist jedoch mit hoher Wahrscheinlichkeit mit einer Störung von Diffusionsbarrieren verbunden [97]. Diese stellen sich im diffusionsgewichteten MRT mit geringerem dephasierungsbedingten Signalverlust, also mit erhöhter Signalintensität dar. Der Verlust der strukturbedingten physiologischen Anisotropie des Diffusionstensors ist ein weiteres Indiz für irreversible Gewebeschädigung. Aus diesem Grund ist die Messung von diffusionsgewichteten Bildern nacheinander mit Diffusions-Wichtungs-Gradienten in drei Raumrichtungen klinisch etabliert (auch wenn damit beispielsweise prinzipiell nicht zwischen vollständig

isotroper Diffusion und vollständig gerichteter Diffusion in der durch die Diffusionsgradienten aufgespannten Raumdiagonalen unterschieden werden kann).

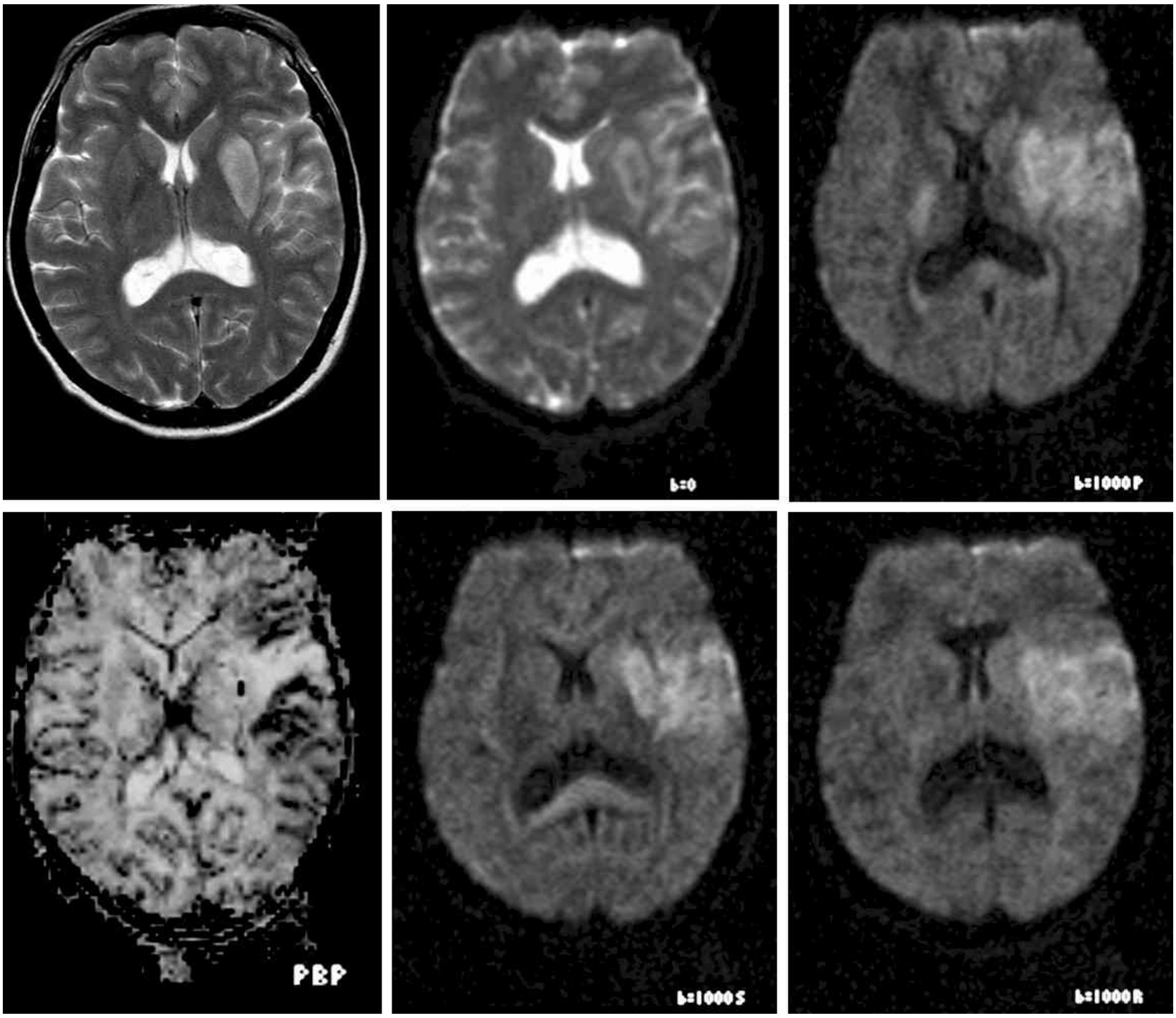

Abbildung 34: Hirninfarkt. Links oben: T_2-gewichtetes Turbo-Gradientenecho, das infarzierte Areal demarkiert sich rechts im Bild (anatomisch links) hyperintens. Mitte oben: T_2^*-gewichtetes EPI (gleiche Ortsauflösung wie Diffusions- und Perfusionsbilder). Links unten: Peripheres Blutvolumen, berechnet aus T_2^*-gewichteten Messungen der Passage eines Kontrastmittel-Bolus. Ein großer Bereich rechts im Bild ist durch Minderdurchblutung gekennzeichnet. Rechts oben, Mitte und rechts unten: Diffusionsrichtung in Phasencodier-, Schichtselektions- und Ausleserichtung. Der Bereich mit isotrop erhöhtem scheinbaren Diffusionskoeffizienten ADC umfasst nicht die gesamte Ausdehnung des minderdurchbluteten Bereiches.

2.2.6.3 Volumetrie

Der gute Weichteilkontrast macht die Kernspintomographie besonders geeignet für die Bestimmung von Volumina anatomischer oder pathologischer Regionen. Genauigkeits-begrenzende technische Faktoren sind hier in absteigender Wichtung ausreichender Kontrast zwischen zu vermessender Struktur und Umgebung, die Dicke bzw. der Abstand der gemessenen Schichten, die räumliche Auflösung innerhalb der Schicht sowie u. U. Inhomogenitäten des Hauptmagnetfeldes, die über die Verschiebung der

Larmor-Frequenz zu einer verzerrten geometrischen Darstellung bis hin zu Signalausfällen führen können. Von mindestens ebenso großer Bedeutung ist die Segmentation des zu vermessenden Areals. Diese wird durch den Partialvolumen-Effekt insbesondere bei dickeren Schichten, aber auch durch die inhomogene „Ausleuchtung“ (Abhängigkeit der Signalintensität vom Abstand zur Oberflächenspule) erschwert. Schwellwertbasierte Segmentationsalgorithmen, die in der Auswertung computertomographischer Bilder vielfach eingesetzt werden, setzen RF-Empfangsspulen mit hinreichend homogenem B_1-Feld voraus, wie sie z. B. als „Birdcage“-Spulen für Schädel- und Knie-Untersuchungen verfügbar sind. Weiterhin kann in gewissem Umfang die Signalintensität auf die Sende-/Empfangscharakteristik der Spule normiert werden. Schließlich können, allerdings u. U. unter Inkaufnahme eines stärkeren Rauschens, unter unterschiedlichen Messbedingungen gewonnene Bilder zu Parameterbildern kombiniert werden (z. B. T_2-, T_1-, ρ-Bilder oder Multispektralanalyse [42]), die dann mit Schwellwerten sinnvoll analysiert werden können.

Am Beispiel der SPECT demonstrierten Schmidt et al. aus den Gruppen von Franke und Freyer [158, 39] einen mehrstufigen Segmentationsalgorithmus. Hier wird zunächst schwellwertbasiert ein Objektkern definiert. Innerhalb eines diesen Objektkern umschließenden, manuell vorzugebenden Quaders werden mit Hilfe von Konturinformationen (z. B. Signalgradienten, Faltungen mit Kantendetektoren) Signaleigenschaften definiert, die als scharfe Eingangsgrößen für eine fuzzy-basierte Oberflächensegmentierung dienen.

Mittels Volumenbestimmung von Mandelkern und Hippocampus kann die Atrophie dieser Strukturen bei Temporallappen-Epilepsie in ca. 86 % der Fälle nachgewiesen werden, wobei in 83 % die für die chirurgische Intervention wichtige Seitenzuordnung durch Volumetrie allein möglich war [22].

Bei Patienten mit der Alzheimerschen Krankheit lässt sich mittels MR-Volumetrie eine hohe Korrelation zwischen Schwere der Erkrankung und den Volumina des Mandelkern-Hippocampus-Komplexes sowie beider Temporallappen nachweisen [136]. Die gleiche Arbeitsgruppe findet bei Patienten sowohl mit vaskulär bedingter Demenz als auch mit Mb. Alzheimer atrophische Veränderungen im Vergleich zu einer Kontrollgruppe [135]. Die Differenzierung zwischen beiden Demenzformen erscheint jedoch eingeschränkt – die lokalen und globalen Volumenänderungen sind offenbar kaum spezifisch (lediglich für das Kleinhirn lässt sich im Mittel ein ausgeprägterer Volumenverlust für die vaskuläre Demenz zeigen; die starke Überlappung erlaubt jedoch keine differentialdiagnostische Nutzung dieses Parameters).

Bei gegebener Tumorart ist die Prognose um so günstiger, je kleiner prätherapeutisch das Tumorvolumen ist, da die Anzahl der clonogenen Zellen mit dem Volumen wächst [149]. Gleichzeitig bestimmen jedoch weitere Faktoren wie die intrinsische Radiosensitivität der Zellpopulation, die Reoxygenierung des Tumors sowie Redistribution der Tumorzellen die durch nicht-chirurgische Therapie erzielbare Tumorkontrolle. Das Tumorvolumen, insbesondere aber auch die Volumenänderung beeinflussen u. U. den weiteren Therapieverlauf. Die Vermessung der Tumor-Flächen in MR-Schnittbildern bietet hierbei eine deutlich höhere Genauigkeit als die Volumenschätzung anhand der maximalen Tumor-Ausdehnung in drei Ebenen auf konventionellen Röntgenbildern – entsprechend höher ist die Aussagekraft der MR-tomographisch bestimmten Volumina [120, 95]. Unzweckmäßige Sequenzwahl

bezüglich Kontrast und/oder Schichtdicke kann diesen Gewinn jedoch maskieren [169]. Trotz der prognostischen Bedeutung des Tumorvolumens scheinen die Genauigkeits-Anforderungen an dessen Bestimmung nicht allzu scharf zu sein. In einer Arbeit von Dubben et al. [30] werden hierfür lediglich ± 50 % angegeben.

Die Definition der zu vermessenden Region erweist sich u. U. als medizinisches Problem. Beim Hirninfarkt lässt sich mittels Perfusions-Bildgebung (Kapitel 2.2.4.4) die durchblutungs-gestörte Region darstellen, wobei die Darstellung der mean transit time (MTT) von besonderem Wert zu sein scheint. Mittels Diffusionsbildgebung (Kapitel 2.2.6.2) wird eine Region von mit hoher Wahrscheinlichkeit irreversibel geschädigtem Gewebe dargestellt [62].

In-vitro-Analysen der Genauigkeit der Volumetrie mittels Kernspintomographie an Ausgusspräparaten von Schweineherzen zeigten einen mittleren Fehler von ca. 4 % ohne systematische Tendenz, wobei als Goldstandard eine gravimetrische Volumenbestimmung (Wasserverdrängung) genutzt wurde [140]. Genauigkeits-bestimmend wirkt (neben Kontrast und Abbildungsmaßstab) die Orientierung des zu vermessenden Objektes in Bezug auf die Raumrichtung mit der geringsten räumlichen Auflösung (in der Regel die Achse senkrecht zur Bildebene).

Einen interessanten diagnostischen Ansatz stellt die Kombination von Aussagen zu Volumen und Oberfläche dar. Beier [9, Kapitel 8] analysierte aus computertomographischen Daten die „Rauhigkeit" der Oberfläche intrapulmonaler Läsionen. Als Maß dafür diente ein „fraktaler Index" F (Gleichung (29)).

$$F(thres) = \frac{\sqrt[2]{S(thres)}}{\sqrt[3]{V(thres)}} \qquad (29)$$

Die Abweichung von F(thres) vom Wert $\sqrt[6]{9/16*\pi} \approx 1.1$ ist ein Maß für die Abweichung der untersuchten Läsion von der Kugelform. F(thresh) erwies sich im Gegensatz zu Volumen V oder Oberfläche S der Läsion als sehr stabil bezüglich des Segmentationsschwellwertes *thres*. Der fraktale Index erwies sich als sehr geeignet zur Differentialdiagnose zwischen Lungenmetastasen (eher glatte Oberfläche, also kleinerer fraktaler Index) von infiltrativ wachsenden Prozessen. In der Arbeit von Beier gab es keine Überlappung zwischen beiden Gruppen; als Unterscheidungskriterium konnte dort F = 2,7 dienen.

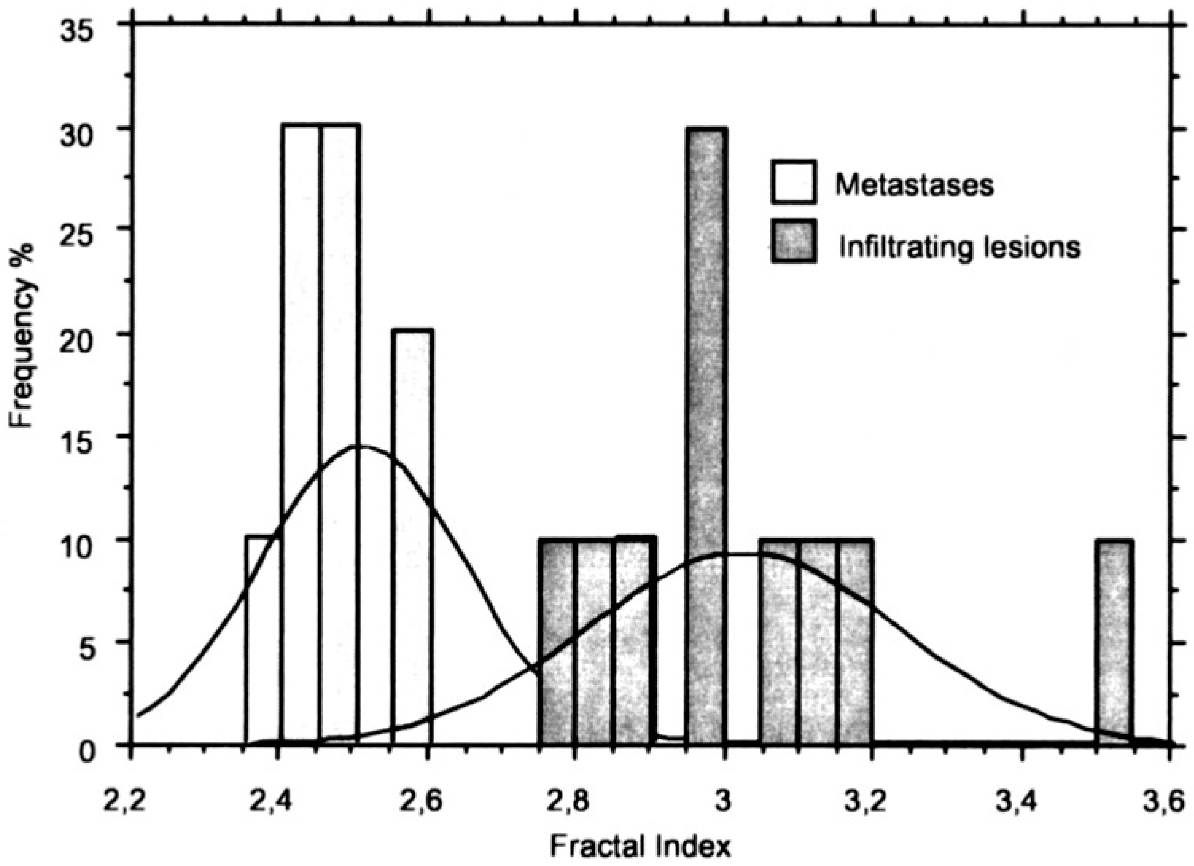

Abbildung 35: Häufigkeitsverteilungen des fraktalen Indexes (Gleichung (29)) für die Kollektive „Metastasen“ und „infiltrative Prozesse“ von intrapulmonalen Raumforderungen (aus [9]).

2.3 Gerätetechnische Präzisierung der Aufgabenstellung

Die bildgebende Diagnostik als Teil der gerätegestützten medizinischen Diagnostik lässt sich vereinfacht als Signalverarbeitungs-Prozess gemäß Abbildung 36 darstellen. Ein physiologischer Prozess manifestiert sich in der Veränderung mindestens einer, im Allgemeinen jedoch einer Vielzahl von physikalischen Größen. Mindestens eine dieser Größen kann nun als Signal interpretiert und damit einer gerätetechnischen Erfassung zugeführt werden. Hierunter soll im Weiteren der gesamte Prozess von der Umsetzung der physiologisch modulierten Größe(n) in Messwerte über Prozesse der Signal-Aufbereitung (z. B. Transformationen, Filterungen usw.) bis hin zur Schnittstelle zum Anwender (z. B. Visualisierung, Parametrisierung, ...) verstanden werden. Schließlich müssen die so aufbereiteten Informationen durch den Anwender (Arzt) im Kontext weiterer klinischer Informationen interpretiert und für den Patienten relevante Schlussfolgerungen gezogen werden.

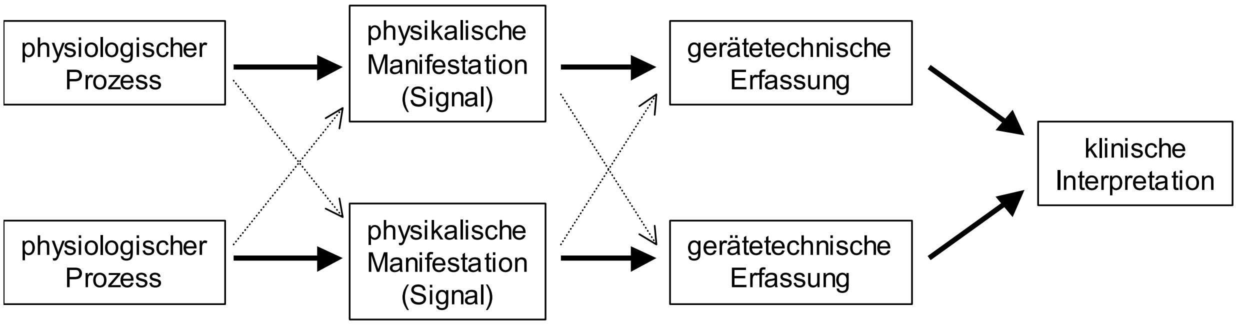

Abbildung 36: Prozess der gerätegestützten medizinischen Diagnostik

Im Allgemeinen wird dieser Prozess dadurch verkompliziert, dass ein physiologischer Prozess mehrere physikalische Manifestationen aufweist, die ihrerseits von mehreren physiologischen Prozessen beeinflusst werden. Weiterhin wird die Messung einer bestimmten physikalischen Manifestation, also eines Signals, unter Umständen von weiteren Signalen überlagert.

Hieraus resultieren mehrere Teilaufgaben für die gerätetechnische Erfassung physiologischer Prozesse:

1. Auswahl von für das klinische Problem relevanten physikalischen Größen
2. eventuell Manipulation der physikalischen Manifestation physiologischer Prozesse
3. Umsetzung ausgewählter physikalischer Größen und/oder von deren räumlicher und/oder zeitlicher Veränderung in Messwerte
4. Separation der Einflüsse der physikalischen Größen auf die Messwerte
5. Separation der Einflüsse der physiologischen Prozesse auf die physikalischen Größen
6. Aufbereitung der Messgrößen so, dass daraus auf die zu untersuchenden physiologischen Prozesse und/oder deren Veränderung geschlossen werden kann

Aus den klinischen Anforderungen an die über physiologische Prozesse bereitzustellende Informationen leiten sich Forderungen an die der klinischen Interpretation vorgelagerten, gerätetechnisch zu realisierenden Schritte ab. Diese umfassen neben der Auswahl der zu messenden physikalischen Größe(n) insbesondere Anforderungen an Genauigkeit und Reproduzierbarkeit sowie an das räumliche und/oder zeitliche Auflösungsvermögen. Weiterhin können Forderungen an das Ausmaß der geräteseitig zu realisierenden Umsetzung physikalischer in physiologische Größen resultieren.

Im Fall der kernspintomographischen Gewebe-Charakterisierung mittels Kontrastmittel-Dynamik entsprechen die oben genannten Teilaufgaben folgenden Vorgängen:

1. Wegen Anforderungen an räumliche Auflösung Entscheidung für eine bildgebende Methode. Ein Vorteil der Kernspintomographie im Vergleich zur Röntgen-Computertomographie oder zu nuklearmedizinischen Methoden ist das Fehlen einer Exposition des Patienten mit ionisierender Strahlung (sie steht Limitationen z. B. durch bewegliche Metallteile im Patienten (Clips,

Granatsplitter) oder Störung lebenserhaltender Systeme (Herzschrittmacher), aber auch Verfügbarkeits- und Kosten-Aspekte entgegen).

2. Markierung eines Teils des Blutes (z. B. Gabe von Kontrastmittel oder Spin-Tagging).

3. räumlich und zeitlich aufgelöste Messung einer von der Methode der Markierung abhängigen Größe (von Spin-Relaxation abhängige(s) Signal(e)).

4. Berechnung der markierungs-bedingten Veränderung des Signals bzw. der Signale (z. B. Berechnung von Relaxationszeiten oder Signal-Differenzen mit/ohne Tagging)

5. ggf. Separation der Kontrastmittel-Konzentrationen in unterschiedlichen Kompartimenten (Blutgefäße, Interstitium)

6. Berechnung von physiologisch interpretierbaren oder zumindest von zwischen Patienten vergleichbaren Parametern (z. B. relative Änderung der Relaxationsrate, relatives Blutvolumen, mittlere Passagezeit, Permeabilität der Blutgefäßwände ...)

In der Praxis der Methoden-Entwicklung beeinflussen klinische Anforderungen und gerätetechnische Realisierung einander wechselseitig: Ein gerätetechnisch erschlossener Effekt wird in klinischen Studien auf seine diagnostische Relevanz geprüft. Hieraus leiten sich Anforderungen an Messtechnik und Signalverarbeitung ab, nach deren Realisierung sich unter Umständen neue klinische Anwendungen mit wiederum veränderten gerätetechnischen Anforderungen eröffnen. Im Fall der medizinischen Nutzung der magnetischen Kernresonanz ging aus einer spektroskopischen Methode ein Verfahren der Bilderzeugung hervor, welches seinerseits verschiedene quantitative Verfahren wie z. B. Messungen von Blutflüssen und –volumina, (scheinbaren) Diffusionskoeffizienten, Gefäßwand-Permeabilitäten usw., aber auch wiederum räumlich aufgelöste spektroskopische Methoden hervorbrachte. Eine klinisch sinnvolle Anwendung der Kontrastmitteldynamik ist sowohl an geräteseitige Voraussetzungen wie die Erreichbarkeit einer hinreichenden zeitlichen Auflösung bei Erfassung eines ausreichend großen Volumens mit angemessener räumlicher Auflösung gebunden als auch an die Verfügbarkeit von Kontrastmittel, welches bei akzeptabler Verträglichkeit den Messeffekt beeinflussende physikalische Größen hinreichend stark und bezüglich physiologischer Prozesse ausreichend spezifisch beeinflusst. Beim gegenwärtigen Stand der Gerätetechnik (einschließlich der Pharmakologie von MR-Kontrastmitteln) bietet sich dafür der Einsatz von Gadolinium-Chelaten bei hinreichend hoher Magnetfeldstärke an.

Für folgende Teilprobleme der kontrastmittelgestützten Gewebscharakterisierung mittels Kernspintomographie sollen im Rahmen der vorliegenden Arbeit Lösungsansätze entwickelt werden:

1. Kernspintomographische Perfusionsmessungen basieren auf der Beeinflussung der T_2*-Relaxationszeit durch paramagnetische Kontrastmittel. In deren Umgebung wird gleichzeitig T_1 verkürzt. Die Verringerung von T_1 und T_2^* wirken konkurrierend auf die Signalintensität. Der Einfluss der T_2^*-Zeit kann hierbei selektiv durch die Wahl der Echozeit T_E beeinflusst werden. Zwar überwiegt in einem Netzwerk kontrastmittel-undurchlässiger Kapillaren die durch Suszeptibilitätsunterschiede zwischen KM-haltigem Blut und umgebendem Gewebe verursachte T_2^*-Verkürzung erheblich die

T_1-Verkürzung innerhalb der Kapillaren. Bei Kontrastmittel aufnehmenden Geweben (z. B. verschiedenen Tumoren und entzündlichen Prozessen) ist die T_1-Verkürzung durch in den interzellulären Raum übertretendes KM jedoch nicht mehr vernachlässigbar. Es war eine Methode zu entwickeln, mit der die Veränderung von T_2^* in kontrastmitteldynamischen Untersuchungen unabhängig von einer etwaigen T_1-Veränderung bestimmt werden kann (Abschnitt 5.1).

2. Das bei Kontrastmittel aufnehmenden Läsionen in das Interstitium übertretende KM führt nicht nur zu einer T_1-Verkürzung (die unter Punkt 1 zu behandeln war), sondern trägt durch seine räumlich inhomogene Verteilung auch zur Verkürzung von T_2^* bei. Eine Berechnung der T_1-Veränderung ist zum Beispiel möglich, wenn Messungen bei zwei unterschiedlichen Repetitionszeiten (Spinecho- oder Gradientenecho-Sequenzen) oder Delay-Zeiten (Inversion-Recovery- oder Saturation-Recovery-Sequenzen) vorliegen. In der klinischen Routine unterbleiben solche zusätzlichen Messungen in der Regel aus Zeitgründen. Es waren daher für die Fälle des Vorliegens und des Fehlens von für die T_1-Bestimmung erforderlichen Zusatzmessungen jeweils Algorithmen zu entwickeln, die die suszeptibilitäts-inhomogenitäts-bedingte T_2^*-Verkürzung vom Anteil des interstitiellen KM bereinigen (Abschnitt 5.2).

3. Menge und Geschwindigkeit des Übertritts von paramagnetischem Kontrastmittel in Gewebe werden von verschiedenen (patho-)physiologischen Parametern wie z. B. Gefäßdichte und Gefäßwand-Permeabilität beeinflusst und enthalten daher diagnostisch relevante Information. Unter verschiedenen messtechnischen Bedingungen, insbesondere bezüglich Magnetfeldstärke und Gradienten-Parametern, war der Zeitverlauf des durch die KM-bedingte T_1-Verkürzung verursachten Signalzuwachses nach Injektion eines Kontrastmittel-Bolus zu modellieren und aus dem Modell diagnoserelevante Parameter abzuleiten. Die Methode war insbesondere in der kernspintomographischen Mamma-Diagnostik zu etablieren. Für die Einstellung eines sinnvollen Kompromisses zwischen räumlicher und zeitlicher Auflösung der kontrastmitteldynamischen Untersuchung war retrospektiv die geringste Zeitauflösung ohne klinisch relevanten Informationsverlust zu bestimmen. Mit dem Ziel, nach Installation neuer MR-Scanner-Hardware in möglichst kurzer Zeit wieder klinisch relevante Informationen liefern zu können, war das bei 0,5 Tesla akquirierte statistische Wissen auf die 1,5 Tesla veränderte Messbedingungen zu übertragen (Abschnitt 5.3).

4. Sowohl aus der Verkürzung von T_1 als auch von T_2^* abgeleitete Parameter tragen – in Abhängigkeit von der differentialdiagnostischen Fragestellung – mehr oder weniger diagnostisch relevante Information. Am Beispiel von Tumoren im Schädelbereich war zu untersuchen, inwieweit eine gleichzeitige Messung der Veränderungen von T_1 und T_2^* klinisch vorteilhaft ist. Dies schloss die Prüfung der unter 1. zu entwickelnden Methode ein (Abschnitt 5.4).

5. Am Beispiel einer Studie an pharyngealen Tumoren, die neben MR-tomographischen Untersuchungen auch Positronen-Emissions-Tomographie sowie eine invasive Bestimmung des intratumoralen Sauerstoff-Partialdrucks umfasste, war die Beziehung verschiedener MR-Parameter zu MR-unabhängigen tumorphysiologischen Parametern zu evaluieren. Hierbei waren u.a. die in den Punkten 1, 2 und 3 erwähnten Auswertestrategien sowie deren Kombinationen zu berücksichtigen (Abschnitt 5.5).

6. Neben der in Kontrastmittel aufnehmenden Läsionen auftretenden Verkürzungen von T_1 und T_2^* durch interstitiell eingelagertes KM stellt der Zeitverlauf der Kontrastmittel-Konzentration im in das zu untersuchende Gewebe eintretenden Blut (AIF) eine Störgröße bei der quantitativen Perfusionsanalyse dar. Nach Implementierung eines entsprechenden Korrekturalgorithmus war zu prüfen, unter welchen Bedingungen die Berücksichtigung dieser Einflüsse zu einer Verbesserung (wegen erhöhter Richtigkeit) oder Verschlechterung (wegen Einbeziehung von mehr fehlerbehafteten Größen) der Ergebnisse von Bildgebung und quantitativer Analyse führt (Abschnitte 5.5.2 und 6.3).

7. Ausgehend vom Wertebereich der bei kontrastmittel-gestützten MR-Darstellungen von Blutgefäßen auftretenden T_1-Relaxationszeiten war der Verlauf der Spin-Gitter-Relaxation während einer Messsequenz zur Darstellung von Koronararterien bezüglich des Kontrast-Rausch-Verhältnisses zwischen Blutgefäßen und deren Umgebung zu optimieren (Abschnitt 5.6).

3 Messung kontrastmitteldynamischer Parameter

3.1 Modellierung der Signalverläufe

3.1.1 Kontrastmittelaufnahme

Unter den vereinfachenden Annahmen eines Drei-Kompartment-Modells (Abbildung 26 unter Vernachlässigung des Extrazellular-Raumes des gesamten Körpers) und einer linearen Abhängigkeit zwischen relativem Signalzuwachs und KM-Konzentration (vgl. Abschnitt 2.2.4) für die Infusion von KM geben Brix et al. [17] Gleichung (30) an

$$\frac{S}{S_0} = 1 + A * \{v * [\exp(k_{el} * t') - 1] * \exp(-k_{el} * t) - u * [\exp(k_{21} * t') - 1] * \exp(-k_{21} * t)\} \qquad (30)$$

mit A einer von Gewebe, Sequenz und Infusionsrate abhängigen Konstante, k_{el} der (im wesentlichen renalen) Eliminationsrate des KM aus dem Blut-Pool, k_{21} der Austauschrate zwischen Blut-Pool und Läsion sowie u und v Kombinationen aus diesen Austauschraten ($u = [k_{21}*(k_{21}-k_{el})]^{-1}$; $v = [k_{el}*(k_{21}-k_{el}])^{-1}$). t' ist während der Infusionsdauer τ identisch mit t (t' = t), danach gilt t = τ).[6]

Unter Zugrundelegung eines ähnlichen Ansatzes wie bei Brix et al. [17] analysieren Daldrup et al. [24] die Auswaschphase des KM über eine Stunde hinweg mit einer Zeitauflösung von 2 Minuten. Wenn man in einem Zwei-Kompartment-Modell (Blutplasma und interstitielles Wasser) den Transfer des KM vom Plasma zum Interstitium mit der Austauschkonstanten K^{PS} (entspricht in etwa dem Parameter k_{21} bei Brix et al. [17]) und den Transfer in umgekehrter Richtung mit k beschreibt, lässt sich die KM-Konzentration im interstitiellen Wasser C_I in Abhängigkeit von der Konzentration im Blut(plasma) C_p darstellen (Gleichung (31)):

$$\frac{dC_I(t)}{dt} = K^{PS} * C_P(t) - k * C_I(t) \qquad (31)$$

Als Lösung dieser Differentialgleichung ergibt sich für C_I im wesentlichen eine Summation der bis zum Zeitpunkt Θ in den benachbarten Gefäßen aufgetretenen Konzentrationen C_p, die mit dem Reflux vom Gewebe zum Blut gefaltet ist (Gleichung (32)):

$$C_I(t) = K^{PS} * \int_0^t C_P(\Theta) * \exp(-k * (t - \Theta)) d\Theta \qquad (32)$$

Im Rahmen der von Daldrup benutzten Zeitauflösung ist es gerechtfertigt, C_P durch die venöse KM-Konzentration, dividiert durch (1-Hct) zu ersetzen (Korrektur mit dem Hämatokrit des venösen Blutes).

---

[6] In der vorliegenden Arbeit wurde dieser Ansatz weiterentwickelt (vgl. Abschnitt 5.3.2).

Demsar et al. [26] gehen bei der Beschreibung der Aufnahme eines Blood-pool-Kontrastmittels (Albumin-$(Gd\text{-}DTPA)_{30}$, vgl. Abschnitt 2.2.5.1) davon aus, dass während der Dauer der dynamischen Studie (ca. 60 Minuten) eine Rückdiffusion von Kontrastmittel aus dem Interstitium in das Gefäßbett vernachlässigt werden kann. Unter dieser Annahme ist die interstitielle KM-Konzentration $C_{T\text{-}E}$ proportional zum Integral über die bis zum Zeitpunkt t durch KM in den Kapillaren des Gewebes bedingte Konzentration $C_{T\text{-}B}$: (Gleichung (33)):

$$C_{T-E} = FLR * \int_0^t C_{T-B}(t')dt' \tag{33}$$

Der Proportionalitätsfaktor FLR (fractional leak rate) beschreibt die Durchlässigkeit der Gefäßwände für das eingesetzte KM. Die im Gewebe MR-tomographisch zugängliche KM-Konzentration C_T ist die mit den entsprechenden Volumenanteilen gewichtete Summe der intra- und extravasalen Konzentrationen (BV steht für den relativen Anteil der Gefäßlumina am Gewebsvolumen; wegen BV << 1 wird der Faktor (1 – BV) für den Anteil des „gefäßfreien“ Gewebes am Gesamtvolumen gleich 1 gesetzt; weiterhin lasse sich in hinreichender Näherung der gefäßbedingte Anteil an der KM-Konzentration im Gewebe $C_{T\text{-}B}$ durch die Konzentration in einem langsam durchströmten Blutgefäß (hier der vena cava inferior) als $C_{T\text{-}B}(t) = BV * C_B(t)$ darstellen) (Gleichung (34)):

$$C_T(t) = BV * C_B(t) + FLR * \int_0^t BV * C_B(t')dt' \tag{34}$$

Die von Demsar et al. für Albumin-$(Gd\text{-}DTPA)_{30}$ in Ratten bestimmte sehr kleine Zeitkonstante $\beta = 0.0052\ min^{-1}$ für die monoexponentiell beschreibbare Ausscheidung rechtfertigt im Rahmen der Gesamtgenauigkeit der Untersuchung die Näherung gemäß Gleichung (35):

$$\frac{\int_0^t C_B(t')dt'}{C_B(t)} \approx \frac{1-\exp(-\beta * t)}{\beta * \exp(-\beta * t)} \approx t \tag{35}$$

Damit ergibt sich schließlich nach Division beider Seiten von Gleichung (34) durch $C_B(t)$ eine lineare Abhängigkeit des Konzentrationsverhältnisses zwischen Gewebe und Blutgefäß von der Zeit mit dem relativen Blutvolumen BV als Schnittpunkt mit der Ordinate sowie einem zum Permeabilitäts-Flächen-Produkt des Gewebes PS proportionalen Anstieg (Gleichung (36), Abbildung 37, Abbildung 38)

$$\frac{C_T(T)}{C_B(t)} = BV + \frac{PS}{1-Hct} * t \tag{36}$$

(Htc steht für den Hämatokrit, der in der zitierten Studie zwischen 38 % und 47 % liegt).

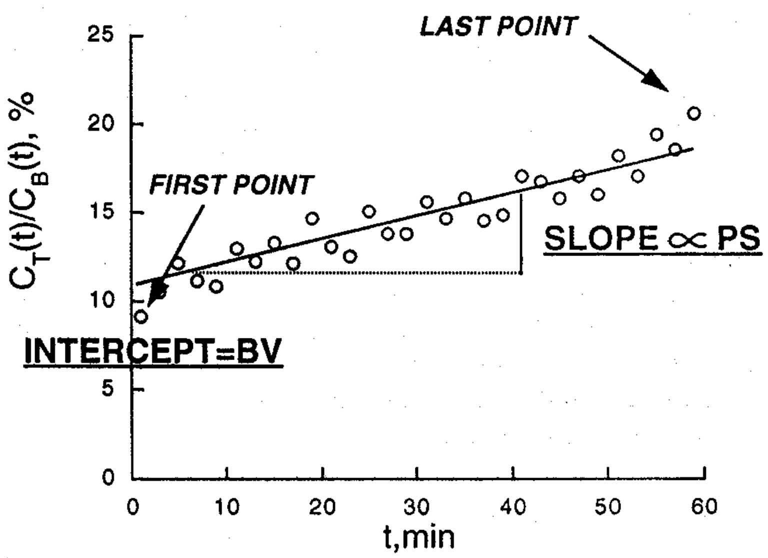

Abbildung 37: Abhängigkeit des Verhältnisses zwischen der Konzentration von Albumin-(Gd-DTPA)$_{30}$ in einem in eine Fischer-Ratte implantierten Adenokarzinom $C_T(t)$ und in der Hohlvene $C_B(t)$ von der Zeit. Ableitung des relativen Blutvolumens BV und des „permeability surface area product“ PS (aus [26])

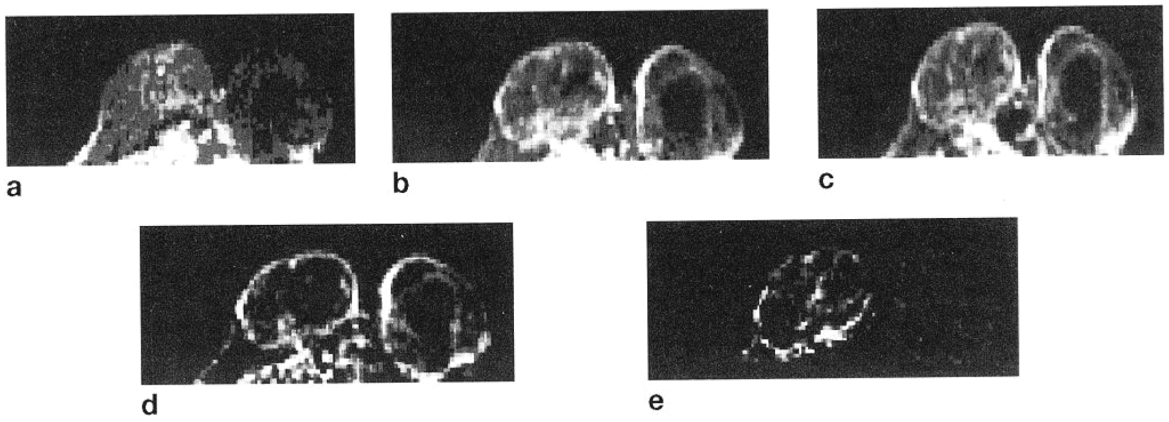

Abbildung 38: Transversale MR-Bilder eines Rattentumors gemäß Abbildung 37 (a) vor KM-Gabe, (b) eine bzw. (c) 30 min nach KM-Gabe, Parameterbilder des relativen Blutvolumens (d) bzw. des permeability surface area product (e) (aus [26])

Gleichung (34) stellt nach Division beider Seiten durch $C_B(t)$ die Grundlage für den Patlak-Plot dar [138, 150]. In der Formulierung gemäß Gleichung (37)

$$\frac{C_T(t)}{C_B(t)} = BV + K * \frac{\int_0^t C_B(t')dt'}{C_B(t)} \qquad (37)$$

mit K = FLR * BV lassen sich BV und K (und damit wiederum FLR) auch unter Verzicht auf die Näherung (35) aus den Parametern der Geradengleichung bestimmen (Abbildung 39):

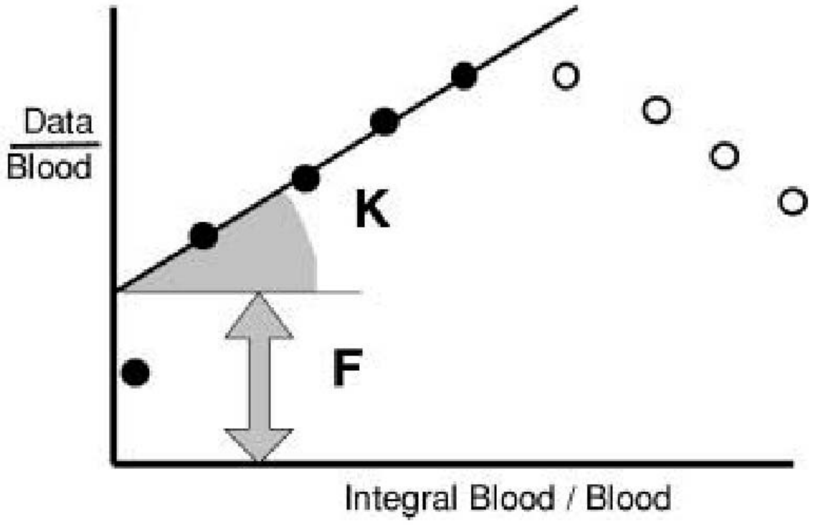

Abbildung 39: Rutland-Patlak-Plot als verallgemeinertes Werkzeug zur Bestimmung von Parametern des Übertritts eines Tracers aus dem Blut in Gewebe (aus [150])

Eine Abweichung der Punkte von einer Geraden wird hier als Beginn der Exkretion des Tracers interpretiert.

Der Patlak-Plot ist ebenso zur Beschreibung der Tracer-Kinetik z. B. in der Nierenfunktionsdiagnostik mittels Computertomographie anwendbar (vgl. z. B. Hackstein et al. [53])

3.1.2 Indikator-Verdünnungs-Theorie

Grundannahme der Indikator-Verdünnungs-Theorie (Indicator dilution theory – IDT) [6] ist der Verbleib des Indikators (hier: Kontrastmittels) in dem betrachteten Kompartiment (hier: Blutvolumen im Gefäßbett).

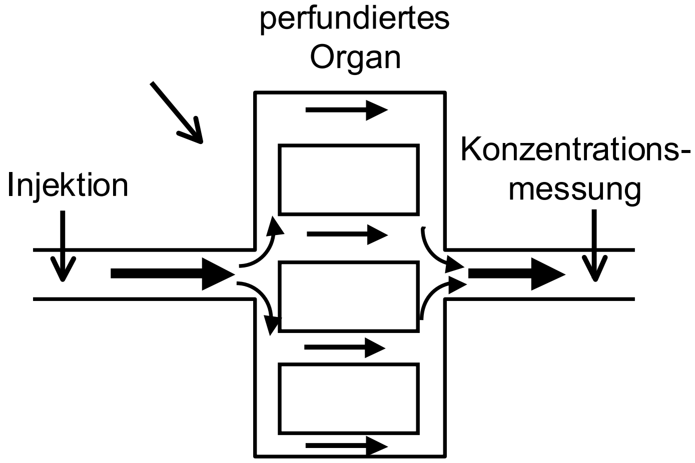

Abbildung 40: Ein-Kompartment-Modell der Indikator-Verdünnungs-Theorie (nach [6])

Weitere Voraussetzungen[7] sind:

1. stabiler und durch den Tracer nicht beeinflusster Fluss
2. vollständige Vermischung des Tracers mit der Trägerflüssigkeit
3. Messbarkeit der Tracer-Konzentration
4. Vernachlässigbarkeit von Rezirkulation (mehrfaches Passieren desselben Gefäßabschnittes durch den Tracer

Aus der postulierten Erhaltung der Gesamt-Kontrastmittel-Menge folgt Gleichung (38)

$$m = \int_0^\infty F * C(t)\, dt \qquad (38)$$

mit m als der Tracer-Gesamtmenge (z. B. in mg), F dem Fluss (z. B. in ml/s) und C der zeitabhängigen Tracer-Konzentration (z. B. in mg/ml). Mit der daraus hervorgehenden *Stewart-Hamilton-Gleichung* (39) lässt sich bei bekannter Indikator-Menge und Konzentrations-Zeit-Kurve der Fluss angeben:

$$F = \frac{m}{\int_0^\infty C(t)\, dt} \qquad (39)$$

Das Erste Moment der Konzentrations-Zeit-Kurve (das „mit der KM-Konzentration gewichtete Mittel über alle Zeitpunkte") wird als Mean Transit Time (MTT) bezeichnet (Gleichung (40))

$$MTT = \frac{\int_0^\infty t * C(t)\, dt}{\int_0^\infty C(t)\, dt} \qquad (40)$$

---

[7] Jede dieser Voraussetzungen ist bei Perfusionsmessungen mittels Gd-DTPA-basiertem Kontrastmittel nur bedingt erfüllt: Je nach Molekülgröße verlässt ein mehr oder weniger großer Anteil das Gefäßbett (bei Gd-DTPA um 45 % während der ersten Passage). Der arterielle Fluss ist pulsatil – die Betrachtung eines mittleren Flusses ist jedoch ausreichend. Bedingt durch die zellulären Blutbestandteile ist eine vollständige Vermischung nicht möglich – dies kann durch die Berücksichtigung eines unterschiedlichen Hämatokrits für Kapillaren und große Gefäße behandelt werden (Verhältnis ≤ 0.86 [94]). Über die Veränderung von ΔR_2^* ist lediglich ein relatives Maß für die Kontrastmittel-Konzentration verfügbar. Dessen Normierung über eine Arterielle Inputfunktion wird weiter unten diskutiert. Wegen des Übertritts von Kontrastmittel in das Gewebe ist diese Normierung nur in zum auszuwertenden Gewebe nicht zu weit entfernten Gefäßen sinnvoll. Die tatsächlich in beträchtlichem Maße auftretende Rezirkulation muss z. B. über Kurvenanpassung oder geschickte Wahl von Integrationsgrenzen berücksichtigt werden

Gleichung (38) gilt für jeden der Gefäßzweige in Abbildung 40 bezüglich der in ihn einströmenden Teilmenge des Indikators. Da aus Kontinuitätsgründen unmittelbar an der Verzweigungsstelle die arterielle Konzentrationskurve $C_{art}(t)$ jedem der Zweige aufgeprägt wird, der Fluss innerhalb eines nicht weiter verzweigten Gefäßabschnittes als konstant angenommen wird sowie vom Verbleib des Tracers im Gefäßbett ausgegangen wird, folgt für jeden (zu 100 % mit der Trägerflüssigkeit gefüllten) Gefäßzweig Gleichung (41):

$$\int_0^\infty C(t)\,dt = const \qquad (41)$$

Damit ist das Verhältnis von Trägerflüssigkeit zu sonstigem (tracer-freiem) Gewebe in einem Volumenelement – also im Fall von Perfusionsmessungen das relative Blutvolumen rBV – gegeben durch das Verhältnis der Integrale der Konzentrationskurven in diesem zum entsprechenden Integral in einer zuführenden Arterie (Gleichung (42))

$$rBV = \frac{\int_0^\infty C(t)\,dt}{\int_0^\infty C_{art}(t)\,dt} \qquad (42)$$

Der relative Fluss rBF durch das Volumenelement ist dann gegeben durch das Volumen der Trägerflüssigkeit rBV, das in der Transitzeit MTT nach Gleichung (40) das Element passiert (Gleichung (43)):

$$rBF = \frac{rBV}{MTT} \qquad (43)$$

Die der Messung zugängliche Kontrastmittelkonzentration im Gewebe $C_{tiss}(t)$ resultiert aus der Transformation, der das Gefäßbett des Gewebes einen deltafunktionsförmigen Bolus unterwerfen würde, R, gefaltet mit dem Konzentrations-Zeit-Verlauf des anströmenden Blutes, also der Arteriellen Inputfunktion (AIF), und dem Fluss F_{tiss} als Proportionalitätsfaktor. Die AIF wird in guter Näherung durch die Konzentrationskurve C_{art} eines in hinreichender Nähe zum untersuchten Gewebe befindlichen großen arteriellen Gefäßes repräsentiert (Gleichung (44)).

$$C_{tiss}(t) = F_{tiss} * R \otimes C_{art} \qquad (44)$$

Hier ergeben sich formale Parallelen zu systemtheoretischen Ansätzen [126], indem die arterielle Inputfunktion als Eingangssignal und deren Veränderung durch das untersuchte Gewebe als Punktbildfunktion (point spread function) aufgefasst werden können. Folglich steht zur Extraktion von R eine breite methodische Palette von algebraischen Ansätzen bis zur Entfaltung über verschiedene Transformationen prinzipiell zur Verfügung.

3.1.3 Modelle für die Zeitabhängigkeit der intravasalen KM-Konzentration

Ein Problem bei der Berechnung von Perfusionsparametern nach der Indikator-Verdünnungs-Therorie aus kontrastmitteldynamischen Messungen besteht in der Überlagerung der dem ersten Durchlauf (first pass) entsprechenden KM-Konzentration sowohl mit dem zweiten Durchlauf (second pass - vgl. z. B. Abbildung 41) als auch unter Umständen zusätzlich mit durch KM-Aufnahme in das Gewebe bedingten Einflüssen auf die Signalintensität. Hierfür sind im wesentlichen vier Lösungsansätze gebräuchlich:

1. Bewertung der Konzentrationskurve für intravasales Kontrastmittel nur bis zu einem bestimmten Zeitpunkt (z. B. dem Peakmaximum)
2. Anpassung einer Modellfunktion, die nach Boluspassage per Definition auf die Konzentration 0 zurückgeht
3. Modellierung des Konzentrationsverlaufes unter Berücksichtigung wenigstens von einem dieser Störeinflüsse
4. Modellierung des von der arteriellen Inputfunktion entflochtenen intraarteriellen KM-Konzentrationsverlaufes

zu 1.: Bei dieser Strategie werden die Perfusionsparameter auf Grundlage einer erheblich reduzierten Anzahl von Messpunkten berechnet – die Reduktion systematischer Fehler erfolgt hier um den Preis der Erhöhung des zufälligen Fehlers. Die Methode ist dafür hinreichend robust und leicht implementierbar.

zu 2.: Hier wird als Modellfunktion meist eine Gamma-Variate-Funktion eingesetzt (Gleichung (45), z. B. [144, 61, 182, 164, 124])

$$C(t) = A * (t - t_0)^B * \exp\left(-(t - t_0)/D\right) \qquad t > t_0$$
$$C(t) = 0 \qquad t \le t_0 \qquad (45)$$

Deren Anwendung ist aus mehreren Gründen nicht trivial: Sie spiegelt bewusst nicht den Verlauf der anzupassenden Messpunkte wieder; es sind vier freie Parameter anzupassen, und die Parameter sind stark voneinander abhängig (wesentlich voneinander verschiedene Parameterkonstellationen für einander sehr ähnliche Kurvenverläufe möglich).

zu 3.: In dieser Herangehensweise wird der tatsächliche Verlauf der Messgröße konsequenter berücksichtigt. Auch hier kann der first pass durch eine (evtl. vereinfachte) Gamma-Variate-Funktion dargestellt werden; zusätzlich wird z. B. ein den KM-Übertritt in das Gewebe sowie die Rezirkulation berücksichtigender Term hinzugefügt. Eine diesem Gedanken folgende Modellfunktion wurde vom Autor in Abschnitt 5.4 vorgeschlagen.

In einem bei Simpson et al. [162] zitierten Ansatz wird ein Rezirkulation und interstitielles KM erfassender exponentieller Term benutzt, gleichzeitig wird die Anzahl der freien Parameter der Gamma-Variate-Funktion reduziert (Gleichung (46))

$$C(t) = A * \frac{t}{t_p^2} * \exp\left(\frac{-t}{t_p}\right) + B * \left(1 - \exp\left(\frac{-C * t}{t_p}\right)\right) \tag{46}$$

Eine ähnliche Denkweise verfolgt die in Abschnitt 5.2 vorgeschlagene Methodik, wobei dort Zusatzinformation aus einer Doppelecho-Messung benutzt wird.

Weiterhin ist es prinzipiell denkbar, die Modellfunktion um einen Term für den second pass zu erweitern. Dies ist wegen der durch die hohe Zahl freier Parameter bedingte Instabilität jedoch nicht üblich.

zu 4.: Die Entflechtung des Zeitverlaufes von ΔR_2^* von der arteriellen Inputfunktion gemäß Strategie 4 stellt die vom Ansatz her exakteste, aber auch aufwendigste und mit der größten Zahl von Fehlermöglichkeiten verbundene Methode dar. Sie wird u.a. von der Heidelberger Gruppe (Brix et al. [18], Rempp et al. [144], Scholdei et al. [159]), aber auch in etlichen anderen, häufig methodisch orientierten Arbeiten (z. B. Vonken et al. [173], Böck et al. [13]) untersucht bzw. angewandt. Fehlerquellen sind hier die insbesondere die Bestimmung der AIF selbst (Klose et al. [102], Benner et al. [10]) sowie Aufschaukelungs-Effekte von Fehlern bei der Entflechtung (sowohl im eigentlichen Sinne als auch über algebraische Ansätze (Single Value Decomposition - SVD)). Dies kann insbesondere kritisch werden, wenn die AIF mit geringer Zeitauflösung und/oder unzureichendem Signal-Rausch-Verhältnis bestimmt wurde. Liu et al. [117] und Ostergaard et al. [133] empfehlen hier das Verwerfen der eine gewisse Schwelle unterschreitenden Diagonalelemente einer bei der SVD zentralen Matrix, was einer Glättung der AIF zumindest verwandt sein dürfte. Alternativ können AIF und/oder Response-Funktion mit Modellfunktionen parametrisiert werden [133, 163]. Ostergaard et al. [132] benutzten für die Response-Funktion eine mit der MTT als Zeitkonstante abfallende e-Funktion. Eine Modellierung höherer Ordnung erwies sich aus Stabilitätsgründen als nicht gerechtfertigt.

3.2 Entkopplung einander überlagernder Effekte

3.2.1 Trennung von Aufnahme- und Passage-Effekten durch Einsatz intravasaler Kontrastmittel

Wenn eine Beziehung zwischen der KM-Konzentration und einem kernspintomographisch zugänglichen Parameter (z. B. Signalintensität, T_2^*) bekannt ist, kann aus dessen Zeitverlauf während/nach Injektion des KM über die Indikator-Verdünnungs-Theorie (Abschnitt 3.1.2) auf Perfusionsparameter wie relatives Blutvolumen, Blutfluss oder mittlere Passagezeit (MTT) geschlossen werden. In Abhängigkeit von Gefäß- und Gewebe-Eigenschaften sowie von Transport-Eigenschaften des Kontrastmittels (insbesondere von dessen Molekülgröße) tritt jedoch ein Teil des das Gefäßbett passierenden Kontrastmittels in das umgebende Gewebe über und beeinflusst auch dort die Relaxationszeiten (Abbildung 41). Da die Kapillargefäße in der klinischen MRT räumlich nicht aufgelöst werden können, verursacht dieser Effekt einen u. U. erheblichen systematischen Fehler. Dieser kann ursächlich durch Einsatz makromolekularer Kontrastmittel reduziert werden. Deren vergleichsweise langsame renale Ausscheidung begrenzt jedoch die Anwendbarkeit [105].

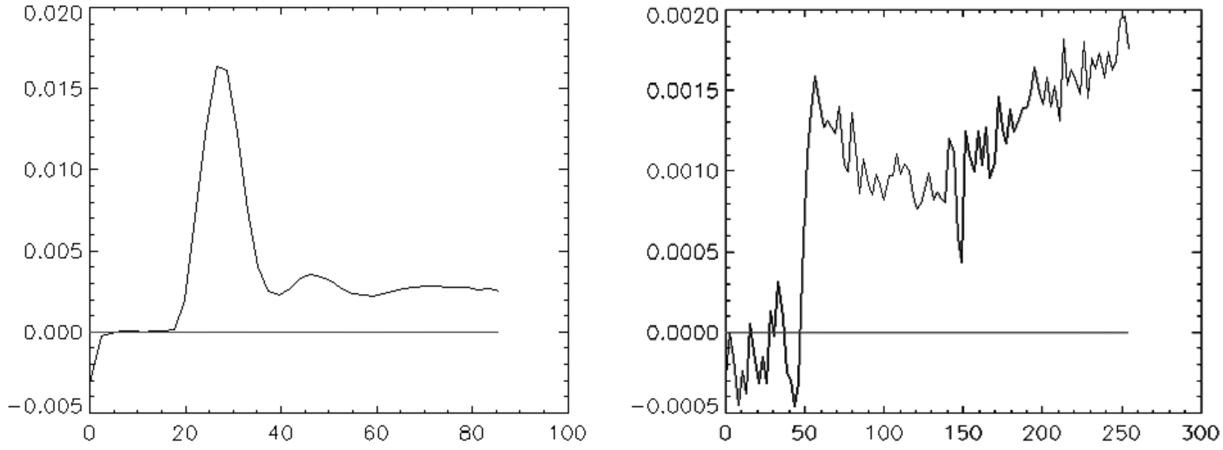

Abbildung 41: Veränderung der Relaxationsrate ΔR_2^* nach Bolus-Injektion eines paramagnetischen Kontrastmittels. Links: stark perfundiertes Gewebe (graue Hirnsubstanz) – die gemessene KM-Konzentration ist nach der Boluspassage von der Rezirkulation im Blutgefäßsystem bestimmt, rechts geringer perfundiertes Gewebe (Beckenmuskulatur) – die langsam weiter ansteigende Konzentration zeigt den KM-Übertritt in das Gewebe an.

3.2.2 Entkopplung über Modellannahmen

Im Fall von EPI-Messungen stellt sich ein Gleichgewichtszustand der Longitudinalmagnetisierung erst nach einigen Messungen ein. Insbesondere findet die erste Messung in einer EPI-Zeitreihe eine $T_R = \infty$ entsprechende Ausgangsmagnetisierung vor. Damit ist eine pixelweise Bestimmung von T_1 prinzipiell möglich. Für die Abhängigkeit der Signalintensität von den Relaxationszeiten kann in ausreichender Näherung Gleichung (15) benutzt werden, wobei T_1 als nur von der extravasalen KM-Konzentration C_{ext} abhängig (Gleichung (47)), und T_2^* als von C_{ext} und den boluspassage-bedingten Suszeptibilitätsinhomogenitäten (die sich in einem gewissen ΔR_2^* äußern) abhängig (Gleichung (48)) beschrieben wird [57]:

$$\frac{1}{T_1(t)} = \frac{1}{T_{1,0}(t)} + r_1 * C_{ext}(t) \tag{47}$$

$$\frac{1}{T_2^*(t)} = \frac{1}{T_{2,0}^*} + r_2 * C_{ext}(t) + \Delta R_2^*(t) \tag{48}$$

Die Relaxivitäten r_i des Kontrastmittels dürften aus in-vitro-Messungen übernehmbar sein (für Gd-DTPA gelten $r_1 = 4{,}5\,mM^{-1}s^{-1}$ und $r_2 = 5{,}5\,mM^{-1}s^{-1}$ [168]). Unter Vernachlässigung einer Rückdiffusion von Kontrastmittel aus dem Interstitium in die Gefäße kann C_{ext} aus der KM-Konzentration im Blut (genauer: im Blutplasma, dessen Volumenanteil am Blut durch den gefäßdurchmesser-abhängigen Hämatokrit beeinflusst wird) C_p geschätzt werden (Gleichung (49)):

$$C_{ext}(t) = k * \int_0^t C_p(t')\, dt' \tag{49}$$

k ist eine gewebe- (bzw. läsions-) abhängige Konstante („capillary transfer constant"). Zur Separation der T_1- und T_2^*-bedingten Signalintensitäts-Änderungen wird die Zeitabhängigkeit von C_p benötigt. Bei Untersuchungen im Hirnschädel kann man bei nicht zu großen Läsionen, d.h. bei im wesentlichen intakter Blut-Hirn-Schranke, den über ein großes Volumen gemittelten Intensitätsverlauf als nur von T_2^* abhängig betrachten. C_p lässt sich hier mit ausreichender Genauigkeit durch den über eine ganze Schicht gemittelten KM-Konzentrationsverlauf ersetzen, der sich aus der Intensitäts-Zeit-Kurve mit Gleichung (26) berechnen lässt. In einem durch Einsetzen von (47), (48) und (49) in Gleichung (15) formulierbaren Ansatz für eine Kurvenanpassung des gemessenen Intensitätsverlaufes bleiben damit als freie Parameter offenbar ρ aus Gleichung (15) und k aus Gleichung (49), sowie ΔR_2^* aus Gleichung (48)[8]. Zusätzlich wird in [57] eine Verschiebung sowie Verbreiterung/Verschmälerung des Zeitverlaufes der Boluspassage in der untersuchten Region verglichen mit der Boluspassage in der gesamten Schicht durch zwei zusätzliche freie Parameter berücksichtigt. Die Wirkung dieser Korrektur ist in Abbildung 42 dargestellt.

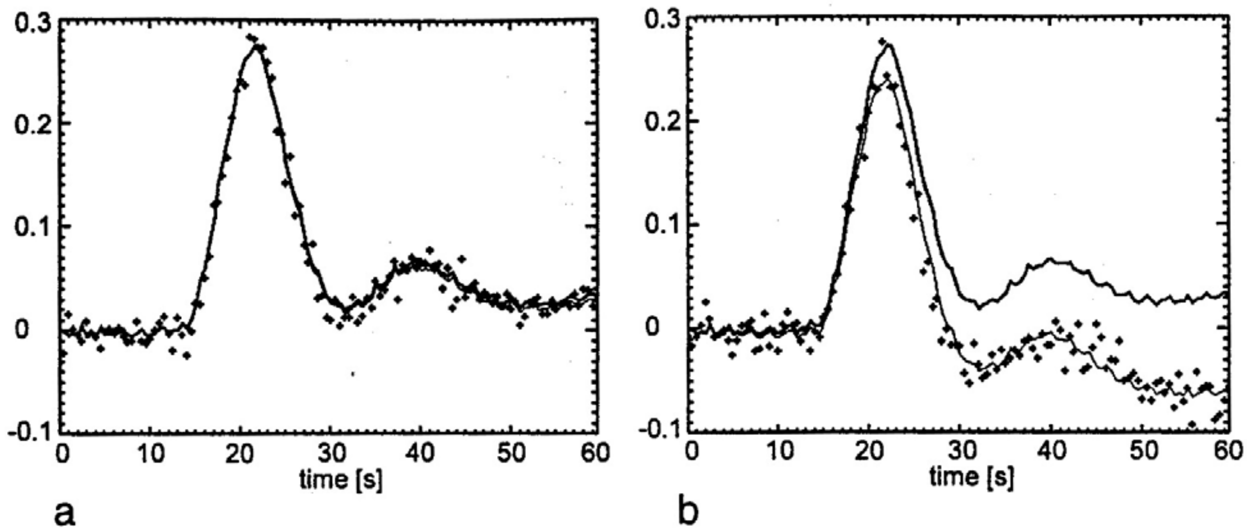

Abbildung 42: Mittels EPI-Messung bestimmte KM-Konzentrationskurven aus weißer Hirnsubstanz (a) und einem KM aufnehmenden MS-Plaque (b). Dünne Linie: Anpassung der Modellfunktion; dicke Linie: korrigierte Kurve nach Elimination von durch die gestörte Blut-Hirn-Schranke bedingten Signaländerungen (aus [57]).

Etwas konsequenter kann als Eingangsgröße C_p in Gleichung (49) die in großen hirnversorgenden Gefäßen messbare AIF benutzt werden. Damit und unter Anwendung der in Abschnitt 5.1 beschriebenen Doppelecho-Korrektur kann der Zeitverlauf der Relaxationsrate ΔR_2^* vom extravasalen Anteil bereinigt werden [172].

[8] Die Festlegung von ΔR_2^* in Gleichung (48) ist in [57] nicht schlüssig beschrieben. Naheliegend wäre, den aus der gesamten Schicht bestimmten KM-Konzentrationsverlauf – ggflls. nach Streckung/Stauchung entlang Intensitäts- und Zeitachse sowie zeitlicher Verschiebung – auch hier zu benutzen.

3.2.3 Vorinjektion von Kontrastmittel

Ein weiterer Ansatz, KM aufnehmende Gewebe der MR-tomographischen Perfusionsmessung zugänglich zu machen, ist die von Aronen et al. vorgeschlagene Verringerung des maximalen KM-Konzentrations-Gradienten zwischen Blutgefäßen und Kontrastmittel durch eine Vor-Injektion etwa 10 bis 15 Minuten vor der Perfusionsmessung [5, 4]. Ein wesentlicher Aspekt dieser Strategie dürfte in der Ausnutzung der Nichtlinearität zwischen ΔR_1 und der Signalintensität bestehen, die Vor-Injektion führt damit bei gegebener Repetitionszeit zu einer geringeren Abhängigkeit der Signalintensität von den KM-bedingten T_1-Änderungen (vgl. Gleichungen (14) für Spinecho bzw. (15) für Gradientenecho sowie Abbildung 43).

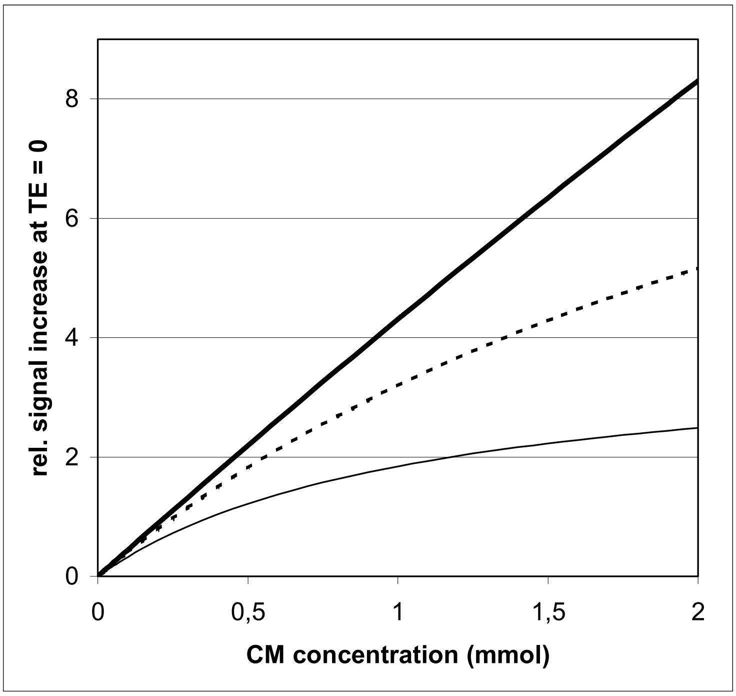

Abbildung 43: Abhängigkeit der Signalintensität einer idealisierten FLASH-Sequenz (T_E = 0) von der Gd-DTPA-Konzentration für Gewebe mit einer T_1 von 1000 ms, zu messen bei einer Repetitionszeit T_R von 24 ms. Flipwinkel α = 15° (—),α = 20° (- - -) und α = 30° (—).

Eine vergleichende Analyse dieser Methode mit Doppelecho-Messungen gemäß Abschnitt 5.1 durch Heiland et al. [60] zeigt, dass damit der Einfluss der T_1-Verkürzung während der Boluspassage zwar verringert wird, in Abhängigkeit vom Ausmaß der KM-Aufnahme in die untersuchte Region jedoch ein mehr oder weniger erheblicher systematischer Fehler bei der Bestimmung des rCBV verbleibt.

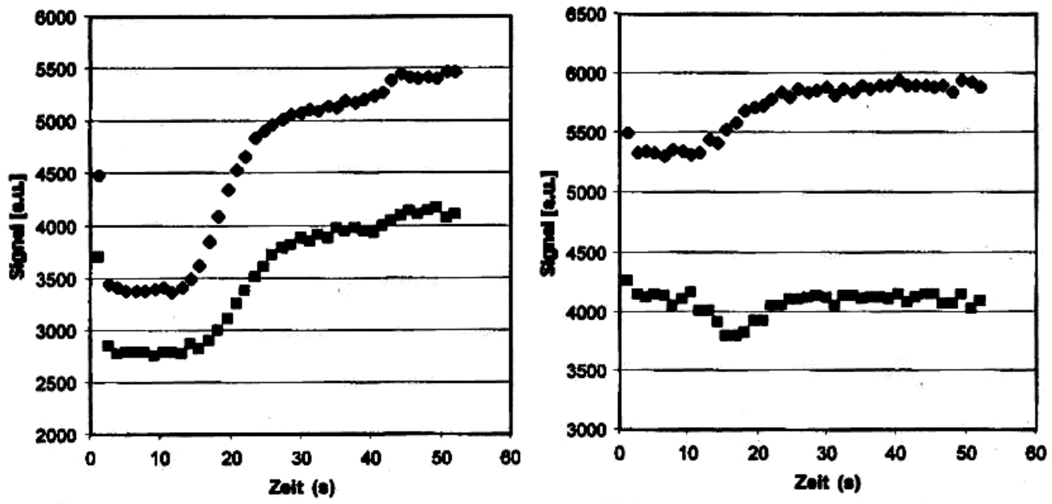

Abbildung 44: Intensitätsverlauf des frühen Echos (T_E = 6 ms, ◆) und des späten Echos (T_E = 23,6 ms, ■) einer Doppelecho-FLASH-Sequenz (T_R = 30 ms, α = 15°) vor (links) bzw. nach Prä-Injektion von 0,1 mmol/kg Gd-DTPA (aus [60])

3.2.4 Zusammenfassung

Für MR-Perfusionsmessungen an KM aufnehmenden Läsionen gibt es unter Einbeziehung der weiter unten beschriebenen Beiträge des Autors im wesentlichen vier unterschiedliche Ansätze:

1. Einsatz intravasal verbleibender Kontrastmittel (Abschnitt 3.2.1),
2. Abschätzung des Beitrages des interstitiell aufgenommenen Kontrastmittels zum Zeitverlauf des gemessenen Signals auf Grundlage von AIF-Schätzungen und von Annahmen über deren Verknüpfung mit der KM-Konzentration im Interstitium (Abschnitt 3.2.2),
3. Vorinjektion von Kontrastmittel (Abschnitt 3.2.3)
4. Bereinigung T_2^*-gewichteter Messungen vom Einfluss der gleichzeitigen T_1-Verkürzung über Doppelecho-Sequenzen (Abschnitt 5.1).

Im letztgenannten Fall ist weiterhin der Einfluss des interstitiell gespeicherten KM auf T_2^* behandelbar (Abschnitt 5.2).

Die Anwendung intravasal verbleibender KM ist durch deren vergleichsweise langsame renale Ausscheidung begrenzt [105]. Doppelecho-Messungen gemäß Abschnitt 5.1 sind bis heute auf nur eine (oder zumindest wenige) Schichten begrenzt. Zukünftige Sequenzentwicklungen sollten die Erfassung eines größeren Volumens mit dieser Strategie ermöglichen. Ein Teil der modellbasierten Ansätze lässt sich auf EPI-Messungen anwenden, mit denen bei ausreichender Zeitauflösung deutlich mehr Schichten erfasst werden können. Sie erfordern jedoch die Bestimmung einer Arteriellen Inputfunktion, was mit erheblichen Unsicherheiten verbunden ist (Die Funktionsfähigkeit der in Abschnitt 5.2 vorgestellten Algorithmen zeigt, dass die Abhängigkeit der Relaxationsrate ΔR_2^* von der KM-Konzentration von der geometrischen Verteilung des KM abhängt). Bezüglich der Vorinjektions-Methode zeigten Heiland et al. [60] die verbleibenden systematischen Fehler in der Bestimmung von Perfusionsparametern.

4 Material und Methoden

4.1 Patienten

MR-Mammographie: Für die retrospektive Auswertung kontrastmitteldynamischer MR-Mammographien an 0.5 Tesla standen Daten von 95 histologisch gesicherten Mamma-Läsionen zur Verfügung. Einschlusskriterien für diese Patientinnen waren: Differenzierung fokaler Läsionen mammographisch und sonographisch unklarer Dignität, in der Regel präoperativ; in Einzelfällen postoperativ bei unvollständiger Tumorresektion. Die Transformation des bei 0.5 Tesla gewonnenen statistischen Wissens auf Messbedingungen bei 1.5 Tesla erfolgte anhand der Untersuchung von insgesamt ca. 85 Regionen in Mammae von 35 Patientinnen.

Hirntumoren und -metastasen: In die retrospektive Analyse von Doppelecho-Messungen bei 0.5 Tesla aus den Jahren 1992 bis 1998 gingen die Daten von 138 Patienten mit vorwiegend extraaxialen Hirnläsionen (Meningeome, Neurinome, Lymphome, Metastasen, Glomustumoren sowie Glioblastome) ein.

Pharyngeale Tumoren: Zum Zeitpunkt der Analyse umfasste die Stichprobe 20 Patienten mit primär inoperablen pharyngealen bzw. laryngealen Plattenepithelkarzinomen in unterschiedlichen Therapiestadien (prätherapeutisch bis 12 Monate nach Ende der Strahlentherapie). Insgesamt lagen Daten von 69 MR-Untersuchungen vor. Zwei Patienten wurden von der weiteren Auswertung ausgeschlossen (vgl. Abschnitt 5.5.1). Die 18 einbezogenen Patienten hatten zum Studien-Eintritt ein mittleres Alter von 55 Jahren und 10 Monaten (Minimum 43 Jahre und 9 Monate, Maximum 67 Jahre und 6 Monate) sowie ein mittleres Körpergewicht von 70 kg (Minimum 56 kg, Maximum 90 kg). Alle Patienten litten an histologisch gesicherten Plattenepithel-Karzinomen.

4.2 Benutzte Geräte und Sequenzen

Die Messungen wurden zunächst an einem 0,5-Tesla-Scanner (Philips Gyroscan T5 XPA) durchgeführt. Da die Signalreduktion durch Suszeptibilitäts-Inhomogenitäten mindestens proportional zur Feldstärke des äußeren Magnetfeldes ist [123], wurde die Methodik baldmöglichst auf einen 1,5-Tesla-Scanner (Siemens Magnetom Vision) übertragen. Die Studie zu pharyngealen Tumoren wurde ausschließlich bei 1,5 Tesla durchgeführt. Zur Anwendung kamen folgende Sequenzen:

Doppelecho-Studie bei 0,5 Tesla:
2D-FFE, $T_{E,1}$ = 11 ms, $T_{E,2}$ = 25 ms, T_R = 70 ms, α = 15°, zeitliche Auflösung ca. 5 s, Scanmatrix 102 * 128, nur eine Schicht messbar.

T_1-Dynamik bei 0,5 Tesla:
2D-FFE, T_E = 15 ms, T_R = 47 ms, α = 35°, zeitliche Auflösung ca 11 s, Scanmatrix 256^2 (nur eine Schicht messbar) bzw. 128^2 (zwei zueinander parallele Schichten messbar).

STIR bei 0,5 Tesla:
IR, T_E = 30 ms, T_R = 1400 ms, T_I = 140 ms

T_1-Volumen bei 0,5 Tesla:
3D-FFE, TE = 13 ms, TR = 52 ms, α = 60°; 0,8 mm Schichtdicke interpoliert

T_1-Dynamik bei 1,5 Tesla:
3D-FLASH, T_E = 4 ms, T_R = 8,1 ms, α = 20°, zeitliche Auflösung ca 60 s, Scanmatrix 256 * 256 * 64

Doppelecho-Studie bei 1,5 Tesla:
2D-FLASH, $T_{E,1}$ = 15 ms, $T_{E,2}$ = 35 ms, T_R = 50 ms, α = 30°, zeitliche Auflösung ca. 5 s, Scanmatrix 60 * 128, nur eine Schicht messbar

T_1-Volumen bei 1,5 Tesla:
3D-FLASH, TE = 6 ms, TR = 32 ms, α = 40°; 0,8 mm Schichtdicke interpoliert

EPI-Perfusionsmessung bei 1,5 Tesla:
EPI-FID, $T_{E,eff}$ = 54 ms, T_R = zeitliche Auflösung $\approx$ 1 s, α = 90°, Scanmatrix 128²

EPI-Messungen am Flussphantom:
$T_{E,eff}$ = 61 ms, α = 90°, zeitliche Auflösung = 2 s, α = 90°, 15 Schichten

Flussmessungen bei 1,5 Tesla:
2D-FLASH, T_E = 6 ms, T_R = 25 ms, α = 30°,, v_{max} = 150 cm/s, Scanmatrix 192*256, FOV = 165 * 220 cm², Schichtdicke 10 mm

Kontrastmittel-Injektor: SPECTRIS (Fa. Medrad)

4.3 Messmethoden

MR-Mammographie:
Bei 0.5 Tesla wurde(n) die suspekte(n) Läsion(en) zunächst mit Hilfe der STIR-Sequenz lokalisiert. Die Kontrastmittelaufnahme wurde mit Hilfe einer T_1-gewichteten 2D-FFE-Sequenz gemessen. Während des zweiten von insgesamt 20 dynamischen Scans wurde 0,1 mmol/kg Körpergewicht Kontrastmittel (Magnevist (Gd-DTPA), Schering) als Bolus injiziert. Vor und nach der dynamischen Studie wurde die gesamte Brust mittels 3D-FFE-Sequenz erfasst, um auch Kontrastmittelaufnahmen außerhalb der dynamisch untersuchten Schicht zumindest qualitativ bewerten zu können. Bei 1.5 Tesla konnte die gesamte Brust bei hinreichender Zeitauflösung mit einer 3D-FLASH-Sequenz erfasst werden.

MR-Perfusionsmessungen an Hirntumoren und -metastasen:
Die MR-Perfusionsmessungen erfolgten bei 0.5 Tesla unter Einsatz einer 2D-FFE-Doppelecho-Sequenz. Die Bolus-Injektion von 0.2 mmol/kg Körpergewicht Kontrastmittel (Magnevist (Gd-DTPA), Schering) erfolgte nach dem zehnten von insgesamt 25 dynamischen Scans. Zur Gewährleistung eines stationären Zustandes der Netto-Magnetisierung in z-Richtung (vgl. Abschnitt 2.2.2.2) wurde der jeweils erste dynamische Scan verworfen.

Pharyngeale Tumoren:

MR-Messungen: Die kontrastmittel-dynamischen Untersuchungen erfolgten mittels Bolus-Injektion eines paramagnetischen Kontrastmittels (Gd-DTPA, Magnevist, Schering) unter Einsatz eines KM-Injektors (SPECTRIS, Medrad). Hierbei wurden die KM-Menge zu 0,4 ml entsprechend 0,2 mmol pro kg Körpergewicht sowie der Fluss zu 2 ml/s konstant gehalten. Damit dauerte die Injektion ca. 11 bis 18 Sekunden entsprechend etwa 2 bis 3 Scans. Zur Vermeidung einer zu schnellen Verbreiterung des Bolus wurden 20 ml physiologische Kochsalzlösung mit gleichem Fluss

nachinjiziert. Vor Injektionsbeginn wurden 10 Baseline-Scans akquiriert, von denen jeweils der erste verworfen wurde, um die Einstellung eines stationären Zustandes der Nettomagnetisierung zu garantieren. Insgesamt wurden 40 Scans aufgenommen. Dies entspricht einem Zeitintervall von bis zu 2 ½ Minuten nach Beginn der Bolus-Injektion. Die Tumorvolumina wurden durch schichtweise manuelle Segmentation aus den T_1-Volumendaten bestimmt (Kittner, vgl. [98]). Hierbei wurden im Sinne eines totalen Tumorvolumens neben dem Primärtumor sowohl Metastasen als auch nekrotische Bereiche einbezogen.

Sauerstoff-Partialdruck: Messungen des (vorwiegend extrazellulären) intratumoralen pO_2 erfolgten polarographisch mittels einer automatisch in 0,5-mm-Schritten in das Gewebe vordringenden Sonde (KIMOC-6650, Eppendorf) (Appold, vgl. [98]). Die Messungen wurden unter endoskopischer Kontrolle durchgeführt. Im Durchschnitt wurden 108 Messpunkte je Tumor (Spannweite 47-193) gewonnen. Zur Auswertung wurden der Median dieser Messwerte sowie der relative Anteil von Punkten herangezogen, die einen pO_2 von 5 mm Hg unterschritten (hypoxische Fraktion).

Stoffwechsel-Aktivität: Die metabolische Aktivität der Tumoren wurde mittels Positronen-Emissions-Tomographie (PET) bestimmt. Hierzu wurde mit dem Positronenstrahler ^{18}F markierte Fluordeoxyglukose (FDG) in einer Menge injiziert, die einer Aktivität von 300 MBq entspricht. Die Bildgebung erfolgte ca. 60 Minuten nach Injektion mit einem PET-Scanner des Typs ECAT EXACT HR+ (Siemens) (Beuthien-Baumann, vgl. [98]). Regions of Interest wurden durch Schwellwert-Segmentation bei ca. 50 bis 70 % der maximalen Tracer-Aufnahme des Tumors unter visueller Kontrolle festgelegt. Aus der Summe dieser ROI-Flächen wurde ein Tumorvolumen bestimmt. Dieses unterscheidet sich prinzipbedingt von dem mittels MRT bestimmten Volumen dadurch, dass nur vitale Bereiche erfasst werden (vitales Tumorvolumen). Aus dem Mittelwert der Aktivitäten im als Tumor segmentierten Volumen wird der Standard Uptake Value berechnet (Gleichung (50):

$$SUV = \frac{activity\ in\ ROI[Bq/g]*body\ weight[g]}{injected\ activity[Bq]} \qquad (50)$$

Die zeitliche Abfolge des Einsatzes der beschriebenen Untersuchungsmethoden ist in Tabelle 8 dargestellt.

Zeitpunkt	Endoskopie	Eppendorf pO2	PET	MRT
1) Primär	X	X	X	X
2) nach 25 Gy	X			X
3) nach 50 Gy	X	X	X	X
4) Ende Strahlentherapie	X			X
5) 3 Monate nach Ende Strahlentherapie	X		X	X
6) 6 Monate nach Ende Strahlentherapie	X		X	X
7) 12 Monate nach Ende Strahlentherapie	X		X	X
8)18 Monate nach Ende Strahlentherapie	X		X	X
9)24 Monate nach Ende Strahlentherapie	X		X	X

Tabelle 8: Zeitplan der Untersuchungen im Rahmen der Studie zur Physiologie pharyngealer Tumoren.

4.4 Auswerte-Werkzeuge

Aus Gründen des Signal-Rausch-Verhältnisses erfolgten alle Analysen der Zeitverläufe der Signalintensitäten bzw. daraus abgeleiteten Größen auf der Basis von Regions of Interest. Deren Definition wie auch die Modellierungen und Quantifizierungen der Signalverläufe erfolgten mittels eines im Rahmen dieser Arbeit unter IDL (Research Systems, Inc.) entwickelten Programms. Die statistischen Analysen wurden in Excel (Microsoft) mit unter VBA entwickelten Zusatzfunktionen durchgeführt.

5 Ergebnisse

5.1 Mathematische Entkopplung der T_1- und T_2^*-Verkürzung nach Doppelecho-Messung für gefäßwand-gängige Kontrastmittel

Bei Einsatz gefäßwand-gängiger Kontrastmittel sind in gut vaskularisierten und gleichzeitig Kontrastmittel aufnehmenden Bereichen (z. B. bestimmten Tumoren) gleichzeitig Verkürzungen der T_1- und T_2^*-Relaxationszeiten zu beobachten. Bei realen Echozeiten ($T_E > 0$) und endlichen Repetitionszeiten ($T_R < \infty$) überlagern sich damit der T_1-bedingte Signalanstieg durch Kontrastmittelaufnahme und der T_2^*-bedingte Signalverlust während der Passage des KM-Bolus. Wenn die entsprechende Untersuchung als Doppelecho-Messung ausgeführt wird, können diese Effekte nach einem vom Autor [78, 79, 76] zeitgleich mit Heiland et al. [59] sowie Miyati et al. [124] entwickelten Ansatz voneinander separiert werden:

Aus den Signalintensitäts-Formeln für Spin- bzw. Gradientenecho (Gleichungen (14) und (15)) lässt sich T_2^* schätzen (Gleichung (51)):

$$T_2^* = \frac{T_{E2} - T_{E1}}{\ln\left(S_1 / S_2 \right)} \tag{51}$$

Mit der in Abschnitt 2.2.4, Gleichung (19) postulierten Proportionalität zwischen Kontrastmittel-Konzentration und Änderung der Relaxationsrate ergibt sich als Schätzwert für die KM-Konzentration Gleichung (52)

$$C(t) = \frac{1}{r} * \Delta R_2(t) = \frac{1}{r} * \frac{\ln\left(S_1(t) / S_2(t) \right) - \ln\left(S_1(0) / S_2(0) \right)}{T_{E2} - T_{E1}} \tag{52}$$

Wenn T_2^* aus Gleichung (51) bekannt ist, lässt sich die Signalintensität des ersten Echos vom Einfluss der T_2^*-Verkürzung bereinigen, also für jeden Messzeitpunkt eine hypothetische Intensität schätzen, die mit einer Sequenz ohne T_2^*-Einfluss, also mit einer Echozeit von $T_E = 0$, gemessen worden wäre (Gleichung (53)):

$$S_{TE=0}(t) = \frac{S_1(t)}{\exp\left(-T_{E2} / T_2^* \right)} = S_1(t) * \left(\frac{S_1}{S_2} \right)^{\frac{T_{E1}}{T_{E2} - T_{E1}}} \tag{53}$$

Für die quantitative Bestimmung der Veränderung von T_1 reicht die Doppelecho-Messung allein nicht aus. Dies kann z. B. aus Gleichung (14) für Spinecho-Messungen abgeleitet werden (Gleichung (54), (55), (56))

$$\frac{S_{TE=0}(t)}{S_{TE=0}(0)} = \frac{1-\exp\left(-T_{E1}/T_1(t)\right)}{1-\exp\left(-T_{E1}/T_1(0)\right)} \tag{54}$$

$$E_R = \exp\left(-T_R\left(\frac{1}{T_1}+\Delta R_1\right)\right) = \frac{S(t)}{S(0)} * \left(\exp\left(-\frac{T_R}{T_1}\right)-1\right)+1 \tag{55}$$

$$\Delta R_1 = -\left(\frac{\ln(E_R)}{T_R}+\frac{1}{T_1}\right) \tag{56}$$

Für Gradientenecho-Messungen gelten analog die Gleichung (57), (58) und (56):

$$\frac{S_{TE=0}(t)}{S_{TE=0}(0)} = \frac{\left(1-\exp\left(-T_{E1}/T_1(t)\right)\right) * \left(1-\cos(\alpha) * \exp\left(-T_{E1}/T_1(0)\right)\right)}{\left(1-\exp\left(-T_{E1}/T_1(0)\right)\right) * \left(1-\cos(\alpha) * \exp\left(-T_{E1}/T_1(0)\right)\right)} \tag{57}$$

$$E_R = \exp\left(-T_R\left(\frac{1}{T_1}+\Delta R_1\right)\right) = \frac{\frac{S(t)}{S(0)} * \left(\exp\left(-\frac{T_R}{T_1}\right)-1\right)+1-\cos(\alpha) * \exp\left(-\frac{T_R}{T_1}\right)}{\frac{S(t)}{S(0)} * \cos(\alpha) * \left(\exp\left(-\frac{T_R}{T_1}\right)-1\right)+1-\cos(\alpha) * \exp\left(-\frac{T_R}{T_1}\right)} \tag{58}$$

Es ist also eine zusätzliche Messung mit anderer Repetitionszeit erforderlich, um zunächst den Ausgangswert der Relaxationszeit $T_1(0)$ vor Kontrastmittelgabe zu bestimmen.

5.2 Empirische Trennung von perfusions- und KM-Aufnahme-bedingter T_2^*-Verkürzung

Im folgenden Korrekturansatz [70] wird bezüglich der Dephasierung unterschieden zwischen der über die Relaxivität r_2 beschreibbaren Spin-Spin-Relaxation und der durch Suszeptibilitätsunterschiede zwischen KM-angereicherten Blutgefäßen und der Umgebung verursachten zusätzlichen Dephasierung. Letztere wird hier mit einer „suszeptibilitätsbedingten Relaxivität“ r_2^* beschrieben, wobei sich die Relaxationsrate R_2^* darstellt als $R_2^* = (r_2 + r_2^*) * C$ (vgl. Abschnitt 2.2.4.4).

Des weiteren setzt der Ansatz die Messung mittels Doppelecho-FLASH-Sequenz sowie die in Abschnitt 5.1 beschriebene Entkopplung von T_1- und T_2^*-bedingten Signalintensitätsveränderungen voraus. Für Gewebe, die kein KM aufnehmen, ist die über Gleichung (52) berechenbare Relaxationsrate ΔR_2^* in guter Näherung proportional zur intravasalen KM-Konzentration [14]. Im Fall KM aufnehmender Läsionen dagegen wird ΔR_2^* wie in Abschnitt 3.2.2 beschrieben jedoch auch durch das in das interzellulare

Kompartiment eingelagerte KM beeinflusst, was zu relativ hohen Werten nach Ende der ersten Boluspassage führt (Abbildung 45 - **+++**)

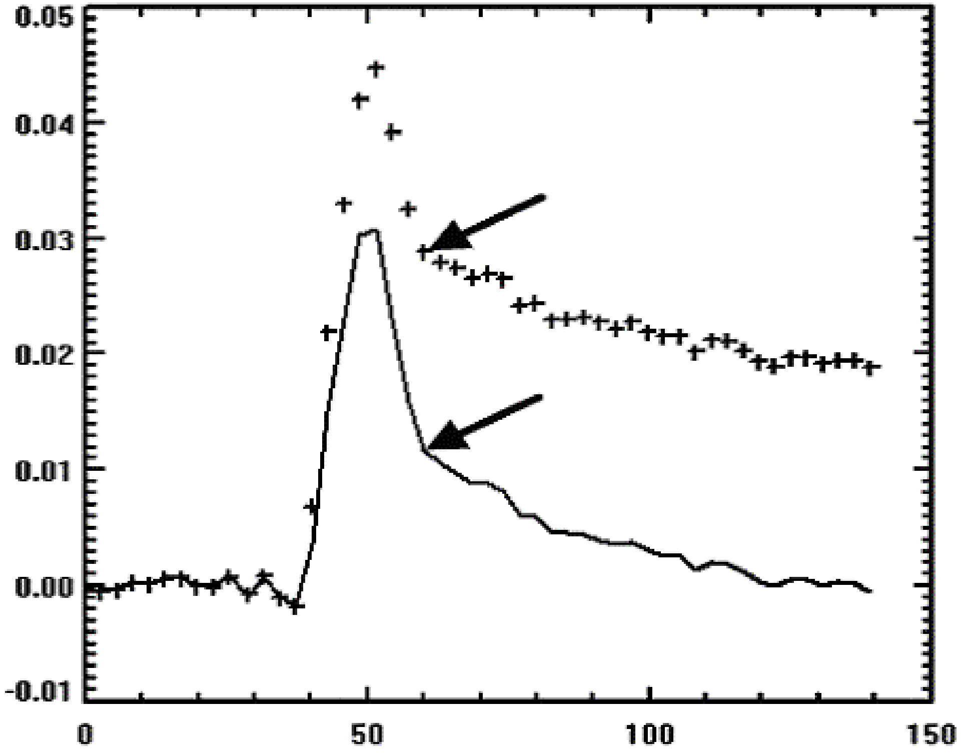

Abbildung 45: Perfusionsmessung an dem Tumor im rechten Halsbereich (siehe Abbildung 66 a). $\Delta R_2^*(t)$ nach Doppelecho-Korrektur (**+++**) und nach Bereinigung von interstitiellem KM auf Grundlage der Signalintensität bei $T_E = 0$ (—). Der Abzweig der langsamer auswaschenden Komponente (Pfeile) reduziert sich durch die Korrektur von 63 % auf 35 % des Maximums von ΔR_2^*. Das Maximum selbst ist auf 67 % seines unkorrigierten Wertes abgesenkt.

Unserer Meinung nach ist der kritische Schritt der in Abschnitt 3.2.2 dargestellten, Modellannahmen benutzenden Algorithmen die Bestimmung der AIF. Haselhorst et al. (Abschnitt 3.2.2, [57]) benutzen in ihrer Modellierung den Verlauf von ΔR_2^* im die Läsion weiträumig umgebenden Hirngewebe (Voraussetzung: intakte Blut-Hirn-Schranke) als zur AIF proportional. Dabei wird die Verteilung der KM-Ankunftszeiten im Hirn vernachlässigt. Vonken et al. [172] bestimmen die AIF von großen hirnversorgenden Gefäßen. Dies wiederum bewerten Klose et al. [102] als zu unsicher für eine quantitative Bestimmung von KM-Konzentrationen. Dieser Ansatz vernachlässigt die unterschiedlichen Beiträge, die die Suszeptibilitäts-Inhomogenitäten in einem relativ großen Gefäßquerschnitt im Vergleich zu einem mit Kapillaren durchsetzten Voxel zum Signalverlust und damit zu ΔR_2* leisten ([119], Abschnitt 2.2.4.4). Nach unserer Erfahrung reagiert die Messung einer intravasalen AIF sehr empfindlich auf die zur Auswahl der einbezogenen Pixel benutzen Kriterien (Abbildung 66).

Das Ziel des folgenden Ansatzes ist daher die Extraktion des Beitrages der intravasalen KM-Konzentration zu ΔR_2^* ohne Annahmen zum Übertritt des KM in das Gewebe und

ohne die Notwendigkeit der Bestimmung einer AIF, um an KM aufnehmenden Läsionen zuverlässige Perfusionsparameter bestimmen zu können.

5.2.1 Methode

Algorithmen: Beide Relaxationsraten R_1 und R_2^* werden sowohl durch die interstitiellen („externen“) als auch durch die intravasalen KM-Konzentrationen $C_{ext}(t)$ und $C_{ves}(t)$ bestimmt. Dies kann in guter Näherung durch Gleichungen (59) und (60) beschrieben werden:

$$\Delta R_1(t) = r_1 * \left((1 - rBV) * C_{ext}(t) + rBV * C_{ves}(t) \right) \quad (59)$$

$$\Delta R_2^*(t) = \left(r_2 + r_{2,ext}^*\right) * (1 - rBV) * C_{ext}(t) + \left(r_2 + r_{2,ves}^*\right) * rBV * C_{ves}(t) \quad (60)$$

Hierbei beschreibt $r_{2,ves}^*$ die durch die Suszeptibilitäts-Inhomogenitäten zwischen Kapillaren und umgebendem Gewebe verursachte Komponente der Relaxivität und $r_{2,ext}^*$ die entsprechende suszeptibilitäts-bedingte Dephasierung durch das interstitielle KM. rBV ist das relative Blutvolumen im untersuchten Gewebe. In einer gewichteten Differenz der Gleichungen (59) und (60) kann der Einfluss von $C_{ext}(t)$ eliminiert werden, wenn der Korrekturfaktor zu $x = \frac{r_2 + r_{2,ext}^*}{r_1}$ gewählt wird (Gleichung (61)).

$$\begin{aligned} &\Delta R_2^*(t) - x * \Delta R_1(t) = \\ &\left(r_2 + r_{2,ext}^* - x * r_1\right) * (1 - rBV) * C_{ext}(t) + \left(r_2 + r_{2,ves}^* - x * r_1\right) * rBV * C_{ves}(t) \end{aligned} \quad (61)$$

Damit ergibt sich die „interstitium-bereinigte“ Relaxationsrate $\Delta R_{2,corr}^*(t)$ gemäß Gleichung (62)

$$\Delta R_{2,corr}^*(t) = \Delta R_2^*(t) - x * \Delta R_1(t) = \left(r_{2,ves}^* - r_{2,ext}^*\right) * rBV * C_{ves}(t) \quad (62)$$

Aus einem Vergleich der Maxima der Relaxationsraten-Änderungen $\Delta R_2^*(t)$ und $\Delta R_{2,corr}^*(t)$ lässt sich für die hier benutzten Messbedingungen $r_2 + r_{2,ext}^* < 0.3 * r_{2,ves}^*$ ableiten. Die durch von interstitiellem KM verursachte Suszeptibilitäts-Inhomogenitäten bedingte Dephasierung $r_{2,ext}^*$ dürfte klein im Vergleich zu der gefäßbedingten Komponente $r_{2,ves}^*$ sein, da das interstitielle KM viel feiner im umgebenden Gewebe verteilt ist als die Kapillarräume. Unter Annahme der in-vitro-Werte für r_1 von 4,5 $mM^{-1}s^{-1}$ und r_2 von 5.5 $mM^{-1}s^{-1}$ [168] ergibt sich für den Korrekturfaktor x eine Größenordnung von 1.

Die Kenntnis von r_1, r_2, $r_{2,ext}^*$ und $r_{2,ves}^*$ ermöglicht im Prinzip sogar eine Korrektur von ΔR_1 (Gleichung (63)):

$$\Delta R_{1,corr}(t) = r_1 * (1 - rBV) * C_{ext}(t) = \Delta R_1 * \left(1 + \frac{r_2 + r^*_{2,ext}}{r^*_{2,ves} - r^*_{2,ext}}\right) - \frac{r_1}{r^*_{2,ves} - r^*_{2,ext}} * \Delta R^*_2 \qquad (63)$$

Um ΔR_1 aus den Signalintensitätsverläufen ableiten zu können, werden Ausgangswerte für T_1 benötigt. Deren Bestimmung erfordert mindestens eine Messung mit anderen T_R oder α. Da sich in Abhängigkeit von der Scanner-Software zwischen den Messungen die Verstärker-Einstellungen geändert haben können, können solche Berechnungen außerhalb der vom Hersteller gelieferten Software fehlerhaft sein. Außerdem reagiert die Bestimmung von T_1-Werten aus z. B. mittels FLASH-Sequenzen gewonnenen Signalintensitätsverhältnissen empfindlich auf den effektiven Flipwinkel, der durch die Energie des RF-Impulses gegeben ist. In diesen Fällen können über Messungen an externen oder internen Standards Korrekturfaktoren für die Signalverhältnisse gewonnen oder aber effektive Flipwinkel geschätzt werden. Für Untersuchungen im Hirnschädel bietet sich als interner Standard normales Hirnparenchym an, dessen T_1-Relaxationszeit in der Größenordnung von 900 … 1000 ms liegt.

Andererseits ist unter entsprechenden Bedingungen bezüglich Messbedingungen und KM-Konzentration die relative Signalintensitätsänderung zum „nullten Echo“ (also die gemäß Abschnitt 5.1 auf $T_E = 0$ extrapolierte Intensität) $\Delta S_0(t) = \frac{S_{TE=0}(t) - S_{TE=0}(0)}{S_{TE=0}(0)}$ in hinreichender Näherung proportional zu ΔR_1 und damit zur KM-Konzentration (Abbildung 43, in praxi muss ein Kompromiss zwischen Linearität und Signal-Rausch-Verhältnis gefunden werden (bei T_R = 24 ms und T_1 = 1000 ms beträgt das Signal vor KM-Gabe für Flipwinkel α = 15° oder 20° nur 5% bzw. 25% des bei α = 30° erzielbaren Signals)).

Vonken et al. benutzten $\Delta S_0(t)$ als Plausibilitätstest, indem sie es mit dem Anteil an $\Delta R_2^*(t)$ verglichen, den sie dem interstitiellen KM zuordneten [172]. Uematsu et al. [171] definierten mit Hilfe des T_1-abhängigen Terms der Doppelecho-Korrektur einen „leakage index“. Es ist also unter gewissen Bedingungen möglich, ΔR_1 durch eine zu ΔS_0 proportionale Größe zu ersetzen (siehe auch Abbildung 43). Wenn die intravasale KM-Konzentration zu einem Zeitpunkt t_{late} hinreichend lange nach der Bolus-Injektion als vernachlässigbar betrachtet wird, kann der Korrekturfaktor x durch Einstellung von $\Delta R^*_{2,corr}(t_{late})$ auf Null in Gleichung (62) bestimmt werden (Abbildung 46 c und f). Für t_{late} kann das Ende der dynamischen Studie (ca. 3 Minuten nach Bolus-Injektion) benutzt werden[9]. Die Annahme $C_{ves}(t_{late}) = 0$ führt wegen der KM-Rezirkulation im Prinzip zur Überkompensation. Die dadurch verursachte Unterschätzung der korrigierten Konzentrationswerte beläuft sich jedoch für schnell und stark KM aufnehmende Läsionen auf nicht mehr als ca. 10 % (entsprechend dem Verhältnis zwischen Maximum und Wert nach der zweiten Passage („second pass“) bei unter unseren Injektionsbedingungen bestimmten AIF). Abbildung 45 zeigt, dass diese

[9] Die hier benutzte Denkweise hat Ähnlichkeit mit der Idee eines Aufladungsterms wie in [162] angegeben (Gleichung (46)); macht aber keine Annahmen oder zusätzliche freien Parameter für die Geschwindigkeit der KM-Aufnahme erforderlich.

Überkompensation im Vergleich zu den Veränderungen charakteristischer Merkmale von $\Delta R_2^*(t)$ von untergeordneter Bedeutung ist.

<u>Messungen:</u> Die Messungen wurden an 1,5-Tesla-Scannern (Magnetom Vision, Magnetom Symphony; beide Siemens) durchgeführt unter Einsatz von Doppelecho-FLASH-Sequenzen (Vision: siehe Abschnitt 7; Symphony (Standort: Universität Frankfurt): $T_{E,1}$ = 5 ms, $T_{E,2}$ = 20 ms, T_R = 24 ms, α = 30°) durchgeführt. Der Korrekturalgorithmus wurde bei 15 Patienten mit pharyngealen Tumoren angewandt. Bei 8 Patienten wurden invasive pO_2-Messungen im Tumor vor Beginn der Strahlentherapie und bei drei Patienten zusätzlich nach 50 Gy durchgeführt. Obwohl die invasive pO_2-Messung keineswegs als Gold-Standard bezeichnet werden kann, stellt sie einen unabhängigen, von der Perfusion abhängigen Parameter dar. Daher wird das Bestimmtheitsmaß zwischen pO_2 und den mittels MRT bestimmten Perfusionsparametern als Maß für deren diagnostische Wertigkeit betrachtet.

5.2.2 Ergebnisse

Abbildung 45 und Abbildung 46 zeigen, dass die oben beschriebenen Korrekturen die Plausibilität des Zeitverlaufes der geschätzten intravasalen KM-Konzentrationen erhöhen.

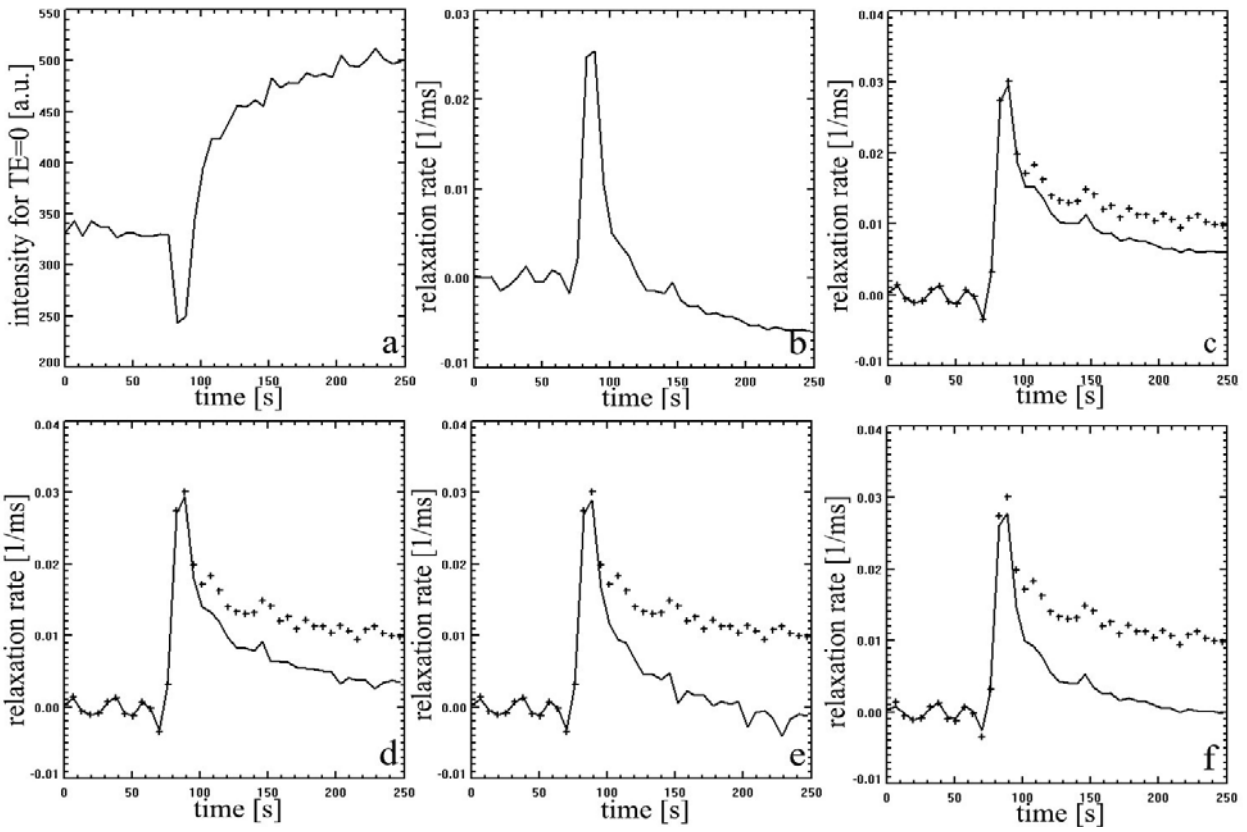

Abbildung 46: Auf $T_E = 0$ extrapolierte Signalintensität (a) und berechnete R_2^*-Änderungen für verschiedene Korrekturansätze (Glioblastom-Rezidiv). b: nur aus Signalveränderung des zweiten Echos; Kreuze in c, d, e, f: aus der T_2^*-Schätzung auf Grundlage des Intensitätsverhältnisses beider Echos für jeden Zeitpunkt; Linien in c, d,

e: Korrektur nach Gleichung (62) unter der Annahme $r^*_{2,ext}$ = 0 (c), $r^*_{2,ext}$ = 3.5 (x = 2) (d), $r^*_{2,ext}$ = 10.25 (x = 3,5) (e); Linie in f: empirische Korrektur auf Grundlage der in a gezeigten Signalveränderung bei T_E = 0.

Es erweist sich, dass die „suszeptibilitätsabhängige Relaxivität“ r_2^* von der geometrischen Verteilung des KM in seiner Umgebung abhängt $r^*_{2,ves} \neq r^*_{2,ext}$ - anderenfalls müsste der Perfusionseffekt in der korrigierten Relaxationsrate $\Delta R^*_{2,corr}(t\ )$ verschwinden (Gleichung (62)).

Die durch interstitielles KM verursachte Dephasierung $r^*_{2,ext}$ sollte im Vergleich zu $r^*_{2,ves}$ klein sein, da das interstitiell aufgenommene KM viel feiner verteilt ist als dies für den Kapillarraum im Gewebe zutrifft. Dies dürfte zu geringeren Suszeptibilitätsdifferenzen führen. Diese Annahme wird durch unser Ergebnis, dass sich der Verlauf von $\Delta R^*_{2,corr}(t)$ nach der Korrektur gemäß Gleichung (62) nicht dramatisch ändert, untermauert.

Wenn zur Einstellung von $\Delta R^*_{2,corr}(t_{\max}) = 0$ am Ende der dynamischen Studie der Korrekturfaktor x „von Hand“ maximiert wird, reduziert sich die maximale Relaxationsrate während der Boluspassage um nicht mehr als ca. 30 %. Dafür musste $x \approx 3.5$ gewählt werden. Da die KM-Aufnahme in das Gewebe langsamer als die Boluspassage vonstatten geht, kann im Rahmen eines groben Vergleiches der Relaxivitätswerte zum Zeitpunkt der maximalen KM-Konzentration während der Boluspassage die interstitielle KM-Konzentration gegenüber der intravasalen vernachlässigt werden. Hieraus kann über die Division von Gleichung (62) durch Gleichung (60) die Relation $\frac{r^*_{2,ves} - r^*_{2,ext}}{r_2 + r^*_{2,ves}} > 0.7$ gefolgert werden. Für die hier angewandten Messbedingungen ergibt sich daraus unter Verwendung von $r^*_{2,ext} > 0$ die Beziehung $r^*_{2,ves} > 2.3 * r_2 = 12.5\ mM^{-1}s^{-1}$.

Da die Korrektur gemäß Gleichung (60) nicht zu negativen Werten von $\Delta R^*_{2,corr}(t)$ führt, gilt die Relation (64)

$$r^*_{2,ves} > r^*_{2,ext} \qquad (64)$$

Der wie oben beschrieben bestimmte x-Wert von 3.5 für maximale Korrektur erlaubt die Abschätzung einer Obergrenze für $r^*_{2,ext}$ von ca. 15 $mM^{-1}s^{-1}$ in unserem Experiment. Damit kann die oben angegebene Größenbeziehung zu Relation (65) verschärft werden:

$$r^*_{2,ves} > 15\ mM^{-1}s^{-1} \qquad (65)$$

Der empirische Ansatz $C_{ves}(t_{late}) = 0$ führt zu einer Überbewertung der durch interstitielles KM bedingten T_2^*-Verkürzung. Daraus resultiert eine Unterschätzung der korrigierten Konzentrationswerte um nicht mehr als ca. 10 % bei schnell und stark aufladenden Läsionen (abgeleitet aus dem Verhältnis zwischen der KM-Konzentration im Peak und nach der zweiten Passage (second pass) an unter unseren Messbedingungen gewonnenen AIF-Kurven – vgl. Abbildung 45).

Das Bestimmtheitsmaß zwischen dem invasiv gemessenen pO_2 und der maximalen Veränderung von ΔR_2^*, die sich in unseren Daten als gegenüber pO_2-Änderungen am sensitivsten erwies, verringert sich durch die Anwendung der Korrektur von 37 % auf 30 % (Tabelle 9, Abbildung 47). In der untersuchten Patientenpopulation führt die Korrektur also zu keiner Verbesserung der diagnostischen Wertigkeit.

Patient	pO_2 Median	ΔT_2^* [%]	ΔT_2^* [%] empirische Korrektur
HF1	33,1	83,1	78,5
SF1	5,35	55,4	49,2
JS1	5,3	38,5	30,6
KK1	20,7	40,1	34,4
KI1	24,2	55,4	42,2
MK1	24,1	70,7	45,0
MK3	26	57,3	49,0
SH1	14,2	53,0	43,5
SH3	4,9	51,4	47,1
SR1	2,9	59,2	51,1
SR3	2,2	42,1	12,3
Bestimmtheitsmaß		37,7%	30,1%
n		10	10

Tabelle 9: Perfusionsmessungen an pharyngealen Tumoren bei 8 Patienten vor Strahlentherapie (Patientenname xx1) und nach 50 Gy (Patientenname xx3). Berechnung der relativen T_2^*-Veränderung aus beiden Echos einer Doppelecho-FLASH-Sequenz mit und ohne empirische Elimination der durch interstitielles KM verursachten Dephasierung. Das Bestimmtheitsmaß r^2 wurde unter Annahme einer linearen Korrelation der Perfusionsparameter zum invasiv gemessenen Sauerstoffpartialdruck bestimmt.

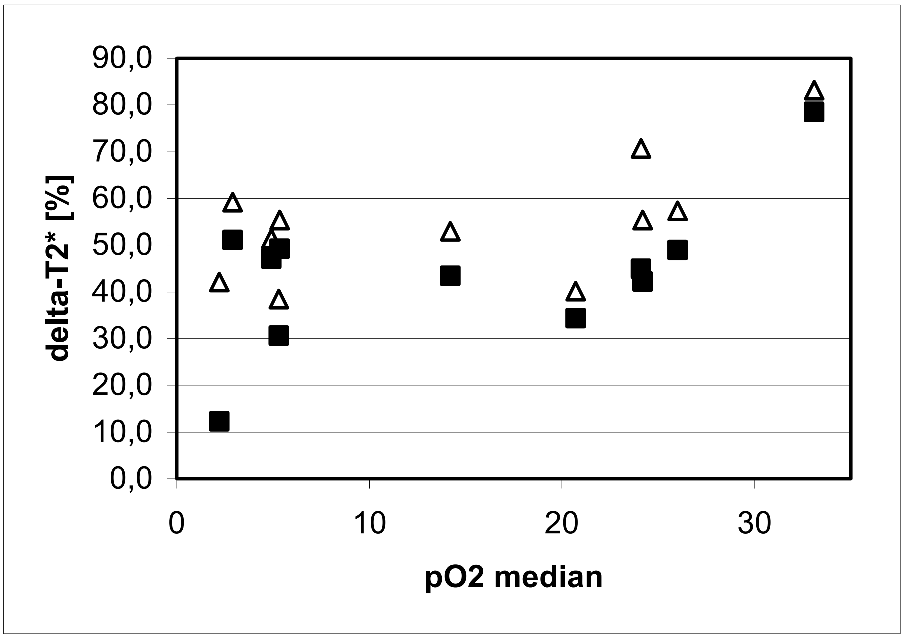

Abbildung 47: Abhängigkeit von ΔT_2^* vor (Δ) und nach (■) empirischer Bereinigung um den Beitrag durch interstitiell eingelagertes KM vom invasiv gemessenen Sauerstoffpartialdruck in pharyngealen Tumoren.

5.2.3 Diskussion

Die in diesem Abschnitt entwickelten Algorithmen sollen die intravasale KM-Konzentration aus der mittels Doppelecho-Sequenz gemessenen T_2^*-Veränderung während der Passage eines Bolus paramagnetischen Kontrastmittels extrahieren. Die angegebenen Daten zeigen die Plausibilität der Korrekturen. Methodisch korrektere Parameter sind allerdings keineswegs zwingend mit verbesserter diagnostischer Wertigkeit verbunden. Im Falle der untersuchten pharyngealen Tumoren führt die Tumor-Neoangiogenese zu verstärkter Perfusion, gleichzeitig tritt durch die lückenhaftere Endothel-Auskleidung dieser Gefäße vermehrt KM in das Interstitium über. Nach Anwendung nur der Doppelecho-Korrektur (d.h. lediglich nach Separation der T_2^*-Verkürzung) führen sowohl das intravasale als auch interstitielle KM zu ausgeprägterer T_2^*-Verkürzung; in diesem Fall verringert also die Separation der intravasalen KM-Konzentration den Messeffekt. Die Bestimmung methodisch korrekterer Perfusionsparameter ist jedoch bedeutsam bei differentialdiagnostischen Fragestellungen, bei denen KM-Aufnahme und Perfusion sich gegenläufig verhalten könnten (z. B. Meningeom vs. Neurinom im Kleinhirn-Brückenwinkel [78]) sowie für die Vergleichbarkeit über verschiedene Messbedingungen hinweg.

Die Funktionsfähigkeit der in Abschnitt 5.2 vorgestellten Algorithmen zeigt, dass die Abhängigkeit der Relaxationsrate ΔR_2^* von der KM-Konzentration von der geometrischen Verteilung des KM abhängt. Dies ist ein genauigkeitsbegrenzender Faktor bei der Bestimmung Arterieller Inputfunktionen.

5.3 MR-Mammographie

Etwa seit Beginn der 90er Jahre wird das Zeitverhalten der Aufnahme von paramagnetischem Kontrastmittel in den Interzellularraum für die Differentialdiagnose

maligner vs. benigner Läsionen in der weiblichen Brust eingesetzt [66, 92]. Die Methode stellt hierbei eine Komponente innerhalb einer Palette bildgebender Verfahren dar. Ausgangspunkt der Diagnostik sind nach wie vor die klassische Röntgen-Mammographie (ggfll. auch mit digitalen Detektoren und/oder computergestützter Diagnoseunterstützung) sowie die Sonographie. Als Indikationen zum Einsatz der MR-Mammographie (MRM) werden heute vorwiegend die Diagnostik bei Patientinnen mit Silikonprothesen bzw. Wiederaufbau-Plastiken bei hohem Rezidiv-Risiko, bei mammographisch eingeschränkter Beurteilbarkeit durch ausgeprägte Vernarbungen (z. B. nach Operationen oder Strahlentherapie), bei unklarem Tastbefund oder Risikopatienten mit stark Röntgenstrahlen absorbierenden („dichten“) Mammae (hoher Anteil an Drüsengewebe bei jüngeren Patientinnen) sowie zur Suche nach Brustwand-Infiltrationen bzw. weiteren Herden bei Karzinompatientinnen betrachtet [125]. Die Methode weist eine hohe Sensitivität auf, was sie zum Ausschluss maligner Tumore > 3 mm prädestiniert [93]. Ein zentrales Problem der MRM (wie auch der anderen Mamma-Diagnostik-Methoden) ist deren eingeschränkte Spezifität in der Unterscheidung maligner von benignen Befunden, die in der Literatur zwischen 21 % und 100 % angegeben wird [93, 25] und sowohl von Untersuchungsmethodik als auch Patientengut abhängt.

5.3.1 MR-Mammographie bei 0.5 Tesla und Übertragung statistischen Wissens zwischen unterschiedlichen Messbedingungen

Einige Parameter der in der MR-Mammographie angewandten halbquantitativen Modelle (und auch der Faktor A im pharmakologischen Modell nach Brix [17]) hängen von den konkreten Messbedingungen ab. Dies schränkt die Übertragbarkeit von mit solchen Modellen parametrisiertem statistischen Wissen auf modifizierte Untersuchungsbedingungen erheblich ein. Bei Inbetriebnahme neuer Hardware sind daher im Vorfeld entwickelte biostatistische Entscheidungskriterien nicht ohne Weiteres anwendbar. In der vorliegenden Arbeit war die Aufgabe zu lösen, die an einem 0,5-Tesla-Gerät etablierte MRM-Methodik auf ein 1,5-Tesla-Gerät so zu übertragen, dass einerseits schnell unter Nutzung des bei 0,5 Tesla gewonnenen statistischen Wissens klinisch relevante Diagnostik möglich wurde, andererseits die mit der neuen Hardware möglichen aussagekräftigeren Messroutinen (dynamische Erfassung eines ganzen Volumens in 2D- oder 3D-Technik) etabliert werden können.

Bei den Messungen mit 0.5 Tesla war der Signalintensitätsverlauf halbquantitativ als Anstieg der relativen Intensität gemäß einer Exponentialfunktion beschrieben worden. In den Messungen bei 1.5 Tesla wurde ein die renale KM-Ausscheidung berücksichtigender Faktor einbezogen [69] (Gleichung (72) in Abschnitt 5.3.2).

Im konkreten Fall konnte auf die retrospektive Analyse der kontrastmitteldynamischen Untersuchung von 95 histologisch gesicherten Mamma-Läsionen zurückgegriffen werden (vgl. Abschnitt 4.1).

Diese Daten wurden bei 0,5 Tesla an einem Philips Gyroscan T5 XPA gewonnen (Sequenzen siehe Abschnitt 4.2). Als Kontrastmittel war Gd-DTPA als Bolus von Hand injiziert worden (Magnevist – Schering AG; 0,1 mmol/kg Körpergewicht). Die gerätetechnische Limitation auf zwei dynamisch erfassbare Schichten beschränkte die Anwendbarkeit der Methode auf Läsionen, die bereits in den vor KM-Gabe aufgenommenen T_1-gewichteten STIR- und Volumen-Sequenzen erkennbar waren. Um Multifokalität bzw. langsam KM aufnehmende Läsionen zu erkennen, wurde auch nach

der dynamischen Messung ein T_1-gewichtetes Volumen akquiriert (k-Raum-Mitte etwa 11 Minuten p.i.). Der Signalintensitätsverlauf der dynamischen Messung wurde monoexponentiell modelliert (Gleichung (66), [77]):

$$S(t) = a * \left(1 - \exp\left(\frac{t_0 - t}{T_e}\right)\right) + b \tag{66}$$

mit a als dem absoluten Signalintensitätszuwachs zum Zeitpunkt $t = \infty$, b als der Signalintensität vor KM-Gabe, t_0 der Ankunftszeit des KM in der Läsion und T_e der Zeitkonstante des Enhancements.

Als bestes diagnostisches Kriterium war der maximale relative Anstieg A_{rel} ermittelt worden (Gleichung (67)):

$$A_{rel} = a / T_e \tag{67}$$

Der relative Signalunterschied der dynamisch untersuchten Läsionen in den Volumensequenzen vor und nach KM E_V enthält zusätzliche Information über eventuelle langsame KM-Aufnahme bzw. bereits beginnende Intensitätsabnahme durch Auswaschung [100]. Er wurde über eine logarithmische Linearkombination in das benutzte diagnostische Kriterium einbezogen (Gleichung (68)).

$$LK = \ln(E_V) + p_1 * \ln(A_{rel}) + p_2 \tag{68}$$

Der Parameter p_1 dieser Linearkombination war graphisch zu $p_1 = 1{,}8$ bestimmt worden. Die Schwelle zur Entscheidung benigne/maligne p_2 wurde unter der Maßgabe, dass keine falsch negativen Klassifikationen zugelassen sind (Spezifität = 100 %), zu $p_2 = 10{,}2$ festgelegt [77, 101].

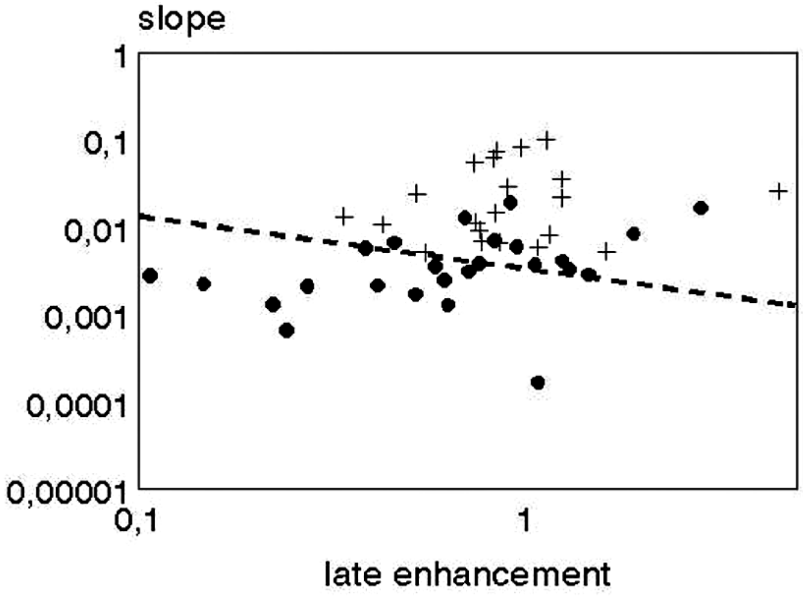

Abbildung 48: Graphische Festlegung der Parameter p_1 und p_2 in Gleichung (68) zur Diskrimination maligner (+) von benignen (•) Mamma-Läsionen anhand der bei 0,5 Tesla gemessenen Kontrastmitteldynamik (aus [77])

Mit diesem Vorgehen konnte eine Sensitivität von 17 richtig negativ klassifizierten aus 48 benignen Befunden (entspricht 35 %) erreicht werden [74]. Im Rahmen dieser Untrsuchungen konnten wir auch zeigen, dass bei Reduktion der zeitlichen Auflösung ausgehend von 11 s bis hinab zu 90 s kein wesentlicher Verlust an diagnostisch verwertbarer Information auftritt [77 bzw. mit höherer Fallzahl [74], dies steht nicht im Widerspruch zum Ergebnis von Henderson et al. [63], nach dem eine Zeitauflösung von mindestens 4 s erforderlich ist, um den dadurch bedingten Fehler in der Bestimmung des Blutvolumens kleiner als 10 % zu halten).

Als Randbedingung für die Messungen bei 1,5 Tesla war die gesamte Brust dynamisch zu erfassen. Um nach nur kurzer Zeitverzögerung klinisch relevante Ergebnisse liefern zu können, wurde die Abhängigkeit der Signalintensität von der KM-Konzentration mittels Messungen einer Verdünnungsreihe unter den alten (2D-FFE an zwei Schichten sowie 3D-FFE bei 0,5 Tesla) und neuen (3D-FLASH über 64 Schichten bei 1,5 Tesla) bestimmt. An ca. 85 Regionen in Mammae von 35 Patientinnen wurden die für Gleichung (68) relevanten Messpunkte auf die unter den bei 0,5 Tesla zu erwartenden Signalintensitäten umgerechnet. Der Anstieg der Linearkombination p_1 wurde aus dieser Transformation übernommen. Damit war die Anzahl der freien Parameter für die Festlegung des Klassifikators von 2 auf 1 reduziert, was die Größe der erforderlichen Stichprobe für eine gegebene Irrtumswahrscheinlichkeit senkt. Die Entscheidungsschwelle wurde anhand des mindestens sechsmonatigen Follow-up bzw. dem Ergebnis der histologischen Sicherung retrospektiv neu bestimmt, um den Einfluss methodischer Unsicherheiten dieser Strategie zu eliminieren (z. B. Abhängigkeit der Gewebe-T_1-Zeiten von der Feldstärke, Ungenauigkeiten der Flip-Winkel und damit der T_1-Wichtung der Sequenz, unterschiedliche Messdauer zur Bestimmung der Intensität 11 min p.i.).

5.3.2 Modell-Entwicklung und Spezifitäten von Modellparametern der dynamischen Kernspin-Mammographie

Parallel zu der sich an diese Phase anschließenden prospektiven klinischen Nutzung der dynamischen Messungen wurden Daten für eine weitere retrospektive Analyse gesammelt. Hierfür wurde folgendes Modell definiert:

Die Injektion eines KM-Bolus und dessen Verteilung im Blutpool wird als im Vergleich zur zeitlichen Auflösung der Messung als in unendlich kurzer Zeit abgeschlossen betrachtet. Die Elimination des KM aus dem Blut erfolgt mit einer zur KM-Konzentration proportionalen Ausscheidungsrate, also mit einem exponentiellen Abfall (diese Vereinfachung im Vergleich zum biexponentiellen Modell nach Weinmann et al. [178] (Gleichung (23)) erscheint gerechtfertigt, da die Ausscheidung aus dem Blutpool ein im Vergleich zur Aufnahme langsamer Prozess ist).

Der KM-Austausch zwischen Blut und Gewebe erfolgt richtungs-unabhängig und proportional zur Konzentrationsdifferenz. Er lässt sich damit mit dem 1. Fickschen Diffusionsgesetz beschreiben (Gleichung (24)). Über eine hinreichend kurze Distanz ist das Konzentrationsgefälle proportional zur Differenz der Konzentration im Gewebe und im Blut. Wenn nun der Konzentrationsverlauf im Blut nach Bolus-Injektion von KM als exponentiell abfallend modelliert wird, lässt sich Gleichung (24) umformulieren (Gleichung (69):

$$\frac{\partial C}{\partial t} = -D * A * \frac{\partial C}{\partial x} \sim -D * \left(C - C_{\max} * \exp\left(-\frac{t}{T} \right) \right) \quad (69)$$

Die Lösung dieser Differentialgleichung lautet (Gleichung (70)):

$$C(t) \sim \left(const + \frac{C_{\max} * D * T}{D * T - 1} * \exp\left(t * \left(D - \frac{1}{T} \right) \right) \right) * \exp(-D * t) \quad (70)$$

(Die Lösung wurde unter Benutzung von „math tools" [34] gefunden). Die Konstante *const* ergibt sich aus der Randbedingung C(0) = 0 (das Gewebe ist zu Beginn der Injektion kontrastmittelfrei) zu (71):

$$const = C_{\max} * \frac{D * T}{1 - D * T} \quad (71)$$

Die KM-Konzentration wird als hinreichend proportional zur Änderung der Relaxationsrate R_1 betrachtet (Gleichung (19), Abschnitt 2.2.4). Weiterhin darf unter gewissen Messbedingungen die relative Änderung der Signalintensität als proportional zur Änderung der Relaxationsrate betrachtet werden (Simulation in Abbildung 43, Abschnitt 3.2.3, vgl. auch Abschnitt 5.2.1). Mit den Abkürzungen $T_w = \frac{1}{1/T - D} = \frac{T}{1 - D * T}$ und $T_e = 1/D$ lässt sich damit die relative Änderung der Signalintensität als Gleichung (72) formulieren [69]:

$$\frac{S - S_0}{S_0} = a * \exp\left(\frac{t_0 - t}{T_e} \right) * \left\{ 1 - \exp\left(\frac{t_0 - t}{T_w} \right) \right\} \quad (72)$$

a ist hier ein Proportionalitätsfaktor, der außer von C_{max}, D, T und den Messbedingungen auch von der Fläche der Gefäßwände und deren Permeabilität abhängt (t_0 verallgemeinert lediglich die Zeitachse auf beliebige Ankunftszeit des KM im Gewebe).

Diese Modellfunktion steht in gewisser Beziehung zum von Brix et al. entwickelten Modell [17] (Gleichung (30), Abschnitt 3.1.1). Letzteres vereinfacht sich für eine extrem kurze Infusionsdauer τ, indem alle t' enthaltenden Terme zu Konstanten werden (Gleichung (73)

$$\frac{S}{S_0} = 1 + B * \{ \exp(-k_{21} * t) - C * \exp(-k_{el} * t) \} \quad (73)$$

mit B und C als von den Mess- und Injektionsbedingungen sowie k_{21} und K_{el} abhängigen Konstanten). Damit entsprechen $1/T_e = D$ ziemlich genau der Austauschrate zwischen Blut und Läsion k_{21} und $1/T_w = 1/T - D$ in hinreichender Näherung für nicht zu große t der Eliminationsrate des KM aus dem Blut-Pool k_{el} im Brix-Modell. Die multiplikative Anbindung des Auswaschterms in Gleichung (72)

vermeidet die in Gleichung (73) mögliche, jedoch physikalisch unplausible[10] Konstellation $S/S_0 < 1$.

Als diagnostisch relevante Parameter erwiesen sich der relative asymptotische Signalzuwachs a und der maximale relative Anstieg a/T_e. Dieser repräsentiert eine Kombination der physiologischen Eigenschaften „KM-Austauschrate D“ und „Diffusionsfläche“. Der KM-Eliminations-Term spielte erwartungsgemäß für die Mamma-Diagnostik keine Rolle, da er eher von mamma-unabhängigen physiologischen Faktoren wie z. B. der Nierenfunktion abhängen dürfte (Tabelle 10). Dessen ungeachtet ist er für gute Kurvenanpassung zumindest im Fall schnell KM aufnehmender Läsionen erforderlich.

Parameter	Sensitivität	Spezifität	negativer Vorhersagewert	positiver Vorhersagewert
rel. Asympt. Signalzuwachs	100	70	100	24
rel. Anstieg	100	76	100	48
Zeitkonstante der Aufladung	100	61	100	37
Zeitkonstante der Auswaschung	53	67	73	13
Linearkombination aus rel. Asympt. Signalzuwachs und Zeitkonstante der Aufladung	100	75	100	47
Linearkombination aus rel. Anstieg und Spätaufladung (monoexponentielles Modell)	100	64	100	38

Tabelle 10: Diagnostische Wertigkeit (in %) verschiedener Parameter des biexponentiellen Modells. Die Entscheidungsschwelle wurde so gewählt, dass keine falsch negativen Klassifikationen auftraten (nicht möglich für die Zeitkonstante der Auswaschung).

Die logarithmische Linearkombination aus relativem asymptotischen Signalzuwachs und Zeitkonstante der Aufladung des monoexponentiellen Modells wurde graphisch bestimmt (Abbildung 49). Ihre diagnostische Wertigkeit unterscheidet sich nicht wesentlich von der des relativen Anstiegs, da dieser ebenfalls eine (wenn auch nicht lineare) Kombination dieser Parameter darstellt.

[10] Signalverluste durch Verkürzung von T_2^* werden von dem Modell nicht behandelt

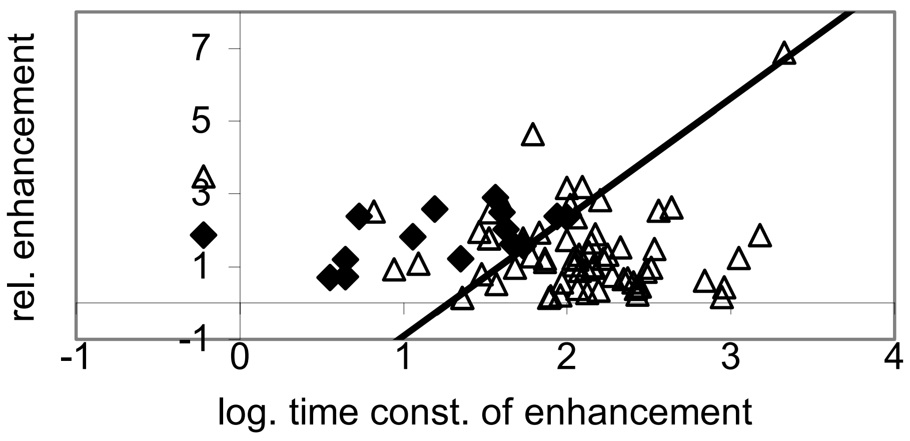

Abbildung 49: Graphische Bestimmung der bivariaten Entscheidungsschwelle. Klassifikation als maligne (◆) bzw. benigne (Δ) gemäß Histologie oder Follow-up. (aus [69]).

Die prospektive Anwendung des monoexponentiellen Models unter den Messbedingungen bei 1,5 Tesla führt auf plausible Ergebnisse. Die Spezifität weicht nicht wesentlich von der bei 0,5 Tesla erreichten ab. Damit kann der Transfer des statistischen Wissens als erfolgreich betrachtet werden. Natürlich sind die aus den beiden Ansätzen gewonnenen diagnostischen Informationen miteinander hoch korreliert (Abbildung 50). Die verbleibenden Abweichungen von einem determinierten Zusammenhang dürften im Wesentlichen durch die geringere Zahl von bei der Anpassung des monoexponentiellen Modells benutzten Messpunkten (auf Grund der verringerten zeitlichen Auflösung nur 4 Punkte gegenüber 19 Punkten bei 0,5 Tesla) sowie dadurch erklärbar sein, dass der Ansatz der Transformation über Verdünnungsreihe die Abhängigkeit der Gewebe-T_1-Relaxationszeiten von der Feldstärke nicht berücksichtigt und damit Nichtlinearitäten in der Beziehung zwischen KM-Konzentration bzw. ΔR_1 und der Signalintensität in unterschiedlichem Maße wirksam werden.

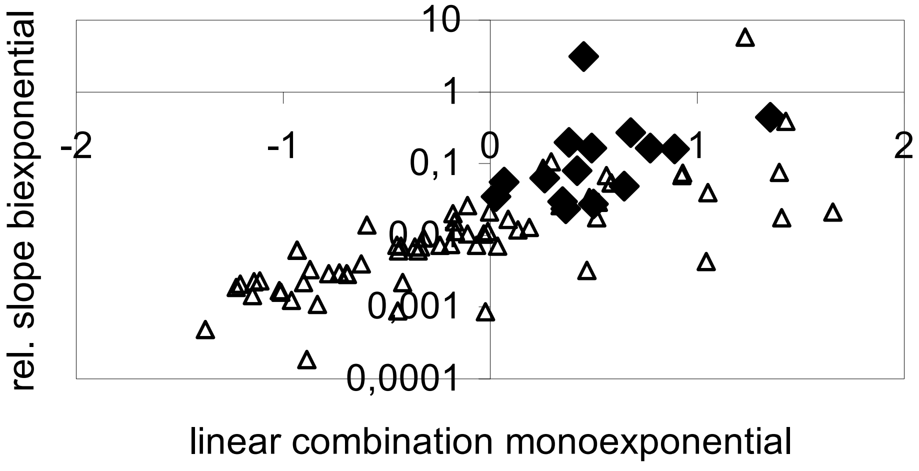

Abbildung 50: Vergleich der Linearkombination aus relativem Anstieg des mono-exponentiellen Modells und Spätaufladung mit dem relativen Anstieg des biexponentiellen Modells. Die Daten wurden bei 1,5 Tesla gewonnen. Klassifikation als maligne (◆) bzw. benigne (Δ) gemäß Histologie oder Follow-up. (aus [69]).

Zumindest für die im Rahmen dieser Arbeit gemessenen Daten erwies es sich als ausreichend, den KM-Austausch zwischen Blut und Gewebe als richtungsunabhängigen Prozess zu beschreiben. Die selbe Annahme liegt auch dem Modell von Brix et al. zu Grunde [17, 84]. Das in der Literatur qualitativ als Zeichen für Malignität beschriebene „Wash-out“-Phänomen lässt sich im benutzten Modell (Gleichung (72)) mit einer hinreichend kurzen Zeitkonstante T_e abbilden. Ein u. U. die KM-Aufnahme-Kurve überlagernder schmaler Peak während des first pass des KM-Bolus im Falle stark vaskularisierter Läsionen (Tumor-Shunts?) wurde aus Gründen des Signal-Rausch-Verhältnisses und der Stabilität der Kurvenanpassung nicht berücksichtigt.

5.4 Hirntumoren und -metastasen

In einer zwischen 1992 und 1998 untersuchten Patientengruppe mit vorwiegend extraaxialen Hirnläsionen wurden jeweils die Läsion und unbeteiligtes Parenchym der kontralateralen Hemisphäre untersucht [78]. Die gewählte Untersuchungstechnik limitierte die kontrastmitteldynamische Untersuchung auf nur eine Schicht. Die gleichzeitige Erfassung einer zuführenden großen Arterie war damit sehr erschwert. Da die KM-Injektionsbedingungen bezüglich Menge pro Körpergewicht sowie Fluss relativ konstant gehalten werden konnten, wurde auf die Messung einer arteriellen Inputfunktion verzichtet.

Bei der Modellierung des Konzentrations-Zeit-Verlaufes setzte der Autor eine intravasale Gleichgewichtskonzentration als Konstante an, die nach einem durch Kurvenanpassung festzulegenden Abbruchzeitpunkt T_a die Gamma-Variate-Funktion ersetzt (Gleichung (74), [78])

$$\begin{aligned} C(t) &= A * t^B * \exp(-t/C) \qquad && t \le T_a \\ C(t) &= D && t > T_a \end{aligned} \tag{74}$$

(vgl. Abschnitt 3.1.3). Eine Normalisierung der KM-Aufnahme- und Perfusionsparameter auf die Gegenseite führte nicht zu einer Verbesserung der diagnostischen Aussagekraft. Das bestätigt, dass die Injektionsbedingungen hinreichend konstant gehalten werden konnten. Die potenzielle Möglichkeit, interindividuelle Streuungen der Perfusionsbedingungen in normalem Parenchym aus den Messungen in den pathologischen Regionen zu eliminieren, wurde offenbar durch die dadurch zusätzlich in das Ergebnis eingehenden Messfehler maskiert (gemäß Gleichung (95) ist der relative Fehler eines Quotienten die Summe der relativen Fehler von Dividend und Divisor).

Aus den in Tabelle 11 dargestellten Ergebnissen lässt sich ableiten, dass durch Doppelechomessungen diagnostisch relevante Information gewonnen werden kann. Beispielsweise weist bei gering perfundierten Läsionen im Kleinhirn-Brückenwinkel (Differentialdiagnose Meningeom / Neurinom) eine langsamere KM-Aufnahme (korrigierte Zeitkonstante im 1. Echo > 10,5 s) auf ein Neurinom hin. Bei der Differentiation zwischen Lymphom und Metastasen wird zwar eine deutliche Überlappung der Parameter beobachtet, jedoch deuten eine relative Signalzunahme bei $T_E = 0$ von weniger als 1,2 oder eine MTT > 0,04 auf Metastasen hin.

Im Rahmen der durch die geringe Stichprobengröße bedingten statistischen Unsicherheit lässt sich für diese Fragestellungen eine Erkennungsrate (Sensitivität = Spezifität) in der Größenordung von 75 % abschätzen.

Läsion	Rel. Signal-zunahme[1]	Korrigierte Zeit-konstante im 1. Echo[2]	max. rel. Signal-verlust[3]	max. intravasale Konzen-tration[4]	Integral der GVF[5]	MTT[6]
Meningeom (gesamt)	1,18 (0,07)	7,05 (8,40)	0,17 (0,13)	0,018 (0,013)	0,065 (0,055)	20,3 (5,3)
Neurinom	1,27 (0,07)	15,35 (5,8)	0,04 (0,037)	0,007 (0,003)	0,028 (0,020)	17,8 (9,3)
Lymphom	1,20 (0,07)	8,2 (3,85)	0,04 (0,027)	0,008 (0,002)	0,023 (0,008)	12,89 (2,84)
Glomus tumor	1,179 (0,037)	0,5 (< 0,005)	0,63 (0,14)	0,069 (0,02)	0,179 (0,044)	11,12 (4,07)
Glioblastom	1,11 (0,04)	7,00 (3,85)	0,11 (0,061)	0,0097 (0,004)	0,033 (0,018)	16,08 (6,16)
Metastase (gesamt)	1,105 (0,072)	6,15 (4,45)	0,093 (0,08)	0,0095 (0,008)	0,035 (0,03)	14,93 (7,32)
Metastase 1	1,06 (0,04)	7,3 (5,25)	0,050 (0,029)	0,005 (0,002)	0,019 (0,011)	14,52 (7,44)
Metastase 2	1,16 (0,07)	4,40 (2,10)	0,16 (0,099)	0,016 (0,009)	0,058 (0,036)	15,55 (7,49)

Tabelle 11: Mittelwerte und Standardabweichungen (in Klammern) von T_1- und T_2^*-abhängigen Parametern für verschiedene Typen von Hirnläsionen. [1] maximaler relativer Signalzuwachs im ersten Echo ohne Korrektur; [2] Zeitkonstante der exponentiellen Modellfunktion nach Extrapolation auf $T_E = 0$; [3] maximaler relativer Signalverlust im zweiten Echo ohne Korrektur, [4] Maximum der nach Gleichung (52) abgeleiteten Kurve der zur Konzentration proportionalen Relaxationsraten-Änderung ΔR_2; [5] Integral über eine an $\Delta R_2(t)$ angepasste Gamma-Variate-Funktion mit Berücksichtigung einer Gleichgewichtskonzentration gemäß Gleichung (74); [6] mean transit time gemäß dem gleichen Modell. Metastase 1: Metastasen von Bronchal- und gastrointestinalen Karzinomen, Metastase 2: Metastasen von Mamma-, Ovarial-, Nierenkarzinomen sowie von Melanomen.

5.5 Pharyngeale Tumoren

Im Rahmen einer von Baumann geleiteten Studie zur Tumorphysiologie von Pharynx-Tumoren (unter Zustimmung der Ethik-Kommission des Universitätsklinikums „Carl

Gustav Carus“ an der TU Dresden) [98] wurden neben der MR-tomographischen Bildgebung und Kontrastmitteldynamik weitere Untersuchungen zum Status der Tumoren und dessen Änderung unter Strahlentherapie bzw. kombinierter Radio-Chemotherapie durchgeführt. So stand als von der MRT unabhängige Größe der invasiv gemessene extrazelluläre Sauerstoff-Partialdruck im Tumorgewebe zur Verfügung. Dessen polarographische Messung hat sich als Goldstandard zur Erfassung der Tumoroxygenierung etabliert (viele weitere Methoden zur Bestimmung der radiobiologischen Hypoxie im weitesten Sinne, wie die Messung der Sauerstoffspannung, des interstitiellen Flüssigkeitsdruckes oder des vitalen Tumoranteiles korrelieren nicht mit den durch die pO_2-Sondemessung gewonnenen Daten [8]). Die Aktivität des Glukose-Stoffwechsels wurde mittels Positronen-Emissions-Tomographie (PET) beschrieben. Der Therapie-Response wurde durch die Veränderung der Stoffwechsel-Aktivität sowie durch Volumetrie des Tumors (ggflls. einschließlich seiner Metastasen) beschrieben. Im Rahmen der vorliegenden Arbeit wurden die gewonnenen Daten genutzt, um verschiedene Auswertestrategien MR-tomographischer Perfusionsmessungen bezüglich prognostischer Wertigkeit, methodischer Korrektheit, Fehleranfälligkeit, aber auch bezüglich ihrer Praktikabilität in der klinischen Routine zu bewerten.

5.5.1 Auswerte-Strategie für die MR-Daten

Aus Gründen des Signal-Rausch-Verhältnisses erfolgten alle Analysen der Zeitverläufe der Signalintensitäten bzw. daraus abgeleiteten Größen auf der Basis von Regions of Interest. Diese wurden innerhalb eines im Rahmen dieser Arbeit entwickelten Programms durch einen erfahrenen Radiologen (Kittner, vgl. [98]) definiert.

Der Zeitverlauf der Kontrastmittel-Aufnahme in das Interstitium wurde mit der in Abschnitt 5.3.2 abgeleiteten Gleichung (72) modelliert. Gemäß Abschnitt 5.2.1 und Abbildung 43 wurde in erster Näherung die relative Änderung der Signalintensität des ersten Echos der Doppelecho-FLASH-Sequenz und in zweiter Näherung deren Korrektur auf eine Echozeit von $T_E = 0$ als proportional zur interstitiellen KM-Konzentration behandelt.

Die intravasale KM-Konzentration wurde in erster Näherung als proportional zum relativen Signalverlust des zweiten Echos der Doppelecho-FLASH-Sequenz angenommen. Im Sinne einer für den klinischen Alltag möglichst robusten Quantifizierung wurde hier nur der maximale relative Signalverlust betrachtet. Unter der (bei KM aufnehmendem Gewebe grob verletzten) Annahme einer Konstanz von T_1 während der Boluspassage lässt sich als zweite Näherung T_2^* aus der relativen Signalintensität des zweiten Echos schätzen. Hierbei ergibt sich die Änderung der zur KM-Konzentration hinreichend proportionalen Relaxationsrate R_2^* (vgl. Abschnitt 2.2.4.4) gemäß Gleichung (75)

$$\frac{S}{S_0} = \exp\left(-T_E * \left(\frac{1}{T_2^*} - \frac{1}{T_{2,0}^*}\right)\right) = \exp\left(-T_E * \left(R_2 - R_{2,0}\right)\right) \qquad (75)$$

zu Gleichung (76)

$$\Delta R_2^* = \frac{\ln\left(S_0 / S\right)}{T_E} \tag{76}$$

(der Index 0 steht für die Baseline-Scans). In weiterer Näherung wurde ΔR_2^* gemäß dem in Abschnitt 5.1 vom Autor [78, 79, 76] gleichzeitig mit Miyati et al. [124] sowie Heiland et al. [59] entwickelten Ansatz aus den Intensitäten beider Echos geschätzt (Gleichung (52)) und damit vom Verhalten von T_1 entkoppelt.

Die nach beiden Ansätzen bestimmten Relaxationsraten-Änderungen (als zur KM-Konzentration proportionale Größen) wurden einer numerischen Entfaltung von der Arteriellen Inputfunktion mittels SVD-Algorithmus unterzogen (vgl. Abschnitt 3.1.3, Punkt 4).

Die T_2^*-Verkürzung durch interstitiell eingelagertes Kontrastmittel wurde empirisch wie vom Autor in [70] vorgeschlagen (vgl. Abschnitt 5.2) berücksichtigt.

An die nach diesen verschiedenen Korrekturen erhaltenen zur intravasalen KM-Konzentration proportionalen Größen $\Delta R_2^*(t)$ wurden Gamma-Variate-Funktionen gemäß Gleichung (74) und ihrer vereinfachten Form gemäß Gleichung (46) bzw. nach Entfaltung von der AIF gemäß Gleichung (74) ohne Abbruch-Zeitpunkt angepasst. Aus den Modellkurven wurden Parameter gemäß Indikator-Verdünnungs-Theorie (Abschnitt 3.1.2) abgeleitet. Aus Gleichung (46) ergibt sich hierbei der Flächeninhalt des den Bolusanteil (ohne Rezirkulation) beschreibenden vereinfachten Gamma-Variate-Funktion nach Gleichung (77)

$$A_{GVFsimplified} = \int_0^\infty A * \frac{t}{t_p^2} * \exp\left(-\frac{t}{t_p}\right) dt =$$
$$\left[\frac{-A * \left(t_p^2 + t_p * t\right)}{t_p^2} * \exp\left(-\frac{t}{t_p}\right)\right]_0^\infty = a \tag{77}$$

Die Mean Transit Time entspricht als erstes Moment der GVF dem Parameter t_1 (Gleichung (79)).

$$\int_0^\infty t * A * \frac{t}{t_p^2} * \exp\left(-\frac{t}{t_p}\right) dt =$$
$$\left[\frac{-A * \left(2 * t_p^3 + 2 * t_p^2 * t + t_p * t^2\right)}{t_p^2} * \exp\left(-\frac{t}{t_p}\right)\right]_0^\infty = a * t_p \tag{78}$$

$$MTT = \frac{\int_0^\infty t * A * \frac{t}{t_p^2} * \exp\left(-\frac{t}{t_p}\right) dt}{\int_0^\infty A * \frac{t}{t_p^2} * \exp\left(-\frac{t}{t_p}\right) dt} = \frac{a * t_p}{a} = t_p \qquad (79)$$

Mit a als dem regionalen Blutvolumen proportionaler Größe lässt sich eine dem regionalen Blutfluss proportionale Größe $rBF_{simplified}$ formulieren (Gleichung (80)).

$$rBF_{simplified} = \frac{a}{t_p} \qquad (80)$$

Voraussetzung hierfür sind hinreichend vergleichbare Injektionsbedingungen. Als robuste Methode wurde die Fläche unter $\Delta R_2^*(t)$ auch über numerische Integration ($\Sigma\ \Delta R_2^*(t) * \Delta t$) bestimmt.

Für die Gamma-Variate-Funktion gemäß Gleichung (74) ohne Abbruch-Zeitpunkt ergeben sich entsprechend die Gleichungen (81) (82)

$$\int_0^\infty A * t^B * \exp\left(-\frac{t}{C}\right) dt = \left[-A * t^{1+B} * \left(\frac{t}{C}\right)^{-1-B} * \Gamma\left(1+B, \frac{t}{C}\right)\right]_0^\infty \qquad (81)$$

$$\int_0^\infty t * A * t^B * \exp\left(-\frac{t}{C}\right) dt = \left[-A * t^{2+B} * \left(\frac{t}{C}\right)^{-2-B} * \Gamma\left(2+B, \frac{t}{C}\right)\right]_0^\infty \qquad (82)$$

Mit den Eigenschaften der unvollständigen Gamma-Funktion (Gleichungen (83) und (84)) sowie (85)

$$\lim_{x\to\infty} \Gamma(a,x) = 0 \qquad (83)$$

$$\Gamma(a,0) = \Gamma(a) \qquad (84)$$

$$\Gamma(x+1) = x * \Gamma(x) \qquad (85)$$

lassen sich die Fläche der GVF, die MTT und eine dem Blutfluss proportionale Größe rBF_{GVF} wie folgt formulieren Gleichungen (86), (88) und (89)):

$$A_{GVF} = \frac{A}{C^{1+B}} * \Gamma(1+B) \qquad (86)$$

$$\int_0^\infty t * A * \frac{t}{t_p^2} * \exp\left(-\frac{t}{t_p}\right) dt = \frac{A}{C^{2+B}} * \Gamma(2+B) \qquad (87)$$

$$MTT_{GVF} = \frac{C^{1+B}}{C^{2+B}} * \frac{\Gamma(2+B)}{\Gamma(1+B)} = C*(2+B) \tag{88}$$

$$rBF_{GVF} = \frac{A}{(2+B)*C^{2+B}} * \Gamma(1+B) \tag{89}$$

Die Integrale wurden mit Hilfe des „Integrators“ [185] berechnet.

Die beim Tumorwachstum zu beobachtende chaotische Gefäßstruktur des Kapillarbettes mit arterio-venösen Shunts wurde nach einem Ansatz des Autors [80]. berücksichtigt, indem $\Delta R_2^*(t)$ durch die Summe aus zwei Gamma-Variate-Funktionen modelliert wurde.

Zur Gewährleistung einer für die oben beschriebene methodische Fragestellung hinreichend homogenen Stichprobe wurden die Patienten mit der größten Tumorvolumen-Veränderung in positiver und negativer Richtung (relativ und absolut) von den weiteren Auswertungen ausgeschlossen (Initialen K-B und L-HP). Für die Kombination der MR-unabhängigen Parameter (Tumorvolumen gemäß MR und PET, Volumenreduktionen nach 50 Gy Strahlentherapie, pO_2, hypoxische Fraktion, SUV, Überlebenszeit) mit allen aus der Kontrastmitteldynamik abgeleiteten Parametern wurden lineare Regressionen berechnet. Zur Auswahl des geeignetsten Vergleichsparameters für die Bewertung der klinischen Relevanz der MR-Perfusionsparameter wurde das maximale, auf dem 5-%-Niveau signifikant von Null verschiedene Bestimmtheitsmaß dieser Regressionen bestimmt. Im Weiteren wurde bewertet, inwieweit die oben beschriebenen Korrekturen die Korrelationskoeffizienten zu diesem Vergleichsparameter beeinflussen.

5.5.2 Ergebnisse

Ergebnisse der Korrelationsanalysen zwischen KM-unabhängigen und kontrastmitteldynamischen MR-Parametern sind exemplarisch für die T_1-bezogenen Parameter in Tabelle 12 sowie für Perfusionsauswertungen direkt aus dem Intensitätsverlauf des 2. Echos Tabelle 13 bzw. aus den T_2^*-Schätzungen gemäß Gleichung (51) in Tabelle 14 dargestellt.

physiol. Parameter	S1 rel. Aufladung	S1 Zeitkonstante	S1 rel. Anstieg	S1DE rel. Aufladung	S1DE Zeitkonstante	S1DE rel. Anstieg
pO2 Median primär	17 / 0,02	17 / 0,00	17 / 0,01	17 / 0,00	17 / 0,02	17 / 0,02
pO2 Median primär + 50 Gy	24 / 0,02	24 / 0,01	24 / 0,01	24 / 0,00	24 / 0,02	24 / 0,00
pO2 hypoxisch primär	17 / 0,01	17 / 0,06	17 / 0,00	17 / 0,00	17 / 0,00	17 / 0,03
pO2 hypoxisch primär + 50 Gy	24 / 0,01	24 / 0,00	24 / 0,00	24 / 0,00	24 / 0,00	24 / 0,01
SUV mean primär	16 / 0,03	16 / 0,02	16 / 0,04	16 / 0,01	16 / 0,02	16 / 0,18
SUV mean primär + 50 Gy	24 / 0,03	24 / 0,15 *	24 / 0,13 *	24 / 0,05	24 / 0,03	24 / 0,19 **
SUV mean	30 / 0,02	30 / 0,13 *	30 / 0,13 *	30 / 0,05	30 / 0,04	30 / 0,21 **
Volumen MR primär	18 / 0,01	18 / 0,13	18 / 0,00	18 / 0,02	18 / 0,01	18 / 0,01
Volumen MR	39 / 0,00	39 / 0,03	39 / 0,01	39 / 0,02	39 / 0,00	39 / 0,00
Vol-Reduktion MR 50 Gy	12 / 0,01	12 / 0,06	12 / 0,00	12 / 0,15	12 / 0,00	12 / 0,08
Volumen PET primär	16 / 0,14	16 / 0,03	16 / 0,06	16 / 0,17	16 / 0,01	16 / 0,00
Volumen PET	31 / 0,06	31 / 0,03	31 / 0,09	31 / 0,11 *	31 / 0,00	31 / 0,00
Vol-Reduktion PET 50 Gy	11 / 0,12	11 / 0,00	11 / 0,11	11 / 0,18	11 / 0,00	11 / 0,36 *
Überlebenszeit	11 / 0,01	11 / 0,16	11 / 0,00	11 / 0,03	11 / 0,01	11 / 0,01

Tabelle 12: Stichprobenumfänge und Bestimmtheitsmaße der Korrelationen zwischen T_1-bezogenen MR-Parametern und sonstigen tumorphysiologischen Parametern. $R^2 \neq 0$ mit Irrtumswahrscheinlichkeit $\leq 0{,}1$ mit einem Stern, $\leq 0{,}05$ mit zwei Sternen gekennzeichnet.

Von den T_1-bezogenen MR-Parametern wird die höchste Korrelation mit dem mittleren Standard-Uptake-Value von FDG beobachtet (Tabelle 12). Die Elimination der T_2^*-Verkürzung (in der Tabelle mit „DE“ für „Doppelecho-Korrektur“ gekennzeichnet) führt zu einer Zunahme der Bestimmtheitsmaße bei abnehmender Irrtumswahrscheinlichkeit. Als relevantester MR-Parameter erscheint der relative Anstieg nach Doppelecho-Korrektur, d.h. der relative asymptotische Signalzuwachs geteilt durch die Zeitkonstante (a/T_e in Gleichung (72)).

physiol. Parameter<br>n / R²	S2<br>rel. Signalverlust	S2<br>Fluss	S2<br>MTT	S2<br>rBV	S2<br>rBV num.
pO2 Median<br>primär	17 / 0,09	17 / 0,06	17 / 0,05	17 / 0,19 *	17 / 0,07
pO2 Median<br>primär + 50 Gy	24 / 0,07	24 / 0,07	24 / 0,06	24 / 0,02	24 / 0,04
pO2 hypoxisch<br>primär	17 / 0,11	17 / 0,18	17 / 0,13	17 / 0,13	17 / 0,05
pO2 hypoxisch<br>primär + 50 Gy	24 / 0,10	24 / 0,21 **	24 / 0,15 *	24 / 0,02	24 / 0,04
SUV mean<br>primär	16 / 0,14	16 / 0,03	16 / 0,00	16 / 0,02	16 / 0,01
SUV mean<br>primär + 50 Gy	24 / 0,11	24 / 0,11	24 / 0,06	24 / 0,01	24 / 0,00
SUV mean	30 / 0,14 *	30 / 0,11 *	30 / 0,11 *	30 / 0,00	30 / 0,00
Volumen MR<br>primär	18 / 0,16	18 / 0,31 **	18 / 0,20 *	18 / 0,22 *	18 / 0,06
Volumen MR	39 / 0,01	39 / 0,02	39 / 0,04	39 / 0,02	39 / 0,01
Vol-Reduktion MR<br>50 Gy	12 / 0,25	12 / 0,08	12 / 0,03	12 / 0,02	12 / 0,10
Volumen PET<br>primär	16 / 0,04	16 / 0,09	16 / 0,09	16 / 0,05	16 / 0,03
Volumen PET	31 / 0,00	31 / 0,00	31 / 0,02	31 / 0,02	31 / 0,00
Vol-Reduktion PET<br>50 Gy	11 / 0,00	11 / 0,03	11 / 0,17	11 / 0,01	11 / 0,06
Überlebenszeit	11 / 0,39 *	11 / 0,13	11 / 0,12	11 / 0,15	11 / 0,47 **

Tabelle 13: Stichprobenumfänge und Bestimmtheitsmaße der Korrelationen zwischen aus dem Signalverlauf des 2. Echos abgeleiteten MR-Perfusionsparametern und sonstigen tumorphysiologischen Parametern. $R^2 \neq 0$ mit Irrtumswahrscheinlichkeit $\leq 0{,}1$ mit einem Stern, $\leq 0{,}05$ mit zwei Sternen gekennzeichnet.

Die Schätzung von ΔR_2^* gemäß Gleichung (52) (Doppelecho-Korrektur) erhöht deutlich die Plausibilität der Konzentrations-Zeit-Verläufe, indem die durch die Verletzung der Bedingung $T_1 = \text{const}$ verursachten negativen Konzentrationen nicht mehr auftreten (Abbildung 51).

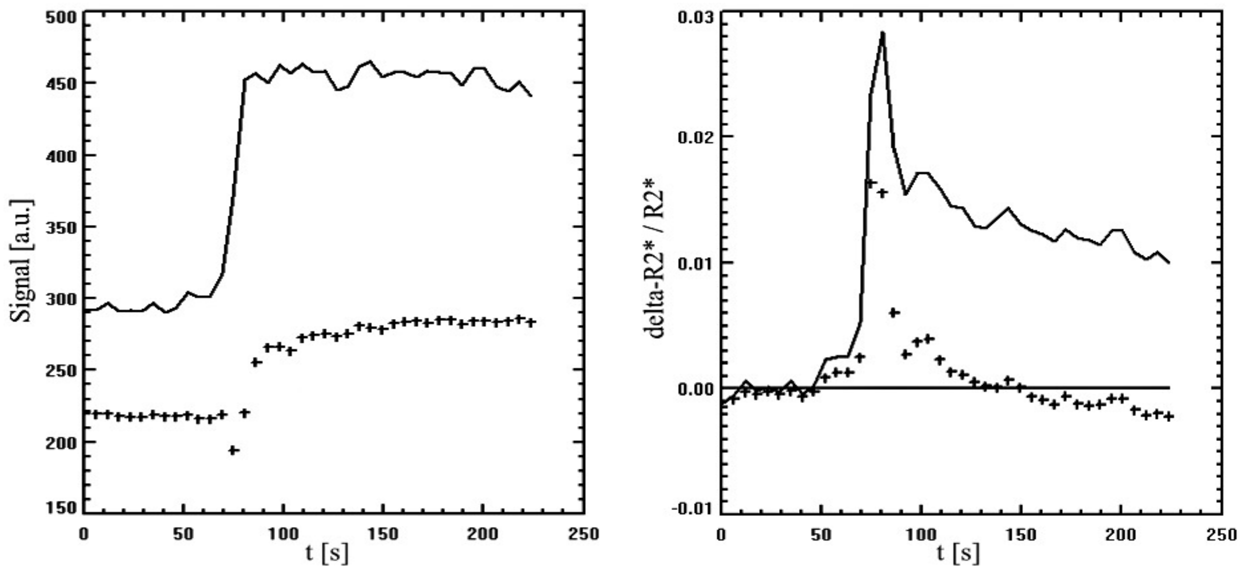

Abbildung 51: Doppelecho-Korrektur: Links Signalverlauf für $T_E = 15$ (+++) und für $T_E = 0$ (——), rechts ΔR_2^* aus Signal mit $T_E = 35$ (+++) und aus dem Quotienten der Signalverläufe zu $T_E = 15$ und $T_E = 35$ (——) (Patient KI vor Therapiebeginn)

Das höchste signifikant von Null verschiedene Bestimmtheitsmaß ergab sich zwischen der nach 50 Gy beobachteten Reduktion des MR-tomographisch bestimmten Tumorvolumens und der maximalen relativen Änderung des aus beiden Echos gemäß Gleichung (52) berechneten R_2^* (in Tabelle 14 grau unterlegt, N = 12). Gehäuft signifikante Korrelationen von MR-Perfusionsparametern finden sich auch zum mittleren Standard Uptake Value und zur hypoxischen Fraktion (N = 24) jeweils zeitnah zur MR-Untersuchung sowie prätherapeutisch zum MR-tomographisch bestimmten Tumorvolumen (N = 18). Zu weiteren physiologischen Parametern wurden nur sporadisch signifikant von Null verschiedene Korrelationskoeffizienten gefunden.

In Abbildung 53 sind die Bestimmtheitsmaße der Korrelationen aller geprüften MR-Perfusionsparameter gegen die vier genannten relevantesten physiologischen Parameter dargestellt (die Minimalwerte für statistische Signifikanz sind Tabelle 15 zu entnehmen).

Die stärkste Korrelation ist zwischen der MR-tomographisch bestimmten Volumenreduktion und Perfusionsparametern zu beobachten, die mit sehr einfachen und damit robusten Algorithmen berechnet wurden (maximale relative Änderung von R_2^*, numerisches Integral über ΔR_2*, beide nach Doppelecho-Korrektur). Nach Entfaltung von der Arteriellen Inputfunktion (mit und ohne Doppelecho-Korrektur) wurden die kleinsten Bestimmtheitsmaße gefunden. Die Bereinigung von ΔR_2*vom interstitiellen Anteil wirkt sich nicht positiv auf die Korrelationen zu unabhängigen Parametern aus. Der Anteil hypoxischen Tumorgewebes scheint wie auch das prätherapeutische Tumorvolumen bevorzugt mit Flussparametern zu korrelieren.

Die Korrelationen der aus den Kurvenanpassungen (rBV) und durch einfache numerische Integration (rBV num) gewonnenen Blutvolumen-Parameter unterscheiden sich erheblich voneinander. Die höhere Korrelation der „rBV-num"-Werte zur Volumenreduktion nach 50 Gy dürfte dabei Ausdruck des Einflusses der interstitiellen KM-Konzentration auf diesen Parameter sein – die relative Aufladung mit und ohne Doppelecho-Korrektur korreliert ebenfalls stark mit der Volumenreduktion (Tabelle 12).

Die Modellierung des intravasalen Konzentrations-Zeit-Verlaufes mit zwei Gamma-Variate-Funktionen scheint sich positiv auf die beobachteten Korrelationen auszuwirken – insbesondere Fluss-Berechnungen profitieren von diesem Modell.

physiol. Parameter<br>n / R²	S2DE<br>ΔR2*/R2*	S2DE<br>Fluss	S2DE<br>MTT	S2DE<br>rBV	S2DE<br>rBV num.
pO2 Median primär	17 / 0,01	17 / 0,13	17 / 0,03	17 / 0,12	17 / 0,02
pO2 Median primär + 50 Gy	24 / 0,00	24 / 0,12	24 / 0,05	24 / 0,01	24 / 0,00
pO2 hypoxisch primär	17 / 0,00	17 / 0,23 *	17 / 0,04	17 / 0,06	17 / 0,02
pO2 hypoxisch primär + 50 Gy	24 / 0,02	24 / 0,14 *	24 / 0,10	24 / 0,00	24 / 0,02
SUV mean primär	16 / 0,00	16 / 0,04	16 / 0,01	16 / 0,08	16 / 0,00
SUV mean primär + 50 Gy	24 / 0,05	24 / 0,14 *	24 / 0,06	24 / 0,00	24 / 0,02
SUV mean	30 / 0,06	30 / 0,15 **	30 / 0,05	30 / 0,01	30 / 0,06
Volumen MR primär	18 / 0,00	18 / 0,43 **	18 / 0,04	18 / 0,08	18 / 0,07
Volumen MR	39 / 0,03	39 / 0,07	39 / 0,05	39 / 0,01	39 / 0,00
Vol-Reduktion MR 50 Gy	12 / 0,61 **	12 / 0,02	12 / 0,01	12 / 0,00	12 / 0,52 **
Volumen PET primär	16 / 0,05	16 / 0,29 **	16 / 0,28 *	16 / 0,00	16 / 0,02
Volumen PET	31 / 0,10 *	31 / 0,01	31 / 0,05	31 / 0,00	31 / 0,07
Vol-Reduktion PET 50 Gy	11 / 0,00	11 / 0,00	11 / 0,23	11 / 0,21	11 / 0,01
Überlebenszeit	11 / 0,11	11 / 0,31	11 / 0,05	11 / 0,08	11 / 0,23

Tabelle 14: Stichprobenumfänge und Bestimmtheitsmaße der Korrelationen zwischen aus den Schätzungen von T_2^* aus dem Verhältnis der Signalintensitäten beider Echos abgeleiteten MR-Perfusionsparametern und sonstigen tumorphysiologischen Parametern. $R^2 \neq 0$ mit Irrtumswahrscheinlichkeit $\leq 0,1$ mit einem Stern, $\leq 0,05$ mit zwei Sternen gekennzeichnet.

Parameter	N	α	R²
SUV mean	30	0,05	0,145
SUV mean	30	0,1	0,100
pO2 hypoxisch (primär + 50 Gy)	24	0,05	0,187
pO2 hypoxisch (primär + 50 Gy)	24	0,1	0,128
Volumen MR (primär)	18	0,05	0,264
Volumen MR (primär)	18	0,1	0,179
Vol.-Reduktion MR (50 Gy)	12	0,05	0,451
Vol.-Reduktion MR (50 Gy)	12	0,1	0,299

Tabelle 15: Minimale Bestimmtheitsmaße für Irrtumswahrscheinlichkeit α zur Ablehnung der Null-Hypothese $R^2 = 0$ für die in Abbildung 53 dargestellten Korrelationen

Für die Bewertung von Korrekturansätzen ist das Ausmaß der Korrelation zwischen Blutperfusion und KM-Aufnahme in das Interstitium von Bedeutung. Sie dürfte am besten durch den Vergleich des relativen Anstiegs des auf $T_E = 0$ korrigierten Signals (S1DE) mit der relativen Änderung von R_2^* bewertbar sein, da dies zum einen die jeweils am stärksten mit unabhängigen physiologischen Parametern korrelierten Größen sind und zum anderen T_1- und T_2^*-Effekte in diesen Größen voneinander separiert sind. In der vorliegenden Stichprobe wird eine ausgeprägte Korrelation zwischen beiden Größen beobachtet (Abbildung 52).

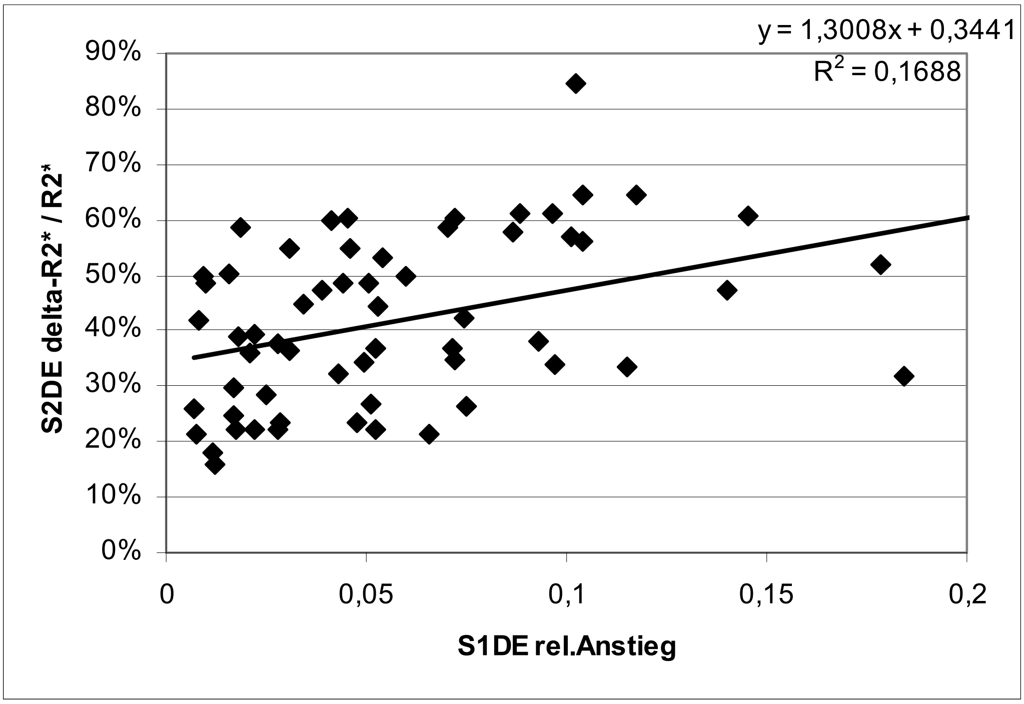

Abbildung 52: Korrelation zwischen relativem Anstieg des auf $T_E = 0$ korrigierten und damit nur von T_1-Änderungen beeinflussten Signals und der relativen Änderung der Spin-Spin-Relaxationsrate R_2^*.

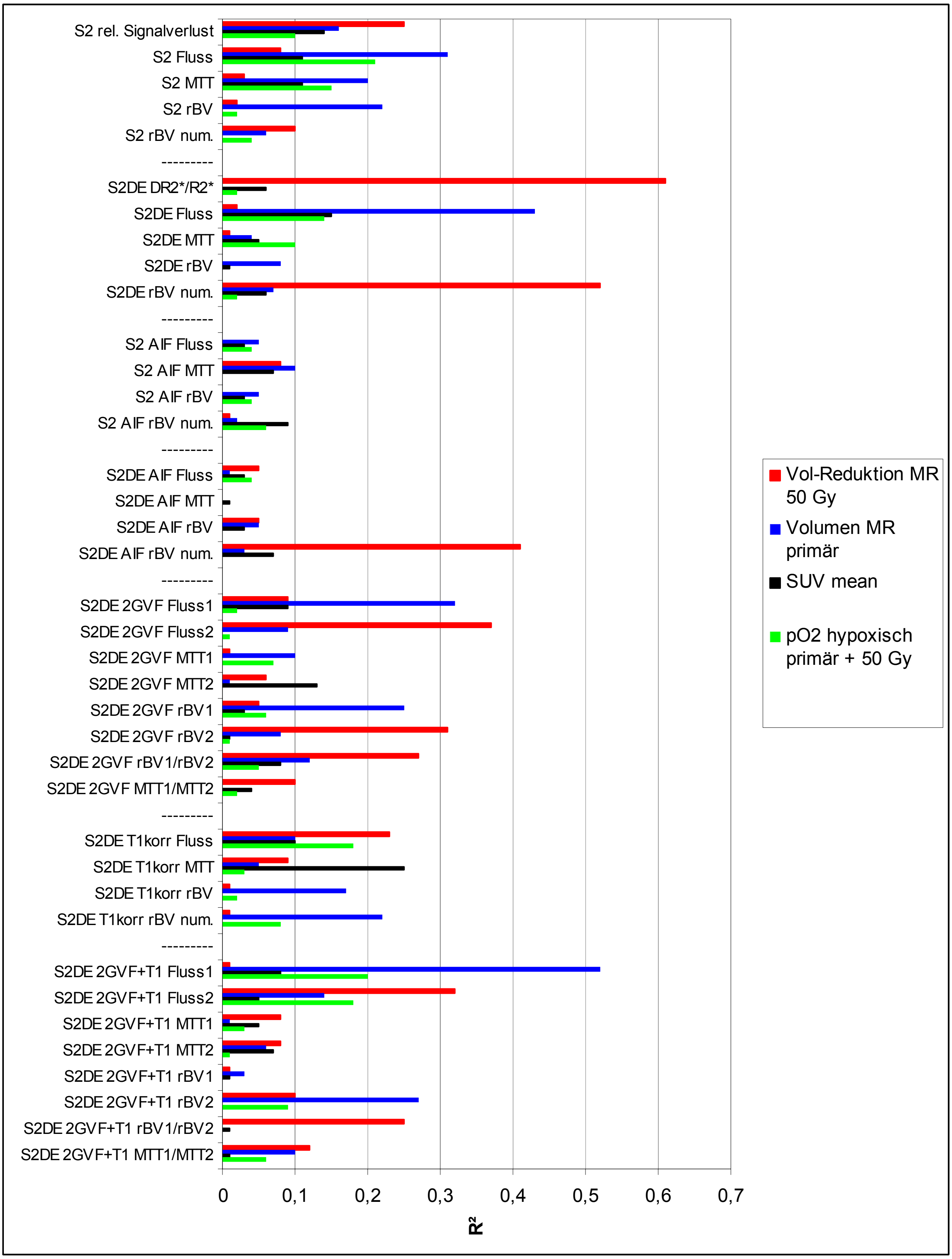

Abbildung 53: Bestimmtheitsmaße der Korrelation MR-tomographischer Perfusionsparameter mit anderen physiologischen Parametern

Die volumetrische Analyse zeigte mit zunehmendem Tumorvolumen einen intensiveren Glukosestoffwechsel (Abbildung 54). Eine direkte Beziehung zwischen

Glukosestoffwechsel-Aktivität und Sauerstoffpartialdruck lässt sich dagegen nicht nachweisen (Abbildung 55).

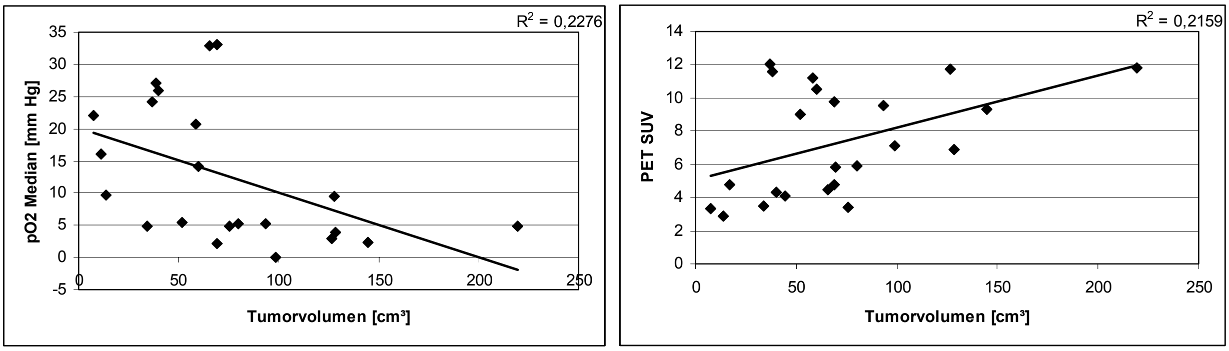

Abbildung 54: Korrelation zwischen Tumorvolumen und extrazellulärer Sauerstoff-sättigung (links) bzw. mittels PET gemessenem standard uptake value von ^{19}F-FDG (rechts). Beide Regressionskoeffizienten sind signifikant verschieden von 0 (t-Test, $p < 5$ %).

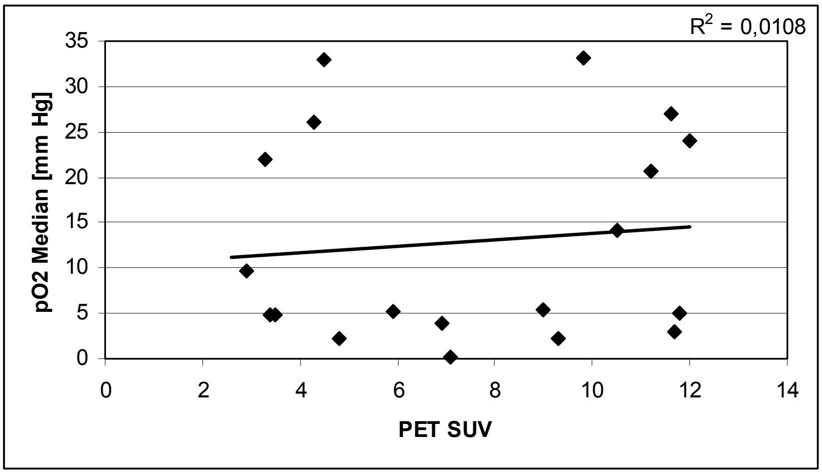

Abbildung 55: Beziehung zwischen Glukosestoffwechsel (PET-SUV) und Sauerstoff-partialdruck in pharyngealen Tumoren

Griftin et al. [49] wiesen neben weiteren Faktoren wie Lokalisation und Lymphknotenbefall der Tumorregression unter Therapie eine hohe prognostische Bedeutung zu. Die im Rahmen der vorliegenden Arbeit MR-tomographisch beobachtete Volumenänderung nach 50 Gy korreliert jedoch nur gering mit der Überlebenszeit der Patienten nach deren Eintritt in die Studie. Für die Prognose der Patienten scheinen das Tumor-Ausgangsvolumen $V_{0,MR}$ bzw. $V_{0,PET}$ bedeutsamer zu sein. Dieser Zusammenhang spiegelt sich auch in den Korrelationen der MR-Parameter zu den Volumen-Parametern sowie untereinander wider (Abbildung 56).

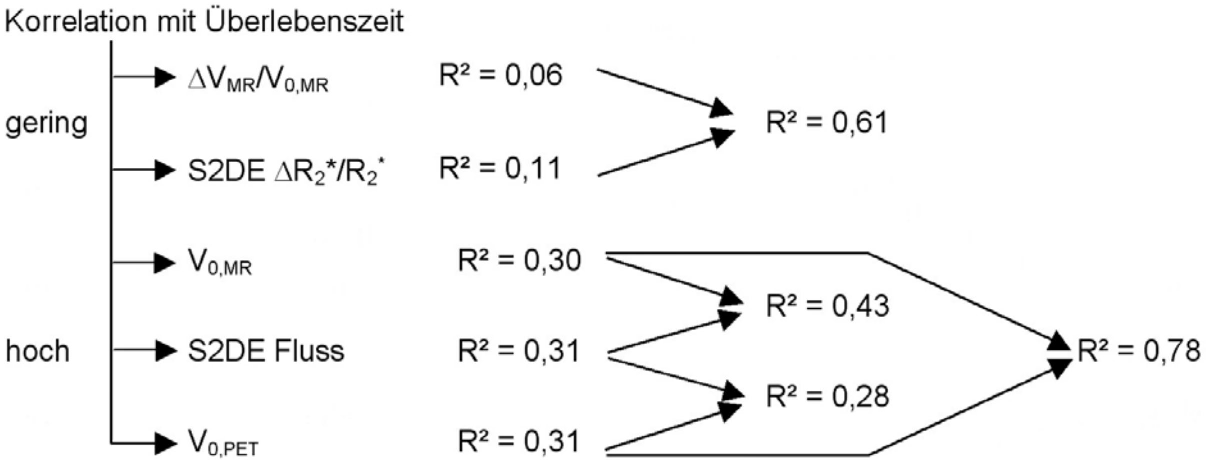

Abbildung 56: Bestimmtheitsmaße einiger MR-Parameter mit der Überlebenszeit ab Eintritt des Patienten in die Studie sowie untereinander (N = 9)

5.5.3 Diskussion

Die Eliminierung des Einflusses der T_1-Verkürzung auf die MR-tomographische Bestimmung von Perfusionsparametern stellt sich als der wichtigste aller analysierten Korrekturverfahren dar. Die Plausibilität der Zeitverläufe der intravaskulären Kontrastmittelkonzentration wird durch diesen Schritt erheblich verbessert (Elimination des Konzentrationsabfalls zu negativen Werten - Abbildung 51). Wegen der ausgeprägten Korrelation zwischen T_1-Verkürzung durch interstitiell aufgenommenes Kontrastmittel und T_2^*-bezogenen Perfusionsparametern sowie wegen der gegenläufigen Einflüsse einer Verkürzung von T_1 bzw. T_2^* auf die Signalintensität resultiert diese Korrektur auch in einer erhöhten Korrelation zwischen einigen MR-Parametern und unabhängigen Vergleichsparametern. Die Konsequenzen der hohen Korrelation zwischen KM-Aufnahme und Perfusion auf die Wirkung der Bereinigung von ΔR_2^* vom durch interstitielles KM bedingten Anteil verhält sich dagegen umgekehrt zur eben geführten Argumentation, da hier beide KM-Konzentrationen gleichsinnig wirken (vgl. Abschnitt 5.2)

Zumindest unter den gegebenen Messbedingungen überwiegen bei der Entfaltung der Schätzungen der intravasalen Kontrastmittel-Konzentration von der Arteriellen Inputfunktion die dabei zusätzlich in das Ergebnis eingeführten Messfehler deutlich den theoretisch zu erwartenden Nutzen. Die mit ca. 5 Sekunden für Perfusionsmessungen relativ geringe zeitliche Auflösung in dieser Studie und die damit verbundenen Probleme bei der Entfaltung mögen ein Grund dafür sein. Weitere Aspekte hierzu werden in Abschnitt 6.3 diskutiert.

Die Interpretation der tendenziell verbesserten Korrelationen zwischen Perfusion und sonstigen physiologischen Parametern bei der Modellierung der intravasalen KM-Konzentration mit zwei Gamma-Variate-Funktionen muss zurückhaltend erfolgen: Angesichts der Störanfälligkeit der Entfaltung von der AIF kann nicht bewertet werden, inwieweit eine zweite Gamma-Variate-Funktion lediglich Rezirkulationseffekte modelliert. Die vorliegenden Daten legen jedoch zumindest den Schluss nahe, dass im Zusammenwirken der Anpassung zweier GVFs und der Bereinigung des Anteils des interstitiellen KM an ΔR_2^* eine Korrelation zwischen (aus der ersten GVF bestimmtem) Fluss und prätherapeutischem Tumorvolumen deutlicher herausgearbeitet wird. Diese

Hypothese wird durch die Korrelationen dieses Flusses mit der Überlebenszeit der Patienten ($R^2 = 0,27$, vgl. mit $R^2 = 0,31$ für die Korrelation zwischen Überlebenszeit und Fluss aus $\Delta R_2^*/\Delta R_2^*$) gestützt.

Die vorliegenden Auswertungen zeigen, dass lokale Tumorvolumen-Reduktion und Überlebenszeit-Erwartung des Patienten deutlich voneinander verschieden sind. Für diese beiden prognostischen Aspekte treten unterschiedliche Perfusionsparameter in den Vordergrund: Während für die Volumenreduktion die maximale relative ΔR_2^*-Änderung (von regionalem Blutfluss und ~volumen bestimmt) bedeutsam ist, korreliert die Überlebenszeit stärker mit Flussparametern. Die in der Literatur (z. B. [48, 82] vertretene hohe prognostische Bedeutung der intratumoralen Sauerstoffsättigung konnte in der vorliegenden Studie nicht bestätigt werden. Es ist nicht auszuschließen, dass die Gründe hierfür methodischer bzw. messtechnischer Natur sind.

Signifikanzbetrachtungen zeigen, dass die diskutierten Tendenzen einer Absicherung durch eine größere Stichprobe bedürfen.

5.6 Darstellung von Blutgefäßen – Koronarangiographie

5.6.1 Ausgangssituation

Gegenwärtig sind die konventionelle Koronarangiographie sowie spezielle Computertomographie-Methoden Standardverfahren für die Bewertung von Stenosen bzw. Verkalkungen in den Koronararterien des Herzens. Hierbei verliert seit Aufkommen der Multislice-Detektoren für Spiral-Computertomographie die Elektronenstrahl-CT (Electron Beam CT – EBT) rasant an Bedeutung. Angesichts des hohen Potentials der Kernspintomographie für die Myokard-Perfusion, sowie kardiale Funktionsdiagnostik und Flussquantifizierung ohne Belastung des Patienten mit ionisierender Strahlung erscheint eine Weiterentwicklung dieser Methode im Interesse einer effizienten Herzdiagnostik möglichst in nur einer Sitzung auch für die Bildgebung der Koronararterien jedoch sinnvoll („One-stop shopping“) [103, 107, 153].

Zur Vermeidung von Atmungsartefakten können Navigatorecho-Sequenzen eingesetzt werden [166, 15]. Diese registrieren über die Messung eines „eindimensionalen Bildes“ senkrecht zum Zwerchfell dessen Position unmittelbar vor der Aufnahme von Daten für die eigentliche Bildgebung. Für die Bildrekonstruktion akzeptiert werden dann nur Beiträge, die innerhalb eines gewissen Fensters möglicher Zwerchfelllagen akquiriert wurden. Das Kontrast-/Rausch-Verhältnis, welches für die Sichtbarkeit der Koronararterien limitierend ist, kann durch die Gabe eines – im Idealfall intravasal verbleibenden – paramagnetischen Kontrastmittels verbessert werden [189]. So scheint die kontrastmittel-verstärkte MRT unter Benutzung von Navigator-Echos eine vielversprechende Methode zur Stenosebewertung in Koronararterien zu sein, obwohl noch Verbesserungen bei der Darstellung distaler Segmente erforderlich sind [155].

Es ist möglich, innerhalb eines Herzzyklus, d.h. zwischen zwei EKG-Triggerimpulsen, das zu messende Volumen mehrfach mittels RF anzuregen und mehrere Linien im k-Raum aufzuzeichnen. Um gleiche und gleichzeitig maximale Intensität aller dieser Linien zu gewährleisten (abgesehen vom durch die Ortskodierung unterschiedlichen Informationsgehalt), müssen die Flipwinkel einer entsprechend dichten Folge von RF-Impulsen in diesem Fall auf eine gewisse T_1-Relaxationszeit optimiert werden.

Prinzipiell relaxiert das Gewebe sowohl von Herzschlag zu Herzschlag als auch zwischen den RF-Impulsen innerhalb einer Triggerperiode. (Abbildung 57)

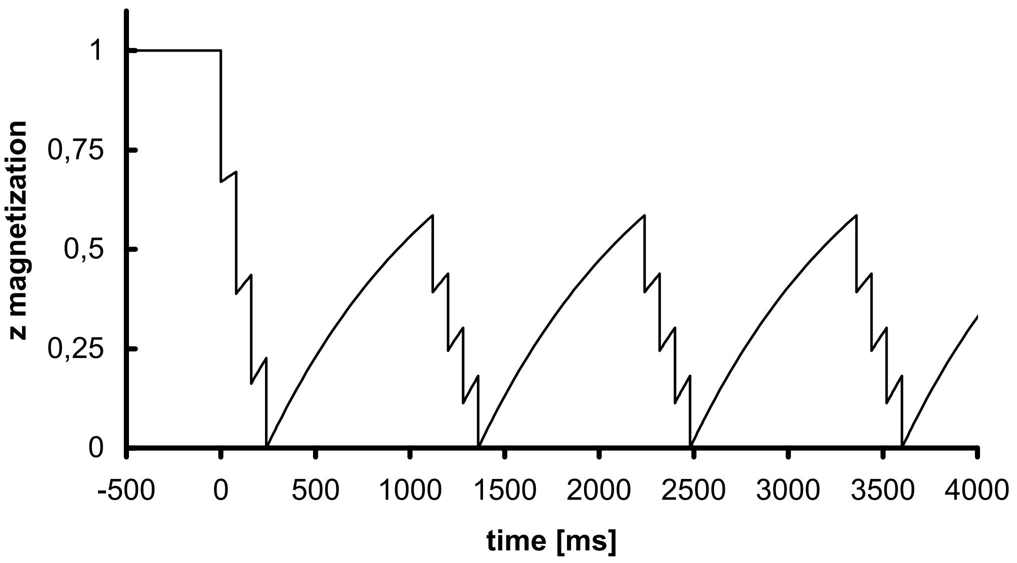

Abbildung 57: Schematische Darstellung des Zeitverlaufes der Nettomagnetisierung in einer Sequenz mit vier RF-Anregungen pro Herzzyklus. T_1-Relaxation erfolgt sowohl zwischen dem letzten RF-Impuls des n-ten Herzzyklus und dem ersten RF-Impuls des (n+1)-ten Herzzyklus als auch zwischen den RF-Impulsen innerhalb eines Herzzyklus. Darstellung berechnet für Zeitabstand zwischen zwei RF-Anregungen innerhalb eines Herzzyklus von 80 ms, T_R = 1120 ms, T_1 = 1000 ms, Flipwinkel α_1 = 48°, α_2 = 56°, α_3 = 68°, α_4 = 90°

Wenn die Zeit zwischen den Anregungen im Vergleich zur T_1-Zeit des Gewebes vernachlässigt werden kann, können die optimalen Flipwinkel ausgehend vom Flipwinkel des letzten Impulses innerhalb der Impulsfolge mittels Gleichung (90) rekursiv bestimmt werden [121].

$$\alpha_{n-1} = \tan^{-1}(\sin(\alpha_n)) \qquad (90)$$

Eine auf diese Art bestimmte Flipwinkel-Folge ist natürlich nur für eine bestimmte T_1-Relaxationszeit optimal. Da die Gabe eines Kontrastmittels den Wertebereich von im zu messenden Volumen auftretenden T_1-Zeiten zu kleineren Werten hin erweitert, erscheint die Vernachlässigbarkeit der T_1-Relaxation innerhalb der RF-Impulsfolge nicht mehr offensichtlich. Es wurde daher ermittelt, inwieweit die Art der Flipwinkel-Optimierung die Darstellung von Strukturen mit anderer als der zur Optimierung benutzten T_1-Zeit beeinflusst [67].

5.6.2 Optimierung der Messmethode

Der Einfluss der Flipwinkel-Folge auf die Darstellung fett-umscheideter kontrastmittelverstärkter Gefäße am Herzmuskel wurde vom Autor mittels Computersimulation analysiert [68].

Für eine jeweils gegebene T_1-Zeit wurden optimale Flipwinkelfolgen für eine 3D-FLASH-Sequenz mit 24 Partitionen, ein Zeitintervall von 7.5 ms zwischen den RF-Impulsen innerhalb eines Herzzyklus und eine Herzfrequenz von 60 Schlägen pro

Minute auf maximale Signalintensität bei gleicher Intensität pro Auslesung innerhalb eines Herzschlages bestimmt. Die T_1-Relaxation zwischen beliebigen RF-Impulsen wurde hierbei wie folgt berücksichtigt:

Ein RF-Anregungsimpuls i mit einem Flipwinkel α_i verändert die Nettomagnetisierung in z-Richtung von $M_{z,i}$ zu $M_{z,i,postRF}$ gemäß Gleichung (91)

$$M_{z,i,postRF} = M_{z,i} * \cos(\alpha_i) \quad (91)$$

Zur Berücksichtigung der T_1-Relaxation innerhalb der RF-Impulsfolge wurde die Nettomagnetisierung vor der (n+1)-ten RF-Anregung $M_{z,i+1}$ zur Zeit t_{i+1} aus $M_{z,i,postRF}$ zur Zeit t_i und der seit der letzten Anregung verstrichenen Zeit $t_{i+1} - t_i$ berechnet (Gleichung (92))

$$M_{z,i+1} = M_{z,0} * \left(1 - \exp\left(-\frac{t_{i+1} - t_i}{T_1} + \ln\left(1 - \frac{M_{z,i,postRF}}{M_{z,0}}\right)\right)\right) \quad (92)$$

$M_{z,0}$ steht hierbei für die z-Magnetisierung nach vollständiger Relaxation. Gleichung (92) impliziert eine unendlich kurze Pulsdauer. Die Randbedingung gleicher Signalintensität für alle Auslesungen kann als Gleichung (93) formuliert werden.

$$M_{z,i+1} * \sin(\alpha_{i+1}) = M_{z,i} * \sin(\alpha_i) \quad (93)$$

Zu Vergleichszwecken wurden ebenso Optimierungen unter Vernachlässigung der Relaxation zwischen den RF-Impulsen innerhalb eines Herzzyklus vorgenommen. Die iterative Bestimmung wurde unter MS-Excel durchgeführt. Relative Intensitäten der Signale nach den einzelnen RF-Anregungen wurden für Gewebe verschiedener Relaxationszeiten an Flipwinkel-Folgen für verschiedene (nicht nur mit der Gewebe-T_1 identischen) Relaxationszeiten bestimmt.

Zur Bestimmung von Kontrast-/Rausch-Verhältnissen (CNR) wurde ein eindimensionales Model eines von Fett umgebenen Blutgefäßes auf dem Myokard definiert. Es besteht aus einer ein Pixel breiten „gefäßartigen“ Struktur mit einer kurzen (T_1 = 100 ms) und einer 11 Pixel breiten „muskelartigen“ Struktur mit einer längeren (T_1 = 500 ms) Spin-Gitter-Relaxationszeit, die durch ein signalfreies Pixel (spektral gesättigtes Fett) voneinander getrennt sind.

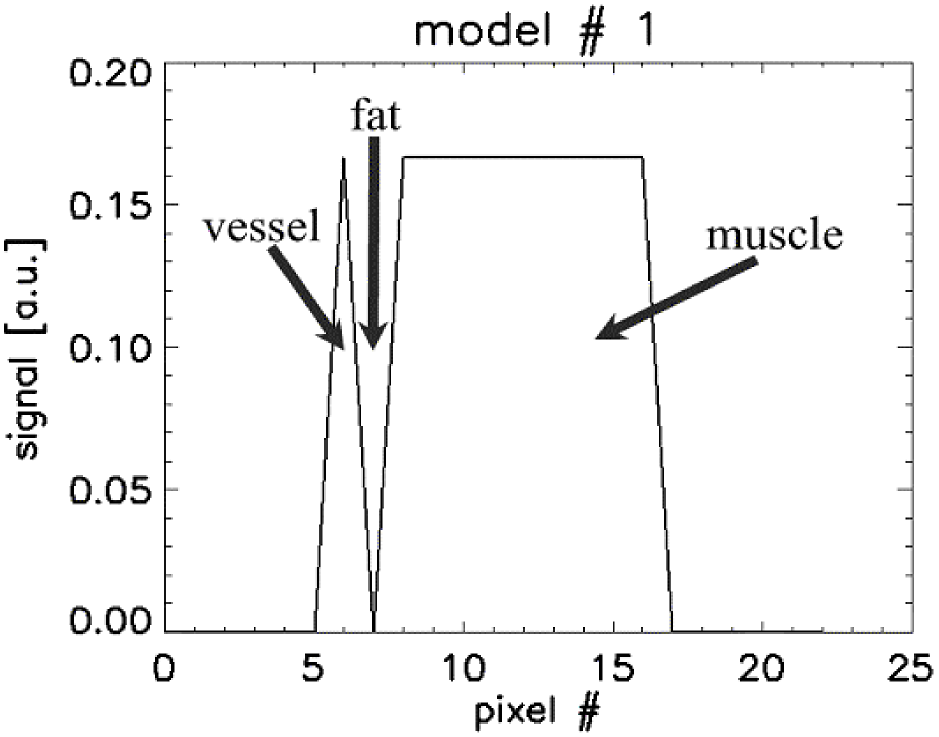

Abbildung 58: Computer-Modell eines von einer Fettscheide umgebenen kontrastmittelverstärkten Gefäßes auf Myocardium

Angaben zur Geometrie dieser Strukturen sind Tabelle 16 zu entnehmen.

	Gefäß	Lücke	Muskel
Modell 1	1	1	9
Modell 2	3	1	9
Modell 3	1	3	9
Modell 4	3	3	3

Tabelle 16: Geometrische Abmessungen (in Pixel) der in den Computersimulationen zu Kontrast-Rausch-Verhältnissen benutzten eindimensionalen Modelle.

Im Sinne gleicher Protonendichte wurden der „gefäßartigen“ und der „muskelartigen“ Struktur die gleiche Intensität zugewiesen. Als Rohdaten im k-Raum für diese Strukturen wurden deren Fourier-Transformierte benutzt. Diese wurden mit den wie oben beschrieben bestimmten relativen Intensitäten entsprechend den jeweils betrachteten T_1 für Sequenzoptimierung und Gewebe sowie Nummer des zugehörigen RF-Impulses multipliziert. Wie in der für reale Messungen benutzten Sequenz wurden die zentralen Werte im k-Raum den im Herzzyklus zuerst gemessenen Signalen zugeordnet. Nach Zusammenfassung der k-Raum-Komponenten beider Strukturen wurde in unterschiedlichem Maße unabhängig voneinander zu Real- und Imaginärteil Gauß-Rauschen hinzugefügt (Standard-Normalverteilung multipliziert mit „Rauschfaktoren“ zwischen 0.01 und 0.1 bei einer Skalierung der k-Raum-Mitte auf 10). Für jedes Signal-/Rausch-Verhältnis (SNR) wurden 1000 Stichproben gerechnet.

Das Kontrast-/Rausch-Verhältnis CNR wurde gemäß Gleichung (94) berechnet

$$CNR = \frac{S_{small} - S_{gap}}{sdev_{air}} \quad (94)$$

wobei S_{small} und S_{gap} die Signalintensitäten der schmalen Struktur bzw. der Lücke zwischen beiden Strukturen sowie $sdev_{air}$ die Standardabweichung über einige per Definition signalfreie Pixel des Modells darstellen. Diese Berechnungen wurden unter IDL (Research Systems, Inc.) ausgeführt.

Die Computersimulationen wurden durch Messungen an einem Kernspintomographen Siemens Magnetom Vision bei einer Feldstärke von 1.5 Tesla validiert. Im Tierversuch (Schweine) wurden 0.05 mmol/kg Körpergewicht des intravaskulär verbleibenden Kontrastmittels Gadomer-17 (Schering AG) sowie im Selbstversuch 0.3 mmol/kg Magnevist (Schering AG) über die gesamte Laufzeit der Navigatorecho-Sequenz hinweg infundiert.

5.6.3 Ergebnisse

5.6.3.1 Optimierung des Flipwinkels

Die Vernachlässigung der T_1-Relaxation innerhalb der RF-Impulsfolge führt zu einer Unterschätzung des optimalen Flipwinkels für späte Anregungen und damit wegen der vergleichsweise geringeren Sättigung zu zunehmender Signalintensität (Abbildung 59). Die Darstellung von Strukturen mit langer T_1-Zeit mittels einer auf eine kurze T_1-Zeit optimierten Sequenz führt zu abnehmender Intensität für die späten Auslesungen und damit beim gewählten Anregungsschema (Auffüllung des k-Raumes von der Mitte zu den Rändern) zu einem Tiefpass-ähnlichen Verhalten.

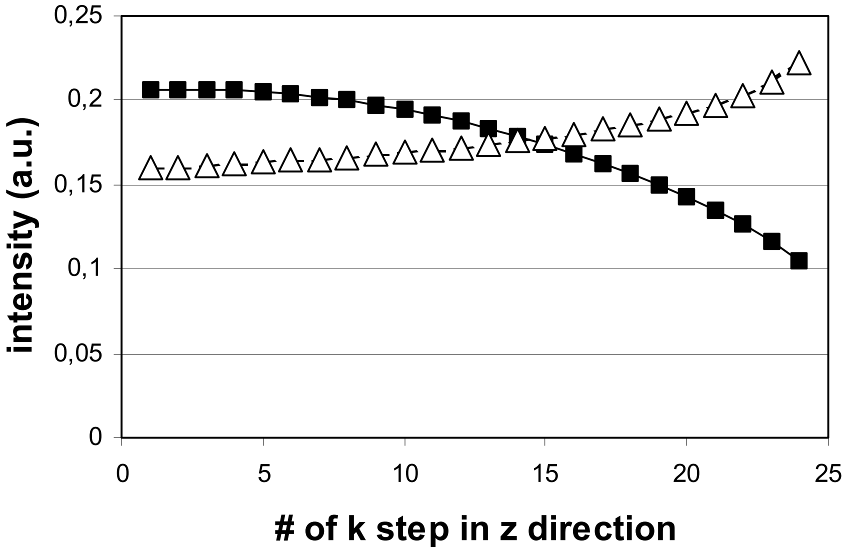

Abbildung 59: Abhängigkeit der Signalintensität einer Struktur mit einer Relaxationszeit T_1 = 500 ms von der Nummer des Anregungspulses in einer auf T_1 = 100 ms

optimierten Sequenz. ■ Berücksichtigung bzw. ∆ Vernachlässigung der T_1-Relaxation zwischen den RF-Impulsen während eines Herzzyklus.

5.6.3.2 Simulationen

Am Beispiel einer Simulation für eine auf T_1 = 50 ms optimierte Sequenz ist die beschriebene Tiefpass-Filterwirkung in Abbildung 60 dargestellt. Sie führt zu einer Verschmierung der „Fett"-Lücke zwischen dem simulieren Gefäß und Myokard. Anders als bei der Tiefpass-Filterung eines Bildes wird hier jedoch nur das Signal der hohen Ortsfrequenzen, nicht aber das Rauschen reduziert. Da die äußeren Bereiche im k-Raum in ihren Intensitäten unterrepräsentiert sind, tritt eine leichte „truncation artefact"-ähnliche Schwingung auf der breiten Struktur auf. Unter Vernachlässigung der Relaxation innerhalb eines Herzzyklus sind dagegen die zentralen Linien des k-Raumes unterrepräsentiert, was sich in einer geringeren mittleren Intensität der breiten Struktur sowie einem leicht kantenanhebenden Effekt ausdrückt (Abbildung 60 rechts). Auch für die schmale Struktur (T_1 = 100 ms) werden durch die auf T_1 = 50 ms optimierte Sequenz die äußeren Linien des k-Raumes überrepräsentiert, wenn der gröbere Optimierungsalgorithmus angewandt wird. Dies findet nach FFT in deutlichen „Füßen" an der Struktur seinen Niederschlag.

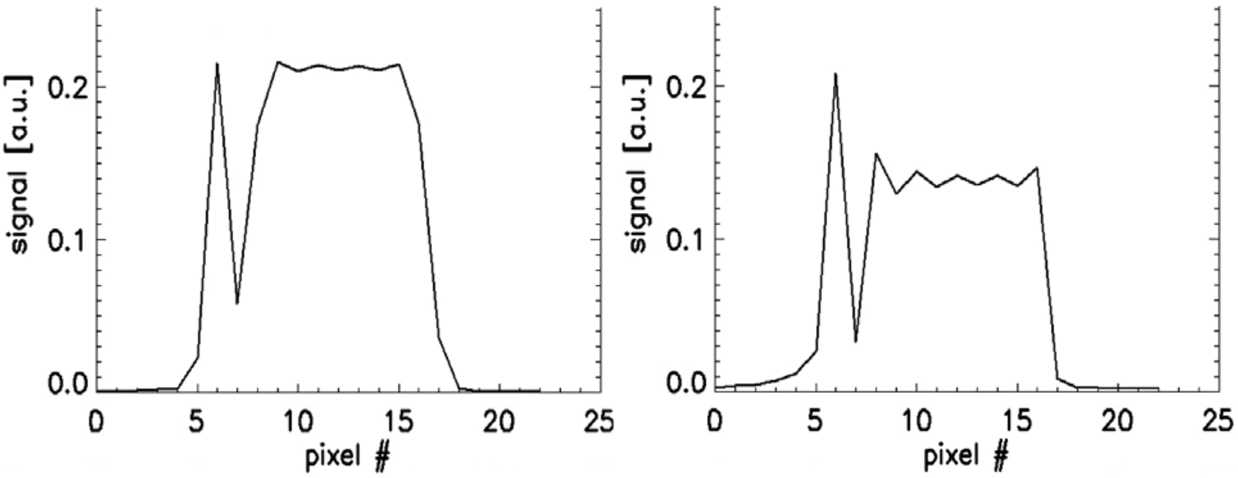

Abbildung 60: Simulation einer Messung mit einer auf T_1 = 50 ms optimierten Sequenz, links unter Berücksichtigung bzw. rechts unter Vernachlässigung der T_1-Relaxation zwischen den RF-Impulsen innerhalb eines Herzzyklus. Man beachte die unscharfe Darstellung der breiten Struktur (langes T_1) bei voller Optimierung (links) im Vergleich zur kantenanhebenden Wirkung (rechts).

Wenn eine schnell relaxierende Struktur mit einer auf lange T_1-Relaxationszeit optimierten Sequenz gemessen wird, führt dies zu einer Überbetonung der äußeren Bereiche des k-Raumes und damit letztlich zur Verschlechterung des Kontrastes zwischen der Struktur und der anschließenden Lücke. In Abbildung 61 ist die Verschmierung der schmalen Struktur durch eine auf 800 ms optimierte Sequenz dargestellt.

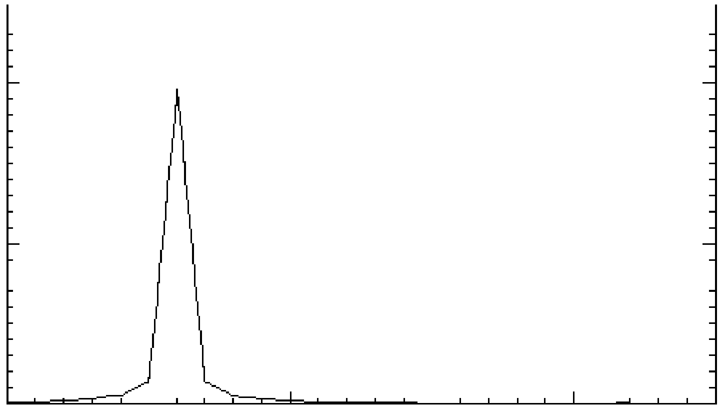

Abbildung 61: Simulation der Messung nur der Struktur mit T_1 = 100 ms mit einer auf T_1 = 800 ms optimierten Sequenz.

Das CNR, welches durch Hinzufügen von Gauss-Rauschen im k-Raum bestimmt wurde (siehe oben), ist selbst eine Zufallsgröße, deren Verteilung sich als relativ breit erweist. Solange nur realistische Rauschanteile addiert werden, führt die Berücksichtigung der T_1-Relaxation auch zwischen den RF-Impulsen innerhalb eines Herzzyklus im Mittel zu verbesserten CNR (Abbildung 63).

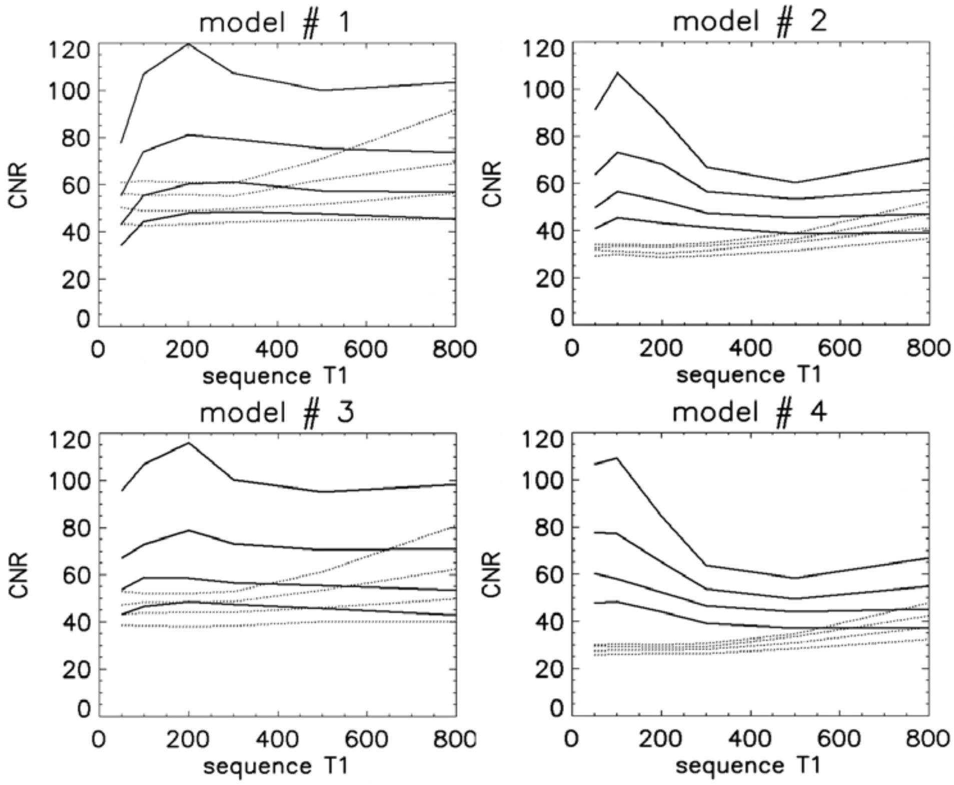

Abbildung 62: CNR in Abhängigkeit von der T_1-Relaxationszeit, auf die die Sequenz optimiert ist. Die T_1-Relaxation zwischen den RF-Impulsen innerhalb eines Herzzyklus wurde berücksichtigt (————) bzw. vernachlässigt (··········). Rauschfaktoren von oben nach unten: 0.01, 0.015, 0.02, 0.025. Modellgeometrien siehe Tabelle 16.

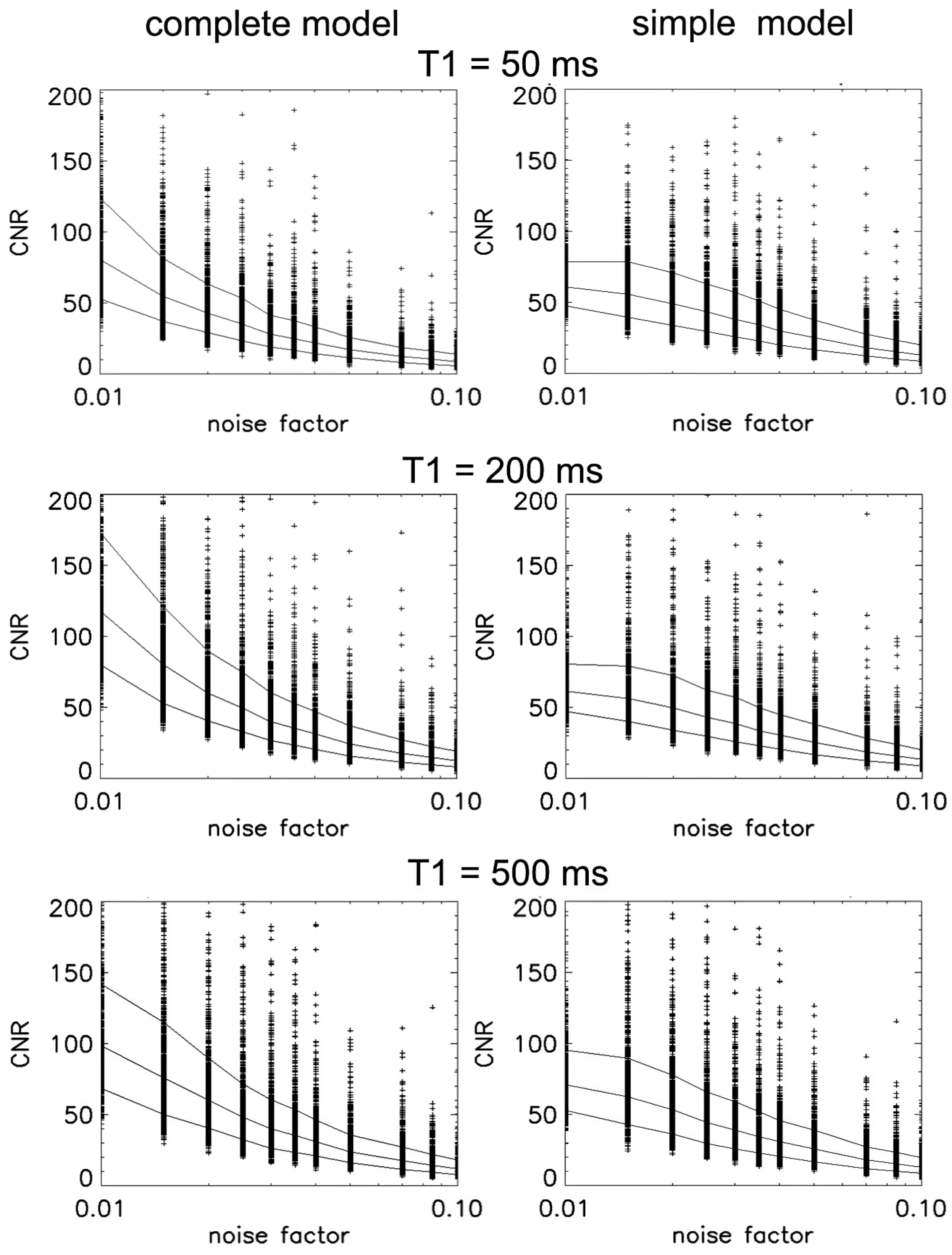

Abbildung 63: Simulation der Abhängigkeit des Kontrast-zu-Rausch-Verhältnisses (CNR) vom im k-Raum zugefügten Rauschen. Die Linien zeigen Mittelwert ± Standardabweichung des logarithmisch skalierten CNR an. Die Sequenz ist auf T_1 = 50 ms, 200 ms und 500 ms unter Berücksichtigung (links) bzw. Vernachlässigung (rechts) der T_1-Relaxation zwischen den RF-Impulsen innerhalb eines Herzzyklus optimiert.

Für die oben beschriebenen Bedingungen dieser Simulation wird optimales CNR durch Sequenzoptimierung auf eine T_1-Relaxationszeit von etwa 200 ms erzielt. Die Art der Optimierung beeinflusst das CNR um mehr als 100 % (Abbildung 62). Die Ursache dafür dürfte hauptsächlich in der Unterrepräsentation der niedrigen Ortsfrequenzen und damit im für die vereinfachte Optimierung niedrigeren Signalniveau zu suchen sein. Zusätzlich beeinflussen die oben beschriebenen Bildverzerrungen in Form von nichtstochastischen Störungen das Hintergrundsignal.

5.6.3.3 Messungen

Die Ergebnisse der Simulationen wurden anhand von Messungen an Schweinen (im Rahmen einer Strahlentherapie-Studie (Studien-Nr. FKZ01ZZ96404 des Bundesministeriums für Bildung und Forschung) mit einem intravaskulär verbleibenden Kontrastmittel (Gadomer-17 (Schering)) sowie mit einer Dreifachdosis Gadolinium-DTPA (0.3 mmol/kg Magnevist (Schering)) im Selbstversuch (die angegebene Dosis ist in der Literatur [129, 36, 111] als sicher beschrieben) validiert.

Für Bestimmungen des CNR wurden Signalintensitäten im rechten Vorhof mit denen in Fett (als praktisch kein Kontrastmittel aufnehmendes Gewebe) sowie in der Aorta mit denen in Muskel verglichen. Obwohl die Reproduzierbarkeit von Signalintensitäts-Bestimmungen in strömendem Blut durch verschiedene Effekte wie Fluss und Phasendispersion nur eingeschränkt ist, wurden Verbesserungen des CNR in der Größenordnung von 25 % (Vorhof gegen Fett) bis zu 100 % (Aorta gegen Muskel) beobachtet. Die Auswirkung oben beschriebener Sequenzoptimierung in der Bildgebung ist in Abbildung 64 und Abbildung 65 demonstriert (Selbstversuch). Obwohl die auf T_1 = 200 ms unter Berücksichtigung der T_1-Relaxation zwischen den RF-Impulsen innerhalb eines Herzzyklus einen verrauschteren Eindruck erweckt als die Originalsequenz (Optimierung auf T_1 = 300 ms unter Vernachlässigung dieser Relaxation), lässt sich die linke vordere absteigende Koronararterie damit über eine längere Strecke verfolgen.

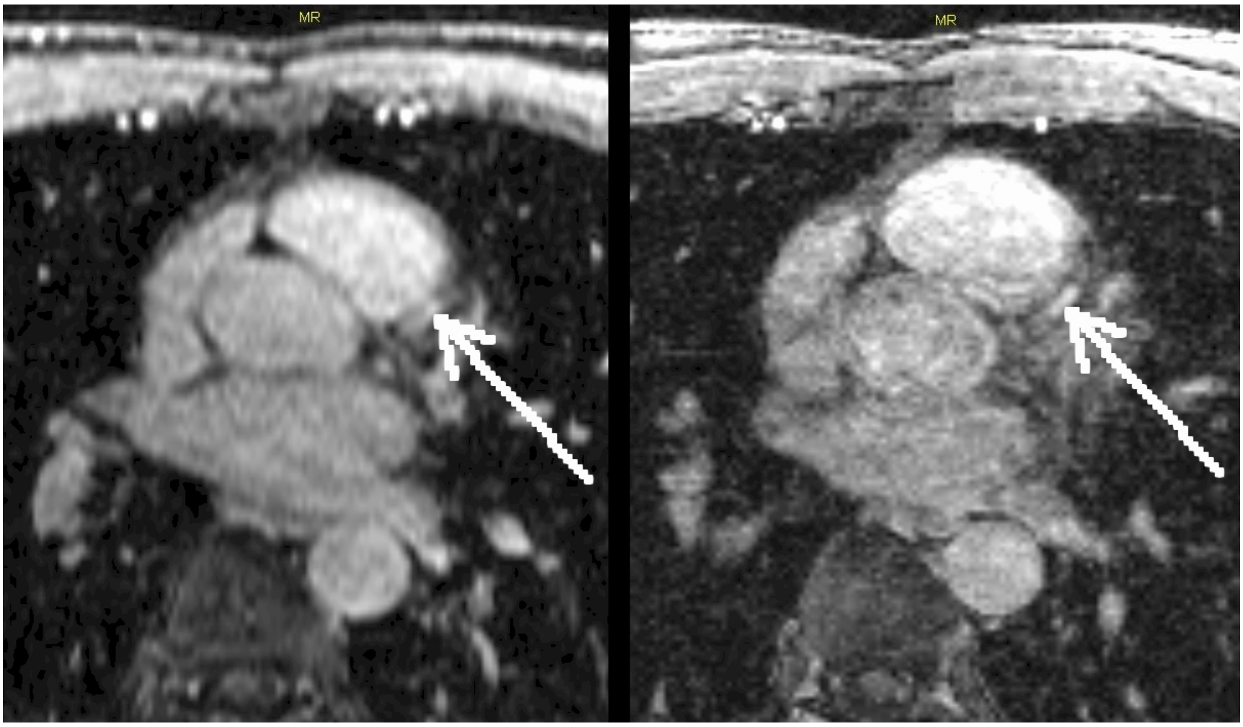

Abbildung 64: Darstellung der linken vorderen absteigenden Koronararterie (left anterior descendent coronary artery – LAD) eines Probanden. Primäre Schnittbilder nach Gabe von Gd-DTPA. Links: Originalsequenz mit einer für T_1 = 300 ms unter Vernachlässigung der T_1-Relaxation zwischen den RF-Impulsen eines Herzzyklus optimierten Flipwinkelfolge. Rechts: Optimierung auf T_1 = 200 ms mit Berücksichtigung dieser Relaxation.

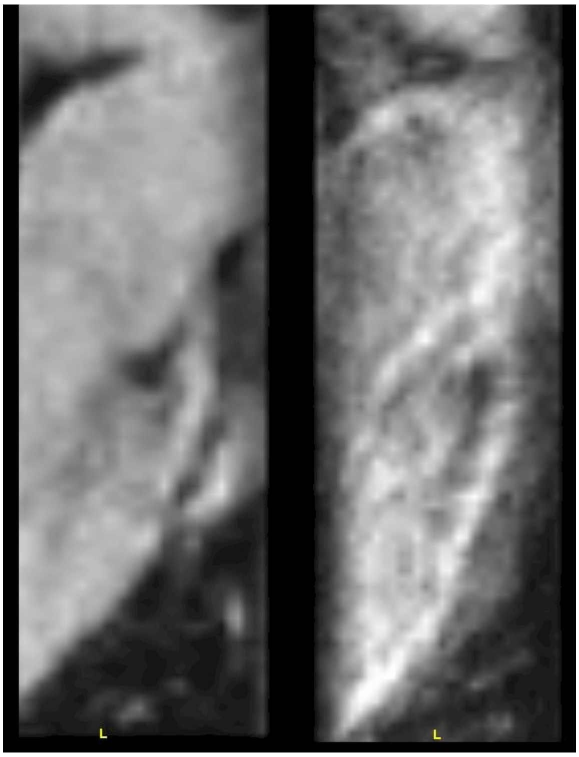

Abbildung 65: Selbe Messung wie in Abbildung 64 (nähere Erläuterung siehe dort). Gekrümmte Rekonstruktion.

5.6.4 Diskussion

In Sequenzen mit mehreren RF-Anregungen innerhalb eines Herzzyklus, wie sie z. B. in der Bildgebung des Herzens angewendet werden, nimmt der Grad der T_1-Relaxation während der aktiven Phase (im Gegensatz zur Warte-Phase auf den nächsten EKG-Trigger-Impuls) mit der Anzahl dieser Anregungen pro Herzzyklus zu. Für Sequenzen zur Darstellung von Gewebe mit sehr kurzer T_1-Zeit ist es daher sinnvoll, diese Relaxation in der Bestimmung einer optimalen Flipwinkel-Folge zu berücksichtigen. Es ergeben sich damit etwas höhere Flipwinkel für die ersten Anregungen, was zu einer höheren Signalausbeute für Strukturen mit kurzer T_1-Zeit sowie zu geringerem Signal für Gewebe mit längerem T_1 führt.

Mittels Computer-Simulation der Abhängigkeit des Kontrast-Rausch-Verhältnisses (CNR) einer 3D-FLASH-Sequenz von der Art der Flipwinkel-Optimierung lassen sich Hinweise erhalten, auf welche T_1-Zeit eine Sequenz optimiert werden sollte. Wenn die Messungen nach Gabe eines paramagnetischen Kontrastmittels erfolgen sollen, ist hierfür ein Wert zwischen den T_1-Zeiten des kontrast-verstärkten Gefäßes und des umgebenden Gewebes zu wählen. Unter den in der vorliegenden Untersuchung getroffenen Annahmen erscheint eine Optimierung auf $T_1 = 200$ ms zweckmäßig. Obwohl die kontrastverstärkten Gefäße die wichtigsten Strukturen bei der MR-Koronarographie darstellen, erscheint es nicht sinnvoll, die Sequenz auf eine extrem kurze T_1-Zeit zu optimieren, da dies über eine verzerrte Darstellung von umgebendem Gewebe mit längerer T_1-Zeit zu einer Reduktion des CNR zwischen dem Blutgefäß und dessen Umgebung führen würde.

Die erhaltenen Ergebnisse sind hochsignifikant. Allerdings ist die Anwendung statistischer Tests auf Untersuchungen mit großen (gemessenen oder simulierten) Stichproben nur bedingt von Wert, da beliebig kleine Unterschiede zwischen statistischen Verteilungen durch hinreichend große Stichprobenumfänge zur Signifikanz geführt werden können. Da die Verteilung des CNR (welches selbst eine Zufallsgröße ist,) sich als ziemlich breit erweist, sollte der Einfluss modifizierter Messbedingungen auf die Bildqualität nicht überschätzt werden. Trotzdem zeigen die vorgestellten Messungen, dass die Bildgebung der Koronararterien durch die Gabe eines intravaskulär verbleibenden, aber auch selbst eines extrazellulären Kontrastmittels spürbar verbessert werden kann (beschrieben auch in [152, 85, 116]), sowie dass das CNR durch die hier beschriebene Sequenzoptimierung weiter angehoben werden kann. Das erhöhte Kontrast-Rausch-Verhältnis sollte sich für die weitere Verbesserung des räumlichen Auflösungsvermögens der MR-Koronarographie verwendet werden.

6 Fehlerbetrachtungen

6.1 Fehlerfortpflanzung

Jede Umrechnung MR-tomographischer Messungen in physiologische Parameter stellt eine mathematische Verknüpfung mehrerer Messergebnisse (unter verschiedenen Messbedingungen, zu verschiedenen Zeitpunkten, ...) dar, von denen jedes prinzipiell fehlerbehaftet ist. Wenn die Messfehler voneinander unabhängig sind, kann der absolute Fehler der berechneten Größe dy in linearer Näherung als Summe der mit den partiellen Differentialen nach den Einzelmessungen gewichteten absoluten Fehlern der Einzelmessungen dx_i abgeschätzt werden (Gleichung (95))

$$dy = \sum_i \left| \frac{\partial y}{\partial x_i} * dx_i \right| \qquad (95)$$

Übliche Maßnahmen zur Verbesserung des Signal-zu-Rausch-Verhältnisses (Erhöhung der Pixelgröße bis hin zu Auswertungen über Regions of Interest, Mittelung über mehrere Messungen) lassen sich exakter über die Eigenschaft der Poisson-Verteilung (Gleichung (96)) behandeln, dass der Verteilungsparameter λ sowohl gleich dem Mittelwert μ als auch der Varianz σ^2 ist (Gleichung (97)) [151 S. 286 ff].

$$P(x) = \frac{\lambda^x * \exp(-\lambda)}{x!} \qquad (96)$$

$$\lambda = \mu = \sigma^2 \qquad (97)$$

Hiernach verbessert sich das Signal-Rausch-Verhältnis mit der Quadratwurzel der Signalintensität (Gleichung (98))

$$SNR = \frac{\mu}{\sigma} = \sqrt{\mu} \qquad (98)$$

Bei der Analyse kernspintomographischer Bilder insbesondere in Bereichen niedriger Signalintensität ist eine additive Rauschkomponente bestimmend. Sie resultiert sowohl aus thermischem Rauschen, welches über die Oberflächenspulen aus dem untersuchten Objekt empfangen wird und mit der Größe des „Sichtfeldes“ der Spule wächst, als auch aus den Rauschbeiträgen im RF-Empfängersystem selbst. Diese Komponente wird durch die Fourier-Transformation bei der Bild-Rekonstruktion an allen Bildpunkten wirksam.

Aus Gleichung (98) folgt, dass das Signal-Rausch-Verhältnis proportional zur Wurzel des in eine Region of Interest eingeschlossenen Volumens wächst (Voraussetzung: Signal innerhalb dieses Volumens hinreichend homogen).

6.2 Intravasale Kontrastmittel-Konzentration

Als abgeleitete Größen sind in dieser Arbeit Schätzungen von ΔR_1 und insbesondere ΔR_2 relevant.

Bei Schätzung von ΔR_2 aus Einzelecho-Messungen gemäß Gleichung (26) addieren sich im Argument der Logarithmus-Funktion die relativen Fehler von Messungen während der Boluspassage (S(t)) und von Baselinemessungen (S(0)). Bei der Schätzung von ΔR_2 über Gleichung (52) summieren sich zeitabhängige ($S_i(t)$) und Baseline-Beiträge ($S_i(0)$). Der Einfluss letzterer kann durch eine hinreichende Anzahl von Wiederholungsmessungen vernachlässigt werden (Bei den im Rahmen dieser Arbeit durchgeführten Doppelecho-Messungen wurden Baseline-Intensitäten aus jeweils 10 Einzelmessungen bestimmt, was einem um etwa den Faktor 3 verbesserten Signal-Rausch-Verhältnis entspricht).

Da mit Gleichung (95) der absolute Fehler einer logarithmierten Größe ln(x) gleich dem relativen Fehler der Größe x ist, ergibt sich der absolute Fehler der Kontrastmittel-Konzentrations-Schätzungen im Wesentlichen aus der Summe der relativen Fehler der Signalintensitäten (Gleichungen (99) und (100)):

Einzelecho-Schätzung nach Gleichung (26)

$$dC = \frac{1}{r * T_E} * \left(\left| \frac{dS(t)}{S(t)} \right| + \left| \frac{dS(0)}{S(0)} \right| \right) \qquad (99)$$

Doppelecho-Schätzung nach Gleichung (52)

$$dC = \frac{1}{r * (T_{E2} - T_{E1})} * \left(\left| \frac{dS_1(t)}{S_1(t)} \right| + \left| \frac{dS_1(0)}{S_1(0)} \right| + \left| \frac{dS_2(t)}{S_2(t)} \right| + \left| \frac{dS_2(0)}{S_2(0)} \right| \right) \qquad (100)$$

Für die Doppelecho-FLASH-Sequenz nach Abschnitt 4.2 folgt hieraus, dass der absolute zufällige Fehler der Konzentrationsschätzung nach Doppelecho-Korrektur um den Faktor 3 größer ist als der nach Schätzung nur aus dem Intensitätsverlauf des zweiten Echos (Annahmen: $T_2^* \approx 50$ ms ($\Rightarrow S_1/S_2 \approx 1{,}5$ für $T_{E1} = 15$ ms und $T_{E2} = 35$ ms), gleiche Varianz der Signalintensitäten beider Echos $dS_1 = dS_2$).

6.3 Arterielle Inputfunktion

Die Schätzung der Arteriellen Inputfunktion wird durch zwei Aspekte erschwert: die Auswahl der zu ihrer Bestimmung einzuschließenden Bildpunkte und Störungen der Beziehung zwischen intravasaler KM-Konzentration und Signalintensitätsverlust.

In der Literatur sind unterschiedliche Pixel-Auswahl-Strategien zwischen vollständig manueller Auswahl und vollautomatischen Algorithmen beschrieben. Abbildung 66 zeigt den erheblichen Einfluss des Auswahl-Algorithmus auf die Schätzung der AIF. Aus Abbildung 67 wird deutlich, dass zufällige und/oder partialvolumen-bedingte Fehler den Einfluss der Gewebs-Passage überdecken können.

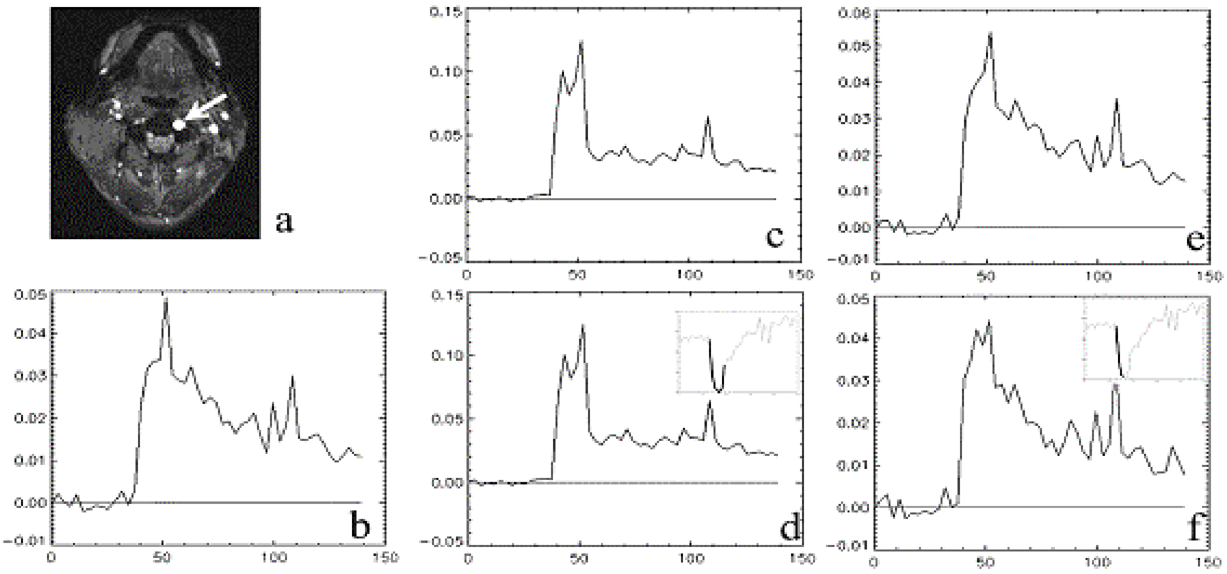

Abbildung 66: Variabilität der aus der linken A. carotis bestimmten AIF in Abhängigkeit von den Pixel-Auswahlkriterien. Der Pfeil in a markiert die manuell eingezeichnete Region, die durch weitere Bedingungen wie folgt eingegrenzt wird: b: keine weiteren Bedingungen; c: maximaler relativer Signalverlust mindestens 50 % sowie Baseline-Wert von mindestens 50 % des signalreichsten Pixels der Region; d: maximaler Signalverlust innerhalb des in der rechten oberen Ecke fett markierten Zeitfensters; e: Bedingungen von c und d; f: wie d innerhalb eines engeren Zeitfensters.

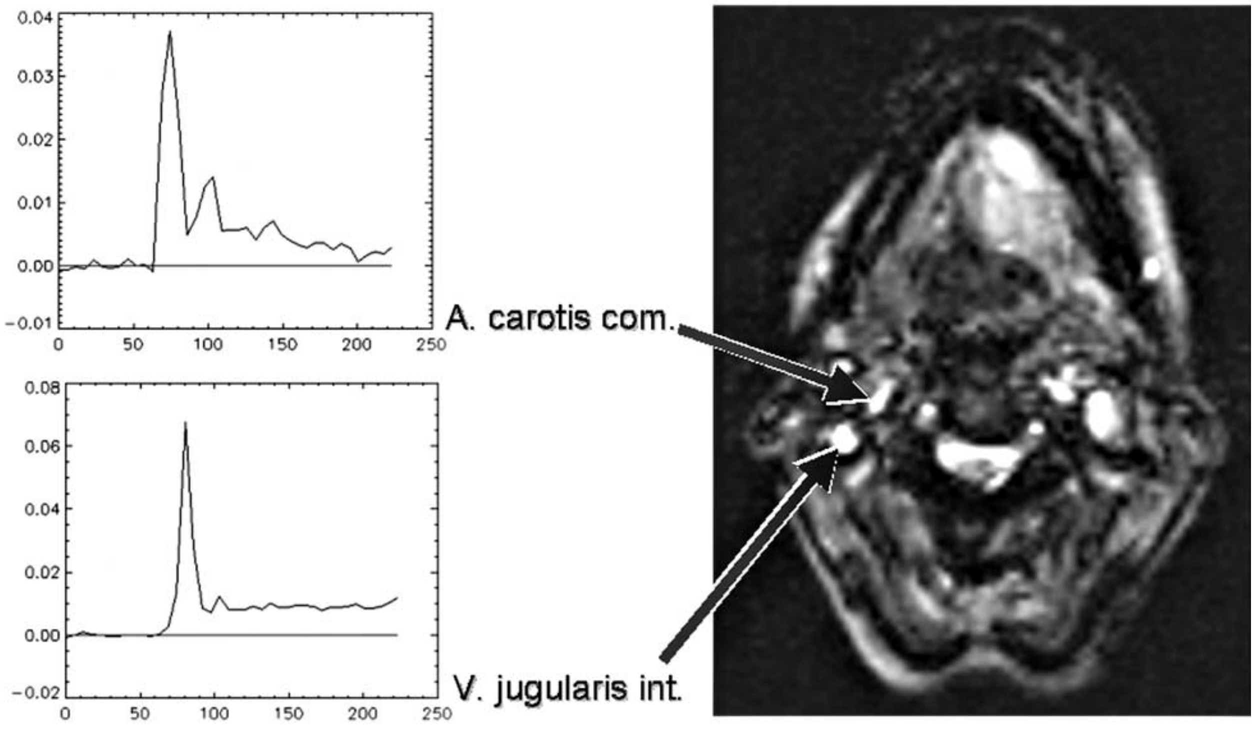

Abbildung 67: Variabilität der AIF in Abhängigkeit von der Pixel-Auswahl. Obwohl im Blutstrom nachgelagert, stellt sich die Boluspassage in den venösen Pixeln schmaler dar als in den arteriellen.

Hohe KM-Konzentrationen, mit denen in Arterien zu rechnen ist, resultieren in sehr niedrigen Signalintensitäten. Hier führt die oben erwähnte additive Rauschkomponente zu einer systematischen Überschätzung der Intensität $S_i(t)$ in Gleichung (26) bzw. (52) und damit zu einer Unterschätzung der maximalen KM-Konzentration. Die auf Grund des Rauschens maximale nachweisbare Konzentration lässt sich aus dem Signal-Rausch-Verhältnis einer (einzelnen) Baseline-Messung $SNR(t_0)$ abschätzen (Gleichung (102) für Einzelecho-Auswertungen)

$$\frac{S(t_0)}{S_{\min}} = \frac{S(t_0)}{\sigma} = SNR(t_0) \qquad (101)$$

$$C_{\max} = \frac{1}{r * T_E} \ln(SNR(t_0)) \qquad (102)$$

Für Doppelecho-Auswertungen ist die obere Nachweisgrenze prinzipiell auch vom Signalzuwachs im ersten Echo zum Zeitpunkt der maximalen intravasalen KM-Konzentration abhängig. Im ungünstigsten Fall, d.h. für $S_1(t_{C=C\max}) \approx S_1(t_0)$ ergibt sich Gleichung (103):

$$C_{\max} = \frac{1}{r * \left(T_{E2} - T_{E1}\right)} * \ln\left(SNR_2(t_0)\right) \qquad (103)$$

wobei $SNR_2(t_0)$ für das Signal-Rausch-Verhältnis des zweiten Echos einer Einzelmessung vor KM-Gabe steht. In Abschnitt 6.4 wird die systematische Verzerrung der Schätzung niedriger Signalintensitäten näher behandelt.

Im Fall von EPI-Messungen muss als weitere Fehlerquelle die Flussabhängigkeit des Signals berücksichtigt werden. Im Gegensatz zu anderen Messstrategien akkumulieren Spins, die sich entlang der Phasencodierrichtung bewegen, während der gesamten Dauer der Gradientenpulsfolge (Größenordnung 0,1 s) einen flussabhängigen Phasenfehler. Innerhalb dieser Zeit legt Blut in der A. carotis eine Strecke von ca. 10 bis 15 cm zurück – dies entspricht etwa der halben Größe des Field of View (FOV).

Die folgende Betrachtung bezieht sich im Interesse der Übersichtlichkeit auf ein Voxel am Rand des FOV. Wenn dieses sich nicht bewegt, wächst das Integral über den Phasencodiergradienten für diese Spins $k_{stat}(i)$ mit jedem Codierschritt i beginnend bei einem Startwert k(0) um das Inkrement Δk_{stat}: (Gleichung (104)):

$$k_{stat}(i) = k(0) + \Delta k_{stat} * i \qquad (104)$$

Wenn sich die Spins dieses Voxels während der Akquisitionsphase der Sequenz mit der Geschwindigkeit v (in Einheiten der Größe des FOV, geteilt durch die Dauer der Gradientenpulsfolge) entlang der Phasencodierrichtung bewegen, ist das Inkrement Δk_{flow} nicht mehr konstant (Gleichungen (105), (106)):

$$\Delta k_{flow} = \Delta k_{stat} * \left(1 - \frac{2*i}{nrows} * v\right) \qquad (105)$$

$$k_{flow}(i) = k(0) + \Delta k_{stat} * \left(i - \frac{i^2}{nrows} * v\right) \qquad (106)$$

mit nrows als der Anzahl von Phasenkodierschritten. Dadurch werden sich bewegende “Voxel” geschwindigkeitsabhängig verzerrt dargestellt. In Abbildung 68 ist dies in einem simulierten k-Raum und Ortsraum dargestellt (die Verschmierung des Punktes nach rechts ist in den linken Bildteil gefaltet). Die Signalintensität der fließenden Spins ist über eine größere Strecke verteilt, als diese tatsächlich zurücklegen. Dies führt zu einer drastischen Reduktion der in entlang der Phasencodierrichtung durchströmten Gefäßen gemessenen Signalintensität.

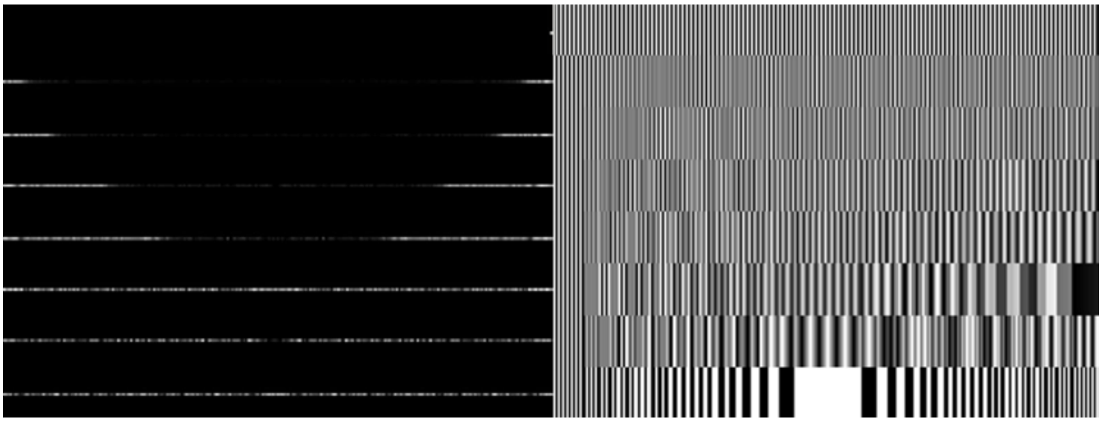

Abbildung 68: Simulation der Verzerrung der Darstellung eines sich in Phasencodierrichtung bewegenden Punktes bei Messung mittels EPI-Sequenz. Links Ortsraum, rechts k-Raum. Flussgeschwindigkeit (in Einheiten der Bildgröße pro Echozuglänge) von oben nach unten 0.0, 0.05, 0.1, 0.2, 0.3, 0.5, 0.75, 1.0

Zum experimentellen Nachweis dieses Effektes wurden Messungen mit einem Flussphantom durchgeführt. Schläuche mit Innendurchmessern von 2,5 und 4 mm wurden so in einem Wasserbad fixiert, dass Fluss in drei orthogonalen Raumrichtungen in einem Schnittbild dargestellt werden konnte. Mittels Kontrastmittel-Injektor wurden Flussraten von 0, 2, 5 und 10 cm³/s erzielt, die mittleren Flussgeschwindigkeiten von bis zu 200 cm/s im dünneren Schlauch und bis zu 80 cm/s im dickeren Schlauch entsprechen.

In der Darstellung des Phantoms mittels Doppelecho-FLASH (zweites Echo) stellt sich der in der Time-of-Flight-Angiographie benutzte Inflow-Effekt dar (Abbildung 69 a - d), der bei zu langem Aufenthalt der Spins in der Bildebene verschwindet (Abbildung 69 b). Ein flussabhängiger Signalverlust ist nicht nachzuweisen.

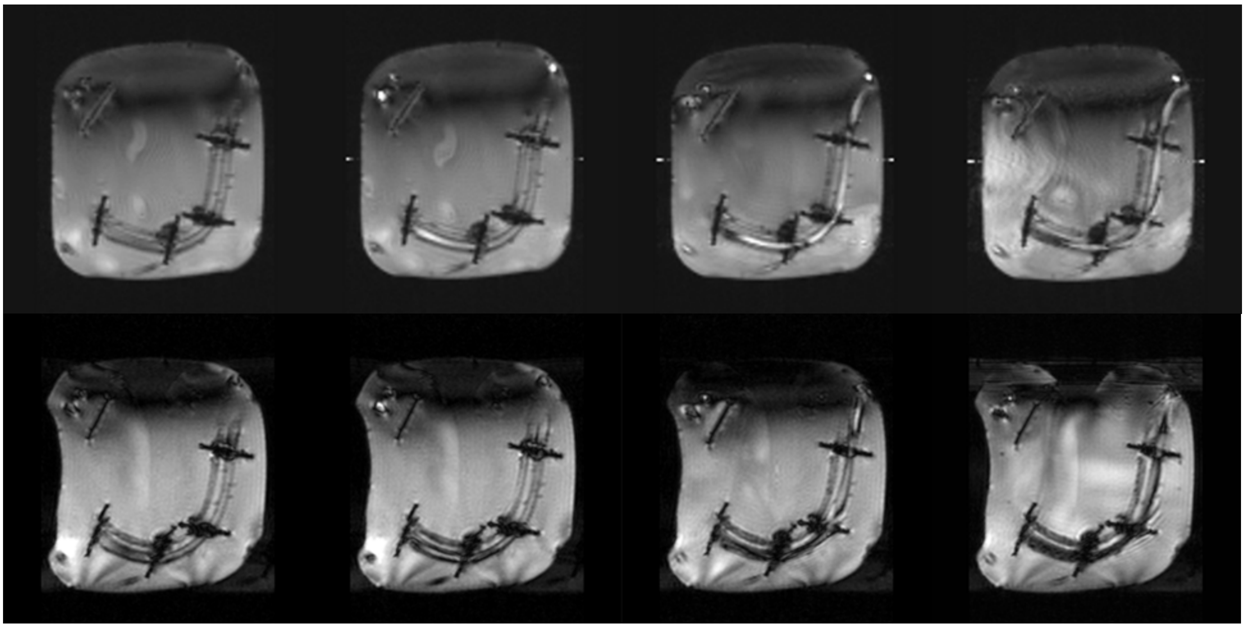

Abbildung 69: Darstellung strömender Flüssigkeit. Obere Zeile (a – d): mittels Doppelecho-FLASH-Sequenz (zweites Echo), untere Zeile (e – h): mittels EPI-Sequenz. Flussgeschwindigkeiten von links nach rechts: 0 cm³/s, 2 cm³/s, 5 cm³/s, 10 cm³/s. Phasenkodierrichtung von links nach rechts.

Bei den EPI-Bildern (Abbildung 69 e - h) deutet sich in Schichtselektions- und Ausleserichtung ebenfalls der Inflow-Effekt an (Abbildung 69 f). Die Bewegung in Phasencodierrichtung führt dagegen hier wie oben abgeleitet zu einer signalarmen Darstellung der Schlauch-Lumina (Abbildung 69 g - h). Dies gilt natürlich auch für Flusskomponenten in den gekrümmten Schlauchabschnitten.

Eine weitere Störgröße ist der Durchmesser der zum Signalverlust beitragenden Gefäße. Auf die Abhängigkeit des dephasierungsbedingten Signalverlustes vom Durchmesser der Bereiche mit von der Umgebung unterschiedlicher Suszeptibilität (hier: große Arterien vs. Kapillaren) wurde im Abschnitt 2.2.4.4 eingegangen.

Die hier aufgezeigten Aspekte könnten mit dafür verantwortlich sein, dass Klose et al. [102] die Bestimmung von absoluten KM-Konzentrationen auf Grundlage einer Normierung auf die AIF als zu stark fehlerbehaftet einschätzen.

6.4 Schätzung niedriger Signalintensitäten

MR-tomographische Bilder repräsentieren üblicherweise den Betrag der Fourier-transformierten des k-Raumes (abweichend hiervon wird beispielsweise bei Inversion-Recovery-Messungen auch der Realteil dargestellt). Bei einem Mittelwert der komplexen Signalintensität $\bar{I}$ von Null führt die Betragsbildung zu einer systematischen Verzerrung in Richtung höher Intensitäten.

Unter der Annahme einer normalverteilten additiven Rauschkomponente im Bild mit der Streuung σ lässt sich der Erwartungswert des Betrages (also des Messwertes) $\widehat{I}$ wie folgt formulieren (Gleichung (107):

$$\widehat{I} = \int_{-\infty}^{\infty} x * \left| \frac{1}{\sqrt{2*\pi}*\sigma} * \exp\left(-\frac{(x-\bar{I})^2}{2*\sigma^2} \right) \right| dx$$

$$= \int_{0}^{\infty} x * \frac{1}{\sqrt{2*\pi}*\sigma} * \exp\left(-\frac{(x-\bar{I})^2}{2*\sigma^2} \right) dx + \int_{0}^{\infty} x * \frac{1}{\sqrt{2*\pi}*\sigma} * \exp\left(-\frac{(-x-\bar{I})^2}{2*\sigma^2} \right) dx \qquad (107)$$

Die Lösung dieses bestimmten Integrals (unter Benutzung des „Integrators“ [185]) lautet (Gleichung (108):

$$\widehat{I} = \frac{1}{\sigma*\sqrt{2*\pi}} * \left(2*\sigma^2*\exp\left(-\frac{\bar{I}^2}{2*\sigma^2} \right) + 2*\bar{I}*\sqrt{\frac{\pi}{2}}*\sigma*erf\left(\frac{\bar{I}}{\sigma*\sqrt{2}} \right) \right) \qquad (108)$$

mit dem Fehlerintegral $erf(z) = \frac{2}{\sqrt{\pi}} * \int_0^z \exp(-t^2)dt$. Das Ausmaß der Verschiebung des Erwartungswertes durch Rauschen ist in Abbildung 70 dargestellt. Sie unterschreitet die Schwelle von 1 % der ohne Rauschen zu messenden Intensität, wenn dieser Wert um ca. 2 * σ über Null liegt.

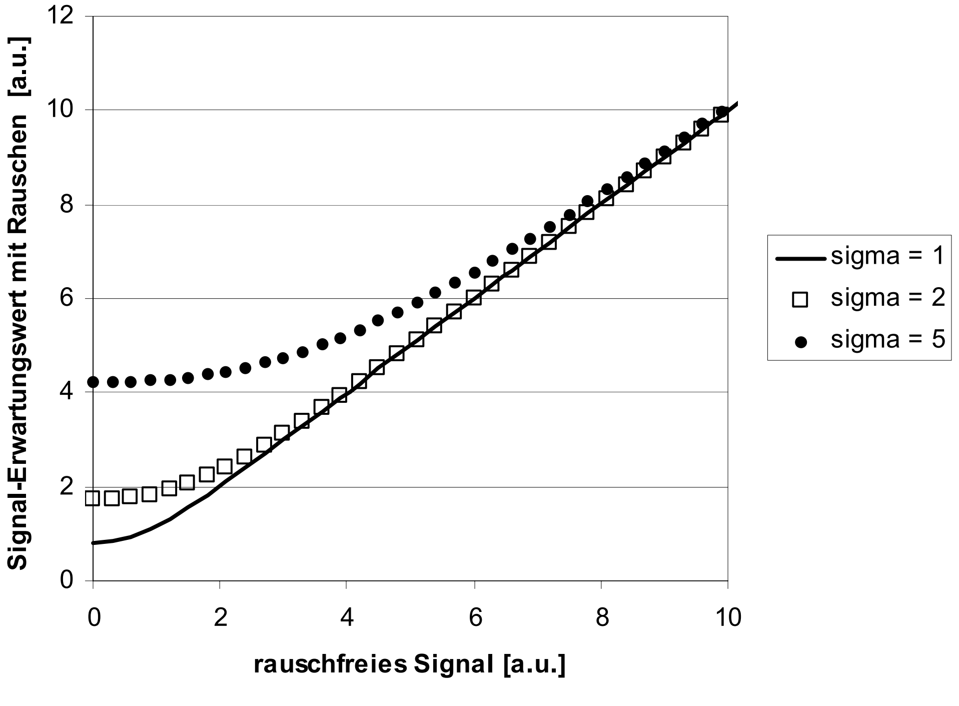

Abbildung 70: Verzerrung des Erwartungswertes des Betrages einer Signalintensität durch additives Gauß-Rauschen.

Unter der Annahme eines gleichmäßig über das gesamte Bild verteilten stochastischen Rauschens (im Gegensatz zu strukturellem Rauschen, welches durch Inhomogenitäten des dargestellten Objektes oder durch Bildartefakte verursacht ist) sollte σ in einem „signalfreien“ Bildbereich bestimmt werden. Für die in den Abschnitten 5.4 und 5.5 dargestellten Messungen erweist sich die rauschbedingte Verzerrung von Signalintensitäten als vernachlässigbar (Abbildung 71 und Tabelle 17).

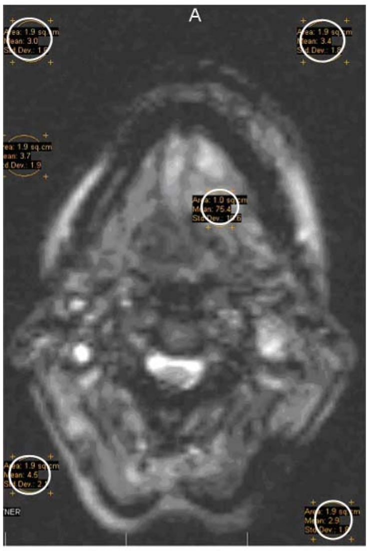

Abbildung 71: Regionen zur Bestimmung von Rauschen und Signalintensität bei der MR-Perfusionsmessung an pharyngealen Tumoren (Patient KI vor Therapie)

Region	Mittelwert	Standard-abweichung
Signalfrei # 1	3,0	1,6
Signalfrei # 2	3,4	1,8
Signalfrei # 3	3,7	1,9
Signalfrei # 4	4,5	2,1
Signalfrei # 5	2,9	1,8
Tumor	75,4	10,6
A. carotis (Signalminimum)	40,1	13,5

Tabelle 17: Signalintensitäten und Standardabweichungen in den in Abbildung 71 dargestellten Regionen

6.5 Funktionelle Bilder

In verschiedenen Bildverarbeitungs-Schritten (Erzeugung relativer Parameter, Berechnung von T_2^*-Bildern – Gleichung (51)) spielen Signalintensitäts-Quotienten eine Rolle. Aus Gleichung (95) folgt, dass der relative Fehler von Produkten bzw. Quotienten gleich der Summe der relativen Fehler der Faktoren (bzw. von Dividend und Divisor) ist. Bei über das Bild homogenem Rauschen, wie es in der MRT mit hinreichender Genauigkeit angenommen werden kann, wird daher der relative Fehler von Produkt- bzw. Quotientenbildern in Bereichen niedriger Intensität ggflls. extrem groß. Dies betrifft die Doppelecho-Korrektur von Perfusionsmessungen an Kontrastmittel aufnehmenden Läsionen (Abschnitt 5.1, Gleichung (51)), nach der

erhebliche statistische Ausreißer im Bild der berechneten T_2^*-Werte auftreten (Abbildung 72). Diese lassen sich zwar deutlich unterdrücken, wenn die Korrektur an geglätteten Bildern mit entsprechend besserem Signal-Rausch-Verhältnis durchgeführt wird, dieses Vorgehen entspricht jedoch nicht dem Anliegen räumlich hochaufgelöster Messungen.

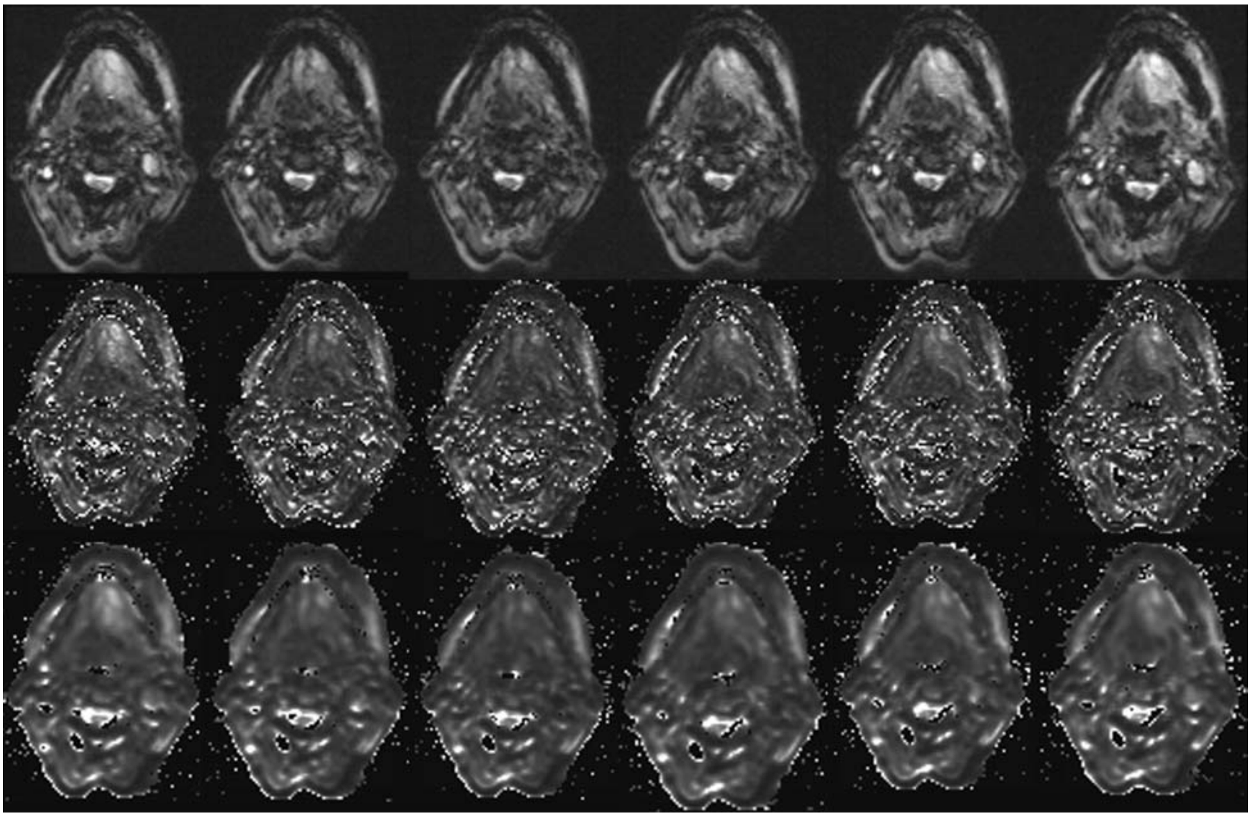

Abbildung 72: Doppelecho-FLASH-Perfusionsmessung an einem Pharynx-Tumor. Von links nach rechts: Boluspassage (Bilder a bis e) und letztes Bild der dynamischen Serie (Bild f – ca. 2:30 min nach Injektion). Obere Zeile: Intensitätsverlauf des zweiten Echos; mittlere Zeile: nach Gleichung (51) berechnete T_2^*-Bilder; untere Zeile: nach Glättung der Bilder des 1. und 2. Echos berechnete T_2^*-Bilder

Die gleiche Argumentation trifft auf die Darstellung der Passage eines Kontrastmittel-Bolus (Abbildung 73) nach Entfaltung von der arteriellen Inputfunktion zu (vgl. Abschnitt 3.1.2). Diese Entfaltung wird üblicherweise im Raum der Signalintensitäten durchgeführt (Abbildung 74). Sie bezieht sich jedoch streng genommen auf Kontrastmittel-Konzentrationen, wie sie sich als Veränderungen der Relaxationsrate ΔR_2^* gemäß Gleichung (26) (Einzelecho-Messung) oder (52) (Doppelecho-Messung) berechnen lassen. Beide Gleichungen enthalten Intensitätsquotienten, was zu numerisch instabilen Ergebnissen in Bildbereichen mit niedriger Intensität führt (Abbildung 75). Vom klinischen Standpunkt führt die methodisch inkorrekte Entfaltung im Raum der Signalintensitäten zu besser interpretierbaren Ergebnissen als die Entfaltung im Raum der KM-Konzentrationen. Die Kernaussagen (verzögerte Perfusion im Infarktgebiet (pathologisch) sowie im intraventrikulären Plexus (physiologisch)) sind in allen drei abgebildeten Darstellungsvarianten deutlich sichtbar, wobei die Erzeugung der Differenzbilder (Abbildung 73) wegen des geringen Auswerte-Aufwands am praktikabelsten erscheint.

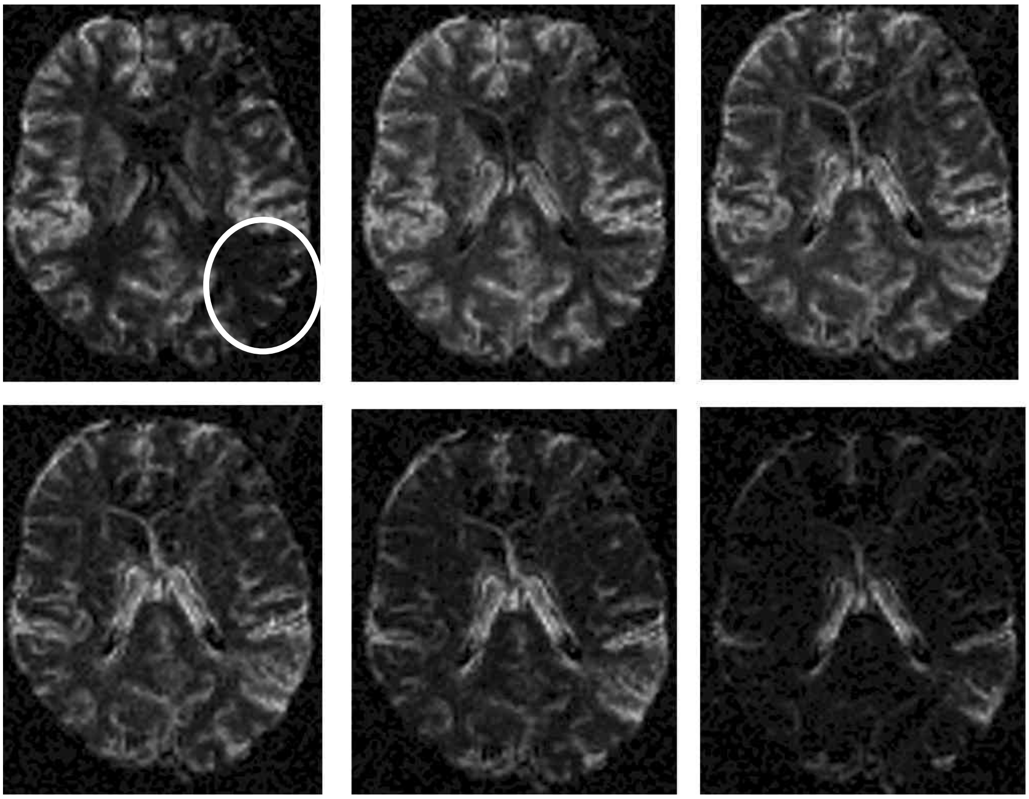

Abbildung 73: EPI-Messung der Passage eines Kontrastmittel-Bolus bei einem Patienten mit einem infarzierten Bereich im hinteren linken Hirnbereich (in Abbildung rechts – siehe Markierung). Differenzbilder zwischen Baseline-Scans und Aufnahmen während der Boluspassage.

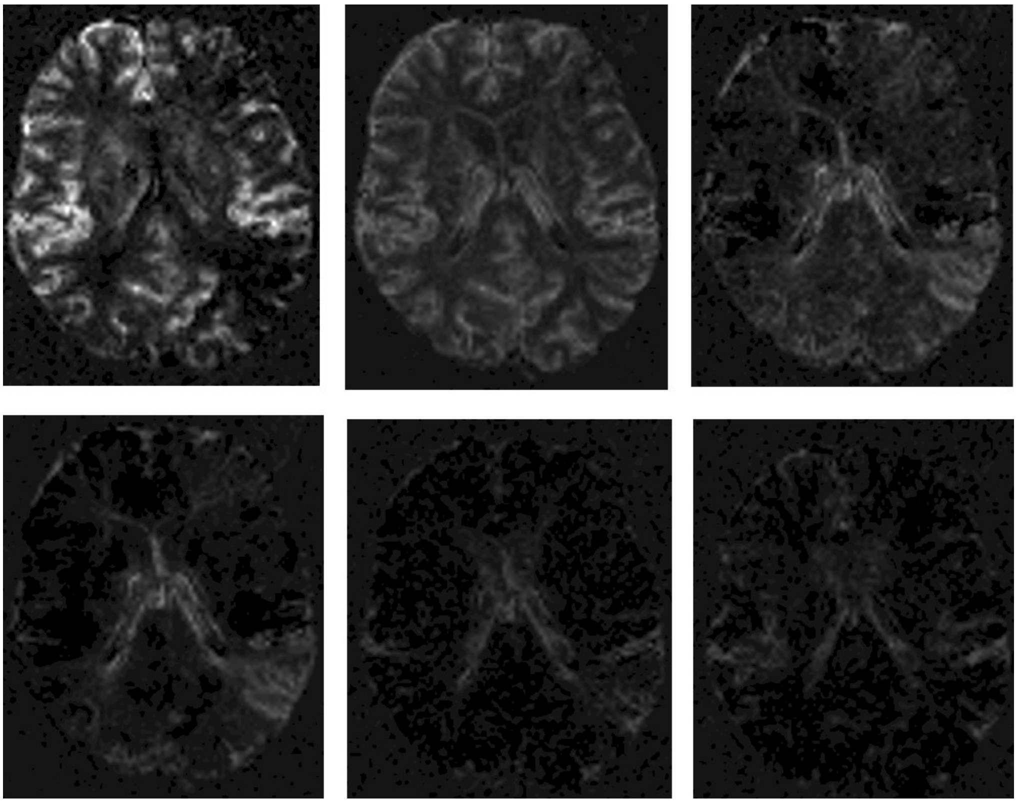

Abbildung 74: Selbe Messung wie in Abbildung 73, Entfaltung von der Arteriellen Inputfunktion im Raum der Signalintensitäten.

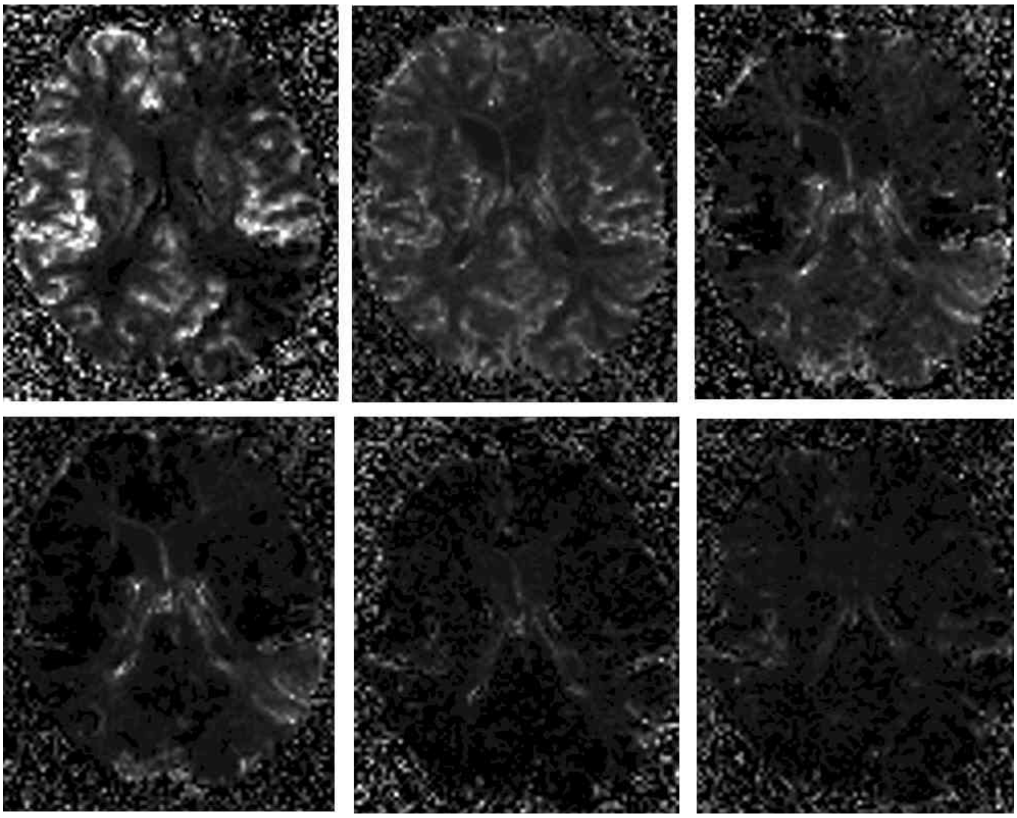

Abbildung 75: Selbe Messung wie in Abbildung 73, Entfaltung von der Arteriellen Inputfunktion im Raum der Kontrastmittel-Konzentrationen.

Als praktikable, weil schnell und robust berechenbare Parameterbilder zur Visualisierung des perfusionsbedingten temporären Signalverlustes bieten sich Darstellungen des maximalen Signalverlustes an und werden auf Medizingeräten entsprechend realisiert. Aus Gleichung (44) (Abschnitt 3.1.2) folgt, dass im Fall einer deltaförmigen arteriellen Inputfunktion die maximale im Gewebe beobachtete Kontrastmittel-Konzentration proportional zum Fluss ist. Der Signalverlust ist also „fluss-gewichtet“, und zwar umso mehr, je schmaler die arterielle Inputfunktion ist (Abbildung 26).

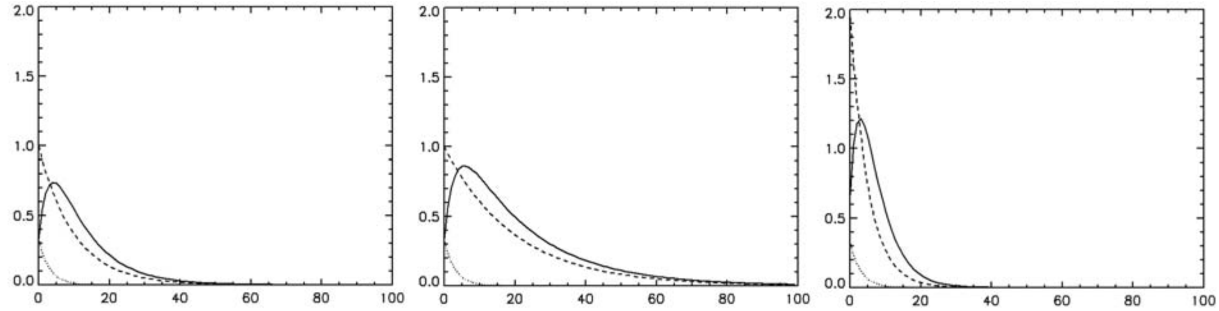

Abbildung 76: Simulation des Einflusses von Blutfluss und Blutvolumen auf den maximalen Signalverlust. A: Ausgangssituation; b: verdoppeltes Volumen bei gleichem Fluss wie in a; c: verdoppelter Fluss bei gleichem Volumen wie in a:. Durchgehende Linie: KM-Konzentration im Gewebe; gestrichelte Linie: Residuenfunktion; gepunktete Linie: Dispersionsfunktion.

Da sich die KM-Konzentration gemäß Gleichung (26) bzw. (52) aus relativen Intensitätsveränderungen bestimmen lässt und absolute Intensitätsveränderungen

daher prinzipiell in stärkerem Maße von nicht perfusionsabhängigen Gewebeparametern beeinflusst werden, sollten Darstellungen des maximalen relativen Signalverlustes die Vaskularisation korrekter widerspiegeln als der maximale absolute Signalverlust. Die geringere Stabilität von Quotienten im Vergleich zu Differenzen (Abbildung 77) führt in praxi jedoch dazu, dass die Betrachtung absoluter Signalveränderungen von größerer klinischer Relevanz ist – um so mehr, als der visuelle Vergleich der Durchblutungssituation zwischen strukturell sehr ähnlichen Gewebsregionen vorgenommen werden wird.

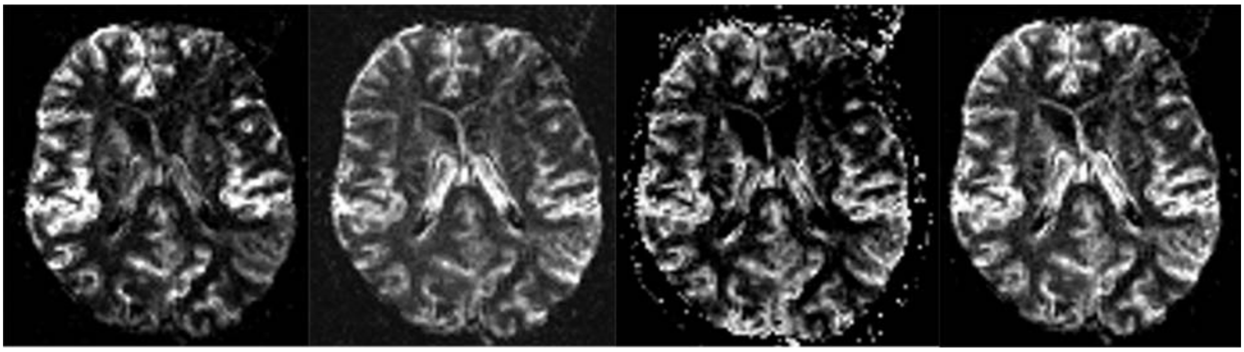

Abbildung 77: Selbe Messung wie in Abbildung 73, von links nach rechts Blutfluss (a), Blutvolumen (b), maximaler relativer (c) und maximaler absoluter (d) Signalverlust.

Bei der Generierung von „time-to-peak"-Darstellungen, also der Codierung des Zeitpunktes des Konzentrations-Maximums des Kontrastmittels als Pixelwerte, tritt eine Fehlerquelle auf, die nur bedingt als „Fehlerfortpflanzung" bezeichnet werden kann. Im einfachsten, an verschiedenen Medizingeräten realisierten Fall wird für jeden Bildpunkt die Nummer des Zeitpunktes helligkeitscodiert, zu dem die niedrigste (z. B. bei MR-Suszeptibilitäts-Bildgebung) oder höchste (z. B. bei kontrastmitteldynamischen Studien am CT) Intensität in der Zeitreihe beobachtet wurde. Bei gegebenem Signal-Rausch-Verhältnis führt diese Strategie dazu, dass die Wahrscheinlichkeit, innerhalb der Zeitreihe einen statistischen Ausreißer zu beobachten, mit der Anzahl der Messungen wächst. Zur Unterdrückung dieses Effektes können Glättungen in Raum und/oder Zeit vorgenommen werden, die jedoch mit einem Informationsverlust verbunden sind. Konsequenter wäre die Schätzung des Zeitpunktes der maximalen Kontrastmittel-Konzentration über eine Kurvenanpassung der Zeitverläufe oder aber die Berechnung der MTT. Bei diesen beiden Ansätzen geht jedoch der Vorteil einer simplen und damit schnellen Auswerte-Strategie verloren (Abbildung 78).

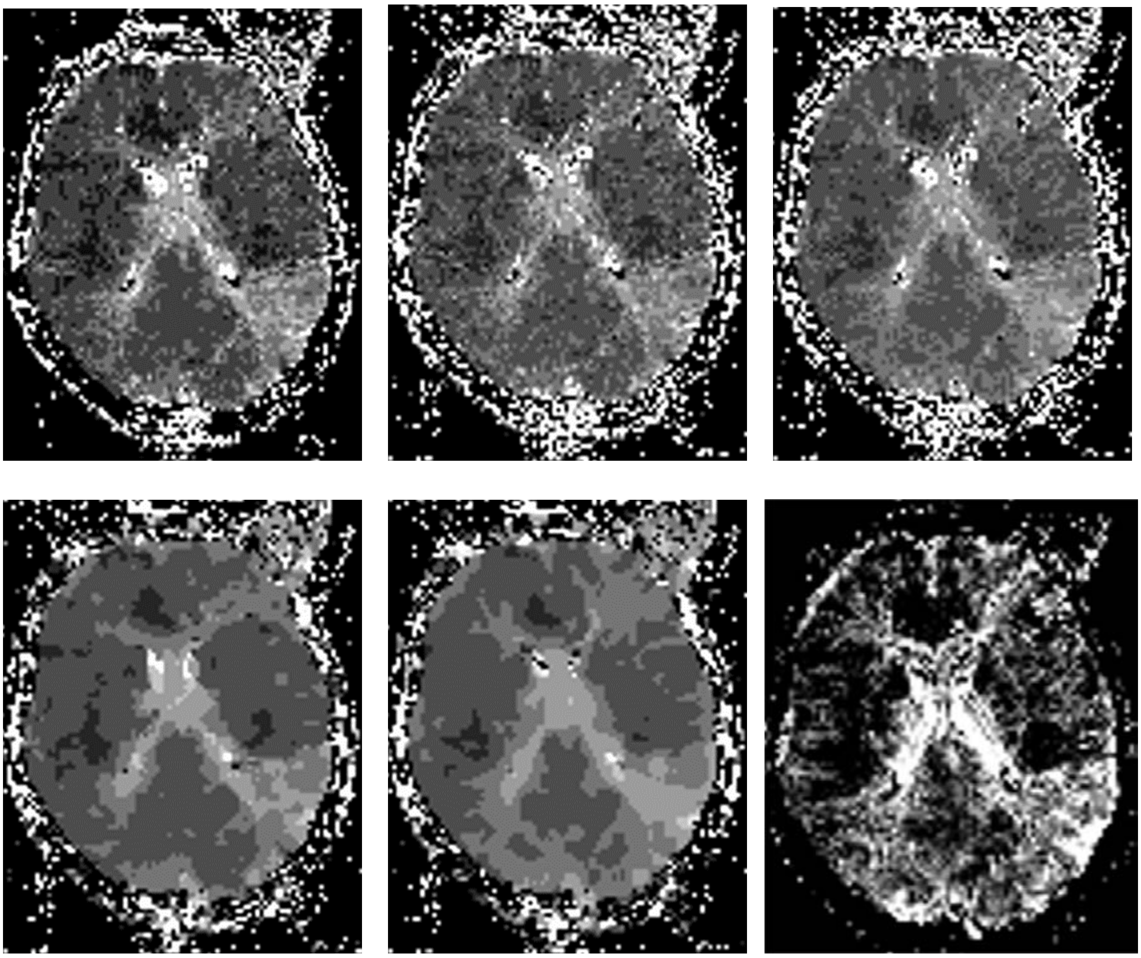

Abbildung 78: „Time-to-Peak"-Darstellung der selben Messung wie in Abbildung 73 ff.: Von links oben nach rechts unten: a) TTP-Darstellung des MRT-Scanner-Herstellers; b) Bildnummern, an denen der maximale Signalverlust beobachtet wurde; c) wie b), jedoch nach pixelweiser Glättung entlang der Zeitachse; d) wie b), jedoch nach Glättung im Raum, e) wie b), jedoch nach Glättung in Raum und Zeit; f) Berechnung der Mean Transit Time (MTT).

6.6 Diskussion

Es versteht sich von selbst, dass jegliche physiologische Information, die aus MRT-Bildern abgeleitet werden kann, bereits in den nicht nachverarbeiteten Bildern enthalten ist. So kann z. B. der perfusionsbedingte Signalverlust durch sehr einfache Verarbeitungsschritte wie die Erzeugung von Differenzbildern dargestellt werden. Mit Hilfe von Parameterbildern wird die physiologische Information in eine geringere Anzahl von Bildern komprimiert. In Abhängigkeit vom angewandten Algorithmus kann diese Kompression jedoch auf Grund der Überlagerung von Messfehlern oder durch die Zunahme der Wahrscheinlichkeit für statistische Ausreißer sogar zu einem Verlust an diagnostischer Information führen – letzteres wurde am Beispiel der Time-to-Peak-Bilder demonstriert. Das Ausreißer-Problem kann z. B. durch pixelweise Kurvenanpassungen reduziert werden. Für stabile Kurvenanpassungen müssen jedoch mehr oder weniger vereinfachende Modellannahmen getroffen werden.

Die wesentlichen Perfusionseigenschaften können durch Bilder des maximalen relativen oder sogar des maximalen absoluten Signalverlustes dargestellt werden, da diese durch relativen Blutfluss und –Volumen bestimmt sind. Die Berechnung relativer

regionaler Blutflüsse bzw. -volumina könnte zusätzliche klinische Information visuell zugänglich machen. Gegenstand weiterer Untersuchungen sollte sein, unter welchen Bedingungen hierfür Kosten (Nachbearbeitung, Fehlerfortpflanzung) und Nutzen (Diagnosebeeinflussung) in einem akzeptablen Verhältnis zueinander stehen.

Die Berücksichtigung der Arteriellen Inputfunktion scheint eine Voraussetzung sowohl für eine hinreichende Differenzierung zwischen relativem regionalen Blutfluss (rBF) und –volumen (rBV) ebenso wie für die Berechnung mittlerer Transitzeiten (MTT) zu sein. Im Prinzip müssen die entsprechenden Entfaltungs- bzw. Normierungsschritte im Raum der KM-Konzentrationen und nicht an den Signalintensitätsänderungen ausgeführt werden. Die rauschverstärkende Wirkung dieser Nachverarbeitungen macht es in Abhängigkeit vom Kontrast-Rausch-Verhältnis jedoch u. U. ratsam, den Einfluss der AIF vor der Transformation zu einer KM-Konzentration zu eliminieren. In der Literatur sind verschiedene Analysen zur Korrelation zwischen MR-Perfusionsparametern und unabhängigen Methoden beschrieben (^{133}Xe-SPECT [184], NIRS [186], Xe-CT [54], SPET [119]) – die gefundenen Korrelationen unterscheiden sich deutlich. Für die Bestimmung absoluter Blutvolumina ist eine zuverlässige Bestimmung der Zeitabhängigkeit der KM-bedingten ΔR_2^*-Änderung in arteriellem Blut – möglichst nahe dem untersuchten Gewebe – erforderlich.

Da bei EPI-Messungen die Signalintensität in Arterien während der Maximalkonzentration der Boluspassage sehr niedrig wird (bzw. angesichts endlichen Rauschens praktisch verschwinden kann), scheint die Messung einer AIF mit EPI die Einbeziehung von Voxeln zu erfordern, die zumindest teilweise die Arterie umgebendes Gewebe umfassen. Unter diesen Umständen sind Entfaltungen von der AIF sehr nützlich für die Betrachtung zeitlicher Aspekte wie der Berechnung von MTT oder relativem Fluss. Sie sind jedoch – durch den beschriebenen Partialvolumeneffekt – prinzipiell problematisch für die Berechnung von Absolutwerden des regionalen Blutvolumens. Darin könnte ein Grund für die unterschiedlichen Beziehungen zwischen mittels MRT und mittels anderer Methoden bestimmter Perfusionsparameter sein. Mit FLASH-Sequenzen ist die Bestimmung von Relaxationsveränderungen in arteriellem Blut zumindest in höherem Maße möglich, sie erlauben aber bei Mehrschichtmessungen nur eine begrenzte zeitliche Auflösung. Die oben dargestellten Aspekte der Fehlerfortpflanzung weisen darauf hin, dass die Berücksichtigung der AIF zumindest unter den hier angewandten Bedingungen den klinischen Wert der Perfusionsmessungen nicht wesentlich erhöht.

Bei quantitativen Perfusionsuntersuchungen an KM aufnehmenden Läsionen ist die Berücksichtigung der T_1-Verkürzung wesentlich. Die rauschverstärkende Wirkung der Korrektur bedingt, dass stabile Ergebnisse Glättungs- (oder Modellierungs-?) Schritte erfordern wie Verringerung der räumlichen Auflösung bis hin zur Beschränkung auf die Auswertung von Regions of Interest. Parameter, die ohne T_1-Korrektur abgeleitet wurden, enthalten Information sowohl über die Mikrozirkulation als auch über die KM-Aufnahme, und zwar in einer von den Sequenzparametern abhängigen Kombination. In der vorliegenden Arbeit wurde gezeigt, dass solche „versehentlich bivariaten" Parameter wie z. B. der maximale relative Signalverlust wertvolle differential-diagnostische Information enthalten können [78].

7 Diskussion und gerätetechnische Schlussfolgerungen

7.1 Globale Einschätzung

Die Kernspintomographie hat sich als Methode etabliert, die in der Lage ist, verschiedenste Informationen über Gewebe und dessen Beziehung zum Organismus bereitzustellen. Dies ist durch die Vielzahl an physikalischen Effekten begründet, die – je nach Gestaltung der Messung – signal-beeinflussend wirken. Als intrinsische Parameter werden die Protonendichte sowie Spin-Gitter- (T_1) und Spin-Spin-Relaxationszeiten (T_2) durch geeignete Wahl der Parameter von Standard-Mess-sequenzen als mehr oder weniger kontrastbestimmend hervorgehoben. Insbesondere die Signalreduktion durch lokale Suszeptibilitäts-Inhomogenitäten wird vielfältig genutzt. Erwähnt seien hier z. B. der Nachweis älterer Blutungen, die Dichte trabekulären Knochens oder die Darstellung aktivierter Hirnareale (fMRI). Bei verschiedenen Ansätzen der Darstellung von Blutgefäßen, aber auch bei der Diffusionswichtung beeinflusst die Bewegung von Wasser relativ zu Magnetfeld-Gradienten oder zu mittels Hochfrequenz angeregten Regionen die Signalintensität.

Zunehmend breiter wird die Palette von Aussagen, die über die Gabe von Kontrastmitteln vor oder während einer MR-Untersuchung gewonnen werden kann. Durch geeignete Konstruktion von Kontrastmitteln kann deren Verteilungsmuster im Körper beeinflusst werden. Hier sind neben allgemeinen physiko-chemischen Eigenschaften wie z. B. der Molekül- bzw. Partikelgröße zunehmend spezifische Wechselwirkungen mit bestimmten Zellpopulationen (z. B. über Phagozytose) und/oder Oberflächen-Eigenschaften (z. B. Zellrezeptoren) von Bedeutung.

Paramagnetische Kontrastmittel auf der Basis von Gadolinium-Chelaten zeichnen sich durch gute Verträglichkeit aus. Durch ihren Einfluss auf die Relaxationszeiten T_1, T_2 und T_2^* ermöglichen sie gleichzeitig den Zugang zu verschiedensten klinisch relevanten Informationen. Der Schwerpunkt der vorliegenden Arbeit liegt auf der Analyse des Zeitverlaufes der Veränderung dieser Relaxationszeiten nach Bolus-Injektion solcher KM, dessen Modellierung sowie der Interpretation von Modellparametern und deren klinischer Relevanz. Übliche Näherungen (exponentielle Modellierung des Zerfalls der Quermagnetisierung durch Suszeptibilitäts-Inhomogenitäten, Vernachlässigung des systematischen Fehlers der Schätzung niedriger Signalintensitäten) werden durch Modellrechnungen gerechtfertigt. Gleichzeitig werden aufwändigere Bildnachbearbeitungs- bzw. Auswertungs-Algorithmen unter dem Aspekt der Stabilität bzw. Fortpflanzung von Messfehlern kritisch betrachtet. Im Rahmen der Bildgebung von Herzkranzgefäßen wird die KM-bedingte Signalintensitäts-Zunahme für eine Klasse von Messsequenzen durch Simulation optimiert und diese Optimierung messtechnisch umgesetzt.

7.2 Spezielle Aspekte

7.2.1 Kontrastmittelaufnahme

Sowohl Robustheit und einfache und schnelle messtechnische Zugänglichkeit als auch diagnostische Aussagekraft und die Übertragbarkeit von in der Computertomographie etablierten Denkweisen dürften dazu geführt haben, dass die qualitative Bewertung der Kontrastmittelaufnahme durch das untersuchte Gewebe am häufigsten von allen

kontrastmittel-gestützten kernspintomographischen Untersuchungsmethoden sein dürfte. In den letzten Jahren wurde die Spezifität dieses Ansatzes noch durch mehr oder weniger gewebespezifische Kontrastmittel erhöht. Auch die „molekulare Bildgebung“, bei der z. B. durch die Konjugation eines Kontrastmittels mit Antikörpern die Affinität zu bestimmten Geweben oder Gewebszuständen erhöht wird, fällt messtechnisch zum Teil in diesen Bereich.

Der Vergleich von Kontrastmittel-Konzentrationen zu einer bestimmten Zeit nach Bolus-Injektion erlaubt die Diagnostik von Perfusionsstörungen in unter physiologischen Bedingungen KM aufnehmendem Gewebe. Je nach Austausch-Geschwindigkeit zwischen Blutpool und Gewebe kann es dabei sinnvoll sein, KM-Konzentrations-Unterschiede vor, während oder auch nach Erreichung der Maximalkonzentration zu betrachten. Letzteres wird z. B. bei der Infarkt-Diagnostik am Myokard angewandt. Es wurde gezeigt, dass diese Ansätze mit dem gleichen Modell einer richtungsunabhängigen Diffusion zwischen Blutpool und Gewebe erklärbar sind.

Detailliertere Informationen sind über eine semiquantitative Beschreibung des Zeitverlaufes der KM-Aufnahme oder aber deren mathematische Modellierung und Kurvenanpassung zugänglich. Dieser Ansatz hat sich in der MR-Mammographie etabliert. Hier ist es möglich, bei diagnostisch ausreichender Zeitauflösung das gesamte Organ zu erfassen. Phänomenologische Beschreibungen des Intensitäts-Zeitverlaufes, der durch die KM-bedingte Verkürzung der T_1-Relaxationszeit hervorgerufen wird, sind von hohem klinischen Wert und durchaus akzeptabel, solange die Untersuchungsbedingungen konstant gehalten werden können. Verschiedene Ansätze mit empirischen bzw. pharmakokinetischen Modellen streben eine Objektivierung und Unabhängigkeit von den Messbedingungen an. Unabhängigkeit von Untersuchungs-bedingungen lässt sich hierbei nur erzielen, wenn zusätzliche Messungen von Intensitätsverläufen in großen Blutgefäßen einbezogen werden, was mindestens den Auswerteaufwand erhöht. Die Akzeptanz solcher Ansätze in der klinischen Routine ist nur gewährleistet, wenn dem erhöhten Aufwand ein spürbarer diagnostischer Gewinn gegenüber steht. Dies ist in der MR-Mammographie jedoch offenbar nicht gewährleistet. In der vorliegenden Arbeit wurden daher empirische Modelle zur Beschreibung der KM-Anflutung in Mamma-Läsionen entwickelt, in den Kontext pharmakokinetischer Modelle gestellt und klinisch eingesetzt. Da bei dynamischen Untersuchungen ein Kompromiss zwischen räumlicher und zeitlicher Auflösung eingegangen werden muss, wurden die hierbei gewonnenen Daten auch hinsichtlich der vom klinischen Aspekt minimal erforderlichen Zeitauflösung analysiert. Anlässlich der Inbetriebnahme neuer MR-Hardware wurde demonstriert, dass auch auf empirischen Modellen beruhendes statistisches Wissen zwischen verschiedenen Messbedingungen transferiert werden kann.

7.2.2 Perfusion

Der Begriff der Perfusion wird in der Literatur unterschiedlich benutzt. In dieser Arbeit wird dieser Begriff für die Blutversorgung von Gewebe, nicht aber für den Übertritt von Kontrastmittel in den Interzellularraum verwendet.

Am weitesten verbreitet dürften MR-Perfusionsmessungen im Sinne dieser Begriffs-bestimmung bei der Diagnostik des Hirninfarktes sein. Neben der klinischen Bedeutung dieser Fragestellung ist dafür insbesondere die Existenz der Blut-Hirn-Schranke verantwortlich. Sie sorgt dafür, dass Veränderungen der Relaxationszeiten in

Hirngewebe praktisch ausschließlich dem Kontrastmittel in den Blutgefäßen zugeschrieben werden können. Die Differenz der magnetischen Suszeptibilität zwischen kontrastmittel-beladenen Blutgefäßen und (KM-freiem) Gewebe führt dazu, dass in geeignet gewichteten Sequenzen der Signalabfall durch die T_2^*-Verkürzung groß gegenüber dem Signalzuwachs durch die gleichzeitige T_1-Verkürzung ist. Die damit gerechtfertigte Näherung T_1 = const erlaubt die Schätzung der Veränderung von T_2^* aus der Veränderung der nur einer Echozeit zuzuschreibenden Signalintensität. Mit von Haus aus stark T_2^*-gewichteten EPI-Sequenzen kann der gesamte Hirnschädel mit einer Zeitauflösung in der Größenordnung von < 1 Sekunde untersucht werden. Damit wird die Bestimmung von physiologischen Parametern wie einer mittleren Boluspassage-Zeit (MTT) sowie relativem Blutvolumen und –fluss prinzipiell möglich. Exakte Auswertungen setzen hier jedoch die Kenntnis einer Arteriellen Inputfunktion voraus. Fehlerquellen bei deren Bestimmung werden in dieser Arbeit analysiert.

Viele Gewebe nehmen auch unter physiologischen Bedingungen paramagnetische Kontrastmittel mit üblichen Molekülgrößen in das Interstitium auf. Die Annahme T_1 = const ist hier nicht zulässig. Bereits 1993 schlug der Autor vor, in diesen Fällen Doppelecho-Sequenzen einzusetzen [73]. In der vorliegenden Arbeit wurden verschiedene Korrekturansätze basierend auf Messungen mit Doppelecho-Sequenzen entwickelt, darunter zeitgleich mit Heiland [59] und Miyati [124] die Schätzung von T_2^* bei $T_1 \neq 0$ [76]. Unter Berücksichtigung des Einflusses einer T_1-Verkürzung auf die Intensität T_2^*-gewichteter Signale wurde die klinische Relevanz von aus dynamischen Doppelecho-Messungen abgeleiteten Parametern zur Beschreibung sowohl der interstitiellen KM-Aufnahme wie auch der Perfusion für verschiedene differentialdiagnostische Fragestellungen bei Hirntumoren untersucht.

In einem weiteren Schritt wurde neben dem Einfluss einer T_1-Verkürzung auf die Intensität $T_2^{(*)}$-gewichteter Signale auch der Beitrag interstitiell eingelagerten KMs zur T_2^*-Verkürzung berücksichtigt. Das hierfür entwickelte Modell erlaubt unter gewissen messtechnischen Voraussetzungen ebenso die Bereinigung der über die T_1-Abhängigkeit des MR-Signals beschriebenen interstitiellen KM-Konzentration vom intravasalen Anteil.

Am Beispiel einer Studie zu pharyngealen Tumoren unter Einschluss verschiedener MR-unabhängiger Parameter wurden Stabilität und klinische Relevanz einer Vielzahl von Kombinationen verschiedener Korrekturansätze analysiert. Hierbei erwies sich die Berücksichtigung der T_1-Verkürzung bei KM aufnehmenden Läsionen als essentiell. Zumindest unter den gegebenen messtechnischen Voraussetzungen überwogen bei der Berücksichtigung einer Arteriellen Inputfunktion die damit zusätzlich in das Ergebnis eingeführten Messfehler den potenziellen Nutzen. Unter Verzicht auf eine quantitative Vergleichbarkeit über unterschiedliche Messbedingungen hinweg erwiesen sich simple, modell-unabhängige Parameter wie insbesondere die maximale relative Änderung der Relaxationsrate R_2^* auf Grund ihrer Robustheit als für den klinischen Alltag besonders geeignet für die Bewertung der Mikrovaskularisation.

7.2.3 Bildgebung

Für Gradientenecho-Sequenzen mit einer Folge von RF-Impulsen innerhalb eines Anregungszyklus wurde der Verlauf der z-Magnetisierung in Abhängigkeit von der Spin-Gitter-Relaxationszeit untersucht. Mittels Computersimulation wurde nachgewiesen, dass bei einer Impulsfolge von 24 Impulsen die Berücksichtigung der T_1-Relaxation

zwischen den RF-Impulsen bei der Optimierung der Flipwinkel eine signifikante Erhöhung des Kontrast-Rausch-Verhältnisses zwischen Strukturen mit unterschiedlicher Spin-Gitter-Relaxationszeit erlaubt. Am Beispiel der KM-gestützten Darstellung von Herzkranzgefäßen wurde gezeigt, dass die Flipwinkel-Folge nicht auf die Spin-Gitte-Relaxationszeit der interessierenden Struktur selbst, sondern auf eine T_1-Zeit zwischen der des kontrastmittelhaltigen Blutes und des umgebenden Gewebes optimiert werden sollte.

8 Ausblick

8.1 Einige Charakteristika der bisherigen Entwicklung der bildgebenden medizinischen Diagnostik

Die Entwicklung der bildgebenden medizinischen Diagnostik macht sich eine zunehmende Zahl von physikalischen Effekten zunutze. Zum Einen werden vorhandene, mehr oder weniger statische bzw. stationäre Ortsabhängigkeiten in zwei- oder dreidimensionale Bildinformationen umgesetzt (z. B. Schwächung verschiedener Bereiche des elektromagnetischen Spektrums, elektrische Leitfähigkeit, Schallimpedanz). Zum Zweiten werden Signale, die mit physiologischen Vorgängen verbunden sind, direkt zur Darstellung gebracht (z. B. Thermographie, Elektro- bzw. Magneto-Enzephalographie). Schließlich werden physiologische Vorgänge genutzt, um physikalische Größen zu verändern und diese Veränderungen räumlich und ggf. zeitlich zu erfassen (z. B. Frequenzverschiebung von an bewegten Partikeln reflektierten Ultraschall-Wellen, Verteilung von endogenen oder exogenen Tracern (Desoxyhämoglobin oder Melanin als interne bzw. Kontrastmittel oder Substrate für verschiedenste Stoffwechsel-Vorgänge als externe Tracer)).

Im Mittelpunkt der Geräte-Entwicklung stehen hierbei die Verbesserung von räumlicher und zeitlicher Auflösung bei gegebenem oder zu verbesserndem Signal-Rausch-Verhältnis, (z. B. Detektor-Entwicklung, Mehrzeilen-Computertomographie, parallel MR imaging), die Erweiterung des Messbereiches in den Dimensionen von Raum, Zeit und gemessenem Parameter, zum Teil die Gewährleistung bzw. Verbesserung einer klaren Zuordnung gemessener Signale zu physikalischen Effekten (z. B. Entwicklung mehr oder weniger organspezifischer Kontrastmittel, molecular imaging, kombinierte Röntgenschwächungs- und Positronen-Emissions-Computertomographen) sowie die Prüfung weiterer physikalischer Größen bzw. Effekte auf ihre Eignung zur Erfassung physiologischer Vorgänge.

Die gerätetechnische Unterstützung der Kombination von auf unterschiedlicher physikalischer und/oder technischer Grundlage gewonnener Information mündet in zwei Entwicklungsrichtungen: Kombination von Methoden während der Untersuchung durch räumliche und technische Kopplung von Geräten (z. B. DSA und MRT, DSA und Ultraschall, PET und CT) bzw. offline-Kopplung mittels Image Fusion bis hin zur Interpretation fusionierter Bilder als multispektrale Darstellungen.

Nicht zu vernachlässigen sind hier auch Kosten-Nutzen-Aspekte – sowohl vom ökonomischen Standpunkt als auch bezüglich potenzieller oder determiniert auftretender Schädigungen des Patienten durch die Informations-Gewinnung (ionisierende Strahlung, Einbringung von körperfremden Substanzen oder Sonden bzw. Werkzeugen). Letzteres führte in jüngerer Vergangenheit beispielsweise zum Rückgang der Bedeutung der Digitalen Subtraktions-Angiographie (DSA) als diagnostische Methode. Die Darstellung der Blutgefäße ist durch gerätetechnische Entwicklungen sowohl der CT als auch der MRT ohne Einbringung von Kathetern in immer besserer Qualität möglich geworden. Gleichzeitig stehen in zunehmendem Maße therapeutische Methoden unter Anwendung der DSA-Technik zur Verfügung, so dass eine Wandlung der DSA von einem diagnostischen zu einem therapeutischen Werkzeug beobachtet werden kann. Die Integration von Diagnostik und Therapie oder von bildgebender (d.h. nichtinvasiver) und invasiver Diagnostik (Gewinnung von Gewebeproben für ex-vivo-

Diagnostik) äußert sich auch als bildgebungs-gestützte Punktion (Ultraschall, CT, MRT) oder – etwas weiter gefasst – in der Nutzung prä- oder intraoperatiav erzeugter Bilder bei der Anwendung computergestützter Operationsmethoden (Ultraschall, MRT). Diese Integration generiert wiederum Anforderungen an die Entwicklung interventioneller Werkzeuge (OP-Instrumente, Katheter, implantierbare Materialien), die mit den einzusetzenden bildgebenden Verfahren kompatibel sind. Sie müssen sich also mit der jeweiligen Methode darstellen lassen und dürfen höchstens in begrenztem Maße Bildartefakte verursachen.

Das Bestreben, Eingriffe in die Integrität des Patienten zu vermeiden (also nichtinvasive Diagnostik zu betreiben), konkurriert mit der Qualität der gewonnenen diagnostischen Information. So wurde die Time-of-Flight-Angiographie als vollständig nichtinvasives kernspintomographisches Verfahren mit der Verfügbarkeit hinreichend schneller Mess-Sequenzen in den letzten Jahren durch die Bolus-Injektion von Kontrastmittel unmittelbar vor bzw. während der Messung modifiziert, womit ein erheblich besseres Kontrast-Rausch-Verhältnis zwischen Gefäßen und umgebendem Gewebe erzielt werden kann (neben weiteren, die Invasivität der Methode nicht betreffenden Modifikationen).

Einen weiteren Aspekt stellt das Wechselspiel zwischen qualitativer und quantitativer Erfassung physiologischer Vorgänge dar. Wiederholt wurde ausgehend von der quantitativen Bestimmung eines physikalischen Parameters zu qualitativen Darstellungen übergegangen. Beispiele hierfür sind die Messung von Zerfalls-Aktivitäten radioaktiver Tracer, die Doppler-Verschiebung von Ultraschallwellen, aber auch die magnetische Kernresonanz. Ebenso kann aber die umgekehrte Entwicklungsrichtung beobachtet werden. Hierzu zählen u.a. die CT- oder MR-basierte Quantifizierung von Blutflüssen und Perfusionsparametern, die MR-Relaxometrie (deren Zenit allerdings überschritten sein dürfte), in-vivo-MR-Spektroskopie oder die nuklearmedizinische Bestimmung von Nierenfunktionsparametern. Im Unterschied zur ingenieurtechnischen oder naturwissenschaftlichen Denkweise tritt bei der medizinischen Diagnostik die Bedeutung des Verständnisses kausaler Zusammenhänge gegenüber einer praktikablen Informationsgewinnung für empirisch erfassbare Konsequenzen bezüglich Diagnose, Therapie und/oder Prognose des Patienten zurück. Das Verhältnis von messtechnischem und zeitlichem Mehraufwand einerseits und Konsequenz für den Patienten andererseits entscheiden über die klinische Relevanz neuer Methoden. Unter diesem Blickwinkel sind zum Teil methodisch weniger genaue Verfahren solchen überlegen, die zwar exakter fassbare Beziehungen zwischen Eingangs- und Ausgangssignal aufweisen, deren Mehraufwand aber in einem ungünstigen Verhältnis zum diagnostischen Zugewinn steht. Als klassisch hierfür darf die Entwicklung schneller bildgebender Sequenzen in der Kernspintomographie erwähnt werden. Der beachtlichen Beschleunigung der Bilderzeugung durch das Turbo-Prinzip (vgl Abschnitt 2.2.2.4) steht hier eine für den einzelnen Bildpunkt weniger scharf definierte Wichtung bezüglich der $T_2^{(*)}$-Relaxationszeit gegenüber.

In diesen Problemkreis ordnen sich auch wesentliche Teile der vorliegenden Arbeit ein. Der deduktiven oder empirischen Formulierung von Modellen und Korrekturalgorithmen bei der Beschreibung von Eigenschaften der Gewebs-Vaskularisation auf der Grundlage kontrastmittel-gestützter MR-tomographischer Messungen wird hier die Bestimmung und kritische Analyse von deren klinischer Wertigkeit gegenübergestellt.

8.2 Zukünftige Entwicklung: Versuch einer Prognose:

Nach den Vorstellungen des Autors könnte die zukünftige Entwicklung der bildgebenden medizinischen Diagnostik geprägt sein von einem Spannungsfeld zwischen Verallgemeinerung und Spezialisierung:

Einerseits wachsen die Möglichkeiten speziell einiger für den Patienten besonders schonender Verfahren, verschiedenste Fragestellungen zu beantworten. Speziell die Kernspintomographie wegen der Vielzahl der hier direkt oder indirekt wirksamen physikalischen Größen und die Sonographie wegen ihrer technisch vergleichsweise leichten Realisierbarkeit scheinen hier zukunftsträchtig zu sein.

Andererseits erscheint es möglich, für spezielle Fragestellungen Geräte mit technischen Parametern zu entwickeln, die für „gesamtradiologisch“ einsetzbare Maschinen aus physikalischen Gründen nicht erreichbar sind. Auch hier könnte die Kernspintomographie ein erhebliches, gegenwärtig noch nicht eröffnetes Entwicklungspotenzial aufzuweisen. Starke, homogene Magnetfelder (B_0) dürften sich an kleineren Magneten deutlich leichter realisieren lassen als an Ganzkörper-Magneten – entsprechende Erfahrungen aus der chemischen Analytik liegen vor. Die gegenwärtig auf dem Markt befindlichen Spezialgeräte – speziell für orthopädische Fragestellungen – sind mit eher niedrigen Feldstärken ausgestattet und lassen damit Wünsche in Bildqualität und/oder Performance offen.

Zum Dritten vertritt der Autor die Auffassung, dass verschiedenen Methoden ineinander übergehen werden. Im Bereich des konventionellen Röntgens wird durch den Einsatz von Flachbild-Detektoren („Direktradiographie“) in naher Zukunft der Unterschied zwischen Aufnahme- und Durchleuchtungs-Geräten verschwinden. Die gegenwärtige Tendenz zu einer größeren Zahl von Detektor-Reihen in der Computertomographie könnte in den Einsatz von Flachbild-Detektoren auch bei dieser Methode münden (mehrere Medizingeräte-Hersteller forschen bereits in dieser Richtung). Damit könnte sich der Übergang zwischen Aufnahme und Durchleuchtung (Projektionsradiographie) einerseits und Computertomographie als Schnittbildverfahren andererseits auf die Art der Bewegung von Strahlenquelle und Detektor im Raum sowie die Nachverarbeitung der gewonnenen Messwerte reduzieren. Voraussetzung ist hier jedoch noch die Steigerung der ausles- und verarbeitbaren Datenraten um mehrere Größenordnungen. Sowohl aus dieser Überlegung heraus als auch durch die Entwicklung der Kernspintomographie als „Konkurrenzmethode“ ohne Exposition des Patienten mit ionisierender Strahlung erscheint damit für die Weiterentwicklung der Computertomographie als gesonderte Methode ein endliches Zeitfenster zu bestehen – trotz des durch die Entwicklung der Multislice-CT gegenwärtig verursachten Booms dieser Methode.

Verbesserungen von Bildqualität, räumlicher oder zeitlicher Auflösung bei auf Röntgenstrahlen beruhenden bildgebenden Verfahren dürften um maximal noch den Faktor 2 bis 5 zu erwarten sein. Gegenwärtig verfügbare Flachbild-Detektoren erreichen Quantenausbeuten oberhalb von 70 % bei einer Ortsfrequenz von 0 und im zweistelligen Prozentbereich bei der durch die Größe der Detektorelemente gegebenen Nyquist-Frequenz. Eine Erhöhung dieser Grenzfrequenz erscheint technisch nicht ausgeschlossen. Sie dürfte jedoch wegen einerseits sinnvoller Beschränkungen der maximal auf den Patienten anzuwendenden Dosis und andererseits des bei gegebener Dosis zur Wurzel der Pixelfläche proportionalen Signal-Rausch-Verhältnisses eine praktikable Grenze etwa beim Doppelten der jetzt mit Film-Folien-Systemen

erreichbaren Ortsauflösung erreichen, also in der Größenordnung von etwa 20 Linienpaaren pro Millimeter.

Bei der Kernspintomographie könnte sich die Tendenz zu höheren Feldstärken noch fortsetzen. Dafür sprechen die Vorteile hoher Feldstärken bezüglich Signal-Rausch-Verhältnis und bezüglich auf Spin-Dephasierung beruhender Methoden (Darstellung von Suszeptibilitäts-Unterschieden (Perfusion, fMRI, Strukturparameter z. B. im Knochenmark), Difussion). Die in-vivo-MR-Spektroskopie profitiert von hoher Feldstärke zusätzlich durch die Spreizung der Spektren über einen größeren Frequenzbereich, was die Zahl der nachweisbaren Metaboliten erhöhen und deren Nachweisgrenze senken dürfte. Limitierend für die Bildgebung bei hoher Feldstärke wirkt die mit der Feldstärke zunehmende Radiofrequenz-Energie, die zur Herbeiführung einer Spin-Inversion erforderlich ist, und die nicht zu einer unzulässigen Erwärmung des Körpers oder von Körperteilen führen darf (SAR-Limit). Ebenso nimmt die Anfälligkeit der Methode für verschiedene Artefakte mit der Feldstärke zu. Eine drastische Steigerung der Gradienten-Feldstärken und -schaltgeschwindigkeiten ist ebenfalls nicht zu erwarten, da zumindest bei Ganzkörper-Systemen bereits die Schwelle der Nervenstimulation durch induzierte Spannungen erreicht wird. Hier könnten die oben erwähnten kleinen Systeme für spezielle Fragestellungen prinzipielle Vorteile zeigen, da die maximale Änderungsgeschwindigkeit des Magnetfeldes bei gegebener Gradientenstärke proportional zur räumlichen Ausdehnung der Gradientenspulen ist.

Entwicklungspotenzial könnte bei der Kernspintomographie in einer weiteren Effektivierung der Signalgewinnung liegen. Der Autor hält es für möglich, dass die mit der Einführung von phased-array-Spulen begonnene und in den letzten Jahren mit den Methoden des „parallel imaging“ fortgesetzte Tendenz weitere Innovationen hervorbringen könnte. Schließlich dürften weitere Fortschritte in der spezifischen Hervorhebung ausgewählter biologischer bzw. biochemischer Strukturen zu erwarten sein. Hier erscheint dem Autor insbesonder die Bindung von Tracern an ausgewählte Rezeptoren auf Zelloberflächen zukunftsträchtig. Allerdings müssen hier weitere Mechanismen der Signalbeeinflussung gefunden werden, die die mit diesem Ansatz zu erwartenden geringen Tracer-Anreicherungen mit hinreichendem Kontrast-Rausch-Verhältnis zur Darstellung bringen. Bezüglich dieser Nachweisgrenzen sind im Moment nuklearmedizinische Ansätze im Vorteil. Mit dem Einzug höherer Magnetfeldstärken in den klinischen Alltag könnte die Tracer-Verfolgung in Zukunft größeres Gewicht in der MR-Spektroskopie gewinnen.

Generell werden die technischen Hintergründe der Bilderzeugung und –nachverarbeitung immer komplexer und damit für den medizinischen Anwender in der klinischen Routine immer schwerer beherrschbar. Daraus leitet sich die Forderung nach intelligenten Mensch-Maschine-Schnittstellen ab. Standard-Protokolle müssen gewährleisten, dass sich der Anwender auf seine eigentliche Aufgabe – nämlich die Formulierung und Beantwortung von diagnostischen Fragestellungen sowie ggf. therapeutische Maßnahmen – konzentrieren kann und auch ohne wesentliche Nutzer-Eingriffe eine adäquate Bilderzeugung bzw. sonstige Informationsgewinnung (z. B. MR-Spektroskopie) gewährleistet ist. Die Bereitstellung von Standard-Protokollen für viele Fragestellungen wiederum dürfte die Notwendigkeit einer Anwesenheit des Methoden-Spezialisten vor Ort verringern. Damit wäre schließlich eine Tendenz zu zentralisierter, zumindest aber zu räumlich vom Untersuchungsort getrennter Befundung aus ökonomischen Gründen wahrscheinlich – unabhängig davon, ob dies der Entwicklung einer vertrauensvollen Arzt-Patienten-Beziehung dienlich ist.

Anhang

Literaturverzeichnis

1 Anderson PW, Weiss PR. Exchange narrowing in paramagnetic resonance. Rev Med Phys 1953: 25: 269-276

2 Anderson SA, Rader RK, Westlin WF, Null C, Jackson D, Lanza GM, Wickline SA Kotyk JJ. Magnetic Resonance contrast enhancement of neovascualture with alpha(v)beta(3)-targeted nanoparticles. Magn Reson Med 2000: 44: 433-439

3 Antman S. Foundations of Indicator-Dilution Theory. In Bloomfield D, (Ed.). Dye curves: the theory and practice of indicator dilution. University Park Press Baltimore, 1974, 21-40

4 Aronen HJ, Boxerman JL, Goldberg IE, Weisskoff RM, Belliveau JW, Provenzale JM, Vevea JM, Calder CM, Campbell TA, Brady TJ, Rosen BR. Susceptibility-contrast CBV imaging: Optimization of contrast dose and imaging sequences. SMRM, 11th Scientific Meeting, Book of Abstracts 1992: 11: 1129

5 Aronen HJ, Gazit IE, Louis DN, Buchbinder BR, Pardo FS, Weisskoff RM, Harsh GR, Cosgrove GR, Halpern EF, Hochberg FH et al. Cerebral blood volume maps of gliomas: comparison with tumor grade and histologic findings. Radiology 1994 Apr;191(1):41-51

6 Axel L. Methods using blood pool tracers. In: Le Bihan D. (Ed.) Diffusion and Perfusion Magnetic Resonance Imaging. Applications to Functional MRI. New York 1995 ISBN 0-7817-0244-5

7 Balaban RS, Ceckler TL. Magnetization Transfer Contrast in Magnetic Resonance Imaging. Magnetic Resonance Quarterly 1992: 8: 116-137

8 Baumann, M; Appold,S; Zimmer, J; Scharf, M; Beuthien-Baumann,B; Dubben, HH; Enghardt, W; Schreiber, A; Eicheler, W; Petersen,C: Radiobiological hypoxia, oxygen tension, interstitial fluid pressure and relative viable tumour area in two human squamous cell carcinomas in nude mice during fractionated radiotherapy. Acta Oncol. 2001:40:519-28.

9 Beier J. Computerunterstützte Bildauswertung für radiologische Diagnostik und bildgesteuerte Therapie. Shaker Verlag Aachen 2001. ISBN 3-8265-9367-7

10 Benner T, Heiland S, Erb G, Forsting M, Sartor K. Accuracy of gamma-variate fits to concentration-time curves from dynamic susceptibility-contrast enhanced MRI: influence of time resolution, maximal signal drop and signal-to-noise. Magn Reson Imaging 1997: 15: 307-17

11 Berg D, Supprian T, Hofmann E, Zeiler B, Jäger A, Lange KW, Reiners K, Becker T, Becker G. Depression in Parkinson’s disease brainstem midline alteration on trancranial sonography and magnetic resonance imaging. J Neurol 1999: 246: 1186-1193

12 Bloembergen N, Purcell E, Pound RV. Relaxation effects in nuclear magnetic resonance absorption. Phys Rev 1948: 73: 679-712

13 Böck JC, Henrikson O, Götze AHG, Wlodarczyk W, Sander B, Felix R. Magnetic resonance perfusion imaging with Gadolinium-DTPA. A quantitative approach for the kinetic analysis of first-pass residue curves. Invest Radiol. 1995: 30: 693-699.

14 Böck JC: Cerebral Perfusion Imaging.
In: Felix R, Heshiki A, Hosten N, Hricak H: Magnevist. London 1994 ISBN 0 86542 836 0

15 Botnar RM, Stuber M, Danias PG, Kissinger KV, Manning WJ. A fast 3D approach for coronary MRA. J Magn Reson Imaging 1999:10:821-825

16 Boxerman JL, Hamberg LM, Rosen BR, Weisskoff RM. MR Contrast due to intravascular magnetic susceptibility perturbations. MRM 1995: 34: 555-566

17 Brix G, Semmler W, Port R, Schad LR, Layer G, Lorenz W. Pharmacokinetic parameters in CNS Gd-DTPA enhanced MR imaging. J Comput Assist Tomogr 1991:15:621-627

18 Brix G, Schreiber W, Hoffmann U, Guckel F, Hawighorst H, Knopp MV Methodische Ansätze zur quantitativen Beurteilung der Mikrozirkulation im Gewebe mit der dynamischen Magnetresonanztomographie. Radiologe. 1997 37: 470-80

19 Bronstein IN, Semendjajew KA. Taschenbuch der Mathematik. Moskau, Leipzig 1979

20 Brown G. The Adelaide MRI Website. → MRI Papers → Fat Suppression techniques. http://www.users.on.net/vision/index.htm (Stand 10.09.2002)

21 Calvo BF, Semelka RC. Beyond anatomy: MR imaging as a molecular diagnostic tool. Surg Oncol Clin N Am 1999: 8: 171-183

22 Cendes F, Caramanos Z, Andermann F, Dubeau F, Arnold DL. Proton magnetic resonance spectroscopic imaging and magnetic resonance imaging volumetry in the lateralization of temporal lobe epilepsy: a series of 100 patients. Ann Neurol 1997: 42: 737-746

23 Clarke SE, Weinmann HJ, Dai E, Lucas AR, Rutt BK: Comparison of two blood pool contrast agents for 0.5-T MR angiography: experimental study in rabbits. Radiology 2000:2:787-794

24 Daldrup H, Shames DM, Wendland M, Okuhata Y, Link TM, Rosenau W, Lu Y, Brasch RC. Correlation of dynamic contrast-enhanced magnetic resonance imaging with histologic tumor grade: comparison of macromolecular and small-molecular contrast media. Pediatr Radiol 1998: 28: 67-78

25 Dannert S, Krück W, Schick F, Claussen CD, Müller-Schimpfle M. MR-tomographische Charakterisierung suspekter Mammabefunde mittels suszeptibilitätsgewichteter T_2^*-Sequenzen. Fortschr Röntgenstr 2001:173: 38-43

26 Demsar F, Roberts TPL, Schwickert HC, Shames DM, van Dijke CF, Mann JS, Saeed M, Brasch RC. A MRI Spatial Mapping Technique for Microvascular Permeability and Tissue Blood Volume Based on Macromolecular Contrast Agent Distribution. MRM 1997:37:236-242

27 den Boer JA, Hoenderop RKKM, Smink J, Dornseiffen G, Koch PWAA, Mulder JH, Slump CH, Volker EDP, de Vos RAI. Pharmacokinetic analysis of Gd-DTPA enhancement in dynamic three-dimensional MRI of breast lesions. JMRI 1997: 7: 702-715

28 Denekamp J. Angiogenesis, neovascular proliferation and vascular pathophysiology as targets for cancer therapy. Br J Radiol 1993:66:181-186

29 Dong Q, Hurst DR, Weinmann HJ, Chenevert TL, Londy FJ, Prince MR: Magnetic resonance angiography with gadomer-17. An animal study original investigation. Invest Radiol 1998:33:699-708

30 Dubben HH, Thames HD, Beck-Bornholdt HP. Tumor volume: a basic and specific response predictor in radiotherapy. Radiother Oncol. 1998: 47:167-174

31 Duvvuri U, Roberts DA, Leigh JS, Bolinger L. Magnetization transfer imaging of the brain: A quantitative comparison of results obtained at 1.5 and 4.0 T. J Magn Reson Imaging 1999 Oct;10(4):527-532

32 Egelhof T, Essig M, v. Kummer R, Dörfler A, Winter R, Sartor K. Der akute ischämische Hirninfarkt: Eine prospektive, serielle Untersuchung mit der Magnetresonanztomographie. Fortschr Röntgenstr 1998: 168: 222-227

33 Elster AD. An index system for comparative parameter weighting in MR imaging. J Comput Assist Tomogr 1988: 12:130-134

34 Embacher F, Oberhuerner P. math online: Maths links and online tools. http://www.univie.ac.at/future.media/moe/onlinewerkzeuge.html (20.10.2002)

35 Emerson JF, Chen PC, Shankle WR, Haier RJ, Nalcioglu O. Data analysis for dynamic contrast-enhanced MRI-based cerebral perfusion measurements: correcting for changing cortical CSF volumes. MAGMA 1995: 3: 41-48

36 Filippi M, Rovaris M, Capra R et al. A multi-centre longitudinal study comparing the sensitivity of monthly MRI after standard and triple dose gadolinium-DTPA for monitoring disease activity in multiple sclerosis. Implications for phase II clinical trials. Brain 1998:121:2011-2020

37 Fischer HW, Rinck PA, van Haverbeke Y, Muller RN. Nuclear relaxation of human brain grey and white matter – Analysis of field dependence and implications for MRI. Magn Reson Med 1990: 16: 317-334

38 Folkman J. The role of angiogenesis in tumor growth. Semin Cancer Biol 1992: 3: 65-71

39 Franke, W.-G.; Schmitt, T.; Andreeff, M.; Oehme, L.; Freyer, R. A new method for tumor volume quantitation with SPECT based on a fuzzy segmentation technique. In: Lemke HU. (Ed.): CAR"98", Procceedings of the 12th International Symposium, Int.Congress Series, Amsterdam: Elsevier, 1998. S. 188-193.

40 Freyer R: Bildtechnik in der Medizin. Biomedizinische Technik 1998: 43Suppl1 8

41 Friebolin H. Ein- und zweidimensionale NMR-Spektroskopie. Eine Einführung. VHC Weinheim1992. ISBN 3-527-28507-5

42 Friedlinger M, Schroder J, Schad LR. Ultra-fast automated brain volumetry based on bispectral MR imaging data. Comput Med Imaging Graph 1999: 23: 331-337

43 Gan Y, Sasai T, Nishiyama H, Ma X, Zhang Z, Fuchihata H. Magnetic resonance imaging of human mandibular elevator muscles after repetitive maximal clenching exercise. Archs Oral Biol 2000: 45: 247-251

44 Gerber BL, Garot J, Bluemke DA, Wu KC, Lima JA. Accuracy of contrast-enhanced magnetic resonance imaging in predicting improvement of regional myocardial function in patients after acute myocardial infarction. Circulation 2002: 106:1083-1089

45 Gerweck, L.E.: Tumor pH: Implications for treatment and novel drug design. Radiation Oncology 1998:8:176-182

46 Gore JC, Kennan RP. Contrast Agents and Relaxation Effects. In Atlas SW (ed.). Magnetic Resonance Imaging of the Brain and Spine. Lippincott-Raven Publishers Philadelphia 1966. ISBN 0-7817-0282. 89 – 107

47 Gore JC, Kennan RP. Contrast Agents and Relaxation Effects. In: Atlas SW (Ed.)´Magnetic Resonance Imaging of the Brain and Spine. Lippincott – Raven Philadelphia. ISBN 0-7817-0282-8

48 Grabenbauer, GG; Steininger, H; Meyer, M: Nodal CT density and total tumor volume as prognostic factors after radiation therapy of stage III/IV head neck cancer. Radiother. Oncol. 1998:47:175-183

49 Griftin TW; Pajak TF; Gillespie BW. Predicting the response of head and cancers to radiation therapy with a multivariate modeling system: an analysis of the RTOG head and neck registry. Int. J. Radiat.Oncol.Biol.Phys. 1984:10:481-487

50 Gückel F, Brix G, Schmiedek P, Piepgras A, Rempp K, Kopke J, Lammler B, Georgi M. Nichtinvasive Quantifizierung des zerebralen Blutvolumens und Blutflusses mit der dynamischen MR-Tomographie. Untersuchungen an Probanden und Patienten mit zerebrovaskularer Insuffizienz. Radiologe 1995: 35: 791-800

51 Günther H. NMR-Spektroskopie. Grundlagen, Konzepte und Anwendungen der Protonen- und Kohlenstoff-13-Kernresonanz-Spektroskopie in der Chemie.Thieme-Verlag Stuttgart 1992. ISBN 3-13-487503-9

52 Haacke EM, Brown RW, Thompson MR, Venkatesan R. Magnetic Resonance Imaging: Physical Principles and Sequence Design. Wiley-Liss New York 1999. ISBN 0-471-35128-8

53 Hackstein N, Puille MF, Bak BH, Scharwat O, Rau WS. Measurement of single kidney contrast media clearance by multiphasic spiral computed tomography: preliminary results. Eur J Radiol 2001: 39: 201-208

54 Hagen T, Bartylla K, Piepgras U. Correlation of regional cerebral blood flow measured by stable xenon CT and perfusion MRI. J Comput Assist Tomogr 1999: 23: 257-64

55 Hall EJ.Radiobiology for the radiologist. Lippincott, Philadelphia 1994

56 Hänig, V., Klengel, St.; Hietschold, V.; Platzbecker, H.; Köhler, K. Embolisation von Meningeomen und Glomustumoren und Therapiemonitoring mit kernspin-tomographischen Perfusionsstudien. Fortschr. Röntgenstr. 164 (1996) S22 (77. Deutscher Röntgenkongreß 1996 – Abstracts)

57 Haselhorst R, Kappos L, Bilecen D, Scheffler K, Möri D, Radü EW, Seelig J. Dynamic Susceptibility Contrast MR Imaging of Plaque Development in Multiple Sclerosis: Application of an Extended Blood-Brain Barrier Leakage Correction. J Magn Reson Imaging. 2000:11: 495-505

58 Hebrank FX, Gebhardt M. SAFE Model – A New Method for Predicting Peripheral Nerve Stimulations in MRI. International Society of Magnetic Resonance in Medicine, 8th Annual Meeting, 01. – 07.04.2000 Denver. Poster 2007

59 Heiland S, Benner T, Rempp K, Reith W, Forsting M, Sartor K: Ein Verfahren zur simultanen Beurteilung der Hämodynamik und der dynamischen Kontrastmittel-anreicherung bei zerebralen Läsionen mit gestörter Blut-Hirn-Schranke. Fortschr Röntgenstr. 1997; 166: S137

60 Heiland S, Hartmann M, Sartor K. Perfusions-MRT bei gestörter Blut-Hirn-Schranke: Fehlerquellen und Lösungsansätze. Fortschr Röntgenstr 2000: 172: 812-816

61 Heiland S, Sartor K. Magnetresonanztomographie beim Schlaganfall – Methodische Grundlagen und klinische Anwendung. Fortschr Röntgenstr 1999: 171: 3-14

62 Helpern JA, Ordidge RJ, Knight RA, Jiang Q. Brain Ischemia: Mechanisms and □redictive value of the decrease in water diffusion in cerebral ischemia in: Le Bihan D. (Ed.) Diffusion and Perfusion Magnetic Resonance Imaging. Applications to Functional MRI. New York 1995 ISBN 0-7817-0244-5

63 Henderson E, Rutt BK, Lee TY. Temporal sampling requirements for the tracer kinetics modeling of breast disease. Magnetic Resonance Imaging 1998: 16: 1057-1073

64 Henriksen O, de Certaines JD, Spisni A, Cortsen M, Muller RN, Ring PB. In vivo field dependence of proton relaxation times in human brain, liver and skeletal muscle: a multicenter study. Magn Reson Imaging 1993: 11: 851-856

65 Heywang SH, Hilbertz T, Pruss E, Wolf A, Permanetter W, Eiermann W, Lissner J. Dynamische Kontrastmitteluntersuchungen mit FLASH bei Kernspintomographie der Mamma. Digitale Bilddiagn 1988 Mar;8(1):7-13

66 Heywang SH, Wolf A, Pruss E, Hibertz T, Eiermann W, Permanetter W. MR imaging of the breast with Gd-DTPA: Use and limitations. Radiology 1989: 171: 95-103

67 Hietschold V, Abolmaali N, Kittner T. Contrast enhanced MR imaging of coronary arteries: optimization of the flip angle train in a navigator sequence. In: Computer Assisted Radiology and Surgery. Eds.: Lemke HU, Inamura K, Doi K, Vannier MW, Farman AG. San Francisco, 28.06. – 01.07.2000. Elsevier Amsterdam 2000

68 Hietschold V, Abolmaali N, Kittner T. MRI of the coronary arteries: flip angle train optimization for 3D sequences. Z Med Phys 12 (2002) 177-181

69 Hietschold V, Förster A, Abolmaali N, Kittner T, Schreiber M, Köhler K: Dynamic Mamma-MRI: conversion of statistical diagnostic knowledge to different conditions concerning field strength, sequence and model function. Computer Assisted Radiology, Paris 23-26 June 1999. Computer Assisted Radiology and Surgery. Eds: H.U. Lemke, M.W. Vannier, k. Inamura, A.G. Farman. ELSEVIER Amsderdam 1999 144-147 ISBN 0 444 50290 4

70 Hietschold V, Kittner T, Appold S, Abolmaali N, Laniado M. MR Perfusion Measurement of Contrast Uptaking Lesions: Consideration of T_2^* Shortening due to Interstitial Contrast Agent. Rofo Fortschr Geb Rontgenstr Neuen Bildgeb Verfahr 2002: 174: 973-978

71 Hietschold V, Kittner T, Mucha D. Magnetic resonance perfusion imaging: Pros and cons of sophisticated image postprocessing. Abstracts of the 13th European Congress of Radiology, Vienna, 02-6 March 2001. Eur. Radiol. 11 (2001)Suppl.1 147.

72 Hietschold V, Klengel S, Köhler K. Influence of tumor contrast uptake upon T_2^*-based rCBV estimation in brain lesions. 9th European Congress of Radiology Wien 05.03. – 10.03.1995; Book of Abstracts S116, #1174

73 Hietschold V, Klengel S, Köhler K: Simultaneous Dynamic Measurement of Tissue Contrast Enhancement and Perfusion at 0.5 Tesla: Method and Postprocessing. SMRM 11th Annual Meeting 1993 New York 16. – 20. 8. 1993; Book of Abstracts p. 619 ISSN 1065-9889

74 Hietschold V, Klengel S, Neumann U, Kaulen F, Schreiber M, Köhler, K. Quantitative dynamic MRI of the breast: Modelling and determination of minimum temporal resolution. Proceedings of the Fourth Scientific Meeting of the ISMRM, New York, 27. 4. – 3. 5. 1996 S. 750

75 Hietschold V, Klengel S, Neumann U, Köhler K.Simultaneous Dynamic MRI of Contrast Enhancement and Perfusion Induced Signal Loss at a 0.5 Tesla Routine Scanner: Method and Postprocessing. Proceedings 11th Annual Congress of the European Society for Magnetic Resonance in Medicine and Biology, Wien 20. – 24. 04. 1994

76 Hietschold V, Klengel S,Kaulen F, Neumann U,Köhler K: Analyse der diagnostischen Wertigkeit verschiedener Parameter bei der dynamischen Doppelecho-MRT von Hirnläsionen. Fortschr. Röntgenstr. 1997; 166: S136

77 Hietschold V, Klengel St, Köhler K. Quantitative MR mammography: Model function and determination of minimal temporal resolution. In: Lemke HU, Inamura K, Jaffe CC, Vannier MW (Eds.): Computer Assisted Radiology. Springer Berlin 1995; p. 1

78 Hietschold V, Schwensow S, Kittner T, Müller A, Köhler K: Simultaneous measurement of contrast uptake and bolus passage in MRI: Measurement, separation of effects and diagnostic consequences. Computer Assisted Radiology, Paris 23-26 June 1999
Computer Assisted Radiology and Surgery.
Eds: H.U. Lemke, M.W. Vannier, k. Inamura, A.G. Farman.
ELSEVIER Amsderdam 1999 134-138 ISBN 0 444 50290 4

79 Hietschold V, Schwensow S, Müller A, Kittner T, Köhler K: Simultane Messung von KM-Aufladung und Bolus-Passage: Separation der Effekte. Abstracts des 80. Deutschen Röntgenkongresses 12-15 Mai 1999. Fortschr. Röntgenstr. 170 (1999) S123

80 Hietschold V., Kittner T., Abolmaali N. Quantitative MR perfusion imaging of pharyngeal tumors: Is there additional information available from the time course of T2*? Abstracts of the 14th European Congress of Radiology, Vienna, 01-05 March 2002
Eur. Radiol. 12 (2002)Suppl.1 193

81 Higgins CB, Hricak H, Helms CA: Magnetic Resonance Imaging of the Body. Philadelphia 1996 ISBN 0-397-51711-4

82 Höckel M, Vaupel P (1998). The prognostic significance of hypoxia in cervical cancer: A radiobiological or tumor biological phenomenon . In: Molls M, Vaupel P (eds) Blood perfusion and microenviroment of human tumors.Springer Verlag Berlin-Heidelberg 74-79

83 Hoehn-Berlage M, Bockhorst K. Quantitative magnetic resonance imaging of rat brain tumors: In vivo NMR relaxometry for the discrimination of normal and pathological tissues. Technology and Health Care 1994: 2: 247-254

84 Hoffmann U, Brix G, Knopp MV, Heß T, Lorenz WJ Pharmacokinetic Mapping of the Breast: A new Method for Dynamic MR Mammography. MRM 1995: 33: 506-514

85 Hofman MB, Henson RE, Kovacs SJ et al. Blood pool agent strongly improves 3D magnetic resonance coronary angiography using an inversion pre-pulse. Magn Reson Med 1999:41:360-367

86 Högemann D, Basilion JP. „Seeing inside the body“: MR imaging of gene expression. Eur J Nucl Med 202: 29: 400-408

87 Homans SW. A Dictionary of Concepts in NMR. Clarendon Press. Oxford 1995. ISBN 0-19-854765-X (Pbk).

88 Huang Y, Majumdar S, Genant HK, Chan WP, Sharma KR, Yu P, Mynhier M, Miller RG. Quantitative MR relaxometry study of muscle composition and function in Duchenne muscular dystrophy. JMRI 1994: 4: 59-64

89 Jackson A. Perfusion MRI evolves in imaging brain disease. Diagnostic Imaging Europe 1999: 3: 25-32

90 Jackson GD, Kim SE, Fitt GJ, Mitchell AL, Syngeniotis A. Hippocampal T_2 abnormalities correlate with antecedent events and help predict seizure intractability. Dev Neurosci 1999: 21: 200-206

91 Joseph PM. Principles of Image Formation. In: Atlas SW (Ed.)´Magnetic Resonance Imaging of the Brain and Spine. Lippincott – Raven Philadelphia. ISBN 0-7817-0282-8

92 Kaiser WA, Zeitler E. MR-Imaging of the breast: Fast imaging sequences with and without Gd-DTPA. Radiology 1989: 170: 681-686

93 Kaiser WA. MR-Mammographie – eine kritische Bestandsaufnahme. Fortschr Röntgenstr 1996: 165: 425-427.

94 Kanzow G, Pries AR, Gaehtgens P. Analysis of the hematocrit distribution in the mesenteric microcirculation. : Int J Microcirc Clin Exp 1982:1:67-79

95 Kaulen F, Hietschold V, Klengel S, Neumann U, Köhler K. MRT-gestützte Tumorvolumenbestimmung des Osteosarkoms im Kindesalter: erste Verlaufsbeobachtung bei der Anwendung einer schnittbildgestützten Volumenberechnung. 31. Jahrestagung der Gesellschaft für Pädiatrische Radiologie Dresden 1994. Zentralblatt Radiologie 1994: 150: 876

96 Keilholz-George SD, Knight-Scott J, Berr SS. Theoretical analysis of the effect of imperfect slice profiles on tagging schemes for pulsed arterial spin labeling MRI. Magn Reson Med 2001: 46: 141-148

97 Kelly PJ, Hedley–Whyte ET, Primavera J, He J, GonzalezRG. Diffusion MRI in ischemic stroke compared to pathologically verified infarction. Neurology 2001: 56: 914-920

98 Kittner T, Appold S, Beuthien-Baumann B, Hietschold V, Laniado M, Baumann M. Tumorphysiologische Untersuchungen bei inoperablen pharyngealen Malignomen mittels MRT, PET und pO_2-Sondenmessung. Exp. Strahlentherapie und Klinische Strahlenbiologie 2002: 79-82. ISSN 1432-864X

99 Kjos BO, Ehman RL, Brant-Zawadzki M, Kelly WM, Norman D, Newton TH. Reproducibility of relaxation times and spin density calculated from routine MR imaging sequences: clinical study of the CNS. Am J Roentgenol 1985: 144:1165-1170

100 Klengel S, Hietschold V, Neumann U, Kaulen F, Köhler K. Quantitative mamma-MR of X-mammographic suspect postoperative status – Improved specificity combining early and late phase enhancement parameters. Proceedings of the Fourth Scientific Meeting of the ISMRM, New York, 27. 4. – 3. 5. 1996 S. 762

101 Klengel S, Hietschold V, Schreiber M, Köhler K. Quantitative kontrastmitteldynamische Mamma-MRT am 0,5-Tesla-Gerät. Röntgenpraxis 1994: 47: 223-228

102 Klose U, Nägele T, Friese S, Bitzer M. Charakteristische Größen bei der MR-Untersuchung der zerebralen Durchblutung mit hoher räumlicher und zeitlicher Auflösung. Fortschr Röntgenstr 1999: 170: 474-481

103 Knez A, Becker C, Becker A, Leber A, Haberl R, Reiser M, Steinbeck G. Bildgebende Verfahren in der Diagnostik des Herzes. Radiologe 2000:40:103-110

104 Knopp MV, Himmelhan N, Radeleff J, Junkermann H, Heß T, Sinn HP, Brix G. Methodenvergleich zur Quantifizierung der Kontrastmittelanreicherung am Beispiel der dynamischen MR-Mammographie. Radiologe 2002: 42: 280-290

105 Kobayashi H, Sato N, Kawamoto S, Saga T, Hiraga A, Ishimori T, Konishi J, Togashi K, Brechbiel MW. Novel intravascular macromolecular MRI contrast agent with generation-4 polyamidoamine dendrimer core: Accelerated renal excretion with coinjection of lysine.Magn Reson Med 2001: 46: 457-464

106 Kobayashi H, Shirakawa K, Kawamoto S, Saga T, Sato N, Hiraga A, Watanabe I, Heike Y, Togashi K, Konishi J, Grechbiel MW, Wakasugi H. Rapid accumulation and internalization of radiolabeled herceptin in an inflammatory breast cancer xenograft with vaculogenic mimicry predicted by the contrast-enhanced dynamic MRI with the macromolecular contrast agent G6-(1B4M-Gd)$_{256}$. Cancer Res 2002: 62: 860-866

107 Kreitner KF, Voigtlander T, Wittlinger T, Dahm M, Kalden P, Meyer J, Thelen M: Flussquantifizierung in Koronar- und Bypassgefäßen mit der MR-Phasenkontrasttechnik. Radiologe 2000:40:143-149

108 Kroft LJ, Doornbos J, Benderbous S, de Roos A: Equilibrium phase MR angiography of the aortic arch and abdominal vasculature with the blood pool contrast agent CMD-A2-Gd-DOTA in pigs. J Magn Reson Imaging 1999:9:777-785

109 Kucharczyk J, Vexler ZS, Roberts TP, Asgari HS, Mintorovitch J, Derugin N, Watson AD, Moseley ME. Echo-planar perfusion-sensitive MR imaging of acute cerebral ischemia. Radiology 1993: 188: 711-717

110 Kuhl CK, Bieling H, Gieseke J, Ebel T, Mielcarek P, Far F, Folkers P, Elevelt A, Schild HH. Breast neoplasms: T2* susceptibility-contrast, first-pass perfusion MR imaging. Radiology 1997: 202: 87-95

111 Kuhn MJ, Hammer GM, Swenson LC, Youssef HT, Gleason TJ. MRI evaluation of “solitary” brain metastases with triple-dose gadoteridol: comparison with contrast-enhanced CT and conventional-dose gadopentetate dimeglumine MRI studies in the same patients. Comput Med Imaging Graph 1994:18:391-399

112 Kvistad KA, Rydland J, Vainio J, Smethurst HB, Lundgren S, Fjosne H, Haraldseth O. Breast lesions: Evaluation with dynamic contrast-enhanced T1-weighted MR imaging and with T2*-weighted first-pass perfusion MR imaging. Radiology 2000: 216:545-553

113 Laniado M, Kopp AF. Gegenwärtiger Stand der klinischen Entwicklung von MR-Kontrastmitteln. Fortschr. Röntgenstr. 1997: 167: 541-550

114 Le Bihan D, Basser J: Molecular Diffusion and Nuclear Magnetic Resonance. in: Le Bihan D. (Ed.) Diffusion and Perfusion Magnetic Resonance Imaging. Applications to Functional MRI. New York 1995 ISBN 0-7817-0244-5

115 Leach MO. Application of magnetic resonance imaging to angiogenesis in breast cancer. Breast Cancer Res 2001: 3: 22-27

116 Li D, Dolan RP, Walovitch RC, Lauffer RB. Three-dimensional MRI of coronary arteries using an intravascular contrast agent. Magn Reson Med 1998:39:1014-1018

117 Liu HL, Pu Y, Liu Y, Nickerson L, Andrews T, Fox PT, Gao JH. Cerebral blood flow measurement by dynamic contrast MRI using singular value decomposition with an adaptive threshold. MRM 1999: 42: 167-172

118 Loeffler W, Weinmann HJ, Gries H, Speck U: Fundamental Physics and Chemistry.
In: in: Sartor K: MR Imaging of the Skull and Brain. Berlin 1992 ISBN 3-540-52293-X

119 Lythgoe DJ, Østergaard L, Williams SCR, Cluckie A, Buxton-Thomas M, Simmons A, Markus HS. Quantitative perfusion imaging in carotid artery stenosis using dynamic susceptibility contrast-enhanced magnetic resonance imaging. Magnetic Resonance Imaging 2000: 18: 1-11

120 Mayr NA, Yuh WT, Zheng J, Ehrhardt JC, Sorosky JI, Magnotta VA, Pelsang RE, Hussey DH. Tumor size evaluated by pelvic examination compared with 3-D quantitative analysis in the prediction of outcome for cervical cancer. : Int J Radiat Oncol Biol Phys 1997: 39: 395-404

121 McKinnon GC. Ultrafast interleaved gradient-echo-planar imaging on a standard scanner. MRM 1993:30:609-616

122 McLaughlin AC, Ye FQ, Berman KF, Mattay VS, Frank JA, Weinberger DR: Use of Diffusible and Nondiffusible Tracers in Studies of Brain Perfusion. In: Moonen CTW, Bandettini PA (Eds.) Functional MRI. Berlin 1999 ISBN 3-540-64263-3

123 Menon RS, Kim SG, Hu X, Owaga S, Ugurbil K. Functional MR imaging using the BOLD Approach. Field strength and sequence issues. In: Le Bihan D. (Ed.) Diffusion and Perfusion Magnetic Resonance Imaging. Applications to Functional MRI. New York 1995 ISBN 0-7817-0244-5

124 Miyati T, Banno T, Mase M, Kasai H, Shundo H, Imazawa M, Ohba S: Dual dynamic contrast-enhanced MR imaging. J Magn Reson Imaging. 1997; 7: 230-235

125 Mödder U, Mosny DS. Rationelle radiologische Diagnostik des Mammakarzinoms. PRAXIS 1998:87:499-503

126 Morneburg H (Ed.) Bildgebende Systeme für die medizinische Diagnostik. Publicis MCD. Erlangen 1995. ISBN89578-002-2

127 Müller-Schimpfle M, Ohmenhäuser K, Sand J, Stoll P, Claussen CD. Dynamic 3D-MR mammography: is there a benefit of sophisticated evaluation of enhancement curves for clinical routine? JMRI 1997: 7: 236-240

128 Neumann U, Klengel S, Kaulen F, Neumann H, Schorcht J, Köhler K. Dynamic doubl-echo perfusion study of cervix-carcinoma before and after radiation at a0.5 T routine scanner. 14th Annual Congress of the European Society for Magnetic Resonance in Medicine and Biology, Bruessel, 18. – 21. September 1997. Suppl. V, No II, S. 157

129 Niendorf HP, Haustein J, Louton T, Beck W, Laniado M. Safety and tolerance after intravenous administration of 0.3 mmol/kg Gd-DTPA. Results of a randomized, controlled clinical trial. Invest Radiol 1991:26 Suppl 1:S221-S223

130 Nitz W. Bildgebende Sequenzen in der Kernspintomographie und ihre klinische Anwendung.
Teil I. electromedica 1996: 64: 23-29;
Teil II. electromedica 1996: 64: 48-51;
Teil III. electromedica 1997: 65: 8-14

131 Nitz WR: MR Imaging: Acronyms and Clinical Applications. Eur.Radiol. 1999 9:979-997

132 Ostergaard L, Sorensen AG, Kwong KK, Weisskoff RM, Gyldensted C, Rosen BR. High resolution measurement of cerebral blood flow using intravaskulär tracer bolus passages. Part II: Experimental comparison and preliminary results. MRM 1996: 36: 726-736

133 Ostergaard L, Weisskoff RM, Chesler DA, Gyldensted C, Rosen BR. High resolution measurement of cerebral blood flow using intravascular tracer bolus passages. Part I: Mathematical approach and statistical analysis. MRM 1996 : 36 : 715-725.

134 Ozawa S, Imai Y, Suwa T, Kitajima M. What's new in imaging ? New magnetic resonance imaging of esophageal cancer using an endooluminal surface coil and antibody-coated magnetite particles. Recent Results Cancer Res 2000: 155: 73-87

135 Pantel J, Schröder J, Essig M, Jauss M, Schneider G, Eysenbach K, von Kummer R, Baudendistel K, Schad LR, Knopp MV. In vivo quantification of brain volumes in subcortical vascular dementia and Alzheimer's disease. Dement Geriatr Cogn Disord 1998: 9: 309-316

136 Pantel J, Schröder J, Schad LR, Friedlinger M, Knopp MV, Schmitt R, Geissler M, Blüml S, Essig M, Sauer H. Quantitative magnetic resonance imaging and neuropsychological functions in dementia of the Alzheimer type. Psychological Medicine 1997: 27: 221-229

137 Parker GJM, Suckling J, Tanner SF, Padhani AR, Revell PB, Husband JE, Leach MO. Probing tumor microvascularity by measurement, analysis and display of contrast agent uptake kinetics. JMRI 1997:7:564-574

138 Patlak CS, Blasberg RG, Fenstermacher JD. Graphical evaluation of blood-to-brain transfer constants from multiple-time uptake data. J Cereb Blood Flow Metab 1983: 3: 1-7

139 Perin EC, Silva GV, Sarmento-Leite R, Sousa AL, Howell M, Muthupillai R, Lambert B, Vaughn WK, Flamm SD. Assessing myocardial viability and infarct transmurality with left ventricular electromechanical mapping in patients with stable coronary artery disease: validation by delayed-enhancement magnetic resonance imaging. Circulation 2002: 106: 957-961

140 Ponnath MJ. Volumetrie mi der Kernspintomographie bei Ausgusspräparaten von Schweineherzen. Nuklearmedizinische Klinik und Poliklinik der TU München, Dissertation 1996

141 Prato FS, Drost DJ, King M, Keys T, Wisenberg G, Galland C, Pflugfelder PW. Cardiac T_1 calculations from MR spin-echo images. Magn Reson Med 1987: 4: 227-243

142 Reith W, Heiland S, Erb G, Benner T, Forsting M, Sartor K. Dynamic contrast-enhanced T2*-weighted MRI in patients with cerebrovascular disease. Neuroradiology 1997: 39: 250-257

143 Rempp K. Entwicklung einer MR-tomographischen Methode zur Quantifizierung der Mikrozirkulation im Gehirn. Dissertation Fakultät Physik der Universität (TH) Karlsruhe 1995

144 Rempp KA, Brix G, Wenz F, Becker CR, Gückel F, Lorenz WJ: Quantification of Regional Cerebral Blood Flow and Volume with Dynamic Susceptibility Contrast-enhanced MR Imaging. Radiology 1994; 193:637-641

145 Rieber A, Zeitler H, Rosenthal H, Görich J, Kreienberg R, Brambs HJ, Tomczak R. MRI of breast cancer: influence of chemotherapy on sensitivity. The British Journal of Radiology 1997: 70: 452-458

146 Rodewald A, Kittner T, Hietschold V, Platzbecker H. Nutzen der T2*-gewichteten Messung bei mammografisch benignen und malignitätsverdächtigen Läsionen. RöFo 2000: 172(Suppl) S36, VO130

147 Rosen BR, Belliveau JW, Chien D Perfusion imaging by nuclear magnetic resonance. Magn Reson Q. 1989 5: 263-281

148 Rosen BR, Belliveau JW, Vevea JM, Brady TJ. Perfusion imaging with NMR contrast agents. Magn Reson Med 1990:14:249-65

149 Rudat, V; Dietz, A; Schramm, O; Conradt, C; Maier, H; Flentje, M; Wannenmacer, M. Prognostic impact of total tumor volume and hemoglobin concentration on the outcome of patients with advanced head and neck cancer after concomitant boost radiochemotherapy. Radiotherapy and Oncology 1999:53:119-125

150 Rutland M, Que L, Hassan IM. „FUR“ – on size suits all. Eur J Nucl Med 2000: 27: 1708-1713

151 Sachs L. Angewandte Statistik. Springer Berlin 1992. ISBN 3-540-52085-6

152 Sakuma H, Goto M, Nomura Y, Kato N, Takeda K, Higgins CB. Three-dimensional coronary magnetic resonance angiography with injection of extracellular contrast medium. Invest Radiol 1999:34:503-508

153 Sakuma H, Takeda K, Higgins CB: Fast magnetic resonance imaging of the heart. Eur J Radiol 1999 :29 :101-113

154 Sanders JKM, Hunter BK. Modern NMR Spetroscopy: a guide for chemists. Oxford University Press 1994. ISBN 0-19-855567-9 (Pbk)

155 Sardanelli F, Molinari G, Zandrino F, Balbi M. Three-dimensional, navigator-echo MR coronary angiography in detecting stenoses of the major epicardial vessels, with conventional coronary angiography as the standard of reference. Radiology 214 (2000) 808-814

156 Schad LR, Blüml S, Zuna I. IX. MR Tissue Characterization of Intracranial Tumors by Means of Texture Analysis. MRM 1993:11:889-896

157 Schad LR, Brix G, Zuna I, Harle W, Lorenz WJ, Semmler W. Multiexponential proton spin-spin relaxation in MR imaging of human brain tumors. J Comput Assist Tomogr 1989: 13:577-587

158 Schmitt, T.; Gebauer, H.-D.; Freyer, R.; Franke, W.-G.; Oehme, L. Quantitative Auswertung nuklearmedizinischer Bilder auf der Grundlage einer fuzzy-basierten Segmentierung. BMT-Kongress, Zürich, Schweiz, 4.-7. September 1996. In: Boenick, U.; Schaldach, M. (Ed.): Vorträge der gemeinsamen Jahrestagung der Deutschen, Österreichischen und Schweizerischen Ges. für Biomedizinische Technik. Berlin: Schiele und Schön, 1996. S. 218-219

159 Scholdei R, Wenz F, Essig M, Fuss M, Knopp M. Simultane Bestimmung der Arteriellen Inputfunktion für die Dynamische Suszeptibilitätsgewichtete Magnetresonanztomographie aus der A. carotis interna und der A. cerebri media. Fortschr Röntgenstr 1999: 171: 38-43

160 Shahbazi-Gahrouei D, Williams M, Rizvi S, Allen BJ. In vivo studies of Gd-DTPA-monoclonal antibody and gd-porphyrins: potential magnetic resonance imaging contrast agents for melanoma. J Magn Reson Imaging. 2001: 14:169-174.

161 Simonsen CZ, Ostergaard L, Smith DF, Vestergaard-Poulsen P, Gyldensted C. Comparison of gradient- and spin-echo imaging: CBF, CBV, and MTT measurements by bolus tracking. : J Magn Reson Imaging 2000: 12: 411-416

162 Simpson NE, He Z, Evelhoch JL. Deuterium NMR tissue perfusion measurements using the tracer uptake approach: I. Optimization of methods. MRM 1999: 42: 42-52

163 Smith AM, Grandin CB, Duprez T, Mataigne F, Cosnard G. Whole brain quantitative CBF, CBV, and MTT measurements using MRI bolus tracking: implementaion and application to data acquired from hyperacute stroke patients. J Magn Reson Imaging. 2000:12: 400-10

164 Smith AM, Grandin CB, Duprez T, Mataigne F. Whole brain quantitative CBF and CBV measurements using MRI bolus tracking: Comparison of methodologies. MRM 2000: 43: 559-564

165 Stewart GN: Researches on the Circulation Time in Organs and on the Influences Which Affect It. J.Physiol. 1894; 15:1-89

166 Taylor AM, Keegan J, Jhooti P, Gatehouse PD, Firmin DN, Pennell DJ. A comparison between segmented k-space FLASH and interleaved spiral MR coronary angiography sequences. J Magn Reson Imaging 2000:4:394-400

167 Tofts PS, Berkowitz BA. Measurement of capillary permeability form the Gd enhacement curve: a comparison of bolus and constant infusion injection methods. Magn Reson Imaging 1994:12:81-91

168 Tofts PS, Shuter B, Pope JM. Ni-DTPA doped agarose gel – a phantom material for Gd-DTPA enhancement measurements. Magn Reson Imaging 1993: 11: 125-133

169 Toita T, Kakinohana Y, Shinzato S, Ogawa K, Yoshinaga M, Iraha S, Higashi M, Sakumoto K, Kanazawa K, Sawada S. Tumor diameter/volume and pelvic node status assessed by magnetic resonance imaging (MRI) for uterine cervical cancer treated with irradiation. : Int J Radiat Oncol Biol Phys 1999: 43: 777-782

170 Tweedle MF, Wedeking P, Telser J, Sotak CH, Chang CA, Kumar K, Wan X, Eaton SM: Dependence of MR Signal Intensity on Gd Tissue Concentration over a Broad Dose Range. Magn.Reson.Med.1991 22:191-194

171 Uematsu H, Maeda M, Sadato N, Matsuda T, Ishimori Y, Koshimoto Y, Yamda H, Kimura H, Kawamura Y, Matsuda T, Hayashi N, Yonekura Y, Ishii Y: Vascular Permeability: Quantitative measurement with double-echo dynamic MR imaging – theory and clinical application. Radiology 2000 214:912-917

172 Vonken EP, van Osch MJ, Bakker CJ, Viergever MA. Simultaneous quantitative cerebral perfusion and Gd-DTPA extravasation measurement with dual-echo dynamic susceptibility contrast MRI. Magn Reson Med 2000: 43: 820-827

173 Vonken EPA, Beekman FJ, Bakker CJG, Viergever MA. Maximum likelihood estimation of cerebral blood flow in dynamic susceptibility contrast MRI. MRM 1999:41: 343-350

174 Walker PM, Mario PY, Mezeray C, Bessiores M, Escanyé JM, Karcher G, Danchin N, Mattel S, Villemot JP, Bertrand A. Synchronized inversion recovery-spin echo sequences for precise in vivo T_1 measurement of human myocardium: a pilot study of 22 healthy subjects. Magn Reson Med 1993: 29: 637-641

175 Wehrli FW Fast-scan magnetic resonance: Principles and applications. Raven Press New York (1991) ISBN 0-88167-746-9

176 Wehrli FW, McGowan JC. The Basis of MR Contrast. In: Atlas SW (Ed.)´Magnetic Resonance Imaging of the Brain and Spine. Lippincott – Raven Philadelphia. ISBN 0-7817-0282-8

177 Weinmann HJ, Gries H, Speck U: Contrast Agents.
in: Sartor K: MR Imaging of the Skull and Brain. Berlin 1992 ISBN 3-540-52293-X

178 Weinmann HJ, Laniado M, Mutzel W. Pharmacokinetics of Gd-DTPA/dimeglumine after intravenous injection into healthy volunteers. Phys Chem Phys Med NMR 1984:16:167

179 Weinmann HJ. Characteristics of Gd-DTPA dimeglumine. In: Felix R, Heshiki A, Hosten N, Hricak H: Magnevist. London 1994 ISBN 0 86542 836 0

180 Weisskoff RM, Zuo CS, Boxerman JL, Rosen BR. Microscopic susceptibility variation and transverse relaxation: Theory and experiment. MRM 1994: 31: 601-610

181 Weissmantel C, Lenk R, Forker W, Ludloff R, Hoppe J (Eds.): Atom. Struktur der Materie. Leipzig 1970

182 Wenz F, Rempp K, Hess T, Debus J, Brix G, Engenhart R, Knopp MV, van Kaick G, Wannenmacher M. Effect of radiation on blood volume in low-grade astrocytomas and normal brain tissue: quantification with dynamic susceptibility contrast MR imaging. Am J Roentgenol 1996:166: 187-93

183 Wilson JM, Villareal RP, Hariharan R, Massumi A, Muthupillai R, Flamm SD. Magnetic resonance imaging of myocardial fibrosis in hypertrophic cardiomyopathy. Tex Heart Inst J 2002: 29:176-180

184 Wirestam R, Ryding E, Lindgren A, Geijer B, Holtas S, Stahlberg F. Absolute cerebral blood flow measured by dynamic susceptibility contrast MRI: a direct comparison with Xe-133 SPECT. MAGMA 2000: 11: 96-103

185 Wofram Research Inc. The Integrator. http://integrals.wolfram.com (19.10.2002)

186 Wolf M, Weber O, Keel M, Golay X, Scheidegger M, Bucher HU, Kollias S, Boesiger P, Banziger O. Comparison of cerebral blood volume measured by near infrared spectroscopy and contrast enhanced magnetic resonance imaging. Adv Exp Med Biol 1999: 471: 767-73

187 Ye FQ, Frank JA, Weinberger DR, McLaughlin AC. Noise reduction in 3D perfusion imaging by attenuating the static signal in arterial spin tagging (ASSIST). Magn Reson Med 2000: 44: 92-100

188 Yu X, Song SK, Chen J, Scott MJ, Fuhrhop RJ, Hall CS, Gaffney PJ, Wickline SA, Lanza GM. High-resolution MRI characterization of human thrombus using a novel fibrin-targeted paramagnetic nanoparticle contrast agent. Magn Reson Med. 2000: 44: 867-872.

189 Zheng J, Bae KT, Woodard PK, Haacke EM, Li D. Efficacy of slow infusion of gadolinium contrast agent in three-dimensional MR coronary artery imaging. J Magn Reson Imaging 1999:10:800-805

Verzeichnis der verwendeten Symbole

α	Flipwinkel, Pulswinkel (Winkel, um den die Nettomagnetisierung durch einen RF-Impuls um eine durch die Richtung des Magnetfeldvektors des RF-Feldes gedreht wird)
γ	Gyromagnetisches Verhältnis
χ	Magnetische Suszeptibilität. Sie entspricht der um 1 verminderten relativen magnetische Permeabilität μ_r. Diese wiederum ist der material-abhängige Anteil des Proportionalitätsfaktors zwischen magnetischer Feldstärke H und magnetischer Induktion B<br>$B = \mu_r * \mu_0 * H$
μ	magnetisches Moment
B_0	Magnetische Induktion des statischen Magnetfeldes
B_1	Magnetische Induktion des RF-Feldes, senkrecht zu B_0
C	Kontrastmittel-Konzentration
E	Energie
F	Blutfluss (Volumen pro Zeit)
f, ω	Frequenz, $\omega = f * 2\pi$
G	Gradientenfeldstärke
h, $\hbar$	Plancksches Wirkungsquantum, $\hbar = h/2\pi$, $h = 6.6*10^{-34}$ Js
k	Boltzmann-Konstante $1.3805*10^{-23}$ J/K
M	Nettomagnetisierung
m	Masse, speziell Menge des injizierten Kontrastmittels
m_I	magnetische Quantenzahl
PS	Permeability surface area product<br>Durchlässigkeit der Wände der Blutgefäße innerhalb eines Gewebes für eine bestimmte Substanz
r	Relaxivität (Veränderung der Relaxationsrate durch eine Substanz)
R_1, R_2	Relaxationsrate (Reziprokes der Relaxationszeit) $R_i = 1/T_i$
S	Signalintensität, $S(t)$ in Abhängigkeit von der Zeit
sinc	$\mathrm{sinc}(z) = \frac{\sin(z)}{z}$

T	absolute Temperatur
t	Zeit
T_1	Spin-Gitter-Relaxationszeit
T_2	Spin-Spin-Relaxationszeit,<br>(T_2^* unter Einschluss suszeptibilitätsbedingter Dephasierung)
T_E	Echozeit
T_R	Repetitionszeit

Verzeichnis der verwendeten Begriffe und Abkürzungen

2D-Messung	schichtselektive RF-Anregung, zweidimensionale FFT; ggflls. Zeitlich ineinander verschachtelte Messung mehrerer Schichten
3D-Messung	gleichzeitige RF-Anregung des gesamten Messvolumens; dreidimensionale FFT
ADC	MR-Technik: Analog-Digital-Konverter<br>Diffusions-Bildgebung: Apparent Diffusion Coefficient
AIF	Arterielle Inputfunktion
Artefakt	hier: Bildstörung.<br>Struktur im MR-Bild, die an dieser Stelle keine Entsprechung am dargestellten Objekt hat
benigne	gutartig
Bestimmtheitsmaß	Quadrat des Korrelationskoeffizienten. Anteil der durch den linearen Zusammenhang y(x) erklärten Streuung an der gesamten Streuung σ_y
BOLD	Blood oxygenation level dependent (fMRI)
Bolus-Injektion	Im Gegensatz zur Infusion wird das Medikament (hier: Kontrastmittel) in relativ kurzer Zeit mit hoher (durch die Verträglichkeit begrenzter) Konzentration injiziert
CNR	Contrast to noise ratio<br>Kontrast-zu-Rausch-Verhältnis
CT	(Röntgen-) Computertomographie
DSA	Digitale Subtraktionsangiographie
EPI	Echo Planar Imaging<br>Sehr schnelle Bildgebungssequenz, bei der viele (oft alle) Phasencodierschritte eines Bildes nach nur einer Anregung unter schrittweiser oder kontinuierlicher Erhöhung des Integrals des Phasencodiergradienten (d.h. ohne zwischenzeitliche Rewinder-Gradienten) aufgenommen werden. Sehr anfällig gegenüber Suszeptibilitäts- und anderen Artefakten
Feldstärke	exakt: magnetische Feldstärke (Maßeinheit A/m)<br>In der vorliegenden Arbeit wird – wie in der kernspintomographischen Literatur üblich – der Begriff Magnetfeldstärke auch für die magnetische Induktion (Maßeinheit Tesla) benutzt
FFE<br>FLASH	Fast Field Echo<br>Fast Low Angle Shot<br>Gradientenecho-Sequenz mit kleinem Anregungswinkel
FFT	Fast Fourier Transform<br>Schnelle Fourier-Transformation (vgl. z. B. [19] S. 669)

FID	Free induction decay<br>Abklingen der unmittelbar nach Einstrahlung eines Anregungs-RF-Impulses bestehenden Nettomagnetisierung in der xy-Ebene (senkrecht zum Hauptmagnetfeld)
first pass, second pass	Erster bzw. zweiter Durchlauf eines Kontrastmittel-Bolus durch die untersuchte Gewebsregion
FLR	fractional leak rate<br>Anteil des Kontrastmittels (allgemein: des Tracers), der bei Passage durch ein Gewebe aus dem Blut in das Gewebe übertritt
fMRI	functional Magnetic Resonance Imaging; vorwiegend für Darstellungen der Hirnfunktion gebräuchlicher Begriff
FOV	Field of View
Gd-DTPA	Gadolinium diethylenetriaminepentaacetic acid
Gradient; Gradienten-feld	im Kontext der MR-Bildgebung und Spektroskopie ein dem Hauptmagnetfeld überlagertes, in einer Raumrichtung linear ortsabhängiges Magnetfeld parallel bzw. antiparallel zum Hauptmagnetfeld
Hämatokrit	Volumenanteil der Erythrozyten im Blut
IDT	Indicator Dilution Theory<br>Indikator-Verdünnungs-Theorie
IR	Inversion Recovery
KM	Kontrastmittel
k-Raum<br>k space	Datenraum, der die unverarbeiteten Messwerte eines MRT-Datensatzes enthält.<br>Das (zwei- oder höherdimensionale) MRT-Bild ist die Fourier-Transformierte des k-Raumes
Longitudinal-Magneti-sierung	Netto-Magnetisierung parallel zum Hauptmagnetfeld B_0. Beschreibung von deren Rückkehr in den Grundzustand durch T_1-Relaxationszeit
Magnetische Induktion	magnetische Flussdichte (Maßeinheit Tesla). In der kernspintomographischen Literatur wird hierfür auch der Begriff Feldstärke verwendet
maligne	bösartig
MRI, MRT	Magnetic Resonance Imaging<br>Magnetresonanztomographie<br>Kernspintomographie
MRM	Magnetic Resonance Mammography

MTT	Mean Transit Time (charakteristische Zeit für die Passage eines Kontrastmittel-Bolus durch ein Gewebe)
p.i.	post injectionem<br>nach Injektion (des Kontrastmittel-Bolus)
Partial-volumen-effekt	Signalintensität eines Bildelements ist der Mittelwert der Intensitäten aller durch das Pixel erfassten Gewebe (hier: Vernachlässigung von Suszeptibilitätseffekten).
PET	Positronen-Emissions-Tomographie
Proton; Protonen-dichte	im Kontext der MR-Bildgebung und –Spektroskopie werden mit diesem Begriff nur die zum Signal beitragenden Wasserstoffkerne betrachtet (hinreichend frei bewegliche Wasserstoff-Ionen außerhalb des thermischen Gleichgewichtes gemäß Boltzmann-Verteilung)
rBF	relativer Blutfluss (Volumenprozent pro Zeit)
rBV	relatives Blutvolumen (Anteil des Blutgefäß-Lumens am Gesamtvolumen eines Volumenelementes)
RF	Radio frequency<br>elektromagnetische Hochfrequenz, Rundfunkfrequenz
SAR	Specific Absorption Rate
SE	Spinecho
Segmenta-tion	hier: Abgrenzung der interessierenden Region von ihrer Umgebung anhand von Signalintensitäts-Unterschieden
Sensitivität	Verhältnis richtig positiver zu (richtig positiver + falsch negativer) Testergebnisse
SNR	Signal to noise ratio<br>Signal-zu-Rausch-Verhältnis
SPECT	Single Photon Emission Computed Tomography (dreidimensionale Verallgemeinerung der Szintigraphie)
Spezifität	Verhältnis richtig negativer zu (richtig negativer + falsch positiver) Testergebnisse
SPIO	Superparamagnetic iron oxide (durch Umhüllung mit z. B. Dextran biologisch verträglich gemachte Eisenoxyd-Partikel von ca. 15 ... 200 nm Durchmesser)
STIR	Short Tau Inversion Recovery<br>IR-Sequenz, bei der die Inversionszeit T_I so gewählt wird, dass zum Auslese-Zeitpunkt des Signals die z-Magnetisierung von Fettgewebe und damit dessen Beitrag zum Signal verschwindet

SUV

$$SUV = \frac{activity\ in\ ROI[Bq/g] * body\ weight[g]}{injected\ activity[Bq]}$$

Standard Uptake Value (Aktivität des Tracers in einer ROI, bezogen auf die injizierte Aktivität)

SVD — Single Value Decomposition
algebraischer Ansatz, der hier zur „Entfaltung“ intraarterieller KM-Konzentrationskurven von der Arteriellen Inputfunktion benutzt wird

TOF Time of Flight — Signalreiche Darstellung durchströmter Blutgefäße, indem stationäres Gewebe durch relativ große Flipwinkel bei kurzer Repetitionszeit abgesättigt wird. Das einströmende Blut ist nicht vorgesättigt und damit signalreicher.

Transversal-Magnetisierung — Netto-Magnetisierung in der Ebene senkrecht zum Hauptmagnetfeld B_0. Beschreibung von deren Rückkehr in den Grundzustand durch T_2- bzw. T_2^*-Relaxationszeit

TTP — Time to Peak
Zeit bis zum Erreichen der maximalen Kontrastmittel-Konzentration

Turbo — Variante einer Standardsequenz, in der nach einer RF-Anregung der Phasencodier- und Ausleseteil (einschließlich Rephasierungen) mehrfach wiederholt wird, um innerhalb einer Repetitionszeit T_R mehrere Zeilen des k-Raumes zu füllen.

Danksagung

Allen am Zustandekommen dieser Arbeit Beteiligten möchte ich mich an dieser Stelle meinen herzlichen Dank aussprechen.

Herrn Prof. Dr. Rüdiger Poll danke ich sehr herzlich für die Übernahme der Erstbegutachtung und seine vielfältigen Hinweise.

Besonders erwähnen möchte ich an dieser Stelle die ehemaligen Direktoren des Institutes für Radiologische Diagnostik am Universitätsklinikum der TU Dresden, die Professoren Klaus Köhler und Heinrich Platzbecker, die das Entstehen der Arbeit anregten, Freiräume für die Forschungsarbeit gewährten und auch Gelegenheit schafften, deren Ergebnisse national und international zu präsentieren. An Herrn Prof. Michael Laniado darf ich diesen Dank für die Zeit seit seinem Eintritt in dieses Amt ebenso richten.

Zu großem Dank verpflichtet bin ich meinen medizinischen Partnern, von denen ich besonders den Doktoren Nasreddin Abolmaali, Gabriele Hahn, Thomas Kittner, Steffen Klengel und Uwe Neumann für viele wertvolle Anregungen, ergiebige Diskussionen, aber auch die Betreuung bzw. Durchführung der Messungen an Patienten danke. Frau Michelle Schreyer und Herrn Dr. Sebastian Schwensow gilt mein besonderer Dank für ihre umfangreiche Auswertearbeit im Rahmen ihrer Dissertationen. Den Doktoren Bettina Beuthien-Baumann und Steffen Appold danke ich für die Bereitstellung ihrer Messergebnisse im Rahmen der Pharynx-Studie.

Die Messungen hätten ohne die bereitwillige Unterstützung des mittleren medizinischen Personals – hier möchte ich stellvertretend für viele andere Frau Annett Stolze und Frau Mandy Lungwitz erwähnen – deren Geduld und investierte Zeit nicht im vorliegenden Maße zustande kommen können. Auch ihnen gilt mein herzlicher Dank.

Frau Ulrike Dänhardt danke ich sehr für die Hilfe bei Literaturrecherchen und die Beschaffung einer Vielzahl der zitierten Quellen.

Eine Vielzahl von Personen war indirekt am Zustandekommen der Arbeit beteiligt. Hier möchte ich wiederum stellvertretend für viele andere Frau Annett Sichting dafür danken, dass sie durch Übernahme von Arbeiten speziell der Betreuung der Datenverarbeitung im Institut hardware-, software- und nutzerseitig mir einen erheblichen Zeitfonds für Forschungsarbeiten zur Verfügung gestellt hat.

Nicht zuletzt möchte ich meiner Frau Elke sowie meinen Töchtern Nadine und Philine dafür danken, dass sie viel Geduld und Verständnis für die im Zusammenhang mit dem Erstellen dieser Arbeit verbundenen Belastungen aufgebracht haben.

V. Hietschold

Dresden, im Mai 2004
